# Synthesis of *N*-Protected 1-Aminoalkylphosphonium Salts from Amides, Carbamates, Lactams, or Imides

**DOI:** 10.1021/acs.joc.1c00285

**Published:** 2021-04-08

**Authors:** Jakub Adamek, Paulina Zieleźny, Karol Erfurt

**Affiliations:** † Department of Organic Chemistry, Bioorganic Chemistry and Biotechnology, Silesian University of Technology, B. Krzywoustego 4, 44-100 Gliwice, Poland; ‡ Biotechnology Center of Silesian University of Technology, B. Krzywoustego 8, 44-100 Gliwice, Poland; § Department of Chemical Organic Technology and Petrochemistry, Silesian University of Technology, B. Krzywoustego 4, 44-100 Gliwice, Poland

## Abstract

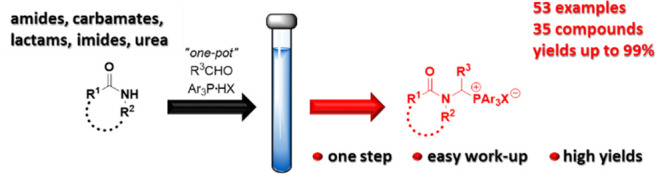

This report describes the development and optimization of the one-pot method for the synthesis of *N*-protected 1-aminoalkylphosphonium salts based on the three-component coupling of aldehydes and either amides, carbamates, lactams, imides, or urea in the presence of triarylphosphonium salts. The proposed strategy is very efficient and easy to carry out even on a larger scale (20 g) in any typical laboratory. Most reactions occur at temperatures between 50 and 100 °C in a short time (1–2 h) without requiring any catalyst, and simple workup procedures afford good to excellent yields. The exceptions are condensations with imides, which require much higher temperatures (150–170 °C) and longer reaction times (even 30 h). The possibility of carrying out the synthesis under solvent-free conditions (neat reactions) is also demonstrated. It is especially important for less reactive substrates (imides), and reactions required high temperature (or generally harsher conditions). Finally, we prove the developed one-pot methodology can be successfully applied for the synthesis of structurally diverse *N*-protected 1-aminoalkylphosphonium salts. Mechanistic studies showed the intermediate products of described couplings are 1-hydroxyalkylphosphonium salts, not *N*-hydroxyalkylamides, -imides, etc., as initially expected.

## Introduction

Phosphonium salts comprise a class of organic compounds that has enjoyed unwavering interest from the chemistry community for decades because of their applicability as reagents (e.g., ylide precursors), catalysts, or solvents (e.g., phosphonium ionic liquids (PILs)) in the synthesis of biologically active compounds.^1−8^

Certain structural features of *N*-protected 1-aminoalkylphosphonium salts make them a very interesting and promising type of phosphonium compounds; however, their synthetic potential has not yet been fully elucidated. The presence of an acylamino group next to a positively charged phosphonium moiety permits the use of *N*-protected 1-aminoalkylphosphonium salts as very effective α-amidoalkylating agents (i.e., precursors of *N*-acylimines or *N*-acyliminium cations) in the α-amidoalkylation reaction. It has been demonstrated that under appropriate conditions, *N*-protected 1-aminoalkylphosphonium salts readily react with both carbon- and heteronucleophiles, leading to the formation of new C–C and C–heteroatom bonds, respectively.^9−12^ Moreover, the reactivity of these salts can be improved by imposing some structural modifications, especially within the phosphonium group. The introduction of electron-withdrawing substituents (e.g., Cl, CF_3_) into the phosphonium moiety weakens the C_α_–P^+^ bond, thereby facilitating its cleavage and promoting the generation of iminium-type cations, which are the proper α-amidoalkylating agents. This phenomenon highlights the possibility of conducting catalyst-free α-amidoalkylation,^13,14^ which is an interesting alternative or complementary approach to those already described in the literature (mostly acid-catalyzed reactions).^15−22^

**Scheme 1:**
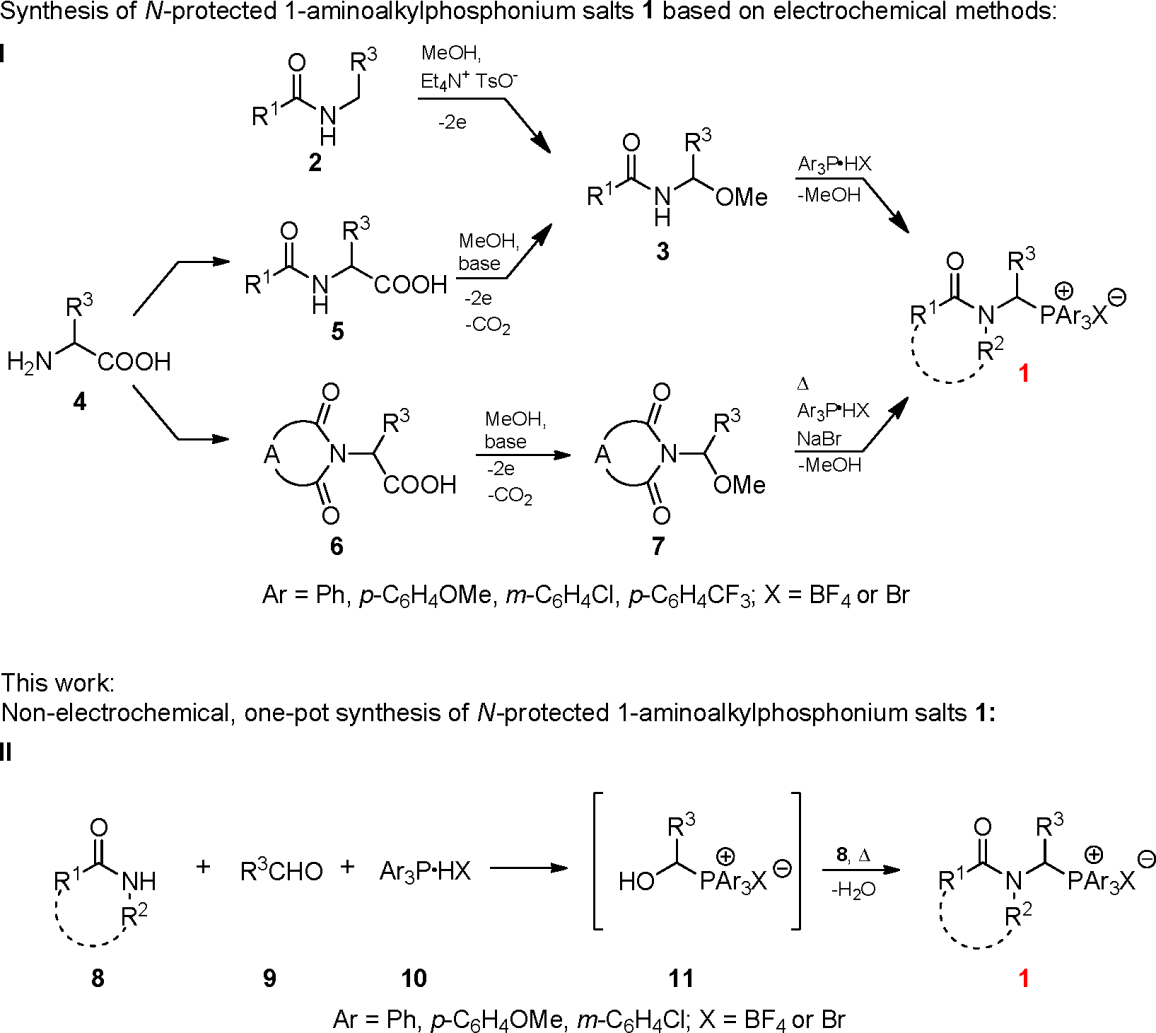
Various Synthetic Routes for Producing *N*-Protected 1-Aminoalkylphosphonium Salts

The most significant challenges regarding the use of phosphonium salts on a large scale involve difficulties with their preparation. So far, the most important methods are based on electrochemical alkoxylation (Scheme 1/I), which is very efficient (especially for electrochemical decarboxylative α-alkoxylation of α-amino acid derivatives **5**) but requires additional, sometimes expensive equipment and basic knowledge of electrochemistry.^23−35^ Therefore, synthetic chemists are often reluctant to employ such strategies. There are several other interesting methods for the synthesis of *N*-protected 1-aminoalkylphosphonium salts described in the literature.^36−39^ However, in most cases, they are multistep, time- and labor-consuming, and have a narrow scope of application, which in practice is limited to *N*-acylaminomethylphosphonium salts (see also Table S1, Supporting Information).^20^

To overcome these challenges, we have developed a novel, nonelectrochemical method for the preparation of *N*-protected 1-aminoalkylphosphonium salts. The proposed synthetic strategy is based on the one-pot reaction between aldehydes and either amides, carbamates, lactams, imides, or urea in the presence of triarylphosphonium tetrafluoroborates or bromides (Scheme 1/II).

## Results and Discussion

**Scheme 2:**
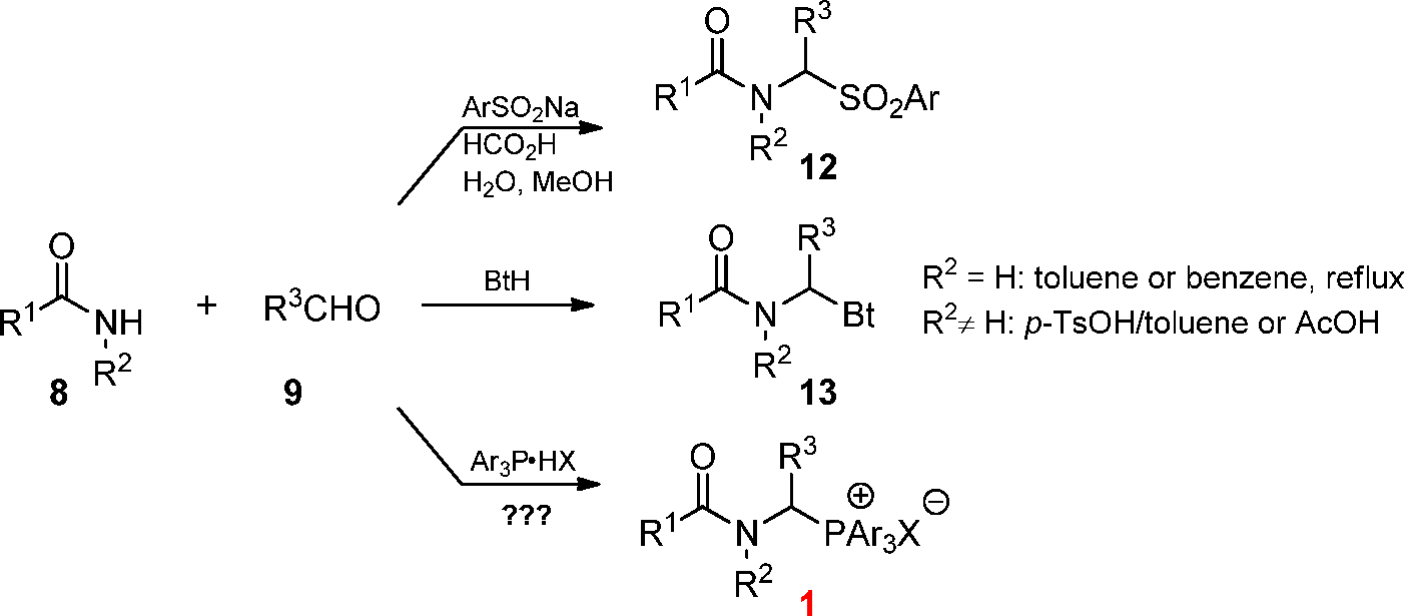
Three-Components Couplings Used for the Synthesis of α-Amido Sulfones **12** or *N*-[1-(Benzotriazo-1-yl)alkyl]amides **13**

During the search for a new, general method for the preparation of *N*-protected 1-aminoalkylphosphonium salts, we turned our attention to the three-component condensations used for the synthesis of structurally related α-amido sulfones **12** or *N*-[1-(benzotriazo-1-yl)alkyl]amides **13** (Scheme 2).^16−18^

The possibility of obtaining phosphonium salts directly from aldehydes and amides (carbamates, etc.) in the presence of an appropriately designed phosphorus-containing component, using a one-pot methodology seems very promising. Therefore, we selected the condensation of propionaldehyde, acetamide, and triphenylphosphonium tetrafluoroborate (in the molar ratio of 1:1:1) as the model reaction, and then performed it under various conditions (see Table 1).

**Table 1:**
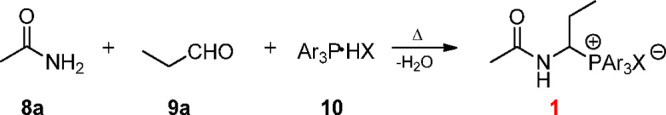
Reactions of Acetamide with Propionaldehyde in the Presence of Triarylphosphonium Salts: Optimization Studies

	phosphonium salts **1**						
entry	Nr	X	Ar	solvent	time (h)	temp. (°C)	yield^a^ (%)
1	**1a**	BF_4_	Ph	CH_3_CN	48	r.t.	87
2	**1a**	BF_4_	Ph	CH_3_CN	1	50	84
3	**1a**	BF_4_	Ph	CHCl_3_	1	50	86
4	**1a**	BF_4_	Ph	THF	1	50	74
5	**1a**	BF_4_	Ph	–	1	50/70	50^b^/78^c^
6	**1a**	BF_4_	Ph	CH_3_CN	1	50	49^d^
7	**1b**	Br	Ph	CH_3_CN	1	50	70
8	**1b**	Br	Ph	–	1	70	57^c^
9	**1c**	BF_4_	*m*-C_6_H_4_Cl	CH_3_CN	1	50	70
10	**1c**	BF_4_	*m*-C_6_H_4_Cl	CHCl_3_	1	50	71
11	**1c**	BF_4_	*m*-C_6_H_4_Cl	–	1	70	traces^c^

a Isolated yields.

b The yield was estimated based on the ^1^H NMR spectrum; Attempts to isolate the pure product **1a** failed due to low conversion of substrates.

c The molar ratio of substrates equals 1.2(aldehyde):1:1.

d Triphenylphosphine and HBF_4_ (tetrafluoroboric acid diethyl ether complex) were used instead of triphenylphosphonium tetrafluoroborate.

Preliminary investigations indicated that the expected transformation occurred in acetonitrile at room temperature, although, when the reaction temperature was raised to 50 °C, the reaction time decreased from 48 to 1 h (compare entries 1 and 2, Table 1). Changing the solvent to CHCl_3_ or THF did not appreciably affect the course of the reaction (entries 3 and 4, Table 1). The one-pot transformation can be carried out in a solvent-free environment; however, a slight excess of propionaldehyde is required, relative to the amide in these cases (molar ratio of 1:1.2; entries 5 and 8, Table 1). It is also preferred to raise the temperature to 70 °C because at 50 °C the reaction is slow (see entry 5, Table 1).

Besides, it was confirmed that *N*-acylaminoalkylphosphonium salt **1a** can be obtained using triphenylphosphine in the presence of HBF_4_ (tetrafluoroboric acid diethyl ether complex) instead of triphenylphosphonium tetrafluoroborate, but the yield of the reaction is much lower (49% vs 84%; compare entries 6 and 2, Table 1). Furthermore, it was demonstrated other triarylphosphonium salts including triphenylphosphonium bromide and tris(3-chlorophenyl)phosphonium tetrafluoroborate can be used in the synthesis (entries 7–10, Table 1). However, it seems the solventless methodology may have some limitations here (entry 11, Table 1).

Next, to evaluate the scope of the developed methodology, we conducted reactions between selected amides (entries 1–19, Table 2), carbamates (entries 20–29, Table 2), imides (entries 35–41), and structurally diverse, simple or functionalized aldehydes in the presence of various triarylphosphonium salts. We also checked the possibility of using lactams (on the example of butyrolactam; entries 30–33, Table 2) and urea (entry 34, Table 2) as the nitrogen-containing component.

**Table 2:**
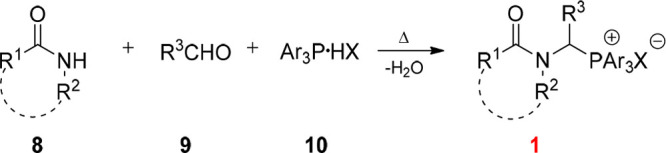

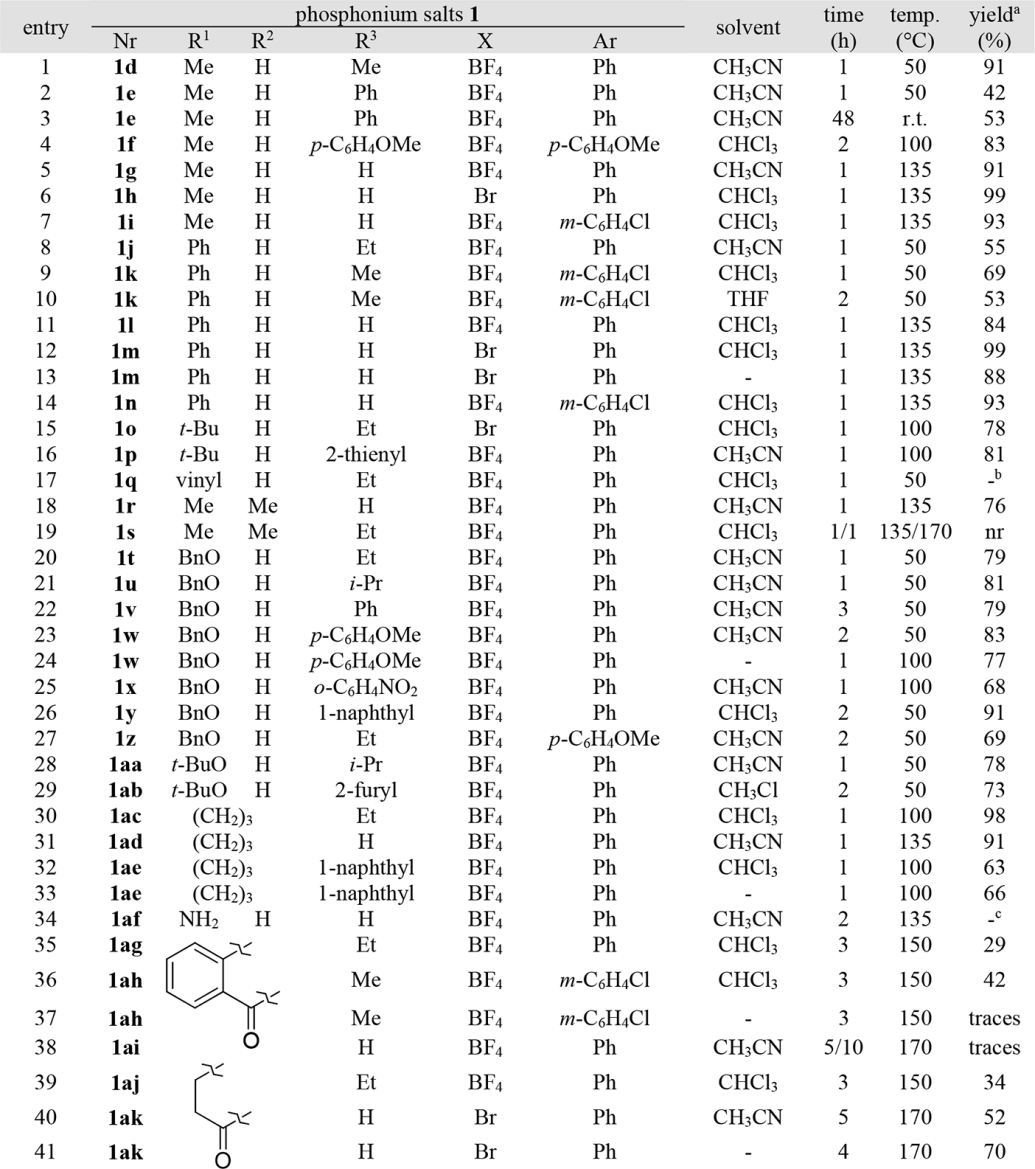
Conditions and Yields for the One-Pot Synthesis of *N*-Protected 1-Aminoalkylphosphonium Salts from Amides, Carbamates, Lactams, Imides, or Urea

a Isolated yields.

b The main reaction product is 2-carbamoylethyltriphenylphosphoniumtetrafluoroborate **14** (78%).

c The main reaction product is 1,1′-(carbonyldimino)bis(methyltriphenylphosphonium) bis(tetrafluoroborate) **15** (84%; the molar ratio of substrates equals 1(urea): 2:2).

In general, aliphatic and aromatic (simple and functionalized) aldehydes, as well as paraformaldehyde, can be used in the one-pot reaction with good results. However, in the case of paraformaldehyde, it was necessary to increase the reaction temperature to 135 °C in order to obtain sufficiently high yields.

The proposed one-pot methodology enables the production of triphenylphosphonium salts (Ar = Ph) as well as phosphonium salts, which are derivatives of triarylphosphines substituted with electron-donating or electron-withdrawing substituents (Ar = *p*-C_6_H_4_OMe or *m*-C_6_H_4_Cl; see, e.g., entries 4, 7, 9, or 36 in Table 2). The type of substituent influences the strength of the C_α_–P^+^ bond, which has a significant impact on the reactivity of the obtained compounds, especially in the α-amidoalkylation-type reaction.^13,14,24^

Amides, carbamates, and lactams react with aldehydes under mild conditions, even at room temperature; however, a temperature of 50–100 °C is usually required.

**Scheme 3:**
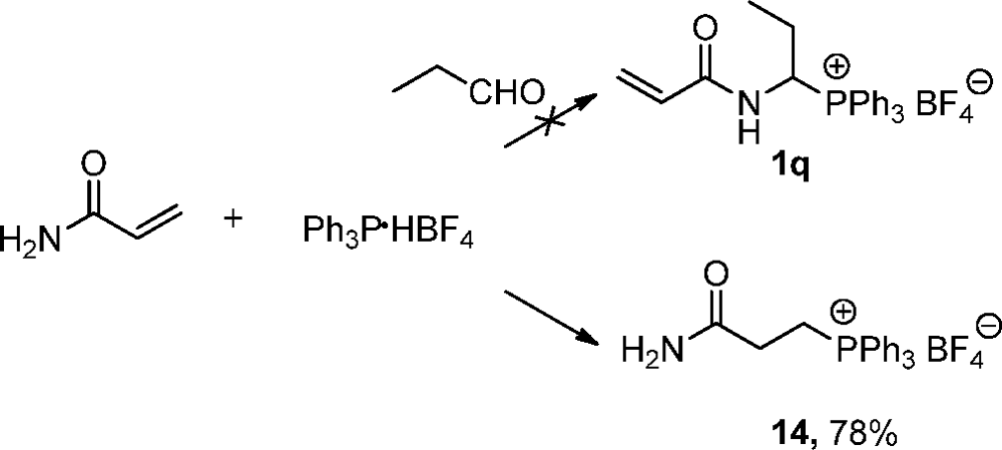
Formation of 2-Carbamoylethyltriphenylphosphonium Tetrafluoroborate **14** in the Reaction of Acrylamide and Triphenylphosphonium Tetrafluoroborate

α,β-Unsaturated amides such as acrylamide in the presence of triphenylphosphonium tetrafluoroborate give a 1,4 electrophilic addition product, e.g., 2-carbamoylethyltriphenylphosphoniumtetrafluoroborate **14** (Scheme 3).

**Scheme 4:**
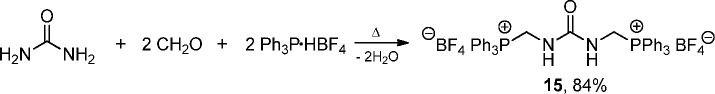
Reaction of Urea with Formaldehyde (Generated in Situ from Paraformaldehyde) in the Presence of Triphenylphosphonium Tetrafluoroborate

Urea, in turn, reacts with paraformaldehyde and triphenylphosphonium tetrafluoroborate (in the molar ratio of 1(urea):2:2) to form a bisphosphonium salt **15** (Scheme 4). When the molar ratio of substrates was 1:1:1, a mixture of phosphonium salts (the major product is bisphosphonium salt **15**) was obtained, but attempts to separate them failed.

Couplings with imides required high temperatures (150–170 °C), which promoted undesirable side reactions and relatively low yields of the products (entries 35–41, Table 2). It seems that one of the crucial factors here is the lower nucleophilicity of the nitrogen in imides compared to amides (see mechanistic studies, vide infra).

Finally, we have explored the possibility of conducting the reaction under solvent-free conditions (neat reactions). This methodology is very useful for less reactive substrates (imides and paraformaldehyde) requiring harsher reaction conditions (compare entries 40 and 41, Table 2). It is also important from the safety point of view because of high pressure in the reaction system when solvents are present (reactions with or without solvents are carried out in screw cap vials; see Experimental Section). Unfortunately, it was confirmed that the solventless procedure can not be used for the preparation of *N*-protected 1-aminoalkylphosphonium salts, which are derivatives of phosphines substituted with electron-withdrawing substituents (see entry 11, Table 1 and entry 37, Table 2).

**Scheme 5:**
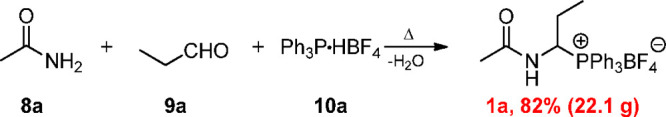
Reaction of Acetamide with Propionaldehyde in the Presence of Triphenylphosphonium Tetrafluoroborate on the 20 g-Scale

In order to present the high practical utility of the developed methodology, we conducted the synthesis of phosphonium salt **1a** on a larger 20 g-scale (Scheme 5). The reaction was carried in a round-bottom flask equipped with a reflux condenser. During the addition of the substrates, the mixture was cooled using an ice–water bath. After that, the reaction mixture was heated at 50 °C for 2 h (after 1h the conversion was 85%). Finally, we isolated over 22 g of product **1a** in 82% yield.

**Figure 1:**
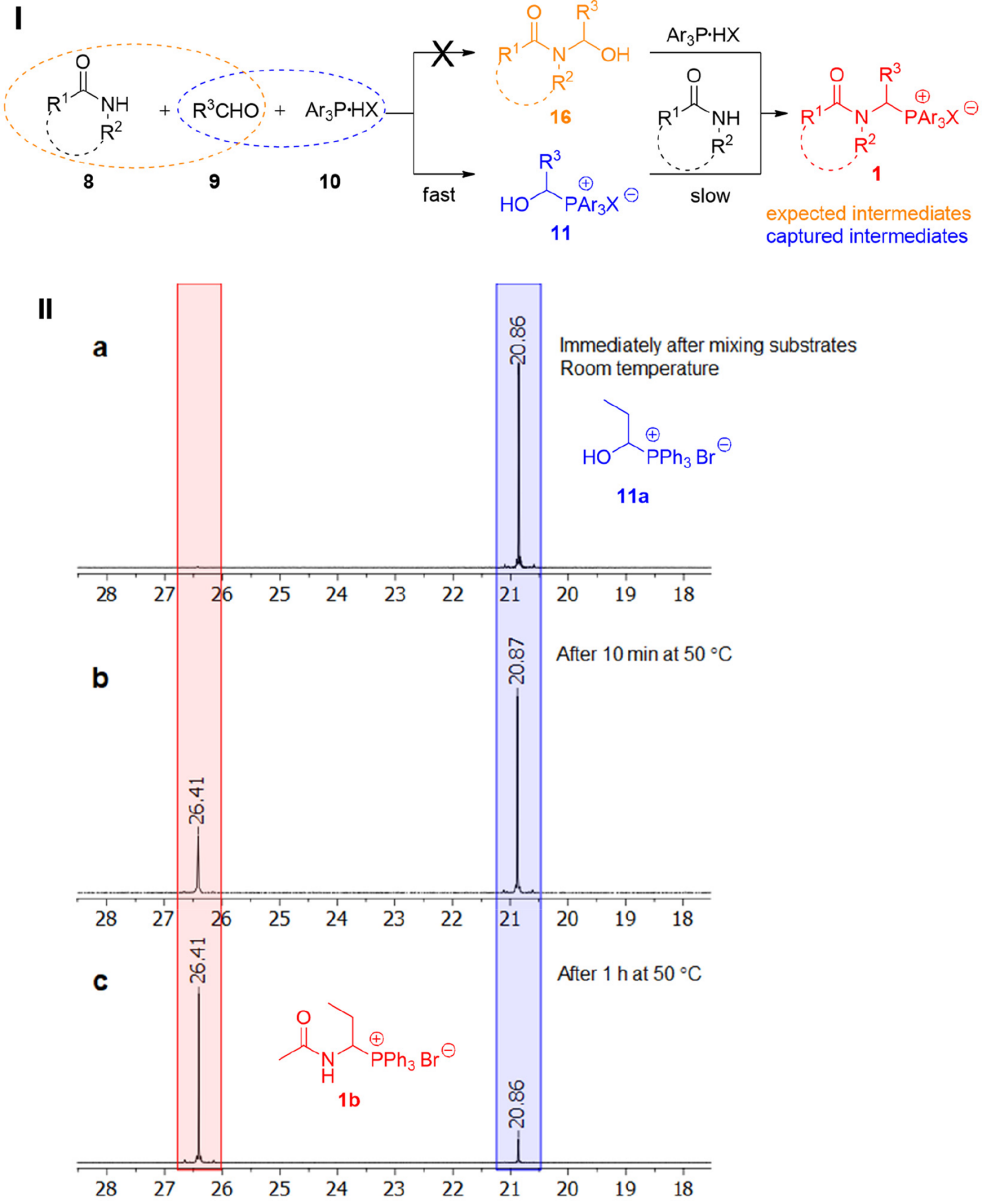
A plausible mechanism for the one-pot synthesis of *N*-protected 1-aminoalkylphosphonium salts (I) proposed based on the analysis of ^31^P NMR spectra (161.9 MHz/CDCl_3_; ppm) acquired at different stages of the reaction between propionaldehyde, acetamide, and triphenylphosphonium bromide (II).

We assumed that the one-pot reaction proceeds via the intermediate formation of *N*-hydroxyalkyl derivatives **16**, as shown in Figure 1.

However, monitoring the reaction of acetamide and propionaldehyde in the presence of triphenylphosphonium bromide by ^1^H and ^31^P NMR (Figure 1, II) revealed a different mechanism. Spectral analysis indicated that the new C–P bond was formed in the first stage (fast step) because the 1-hydroxypropylphosphonium salt **11a** (R^3^ = Et, Ar = Ph, X = Br, Figure 1) appeared early in the reaction mixture (immediately after mixing substrates, already at room temperature). Compound **11a** reacted with the acetamide in the second, slower step to generate the target 1-(*N*-acetylamino)propylphosphonium bromide **1b** (R^1^ = Me, R^2^ = H, R^3^ = Et; Ar = Ph, X = Br, Figure 1). It is worth noting that formation of *N*-(1-hydroxypropyl)acetamide **16a** (R^1^ = Me, R^2^ = H, R^3^ = Et; Figure 1) was not observed during the reaction. That was also confirmed by control reactions between acetamide and propionaldehyde without the addition of triphenylphosphonium salt. In this case, a small amount (about 10%) of the compound **16a** was detected only after heating of substrates at 50 °C for 1 h (the yield of **16a** can be improved by adding KHCO_3_ (10 mol %) to the reaction mixture); at room temperature compound **16a** was not formed at all.

To verify these observations and the associated mechanistic proposal, 1-hydroxypropyltriphenylphosphonium bromide **11a** was synthesized and isolated following the reaction between propionaldehyde and triphenylphosphonium bromide in acetonitrile at room temperature (90% yield). Then, the reaction between salt **11a** and acetamide at 50 °C in acetonitrile afforded the expected 1-(*N*-acetylamino)propyltriphenylphosphonium bromide **1b** with a 98% yield.

Reactions between aldehydes and either *N*-alkylamides, carbamates, lactams, imides, or urea in the presence of Ar_3_P·HX also proceed in accordance with the new proposed mechanism. This was confirmed by the fact that the corresponding 1-hydroxyalkylphosphonium salts were detected in the intermediate stages of all these reactions.

## Conclusions

Herein, we describe the development and optimization of an effective method for preparing *N*-protected 1-aminoalkylphosphonium salts, based on the one-pot reaction between aldehydes and either amides, carbamates, lactams, or imides in the presence of triarylphosphonium bromide or tetrafluoroborates (Ar_3_P·HX). The greatest advantages of this novel method include the versatility, simplicity of the reaction apparatus, high yields, and the ability to synthesize structurally diverse *N*-protected 1-aminoalkylphosphonium salts (i.e., compounds with a modified phosphonium moiety) even on a large scale (up to 20 g). The reaction proceeds under relatively mild conditions in a short time, and it can be carried out in various solvents such as acetonitrile, chloroform, THF, or in a solvent-free environment. Although some amides react at room temperature, it is preferable to perform the transformation at elevated temperature (between 50 and 100 °C). The use of paraformaldehyde as a substrate required a higher temperature (135 °C), and the reaction with imides required much more severe conditions (150–170 °C), extended reaction time (3–5 h), and was not very effective (29–70% yields).

This research revealed mechanistic insights regarding the examined transformations, including the unexpected formation of structurally interesting 1-hydroxyalkylphosphonium salts in the intermediate stage following the reaction of aldehydes with triarylphosphonium tetrafluoroborates or bromides. The 1-hydroxyalkylphosphonium salts are stable and easily separable. The reaction mechanism was confirmed by isolating the intermediate 1-hydroxyalkylphosphonium salts and reacting them further with an amide to obtain the expected 1-(*N*-acylamino)alkylphosphonium salts.

## Experimental Section

### General Methods

Melting points were determined in capillaries using a Stuart Scientific SMP3 melting point apparatus and were uncorrected. Infrared (IR) spectra were measured on a Fourier transform (FT)-IR spectrophotometer (using an attenuated total reflectance (ATR) method). ^1^H and ^13^C{^1^H} NMR (the proton decoupled ^13^C NMR) were recorded at operating frequencies of 400 and 100 MHz, respectively, using tetramethylsilane (TMS) as the resonance shift standard. ^31^P{^1^H} NMR spectra were recorded at an operating frequency of 161.9 MHz without the resonance shift standard, with respect to H_3_PO_4_ set as 0 ppm. All chemical shifts (δ) are reported in ppm and coupling constants (*J*) in Hz. High-resolution mass spectrometry (HR-MS) analyses were performed on a Waters Xevo G2 quadrupole time-of-flight (Q-TOF) mass spectrometer equipped with an electrospray ionization (ESI) source operating in the positive ion mode. The accurate mass and composition of molecular ion adducts were calculated using the MassLynx software incorporated within the instrument. Solvents (ACS grade) were stored over molecular sieves before use. All other commercially available reagents, including compounds **8**, **9** and triphenylphosphonium bromide (**10d**) were purchased and then used as received, without purification or modifications. Triarylphosphonium tetrafluoroborates (**10a**–**c**) were synthesized based on our previously described procedure.^13^

### Synthesis of Triarylphosphonium Tetrafluoroborates **10** from Triarylphosphines (Ar_3_P) and Tetrafluoroboric Acid Diethyl Ether Complex (HBF_4_·Et_2_O)

The reaction was carried out in a round-bottom flask fitted with a calcium chloride drying tube. Tetrafluoroboric acid diethyl ether complex (HBF_4_·Et_2_O; 1.36 cm^3^, 1.619 mg, 10 mmol) was added dropwise to a solution of triarylphosphine (10 mmol) in dichloromethane (10 cm^3^) which was cooled with an ice–water bath. After the addition of the acid, the reaction mixture was stirred for an additional 2 h at room temperature. Triarylphosphonium tetrafluoroborate was then precipitated with diethyl ether. The resulting precipitate was separated by vacuum filtration, washed on a Büchner funnel with CH_2_Cl_2_/Et_2_O (5 cm^3^, 1:3 [v/v]) and dried.

#### Triphenylphosphonium tetrafluoroborate (**10a**)

Colorless crystals (3.26 g, 93% yield), mp 172.0–174.0 °C. ^1^H NMR (400 MHz, CDCl_3_) δ 9.09 (d, *J* = 532.7 Hz, 1H), 7.85–7.71 (m, 9H), 7.70–7.55 (m, 6H) ppm; ^13^C{^1^H} NMR (100 MHz, CDCl_3_) δ 135.5 (d, *J* = 3.2 Hz), 134.0 (d, *J* = 11.6 Hz), 130.5 (d, *J* = 13.5 Hz), 115.7 (d, *J* = 88.5 Hz) ppm; ^31^P{^1^H} NMR (161.9 MHz, CDCl_3_) δ 3.8 ppm; IR (ATR) 3076, 1586, 1482, 1439, 1112, 1076, 1024, 998 cm^–1^. HRMS (ESI-TOF) *m*/*z* [M^+^] Calcd for C_18_H_16_P^+^ 263.0990, found 263.0995.

#### Tris(3-chlorophenyl)phosphonium tetrafluoroborate (**10b**)

Colorless crystals (4.04 g, 89% yield), mp 139.0–141.0 °C. ^1^H NMR (400 MHz, CDCl_3_) δ 7.80–7.65 (m, 3H), 7.65–7.47 (m, 9H) ppm; ^13^C{^1^H} NMR (100 MHz, CDCl_3_) δ 136.4 (d, *J* = 13.4 Hz), 134.1 (br s), 133.2 (d, *J* = 15.5 Hz), 132.3 (d, *J* = 14.1 Hz), 131.6 (br d, *J* = 11.6 Hz), 119.0 (d, *J* = 85.5 Hz) ppm; ^31^P{^1^H} NMR (161.9 MHz, CDCl_3_) δ −1.0 ppm; IR (ATR) 3561, 3076, 1575, 1558, 1462, 1392, 1108, 1088, 1068, 996 cm^–1^. HRMS (ESI-TOF) *m*/*z* [M^+^] Calcd for C_18_H_13_Cl_3_P^+^ 364.9820, found 364.9821.

#### Tris(4-methoxyphenyl)phosphonium tetrafluoroborate (**10c**)

Colorless crystals (4.18 g, 95% yield), mp 171.0–172.0 °C. ^1^H NMR (400 MHz, CDCl_3_) δ 8.92 (d, *J* = 527.2 Hz, 1H), 7.71–7.60 (m, 6H), 7.15–7.08 (m, 6H), 3.89 (s, 9H) ppm; ^13^C{^1^H} NMR (100 MHz, CDCl_3_) δ 165.0 (d, *J* = 2.9 Hz), 135.7 (d, *J* = 13.3 Hz), 116.2 (d, *J* = 14.8 Hz), 106.5 (d, *J* = 95.7 Hz), 55.8 ppm; ^31^P{^1^H} NMR (161.9 MHz, CDCl_3_) δ 1.8 ppm; IR (ATR) 3091, 2984, 1591, 1565, 1504, 1456, 1313, 1300, 1185, 1117, 1049, 1010, 893 cm^–1^. HRMS (ESI-TOF) *m*/*z* [M^+^] Calcd for C_21_H_22_O_3_P^+^ 353.1307, found 353.1307.

### One-Pot Reaction of Amides, Carbamates, Lactams, or Imides with Aldehydes in the Presence of Triarylphosphonium Salts

These one-pot reactions were carried out in a glass vial sealed with a screw-cap. The amide (carbamate, lactam, or imide; 1 mmol) and triarylphosphonium salt (bromide or tetrafluoroborate; 1 mmol) were added to a solution of aldehyde (1 mmol) in CH_3_CN (or CHCl_3_ or THF; 0.65 cm^3^). The obtained mixture was stirred vigorously and heated using an oil bath (time and temperature are given in Table 1 and 2). The *N*-protected 1-aminoalkylphosphonium salt was then precipitated with diethyl ether. Due to the relatively high concentration, the obtained *N*-protected 1-aminoalkylphosphonium salts often crystallized from the reaction mixture (especially from THF solutions). If necessary, the salt was recrystallized from CH_3_CN, CH_3_CN/Et_2_O, or CHCl_3_/Et_2_O.

### One-Pot Reaction of Amides, Carbamates, Lactams, or Imides with Aldehydes in the Presence of Triarylphosphonium Salts without Solvent

These solvent-free reactions were carried out in a glass vial sealed with a screw-cap. Aldehyde (1.0 or 1.2 mmol in the case of a volatile aldehydes such as acetaldehyde or propionaldehyde), amide (carbamate, lactam, or imide; 1 mmol), and triarylphosphonium salt (bromide or tetrafluoroborate; 1.0 mmol) were added to the vial. The obtained mixture was heated using an oil bath (time and temperature are given in Table 1 and 2). The obtained crude 1-(*N*-acetylamino)alkylphosphonium salts were recrystallized from CH_3_CN/Et_2_O or CHCl_3_/Et_2_O.

### One-Pot Reaction of Acetamide with Propionaldehyde and Triphenylphosphine in the Presence of HBF_4_

The one-pot reaction was carried out in a glass vial sealed with a screw-cap. Acetamide (14.8 mg, 0.25 mmol), triphenylphosphine (262.3 mg, 1 mmol), and HBF_4_·Et_2_O (tetrafluoroboric acid diethyl ether complex, 0.1360 cm^3^, 161.9 mg, 1 mmol) were added to a solution of propionaldehyde (0.0717 cm^3^, 58.1 mg, 1 mmol) in CH_3_CN (0.65 cm^3^). The obtained mixture was stirred vigorously and heated at 50 °C for 1 h using an oil bath. The product was precipitated with diethyl ether to afford pure 1-(*N*-acetylamino)propyltriphenylphosphonium tetrafluoroborate in 49% yield.

### One-Pot Reaction of Acetamide with Propionaldehyde in the Presence of Triphenylphosphonium Tetrafluoroborate on 20 g-Scale

The one-pot reaction was carried out in a 150 cm^3^ round-bottom flask equipped with a reflux condenser. Acetamide (3.54 g, 60 mmol) and triphenylphosphonium tetrafluoroborate (21.01 g, 60 mmol) were added to a solution of propionaldehyde (4.3 cm^3^, 3.48 g, 60 mmol) in CH_3_CN (39 cm^3^) which was cooled with an ice–water bath. Then, the obtained mixture was stirred vigorously and heated at 50 °C for 2 h using an oil bath. After that, the product was precipitated with diethyl ether (40 cm^3^), separated by vacuum filtration, washed on a Büchner funnel with CH_3_CN/Et_2_O (25 cm^3^, 1:3 [v/v]) and dried to afford pure 1-(*N*-acetylamino)propyltriphenylphosphonium tetrafluoroborate in 82% yield.

#### 1-(*N*-Acetylamino)propyltriphenylphosphonium tetrafluoroborate (**1a**)

Colorless crystals (390.8 mg, 87% yield), mp 185.0–186.0 °C. ^1^H NMR (400 MHz, CDCl_3_) δ 7.87–7.75 (m, 4H), 7.74–7.65 (m, 12H), 5.67 (dddd, *J* = 12.0, 9.2, 7.8, 2.7 Hz, 1H), 2.13–1.97 (m, 1H), 1.91 (d, *J* = 1.2 Hz, 3H), 1.87–1.72 (m, 1H), 1.12 (td, *J* = 7.1, 1.1 Hz, 3H) ppm; ^13^C{^1^H} NMR (100 MHz, CDCl_3_) δ 172.4 (d, *J* = 3.0 Hz), 135.2 (d, *J* = 3.1 Hz), 134.1 (d, *J* = 9.4 Hz), 130.4 (d, *J* = 12.3 Hz), 117.0 (d, *J* = 81.7 Hz), 49.5 (d, *J* = 53.4 Hz), 25.1 (d, *J* = 5.4 Hz), 22.2, 11.4 (d, *J* = 14.1 Hz) ppm; ^31^P{^1^H} NMR (161.9 MHz, CDCl_3_) δ 26.3 ppm; IR (ATR) 3335, 1683, 1523, 1440, 1286, 1108, 1061, 1019, 996 cm^–1^. HRMS (ESI-TOF) *m*/*z* [M^+^] Calcd for C_23_H_25_NOP^+^ 362.1674, found 362.1674.

#### 1-(*N*-Acetylamino)propyltriphenylphosphonium bromide (**1b**)

Colorless crystals (309.6 mg, 70% yield), mp 171.0–172.0 °C. ^1^H NMR (400 MHz, CDCl_3_) δ 9.91 (br d, *J* = 9.1 Hz, 1H), 7.91–7.76 (m, 9H), 7.74–7.63 (m, 6H), 5.74 (dddd, *J* = 11.9, 9.7, 7.3, 2.6 Hz, 1H), 2.50–2.34 (m, 1H), 2.02 (d, *J* = 1.3 Hz, 3H), 1.86–1.72 (m, 1H), 1.17 (td, *J* = 7.2, 0.9 Hz, 3H) ppm; ^13^C{^1^H} NMR (100 MHz, CDCl_3_) δ 172.6 (d, *J* = 3.1 Hz), 135.0 (d, *J* = 3.1 Hz), 134.4 (d, *J* = 9.4 Hz), 130.2 (d, *J* = 12.2 Hz), 117.2 (d, *J* = 81.3 Hz), 50.0 (d, *J* = 52.1 Hz), 25.0 (d, *J* = 5.6 Hz), 22.6, 11.8 (d, *J* = 14.2 Hz) ppm; ^31^P{^1^H} NMR (161.9 MHz, CDCl_3_) δ 26.7 ppm; IR (ATR) 3143, 2998, 1677, 1527, 1440, 1433, 1286, 1261, 1109 cm^–1^. HRMS (ESI-TOF) *m*/*z* [M^+^] Calcd for C_23_H_25_NOP^+^ 362.1674, found 362.1674.

#### 1-(*N*-Acetylamino)propyltris(3-chlorophenyl)phosphonium tetrafluoroborate (**1c**)

White resin (392.3 mg, 71% yield). ^1^H NMR (400 MHz, CDCl_3_) δ 7.88 (br d, *J* = 8.4 Hz, 1H), 7.86–7.79 (m, 3H), 7.78–7.69 (m, 6H), 7.55–7.48 (m, 3H), 5.65–5.52 (m, 1H), 2.18–2.00 (m, 1H), 1.92 (d, *J* = 1.1 Hz, 3H), 1.84–1.71 (m, 1H), 1.15 (td, *J* = 7.2, 0.9 Hz, 3H) ppm; ^13^C{^1^H} NMR (100 MHz, CDCl_3_) δ 172.5 (d, *J* = 2.8 Hz), 137.1 (d, *J* = 16.1 Hz), 136.0 (d, *J* = 2.9 Hz), 133.4 (d, *J* = 10.4 Hz), 132.4 (d, *J* = 9.1 Hz), 132.2 (d, *J* = 13.6 Hz), 118.6 (d, *J* = 80.9 Hz), 50.4 (d, *J* = 51.1 Hz), 25.0 (d, *J* = 5.6 Hz), 22.0, 11.4 (d, *J* = 14.5 Hz) ppm; ^31^P{^1^H} NMR (161.9 MHz, CDCl_3_) δ 25.8 ppm; IR (ATR) 3345, 1656, 1563, 1521, 1467, 1399, 1130, 1052, 993 cm^–1^. HRMS (ESI-TOF) *m*/*z* [M^+^] Calcd for C_23_H_22_Cl_3_NOP^+^ 464.0505, found 464.0504.

#### 1-(*N*-Acetylamino)ethyltriphenylphosphonium tetrafluoroborate (**1d**)^23^

Colorless crystals (396.0 mg, 91% yield), mp 150.0–151.0 °C. ^1^H NMR (400 MHz, CDCl_3_) δ 7.87 (br d, *J* = 8.8 Hz, 1H), 7.86–7.79 (m, 3H), 7.77–7.66 (m, 12H), 5.95–5.85 (m, 1H), 1.87 (br s, 3H), 1.67 (dd, *J* = 17.4, 7.3 Hz, 3H) ppm; ^13^C{^1^H} NMR (100 MHz, CDCl_3_) δ 171.6 (d, *J* = 2.9 Hz), 135.3 (d, *J* = 3.1 Hz), 134.2 (d, *J* = 9.3 Hz), 130.4 (d, *J* = 12.3 Hz), 116.8 (d, *J* = 81.7 Hz), 43.8 (d, *J* = 54.9 Hz), 22.4, 17.3 (d, *J* = 4.8 Hz) ppm; ^31^P{^1^H} NMR (161.9 MHz, CDCl_3_) δ 27.6 ppm; IR (ATR) 3254, 1669, 1534, 1442, 1372, 1109, 1057, 1046, 1023, 994 cm^–1^.

#### (*N*-Acetylamino)phenylmethyltriphenylphosphonium tetrafluoroborate (**1e**)^23^

Colorless crystals (263.6 mg, 53% yield), mp 217.0–218.0 °C. ^1^H NMR (400 MHz, CDCl_3_) δ 8.50 (br d, *J* = 9.6 Hz, 1H), 7.84–7.78 (m, 3H), 7.69–7.61 (m, 6H), 7.59–7.49 (m, 6H), 7.40–7.31 (m, 1H), 7.30–7.23 (m, 2H), 7.13–7.09 (m, 2H), 6.95 (dd ∼ t, *J* = 9.6, 9.6 Hz, 1H), 2.03 (d, *J* = 1.1 Hz, 3H) ppm; ^13^C{^1^H} NMR (100 MHz, CDCl_3_) δ 172.0 (d, *J* = 3.4 Hz), 135.3 (d, *J* = 3.1 Hz), 134.8 (d, *J* = 9.1 Hz), 130.3 (d, *J* = 1.9 Hz), 130.2 (d, *J* = 12.3 Hz), 130.2 (d, *J* = 3.1 Hz), 129.6 (d, *J* = 1.6 Hz), 129.5 (d, *J* = 1.6 Hz), 116.2 (d, *J* = 80.8 Hz), 54.6 (d, *J* = 52.0 Hz), 22.4 ppm; ^31^P{^1^H} NMR (161.9 MHz, CDCl_3_) δ 24.0 ppm; IR (ATR) 3341, 1690, 1521, 1494, 1437, 1101, 1054, 995 cm^–1^.

#### 1-(*N*-Acetylamino)-1-(4-metoxyphenyl)methyltris(4-metoxyphenyl)phosphonium tetrafluoroborate (**1f**)

White resin (512.4 mg, 83% yield). ^1^H NMR (400 MHz, CDCl_3_) δ 8.24 (br d, *J* = 9.8 Hz, 1H), 7.44–7.35 (m, 6H), 7.14–7.07 (m, 6H), 7.02–6.97 (m, 2H), 6.82–6.77 (m, 2H), 6.71 (dd ∼ t, *J* = 10.0, 10.0 Hz, 1H), 3.92 (s, 9H), 3.78 (s, 3H), 2.05 (d, *J* = 1.0 Hz, 3H) ppm; ^13^C{^1^H} NMR (100 MHz, CDCl_3_) δ 171.7 (d, *J* = 3.6 Hz), 164.8 (d, *J* = 3.0 Hz), 160.7 (d, *J* = 2.5 Hz), 136.6 (d, *J* = 10.7 Hz), 131.0 (d, *J* = 5.4 Hz), 122.7 (d, *J* = 2.1 Hz), 115.8 (d, *J* = 13.3 Hz), 114.71 (d, *J* = 1.8 Hz), 106.8 (d, *J* = 89.6 Hz), 55.8, 55.4, 53.5 (d, *J* = 57.4 Hz), 22.5 ppm; ^31^P{^1^H} NMR (161.9 MHz, CDCl_3_) δ 21.7 ppm; IR (ATR) 3343, 2944, 1682, 1592, 1567, 1502, 1461, 1297, 1264, 1183, 1108, 1056, 1016 cm^–1^. HRMS (ESI-TOF) *m*/*z* Calcd for C_31_H_33_NO_5_P^+^ [M^+^] 530.2096, found 530.2094.

#### (*N*-Acetylamino)methyltriphenylphosphonium tetrafluoroborate (**1g**)^39^

Colorless crystals (383.3 mg, 91% yield), mp 191.0–193.0 °C. ^1^H NMR (400 MHz, CDCl_3_) δ 7.86–7.79 (m, 3H), 7.78 (dd ∼ t, *J* = 5.9, 5.9 Hz, 1H), 7.75–7.65 (m, 12H), 5.06 (dd, *J* = 6.3, 3.3 Hz, 2H), 1.81 (d, *J* = 1.3 Hz, 3H) ppm; ^13^C{^1^H} NMR (100 MHz, CDCl_3_) δ 171.9 (d, *J* = 1.3 Hz), 135.3 (d, *J* = 3.1 Hz), 134.1 (d, *J* = 9.7 Hz), 130.3 (d, *J* = 12.6 Hz), 117.1 (d, *J* = 84.1 Hz), 37.2 (d, *J* = 58.0 Hz), 22.0 ppm; ^31^P{^1^H} NMR (161.9 MHz, CDCl_3_) δ 20.7 ppm; IR (ATR) 3382, 1684, 1519, 1438, 1260, 1112, 1086, 1055, 1016, 996 cm^–1^.

#### (*N*-Acetylamino)methyltriphenylphosphonium bromide (**1h**)^39^

Colorless crystals (410.2 mg, 99% yield), mp 250–251 °C. ^1^H NMR (400 MHz, CDCl_3_) δ 9.68 (dd ∼ t, *J* = 6.1, 6.1 Hz, 1H), 7.87–7.76 (m, 9H), 7.74–7.65 (m, 6H), 5.13 (dd, *J* = 6.3, 2.9 Hz, 2H), 1.90 (d, *J* = 1.4 Hz, 3H) ppm; ^13^C{^1^H} NMR (100 MHz, CDCl_3_) δ 172.1 (d, *J* = 1.3 Hz), 135.1 (d, *J* = 3.1 Hz), 134.3 (d, *J* = 9.8 Hz), 130.2 (d, *J* = 12.6 Hz), 117.4 (d, *J* = 83.9 Hz), 37.5 (d, *J* = 56.8 Hz), 22.4 ppm; ^31^P{^1^H} NMR (161.9 MHz, CDCl_3_) δ 20.7 ppm; IR (ATR) 3163, 3006, 1675, 1525, 1436, 1362, 1267, 1253, 1110 cm^–1^.

#### (*N*-Acetylamino)methyltris(3-chlorophenyl)phosphonium tetrafluoroborate (**1i**)

Colorless crystals (487.8 mg, 93% yield), mp 179.0–181.0 °C. ^1^H NMR (400 MHz, CDCl_3_) δ 7.99 (dd ∼ t, *J* = 6.0, 6.0 Hz, 1H), 7.85–7.74 (m, 6H), 7.71 (td, *J* = 7.9, 4.2 Hz, 3H), 7.55 (dt, *J* = 13.0, 1.7 Hz, 3H), 5.09 (dd, *J* = 6.1, 2.5 Hz, 2H), 1.84 (d, *J* = 1.4 Hz, 3H) ppm; ^13^C{^1^H} NMR (100 MHz, CDCl_3_) δ 172.2 (d, *J* = 1.2 Hz), 137.0 (d, *J* = 16.7 Hz), 136.0 (d, *J* = 3.0 Hz), 133.4 (d, *J* = 11.0 Hz), 132.4 (d, *J* = 9.5 Hz), 132.0 (d, *J* = 13.9 Hz), 118.6 (d, *J* = 83.3 Hz), 38.0 (d, *J* = 55.5 Hz), 21.9 ppm; ^31^P{^1^H} NMR (161.9 MHz, CDCl_3_) δ 20.7 ppm; IR (ATR) 3374, 1683, 1518, 1464, 1403, 1129, 1071, 1046, 1029, 994 cm^–1^. HRMS (ESI-TOF) *m*/*z* Calcd for C_21_H_18_Cl_3_NOP^+^ [M^+^] 436.0192, found 436.0193.

#### 1-(*N*-Benzoylamino)propyltriphenylphosphonium tetrafluoroborate (**1j**)

Colorless crystals (281.2 mg, 55% yield), mp 198.0–199.0 °C. ^1^H NMR (400 MHz, CDCl_3_) δ 8.42 (dd, *J* = 8.3, 2.0 Hz, 1H), 7.82–7.75 (m, 8H), 7.74–7.69 (m, 3H), 7.68–7.61 (m, 6H), 7.48–7.42 (m, 1H), 7.39–7.33 (m, 2H), 5.80 (dtd, *J* = 11.5, 8.3, 3.1 Hz, 1H), 2.55–2.38 (m, 1H), 1.88–1.74 (m, 1H), 1.18 (td, *J* = 7.2, 1.1 Hz, 3H) ppm; ^13^C{^1^H} NMR (100 MHz, CDCl_3_) δ 168.5 (d, *J* = 2.3 Hz), 134.9 (d, *J* = 3.0 Hz), 134.4 (d, *J* = 9.4 Hz), 132.3, 131.7, 130.1 (d, *J* = 12.3 Hz), 128.6, 127.4, 117.9 (d, *J* = 81.7 Hz), 51.4 (d, *J* = 51.7 Hz), 24.9 (d, *J* = 5.2 Hz), 11.7 (d, *J* = 13.9 Hz) ppm; ^31^P{^1^H} NMR (161.9 MHz, CDCl_3_) δ 27.0 ppm; IR (ATR) 3357, 2904, 1670, 1508, 1484, 1437, 1112, 1070, 993 cm^–1^. HRMS (ESI-TOF) *m*/*z* Calcd for C_28_H_27_NOP^+^ [M^+^] 424.1830, found 424.1831.

#### 1-(*N*-Benzoylamino)ethyltris(3-chlorophenyl)phosphonium tetrafluoroborate (**1k**)

White resin (414.4 mg, 69% yield). ^1^H NMR (400 MHz, CDCl_3_) δ 8.72 (dd ∼ t, *J* = 6.2, 6.2 Hz, 1H), 7.86–7.79 (m, 3H), 7.77–7.72 (m, 2H), 7.71–7.57 (m, 7H), 7.55–7.42 (m, 3H), 7.41–7.33 (m, 2H), 6.01–5.86 (m, 1H), 1.86 (dd, *J* = 18.4, 8.7 Hz, 3H) ppm; ^13^C{^1^H} NMR (100 MHz, CDCl_3_) δ 168.1 (d, *J* = 2.1 Hz), 136.8 (d, *J* = 16.3 Hz), 135.6 (d, *J* = 2.9 Hz), 133.7 (d, *J* = 10.5 Hz), 132.7, 132.6 (d, *J* = 9.1 Hz), 131.8 (d, *J* = 13.7 Hz), 130.9, 128.7, 127.3, 119.7 (d, *J* = 81.3 Hz), 46.7 (d, *J* = 51.2 Hz), 17.5 (d, *J* = 4.6 Hz); ^31^P{^1^H} NMR (161.9 MHz, CDCl_3_) δ 27.7 ppm; IR (ATR) 3352, 3069, 1656, 1521, 1468, 1400, 1130, 1054, 993 cm^–1^. HRMS (ESI-TOF) *m*/*z* Calcd for C_27_H_22_Cl_3_NOP^+^ [M^+^] 512.0505, found 512.0502.

#### (*N*-Benzoylamino)methyltriphenylphosphonium tetrafluoroborate (**1l**)^39^

Colorless crystals (405.9 mg, 84% yield), mp 194.0–196.0 °C. ^1^H NMR (400 MHz, CDCl_3_) δ 8.42 (dd ∼ t, *J* = 6.1, 6.1 Hz, 1H), 7.84–7.74 (m, 9H), 7.71–7.60 (m, 8H), 7.48–7.42 (m, 1H), 7.38–7.31 (m, 2H), 5.32 (dd, *J* = 6.1, 3.1 Hz, 2H) ppm; ^13^C{^1^H} NMR (100 MHz, CDCl_3_) δ 168.4 (d, *J* = 1.0 Hz), 135.1 (d, *J* = 3.1 Hz), 134.3 (d, *J* = 9.7 Hz), 132.3, 131.7, 130.2 (d, *J* = 12.6 Hz), 128.6, 127.3, 117.4 (d, *J* = 83.9 Hz), 38.1 (d, *J* = 57.0 Hz) ppm; ^31^P{^1^H} NMR (161.9 MHz, CDCl_3_) δ 21.0 ppm; IR (ATR) 3343, 1655, 1533, 1313, 1113, 1057, 996 cm^–1^.

#### (*N*-Benzoylamino)methyltriphenylphosphonium bromide (**1m**)^39^

Colorless crystals (471.5 mg, 99% yield), mp 234.0–235.0 °C. ^1^H NMR (400 MHz, CDCl_3_) δ 10.04 (dd ∼ t, *J* = 6.0, 6.0 Hz, 1H), 7.95–7.83 (m, 8H), 7.79–7.72 (m, 3H), 7.68–7.60 (m, 6H), 7.47–7.41 (m, 1H), 7.38–7.33 (m, 2H), 5.41 (dd, *J* = 6.1, 2.6 Hz, 2H) ppm; ^13^C{^1^H} NMR (100 MHz, CDCl_3_) δ 168.4 (d, *J* = 0.7 Hz), 135.0 (d, *J* = 3.1 Hz), 134.5 (d, *J* = 9.7 Hz), 132.1, 131.7, 130.1 (d, *J* = 12.6 Hz), 128.4, 127.8, 117.8 (d, *J* = 83.8 Hz), 38.4 (d, *J* = 55.1 Hz) ppm; ^31^P{^1^H} NMR (161.9 MHz, CDCl_3_) δ 21.0 ppm; IR (ATR) 3159, 1644, 1527, 1486, 1436, 1315, 1272, 1111 cm^–1^.

#### (*N*-Benzoylamino)methyltris(3-chlorophenyl)phosphonium tetrafluoroborate (**1n**)

Colorless crystals (545.5 mg, 93% yield), mp 173.0–175.0 °C. ^1^H NMR (400 MHz, CDCl_3_) δ 8.58 (dd ∼ t, *J* = 5.7, 5.7 Hz, 1H), 7.89–7.80 (m, 3H), 7.78–7.73 (m, 3H), 7.72–7.57 (m, 8H), 7.51–7.45 (m, 1H), 7.41–7.34 (m, 2H), 5.32 (dd, *J* = 6.0, 2.4 Hz, 2H) ppm; ^13^C{^1^H} NMR (100 MHz, CDCl_3_) δ 168.6 (d, *J* = 0.7 Hz), 136.9 (d, *J* = 16.4 Hz), 135.9 (d, *J* = 3.0 Hz), 133.6 (d, *J* = 11.0 Hz), 132.6, 132.5 (d, *J* = 9.5 Hz), 131.9 (d, *J* = 14.0 Hz), 131.1, 128.7, 127.4, 118.9 (d, *J* = 83.2 Hz), 38.8 (d, *J* = 54.0 Hz) ppm; ^31^P{^1^H} NMR (161.9 MHz, CDCl_3_) δ 16.3 ppm; IR (ATR) 3341, 1668, 1520, 1472, 1400, 1280, 1132, 1077, 1051, 995 cm^–1^. HRMS (ESI-TOF) *m*/*z* Calcd for C_26_H_20_Cl_3_NOP^+^ [M^+^] 498.0348, found 498.0348.

#### 1-(*N*-Pivaloylamino)propyltriphenylphosphonium bromide (**1o**)

Colorless crystals (383.2 mg, 78% yield), mp 161.0–163.0 °C. ^1^H NMR (400 MHz, CDCl_3_) δ 9.29 (dd, *J* = 7.5, 3.4 Hz, 1H), 7.91–7.82 (m, 6H), 7.78–7.72 (m, 3H), 7.68–7.60 (m, 6H), 5.97–5.88 (m, 1H), 2.66–2.49 (m, 1H), 1.73–1.59 (m, 1H), 1.15 (td, *J* = 7.3, 0.8 Hz, 3H), 0.99 (s, 9H) ppm; ^13^C{^1^H} NMR (100 MHz, CDCl_3_) δ 180.1 (d, *J* = 2.4 Hz), 134.8 (d, *J* = 9.4 Hz), 134.4 (d, *J* = 3.1 Hz), 129.9 (d, *J* = 12.3 Hz), 118.9 (d, *J* = 81.4 Hz), 50.4 (d, *J* = 49.5 Hz), 38.7, 27.3, 24.6 (d, *J* = 5.7 Hz), 11.8 (d, *J* = 13.9 Hz) ppm; ^31^P{^1^H} NMR (161.9 MHz, CDCl_3_) δ 28.2 ppm; IR (ATR) 3201, 2972, 1659, 1509, 1482, 1438, 1183, 1109, 997 cm^–1^. HRMS (ESI-TOF) *m*/*z* Calcd for C_26_H_31_NOP^+^ [M^+^] 404.2143, found 404.2144.

#### 1-(*N*-Pivaloylamino)-1-(2-thienyl)methyltriphenylphosphonium tetrafluoroborate (**1p**)

Colorless crystals (441.8 mg, 81% yield), mp 195.0–197.0 °C. ^1^H NMR (400 MHz, CDCl_3_) δ 8.39 (dd ∼ t, *J* = 7.4, 7.4 Hz, 1H), 7.81–7.68 (m, 9H), 7.66–7.56 (m, 6H), 7.21–7.13 (m, 2H), 7.12–7.09 (m, 1H), 6.91–6.84 (m, 1H), 0.97 (s, 9H) ppm; ^13^C{^1^H} NMR (100 MHz, CDCl_3_) δ 179.8 (d, *J* = 2.6 Hz), 134.7 (d, *J* = 9.2 Hz), 134.5 (d, *J* = 2.1 Hz), 133.7 (d, *J* = 2.9 Hz), 130.3 (d, *J* = 7.2 Hz), 129.8 (d, *J* = 12.5 Hz), 127.9 (d, *J* = 3.6 Hz), 127.2 (d, *J* = 2.3 Hz), 119.2 (d, *J* = 82.9 Hz), 49.9 (d, *J* = 59.4 Hz), 38.4, 26.7; ^31^P{^1^H} NMR (161.9 MHz, CDCl_3_) δ 26.2 ppm; IR (ATR) 3372, 2953, 1655, 1507, 1485, 1439, 1190, 1092, 1012, 995 cm^–1^. HRMS (ESI-TOF) *m*/*z* Calcd for C_28_H_29_NOPS^+^ [M^+^] 458.1707, found 458.1707.

#### *N*-(*N*-Methylacetylamino)methyltriphenylphosphonium tetrafluoroborate (**1r**)

Colorless crystals (330.8 mg, 76% yield), mp 189.0–191.0 °C. ^1^H NMR (400 MHz, CDCl_3_) δ 7.85–7.78 (m, 3H), 7.76–7.66 (m, 12H), 5.31 (d, *J* = 3.4 Hz, 1H), 3.09 (d, *J* = 0.4 Hz, 3H), 1.84 (d, *J* = 1.2 Hz, 3H) ppm; ^13^C{^1^H} NMR (100 MHz, CDCl_3_) δ 172.1 (d, *J* = 1.2 Hz), 135.2 (d, *J* = 3.1 Hz), 134.0 (d, *J* = 9.9 Hz), 130.3 (d, *J* = 12.6 Hz), 117.6 (d, *J* = 83.9 Hz), 45.3 (d, *J* = 56.2 Hz), 38.6, 20.7 ppm; ^31^P{^1^H} NMR (161.9 MHz, CDCl_3_) δ 19.1 ppm; IR (ATR) 1634, 1438, 1401, 1111, 1046, 1035, 1024, 997, 983 cm^–1^. HRMS (ESI-TOF) *m*/*z* Calcd for C_22_H_23_NOP [M^+^] 348.1517, found 348.1520.

#### 1-(*N*-Benzyloxycarbonylamino)propyltriphenylphosphonium tetrafluoroborate (**1t**)

Colorless crystals (427.6 mg, 79% yield), mp 161.0–162.0 °C. ^1^H NMR (400 MHz, CDCl_3_) δ 7.80–7.71 (m, 3H), 7.71–7.58 (m, 12H), 7.33–7.26 (m, 3H), 7.24–7.18 (m, 2H), 6.88 (br d, *J* = 9.1 Hz, 1H), 5.44–5.33 (m, 1H), 4.98 and 4.89 (ABq, *J* = 12.4 Hz, 2H), 2.29–2.12 (m, 1H), 1.85–1.71 (m, 1H), 1.15 (td, *J* = 7.2, 0.9 Hz, 3H) ppm; ^13^C{^1^H} NMR (100 MHz, CDCl_3_) δ 156.7 (d, *J* = 2.8 Hz), 136.0, 135.1 (d, *J* = 3.0 Hz), 134.1 (d, *J* = 9.3 Hz), 130.3 (d, *J* = 12.3 Hz), 128.4, 127.9, 127.8, 116.9 (d, *J* = 81.2 Hz), 67.2, 52.5 (d, *J* = 52.9 Hz), 24.9 (d, *J* = 6.4 Hz), 11.3 (d, *J* = 13.9 Hz) ppm; ^31^P{^1^H} NMR (161.9 MHz, CDCl_3_) δ 25.6 ppm; IR (ATR) 3332, 1710, 1519, 1439, 1227, 1111, 1063, 1035, 1009, 995 cm^–1^. HRMS (ESI-TOF) *m*/*z* Calcd for C_29_H_29_NO_2_P^+^ [M^+^] 454.1936, found 454.1938.

#### 1-(*N*-Benzyloxycarbonylamino)-2-methylpropyltriphenylphosphonium tetrafluoroborate (**1u**)^23^

White resin (449.9 mg, 81% yield). ^1^H NMR (400 MHz, CDCl_3_) δ 7.79–7.68 (m, 9H), 7.65–7.57 (m, 6H), 7.31–7.26 (m, 3H), 7.19–7.14 (m, 2H), 7.03 (br d, *J* = 8.6 Hz, 1H), 5.33 (ddd ∼ q, *J* = 8.9, 8.9, 8.9 Hz, 1H), 4.87 and 4.78 (ABq, *J* = 12.4 Hz, 2H), 2.74–2.60 (m, 1H), 1.01 (d, *J* = 6.5 Hz, 3H), 0.88 (d, *J* = 6.7 Hz, 3H) ppm; ^13^C{^1^H} NMR (100 MHz, CDCl_3_) δ 156.3, 135.7, 134.8 (d, *J* = 3.0 Hz), 134.5 (d, *J* = 9.3 Hz), 130.0 (d, *J* = 12.3 Hz), 128.4, 128.0, 127.8, 118.1 (d, *J* = 80.2 Hz), 67.2, 55.9 (d, *J* = 47.2 Hz), 29.6 (d, *J* = 6.4 Hz), 21.2 (d, *J* = 4.2 Hz), 19.4 (d, *J* = 8.1 Hz) ppm; ^31^P{^1^H} NMR (161.9 MHz, CDCl_3_) δ 27.3 ppm; IR (ATR) 3337, 2975, 1716, 1518, 1438, 1233, 1053, 996 cm^–1^.

#### (*N*-Benzyloxycarbonylamino)phenylmethyltriphenylphosphonium tetrafluoroborate (**1v**)^23^

Colorless crystals (465.6 mg, 79% yield), mp 177.0–178.0 °C. ^1^H NMR (400 MHz, CDCl_3_) δ 7.80–7.71 (m, 3H), 7.65–7.55 (m, 7H), 7.55–7.48 (m, 6H), 7.39–7.32 (m, 1H), 7.31–7.26 (m, 5H), 7.25–7.22 (m, 2H), 7.14–7.09 (m, 2H), 6.65 (dd ∼ t, *J* = 9.7, 9.7 Hz, 1H), 5.00 and 4.94 (ABq, *J* = 12.4 Hz, 2H) ppm; ^13^C{^1^H} NMR (100 MHz, CDCl_3_) δ 156.5, 135.8, 135.3 (d, *J* = 3.1 Hz), 134.8 (d, *J* = 9.1 Hz), 130.4, 130.2 (d, *J* = 12.2 Hz), 130.2, 129.5 (d, *J* = 2.2 Hz), 129.3 (d, *J* = 5.3 Hz), 128.4, 127.9, 127.9, 116.0 (d, *J* = 80.6 Hz), 67.6, 56.9 (d, *J* = 54.5 Hz) ppm; ^31^P{^1^H} NMR (161.9 MHz, CDCl_3_) δ 23.2 ppm; IR (ATR) 3314, 1705, 1526, 1441, 1288, 1247, 1108, 1047, 1011, 996 cm^–1^.

#### 1-(*N*-Benzyloxycarbonylamino)-1-(4-methoxyphenyl)methyltriphenylphosphonium tetrafluoroborate (**1w**)

Colorless crystals (514.1 mg, 83% yield), mp 147.0–148.0 °C. ^1^H NMR (400 MHz, CDCl_3_) δ 7.80–7.73 (m, 3H), 7.64–7.56 (m, 6H), 7.55–7.47 (m, 7H), 7.30–7.26 (m, 5H), 7.05–6.99 (m, 2H), 6.80–6.74 (m, 2H), 6.58 (dd ∼ t, *J* = 9.4, 9.4 Hz, 1H), 5.00 and 4.93 (ABq, *J* = 12.4 Hz, 2H), 3.76 (s, 3H) ppm; ^13^C{^1^H} NMR (100 MHz, CDCl_3_) δ 160.9 (d, *J* = 2.1 Hz), 135.8, 135.3 (d, *J* = 2.8 Hz), 134.7 (d, *J* = 9.0 Hz), 130.8 (d, *J* = 5.1 Hz), 130.2 (d, *J* = 12.2 Hz), 128.4, 127.9, 127.8, 122.0, 116.1 (d, *J* = 80.5 Hz), 114.8, 67.6, 56.3 (d, *J* = 52.0 Hz), 55.4 ppm; ^31^P{^1^H} NMR (161.9 MHz, CDCl_3_) δ 22.6 ppm; IR (ATR) 3334, 2935, 1713, 1608, 1509, 1440, 1290, 1263, 1237, 1066, 1040, 996 cm^–1^. HRMS (ESI-TOF) *m*/*z* Calcd for C_34_H_31_NO_3_P^+^ [M^+^] 532.2042, found 532.2043.

#### 1-(*N*-Benzyloxycarbonylamino)-1-(2-nitrophenyl)methyltriphenylphosphonium tetrafluoroborate (**1x**)

Beige crystals (431.4 mg, 68% yield), mp 147.0–148.0 °C. ^1^H NMR (400 MHz, CDCl_3_) δ 7.96–7.87 (m, 1H), 7.86–7.72 (m, 6H), 7.68–7.50 (m, 13H), 7.49–7.41 (m, 1H), 7.31–7.21 (m, 5H), 5.00 and 4.94 (ABq, *J* = 12.5 Hz, 2H)ppm; ^13^C{^1^H} NMR (100 MHz, CDCl_3_) δ 156.0, 147.3 (d, *J* = 6.3 Hz), 136.2 (d, *J* = 2.3 Hz), 135.5 (d, *J* = 3.1 Hz), 134.7 (d, *J* = 9.2 Hz), 132.2 (d, *J* = 4.3 Hz), 131.1 (d, *J* = 2.4 Hz), 130.3 (d, *J* = 12.4 Hz), 128.4, 128.0, 127.8, 126.8 (d, *J* = 4.7 Hz), 125.2, 115.5 (d, *J* = 81.1 Hz), 67.8, 51.4 (d, *J* = 54.8 Hz) ppm; ^31^P{^1^H} NMR (161.9 MHz, CDCl_3_) δ 26.7 ppm; IR (ATR) 3343, 2995, 1739, 1530, 1512, 1438, 1338, 1240, 1105, 1051, 1013, 994 cm^–1^. HRMS (ESI-TOF) *m*/*z* Calcd for C_33_H_28_N_2_O_4_P^+^ [M^+^] 547.1787, found 547.1784.

#### 1-(*N*-Benzyloxycarbonylamino)-1-(1-naphthyl)methyltriphenylphosphonium tetrafluoroborate (**1y**)

White crystals (581.9 mg, 91% yield), mp 179.0–180.0 °C. ^1^H NMR (400 MHz, CDCl_3_) δ 7.87–7.81 (m, 2H), 7.75–7.69 (m, 3H), 7.60 (br d, *J* = 8.7 Hz, 1H), 7.56–7.49 (m, 8H), 7.48–7.40 (m, 8H), 7.40–7.33 (m, 2H), 7.30–7.21 (m, 5H), 5.02 and 4.93 (ABq, *J* = 12.5 Hz, 2H) ppm; ^13^C{^1^H} NMR (100 MHz, CDCl_3_) δ 156.6, 135.7, 135.4 (d, *J* = 3.1 Hz), 134.6 (d, *J* = 9.2 Hz), 133.4 (d, *J* = 1.1 Hz), 130.8, 130.8, 130.7, 130.2 (d, *J* = 12.3 Hz), 129.3, 128.4, 127.9, 127.7, 127.4, 126.7 (d, *J* = 3.0 Hz), 126.3, 126.1 (d, *J* = 2.9 Hz), 121.4, 115.7 (d, *J* = 80.7 Hz), 67.8, 51.3 (d, *J* = 55.6 Hz) ppm; ^31^P{^1^H} NMR (161.9 MHz, CDCl_3_) δ 24.4 ppm; IR (ATR) 3288, 1702, 1511, 1436, 1270, 1243, 1106, 1053, 1009 cm^–1^. HRMS (ESI-TOF) *m*/*z* Calcd for C_37_H_31_NO_2_P^+^ [M^+^] 552.2092, found 552.2095.

#### 1-(*N*-Benzyloxycarbonylamino)propyltris(4-methoxyphenyl)phosphonium tetrafluoroborate (**1z**)

Colorless crystals (435.7 mg, 69% yield), mp 114.0–116.0 °C. ^1^H NMR (400 MHz, CDCl_3_) δ 7.58–7.49 (m, 6H), 7.33–7.26 (m, 3H), 7.26–7.22 (m, 2H), 7.12–7.06 (m, 6H), 6.62 (br d, *J* = 9.5 Hz, 1H), 5.20 (dtd, *J* = 12.0, 9.5, 2.6 Hz, 1H), 5.04 and 4.96 (ABq, *J* = 12.5 Hz, 2H), 3.89 (s, 9H), 2.16–1.98 (m, 1H), 1.84–1.72 (m, 1H), 1.11 (td, *J* = 7.4, 1.0 Hz, 3H) ppm; ^13^C{^1^H} NMR (100 MHz, CDCl_3_) δ 164.7 (d, *J* = 2.9 Hz), 156.7 (d, *J* = 3.6 Hz), 136.1, 135.9 (d, *J* = 10.9 Hz), 128.3, 127.9, 127.7, 116.1 (d, *J* = 13.4 Hz), 107.4 (d, *J* = 90.0 Hz), 67.2, 55.8, 52.4 (d, *J* = 57.7 Hz), 24.6 (d, *J* = 6.1 Hz), 11.2 (d, *J* = 13.7 Hz) ppm; ^31^P{^1^H} NMR (161.9 MHz, CDCl_3_) δ 23.6 ppm; IR (ATR) 3298, 1729, 1592, 1501, 1288, 1265, 1237, 1186, 1112, 1057, 1036, 1017 cm^–1^. HRMS (ESI-TOF) *m*/*z* Calcd for C_32_H_35_NO_5_P^+^ [M^+^] 544.2253, found 544.2253.

#### 1-(*N*-*tert*-Butoxycarbonylamino)-2-methylpropyltriphenylphosphonium tetrafluoroborate (**1aa**)^23^

Colorless crystals (406.6 mg, 78% yield), mp 104.0–105.0 °C. ^1^H NMR (400 MHz, CDCl_3_) δ 7.86–7.71 (m, 9H), 7.70–7.61 (m, 6H), 6.70 (dd, *J* = 8.8, 2.6 Hz, 1H), 5.31 (ddd ∼ q, *J* = 8.9, 8.9, 8.9 Hz, 1H), 2.70–2.57 (m, 1H), 1.17 (s, 9H), 1.01 (d, *J* = 6.5 Hz, 3H), 0.87 (d, *J* = 6.7 Hz, 3H) ppm; ^13^C{^1^H} NMR (100 MHz, CDCl_3_) δ 155.5, 134.6, 134.6 (d, *J* = 9.2 Hz), 130.0 (d, *J* = 12.2 Hz), 118.6 (d, *J* = 80.1 Hz), 80.9, 55.1 (d, *J* = 47.3 Hz), 29.5 (d, *J* = 7.1 Hz), 27.8, 21.2 (d, *J* = 4.4 Hz), 19.1 (d, *J* = 8.0 Hz) ppm; ^31^P{^1^H} NMR (161.9 MHz, CDCl_3_) δ 27.8 ppm; IR (ATR) 3352, 2965, 1715, 1512, 1440, 1248, 1156, 1105, 1070, 996, 975 cm^–1^.

#### 1-(*N*-*tert*-Butoxycarbonylamino)-1-(2-furyl)methyltriphenylphosphonium tetrafluoroborate (**1ab**)

Colorless crystals (398.1 mg, 73% yield), mp 143.0–144.0 °C. ^1^H NMR (400 MHz, CDCl_3_) δ 7.84–7.73 (m, 3H), 7.70–7.57 (m, 12H), 7.21 (br s, 1H), 7.08 (br s, 1H), 6.85–6.75 (m, 1H), 6.55 (br s, 1H), 6.32 (dd, *J* = 3.0, 1.7 Hz, 1H), 1.27 (s, 9H) ppm; ^13^C{^1^H} NMR (100 MHz, CDCl_3_) δ 155.0, 144.2, 143.4 (d, *J* = 1.7 Hz), 135.1 (d, *J* = 2.0 Hz), 134.6 (d, *J* = 9.4 Hz), 130.1 (d, *J* = 12.4 Hz), 117.0 (d, *J* = 81.1 Hz), 112.7 (d, *J* = 4.6 Hz), 111.9, 81.8, 50.8 (d, *J* = 54.4 Hz), 27.9; ^31^P{^1^H} NMR (161.9 MHz, CDCl_3_) δ 24.8 ppm; IR (ATR) 3348, 2977, 1686, 1508, 1439, 1318, 1252, 1168, 1146, 1052, 1032, 1009, 983 cm^–1^. HRMS (ESI-TOF) *m*/*z* Calcd for C_28_H_29_NO_3_P^+^ [M^+^] 458.1885, found 458.1884.

#### 1-(2-Oxopyrrolidin-1-yl)propyltriphenylphosphonium tetrafluoroborate (**1ac**)

Colorless crystals (465.8 mg, 98% yield), mp 186.0–188.0 °C. ^1^H NMR (400 MHz, CDCl_3_) δ 7.88–7.80 (m, 3H), 7.78–7.68 (m, 12H), 5.72 (ddd, *J* = 12.5, 10.5, 3.0 Hz, 1H), 3.56–3.47 (m, 1H), 3.33–3.21 (m, 1H), 2.46–2.28 (m, 1H), 2.28–2.07 (m, 2H), 1.99–1.78 (m, 3H), 1.08 (td, *J* = 7.3, 1.1 Hz, 3H) ppm; ^13^C{^1^H} NMR (100 MHz, CDCl_3_) δ 176.8 (d, *J* = 2.3 Hz), 135.5 (d, *J* = 3.1 Hz), 134.2 (d, *J* = 9.7 Hz), 130.6 (d, *J* = 12.4 Hz), 116.9 (d, *J* = 81.4 Hz), 53.2 (d, *J* = 51.2 Hz), 46.9, 30.2, 22.7 (d, *J* = 5.1 Hz), 18.5, 11.4 (d, *J* = 14.2 Hz) ppm; ^31^P{^1^H} NMR (161.9 MHz, CDCl_3_) δ 24.9 ppm; IR (ATR) 2880, 1692, 1509, 1438, 1405, 1271, 1109, 1047, 1035, 997 cm^–1^. HRMS (ESI-TOF) *m*/*z* Calcd for C_25_H_27_NOP^+^ [M^+^] 388.1830, found 388.1831.

#### (2-Oxopyrrolidin-1-yl)methyltriphenylphosphonium tetrafluoroborate (**1ad**)

Colorless crystals (407.0 mg, 91% yield), mp 170.0–172.0 °C. ^1^H NMR (400 MHz, CDCl_3_) δ 7.90–7.62 (m, 15H), 5.33 (d, *J* = 3.6 Hz, 2H), 3.31 (t, *J* = 6.9 Hz, 2H), 2.16–2.15 (m, 2H), 1.93–1.80 (m, 2H) ppm; ^13^C{^1^H} NMR (100 MHz, CDCl_3_) δ 176.6 (d, *J* = 1.7 Hz), 135.5 (d, *J* = 3.1 Hz), 134.0 (d, *J* = 10.0 Hz), 130.5 (d, *J* = 12.6 Hz), 116.7 (d, *J* = 83.8 Hz), 48.8, 39.5 (d, *J* = 58.9 Hz), 29.4, 18.2 ppm; ^31^P{^1^H} NMR (161.9 MHz, CDCl_3_) δ 18.0 ppm; IR (ATR) 1671, 1439, 1425, 1289, 1271, 1112, 1032, 997, 982 cm^–1^. HRMS (ESI-TOF) *m*/*z* Calcd for C_23_H_23_NOP^+^ [M^+^] 360.1517, found 360.1518.

#### 1-(2-Oxopyrrolidin-1-yl)-1-(1-naphthyl)methyltriphenylphosphonium tetrafluoroborate (**1ae**)

Colorless crystals (378.4 mg, 66% yield), mp 210.0–212.0 °C. ^1^H NMR (400 MHz, CD_3_CN) δ 8.12–8.07 (m, 1H), 8.05–7.98 (m, 2H), 7.89–7.79 (m, 4H), 7.78–7.71 (m, 6H), 7.65–7.55 (m, 8H), 7.47–7.42 (m, 1H), 7.39–7.34 (m, 1H), 3.08 (td, *J* = 8.6, 3.5 Hz, 1H), 2.97–2.88 (m, 1H), 2.32–2.22 (m, 1H), 2.21–2.11 (m, 1H), 1.92–1.75 (m, 2H) ppm; ^13^C{^1^H} NMR (100 MHz, CD_3_CN) δ 177.5 (d, *J* = 3.0 Hz), 136.1 (d, *J* = 3.1 Hz), 135.8 (d, *J* = 9.6 Hz), 135.3, 132.3 (d, *J* = 1.7 Hz), 132.2 (d, *J* = 8.8 Hz), 131.3 (d, *J* = 5.7 Hz), 131.0 (d, *J* = 12.5 Hz), 130.4, 128.9, 127.9, 126.6 (d, *J* = 3.3 Hz), 125.8 (d, *J* = 1.6 Hz), 123.6, 119.4 (d, *J* = 82.5 Hz), 51.8 (d, *J* = 61.1 Hz), 47.6, 30.5, 18.7 ppm; ^31^P{^1^H} NMR (161.9 MHz, CD_3_CN) δ 23.6 ppm; IR (ATR) 3069, 2906, 1698, 1437, 1381, 1257, 1099, 1050, 997 cm^–1^. HRMS (ESI-TOF) *m*/*z* Calcd for C_33_H_29_NOP^+^ [M^+^] 486.1987, found 486.1989.

#### 1-(*N*-Phthalimido)propyltriphenylphosphonium tetrafluoroborate (**1ag**)

Colorless crystals (155.8 mg, 29% yield), mp 205.0–207.0 °C. ^1^H NMR (400 MHz, CDCl_3_) δ 7.87–7.75 (m, 7H), 7.75–7.67 (m, 12H), 5.85 (ddd, *J* = 12.5, 11.2, 3.5 Hz, 1H), 2.75–2.55 (m, 1H), 2.21–2.06 (m, 1H), 1.08 (td, *J* = 7.2, 0.8 Hz, 3H) ppm; ^13^C{^1^H} NMR (100 MHz, CDCl_3_) δ 167.0 (d, *J* = 1.3 Hz), 136.0 (d, *J* = 3.1 Hz), 135.6, 134.3 (d, *J* = 9.9 Hz), 130.8 (d, *J* = 12.6 Hz), 130.1, 124.2, 116.0 (d, *J* = 83.0 Hz), 50.4 (d, *J* = 52.8 Hz), 23.8 (d, *J* = 4.3 Hz), 11.6 (d, *J* = 13.1 Hz) ppm;^31^P{^1^H} NMR (161.9 MHz, CDCl_3_) δ 27.6 ppm; IR (ATR) 2964, 1779, 1716, 1440, 1379, 1355, 1335, 1109, 1053, 1031, 997 cm^–1^. HRMS (ESI-TOF) *m*/*z* Calcd for C_29_H_25_NO_2_P [M^+^] 450.1625, found 450.1624.

#### 1-(*N*-Phthalimido)ethyltris(3-chlorophenyl)phosphonium tetrafluoroborate (**1ah**)^24^

Colorless crystals (263.2 mg, 42% yield), mp 232.0–234.0 °C. ^1^H NMR (400 MHz, CD_3_CN) δ 7.93–7.87 (m, 3H), 7.86–7.77 (m, 7H), 7.76–7.65 (m, 6H), 6.24 (dq, *J* = 10.0, 7.4 Hz, 1H), 1.99 (dd, *J* = 17.2, 7.4 Hz, 3H) ppm; ^13^C{^1^H} NMR (100 MHz, CD_3_CN) δ 167.9 (d, *J* = 1.2 Hz), 137.4 (d, *J* = 16.8 Hz), 137.1 (d, *J* = 3.0 Hz), 136.4, 134.9 (d, *J* = 11.4 Hz), 134.3 (d, *J* = 9.6 Hz), 133.2 (d, *J* = 14.0 Hz), 131.8, 124.8, 118.7 (d, *J* = 82.4 Hz), 45.4 (d, *J* = 54.2 Hz), 15.8 (d, *J* = 3.6 Hz) ppm; ^31^P{^1^H} NMR (161.9 MHz, CD_3_CN) δ 27.7 ppm; IR (ATR) 3067, 1774, 1707, 1563, 1467, 1402, 1381, 1342, 1131, 1040, 992 cm^–1^.

#### 1-(*N*-Succinimido)propyltriphenylphosphonium tetrafluoroborate (**1aj**)

Colorless crystals (166.3 mg, 34% yield), mp 232.0–234.0 °C. ^1^H NMR (400 MHz, CD_3_CN) δ 7.97–7.89 (m, 3H), 7.79–7.66 (m, 12H), 5.65 (ddd, *J* = 12.1, 11.3, 3.5 Hz, 1H), 2.64–2.37 (m, 5H), 2.03–1.92 (m, 1H), 0.99 (td, *J* = 7.2, 1.1 Hz, 3H) ppm; ^13^C{^1^H} NMR (100 MHz, CD_3_CN) δ 178.1 (d, *J* = 1.1 Hz), 136.8 (d, *J* = 3.1 Hz), 135.6 (d, *J* = 9.9 Hz), 131.5 (d, *J* = 12.6 Hz), 117.5 (d, *J* = 83.4 Hz), 51.2 (d, *J* = 53.7 Hz), 28.5, 23.5 (d, *J* = 3.2 Hz), 11.7 (d, *J* = 13.0 Hz) ppm; ^31^P{^1^H} NMR (161.9 MHz, CD_3_CN) δ 26.8 ppm; IR (ATR) 2883, 1711, 1439, 1367, 1338, 1176, 1107, 1049, 1039, 998 cm^–1^. HRMS (ESI-TOF) *m*/*z* Calcd for C_25_H_25_NO_2_P [M^+^] 402.1623, found 402.1624.

#### 1-(*N*-Succinimido)methyltriphenylphosphonium bromide (**1ak**)

Colorless crystals (318.0 mg, 70% yield), mp 237.0–239.0 °C. ^1^H NMR (400 MHz, CDCl_3_) 7.93–7.80 (m, 9H), 7.79–7.68 (m, 6H), 5.79 (d, *J* = 4.9 Hz, 2H), 2.57 (s, 4H) ppm; ^13^C{^1^H} NMR (100 MHz, CDCl_3_) δ 175.9, 135.6 (d, *J* = 3.1 Hz), 134.2 (d, *J* = 10.3 Hz), 130.4 (d, *J* = 12.9 Hz), 116.4 (d, *J* = 85.5 Hz), 36.2 (d, *J* = 56.7 Hz), 28.2 ppm; ^31^P{^1^H} NMR (161.9 MHz, CDCl_3_) δ 20.9 ppm; IR (ATR) 3069, 1712, 1436, 1395, 1315, 1148, 1112, 1047, 996 cm^–1^. HRMS (ESI-TOF) *m*/*z* Calcd for C_23_H_21_NO_2_P [M^+^] 374.1310, found 374.1313.

#### 2-Carbamoyletyhyltriphenylphosphonium tetrafluoroborate (**14**)

Colorless crystals (328.5 mg, 78% yield), mp 146.0–148.0 °C. ^1^H NMR (400 MHz, CDCl_3_) δ 7.90–7.78 (m, 3H), 7.77–7.60 (m, 12H), 7.02 (br s, 1H), 5.28 (br s, 1H), 3.55–3.37 (m, 2H), 2.90–2.78 (m, 2H) ppm; ^13^C{^1^H} NMR (100 MHz, CDCl_3_) δ 171.3 (d, *J* = 14.3 Hz), 135.5 (d, *J* = 3.1 Hz), 133.5 (d, *J* = 10.0 Hz), 130.7 (d, *J* = 12.7 Hz), 117.5 (d, *J* = 86.7 Hz), 27.4 (d, *J* = 2.9 Hz), 19.3 (d, *J* = 56.0 Hz) ppm; ^31^P{^1^H} NMR (161.9 MHz, CDCl_3_) δ 24.6 ppm; IR (ATR) 3424, 3322, 3195, 1685, 1670, 1622, 1441, 1419, 1111, 1024, 996 cm^–1^. HRMS (ESI-TOF) *m*/*z* Calcd for C_21_H_21_NOP^+^ [M^+^] 334.1361, found 334.1366.

### Synthesis of 1,1′-(Carbonyldimino)bis(methyltriphenylphosphonium) Bis(tetrafluoroborate) (**15**) from Urea

The one-pot reaction was carried out in a glass vial sealed with a screw-cap. Urea (30.0 mg, 0.5 mmol) and triphenylphosphonium tetrafluoroborate (350.1 mg, 1 mmol) were added to a solution of paraformaldehyde (30.0 mg, 1 mmol of CH_2_O) in CH_3_CN (0.65 cm^3^). The obtained mixture was stirred vigorously and heated at 135 °C for 2 h using an oil bath. The product was precipitated with diethyl ether to afford pure 1,1′-(carbonyldimino)bis(methyltriphenylphosphonium) bis(tetrafluoroborate) **15** in 84% yield.

#### 1,1′-(Carbonyldimino)bis(methyltriphenylphosphonium) bis(tetrafluoroborate) (**15**)

Colorless crystals (658.8 mg, 84% yield), mp 232.0–233.0 °C. ^1^H NMR (400 MHz, CD_3_CN) δ 7.93–7.82 (m, 6H), 7.74–7.57 (m, 24H), 5.83 (br t, *J* = 6.5 Hz, 2H), 4.99 (dd, *J* = 6.6, 2.7 Hz, 4H) ppm; ^13^C{^1^H} NMR (100 MHz, CD_3_CN) δ 157.3 (t, *J* = 2.8 Hz), 136.4 (d, *J* = 2.9 Hz), 135.2 (d, *J* = 10.1 Hz), 131.2 (d, *J* = 12.8 Hz), 117.7 (d, *J* = 84.7 Hz), 37.6 (d, *J* = 60.5 Hz) ppm; ^31^P{^1^H} NMR (161.9 MHz, CD_3_CN) δ 18.8 ppm; IR (ATR) 3378, 3058, 1686, 1546, 1438, 1111, 1049, 996 cm^–1^. HRMS (ESI-TOF) *m*/*z* Calcd for C_39_H_36_N_2_OP_2_^2+^ [M^2+^] 305.1152, found 305.1167.

### Synthesis of 1-(*N*-Acetylamino)propyltriphenylphosphonium Bromide **1b** via the Reaction of 1-Hydroxypropyltriphenylphosphonium Bromide **11a** with Acetamide

Reactions were carried out in a glass vial sealed with a screw-cap. Triphenylphosphonium bromide (343.2 mg, 1 mmol) was added to a solution of propionaldehyde (0.0717 cm^3^, 58.1 mg, 1 mmol) in CH_3_CN (0.65 cm^3^). The obtained mixture was stirred vigorously at room temperature for 1 h. Then, the product (**11a**) was precipitated with diethyl ether to obtain pure 1-hydroxypropyltriphenylphosphonium bromide in 90% yield. The salt **11a** (100.3 mg, 0.25 mmol) formed in the first step was dissolved in acetonitrile (0.16 cm^3^) in a glass vial sealed with a screw-cap. Then, acetamide (14.8 mg, 0.25 mmol) was added and the reaction mixture was stirred and heated at 50 °C for 1 h using an oil bath. The product was precipitated with diethyl ether to obtain pure 1-(*N*-acetylamino)propyltriphenylphosphonium tetrafluoroborate **1b** in 98% yield.

#### 1-Hydroxypropyltriphenylphosphonium bromide (**11a**)

Colorless crystals (361.2 mg, 90% yield), mp 157.0–159.0 °C. ^1^H NMR (400 MHz, CDCl_3_) δ 7.88–7.75 (m, 9H), 7.71–7.63 (m, 6H), 7.61–7.54 (m, 1H), 5.94–5.86 (m, 1H), 1.93–1.79 (m, 2H), 1.23 (t, *J* = 7.2 Hz, 3H) ppm; ^13^C{^1^H} NMR (100 MHz, CDCl_3_) δ 134.8 (d, *J* = 3.0 Hz), 134.3 (d, *J* = 9.0 Hz), 130.1 (d, *J* = 12.0 Hz), 117.7 (d, *J* = 80.5 Hz), 68.3 (d, *J* = 60.3 Hz), 25.6 (d, *J* = 6.1 Hz), 10.6 (d, *J* = 14.5 Hz) ppm; ^31^P{^1^H} NMR (161.9 MHz, CDCl_3_) δ 20.9 ppm; IR (ATR) 3072, 2959, 1438, 1438, 1111 cm^–1^. HRMS (ESI-TOF) *m*/*z* Calcd for C_21_H_22_OP^+^ [M^+^] 321.1408, found 321.1408.

## Supporting Information Available

^1^H, ^13^C{^1^H}, ^31^P{^1^H} NMR and IR spectra of compounds **1**, **2**, **10a**–**c**, **11a**, **14**, and **15**; HR-MS spectra for all unknown compounds; Apparatus for the one-pot synthesis of *N*-protected 1-aminoalkylphosphonium salts **1**; A brief comparison of the selected (most important) methods for the synthesis of *N*-protected 1-aminoalkylphosphonium salts (Table S1) (PDF)
