# Supplementary material for: Synthesis of N-Protected 1-Aminoalkylphosphonium Salts from Amides, Carbamates, Lactams, or Imides
Source: J Org Chem. 2021 Apr 8;86(8):5852–62. doi: 10.1021/acs.joc.1c00285 (PMC8154577; doi:10.1021/acs.joc.1c00285)
Supplement: Supplementary file 1 — jo1c00285_si_001.pdf [file jo1c00285_si_001.pdf]

Synthesis of *N*-protected 1-aminoalkylphosphonium salts from amides, carbamates, lactams, or imides

Jakub Adamek,[1,2,] Paulina Zieleźny,[1] and Karol Erfurt[3]*

[1]Department of Organic Chemistry, Bioorganic Chemistry and Biotechnology, Silesian University of Technology, B. Krzywoustego 4, 44-100 Gliwice, Poland

[2]Biotechnology Centre of Silesian University of Technology, B. Krzywoustego 8, 44-100 Gliwice, Poland

[3]Department of Chemical Organic Technology and Petrochemistry, Silesian University of Technology, B. Krzywoustego 4, 44-100 Gliwice, Poland

[*] Corresponding author: Jakub Adamek, e-mail: jakub.adamek@polsl.pl

Supporting information

Experimental and analytical data

Table of contents

1. Apparatus for the one-pot synthesis of *N*-protected 1-aminoalkylphosphonium salts **1**.……..……...……………....S2
2. A brief comparison of the selected (most important) methods for the synthesis of *N*-protected 1-aminoalkylphosphonium salts……………………………………………………………………………....S3-S4
3. ^{1}H NMR, ^{13}C{^{1}H} NMR, ^{31}P NMR spectra of compounds **1, 10a-c, 11a, 14** and **15**.…………………..…….S5-S122
4. IR spectra of compounds **1, 10a-c, 11a, 14** and **15**………..…………..………………………..…………...S123-S161
5. MS spectra of all unknown compounds.……………………………………………………………..…S162-S190

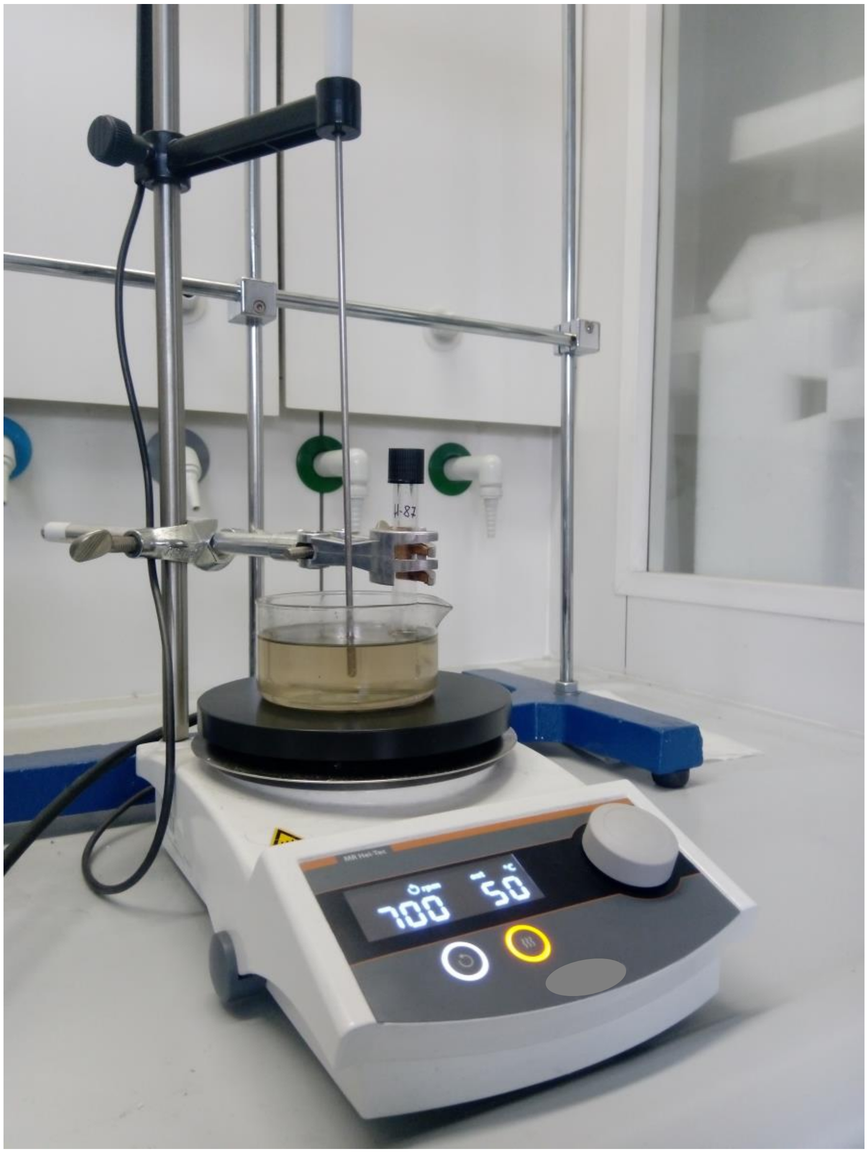

Photo S1. Apparatus for the one-pot synthesis of *N*-protected 1-aminoalkylphosphonium salts **1** (personal photo made by authors).

Table S1. A brief comparison of the selected (most important) methods for the synthesis of *N*-protected 1-aminoalkylphosphonium salts.

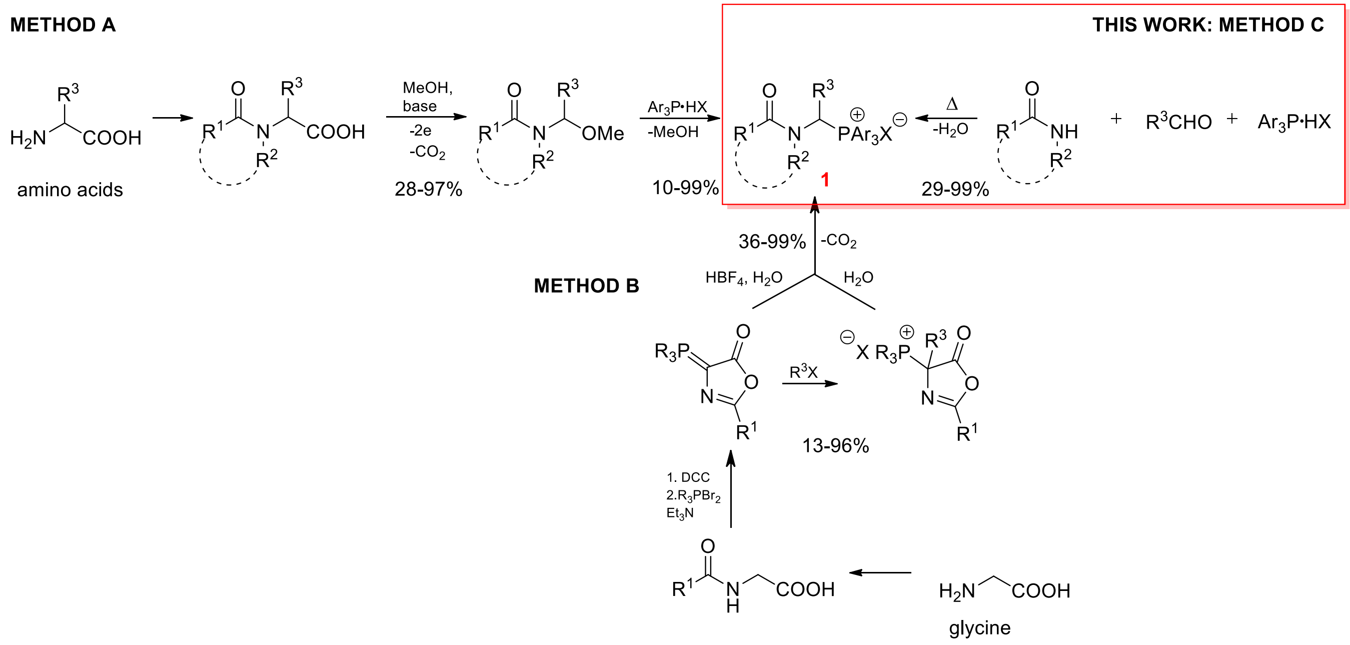

Method	Substrates	Steps/example	Limitations	Literature
A	α-amino acids	1. *N*-protection of α-amino acids (**1h**:<br>2. electrochemical alkoxylation<br>3. C-P bond formation<br>**1g** (R^1= Me, R^2 = R^3 = H, Ar = Ph, X = BF_4):<br>1-2 workdays (reaction and purification time): step 1:[6] r.t., 24h, 89-92%; step 2: 10°C, 2h, 96%, step 3: 60°C, 30 min, 98%; **overall yield: 84%-87%**	electrochemical equipment (electrolyzer, platinum electrodes, power supply)	1-3
B	Glycine, (oxazolones)	1. *N*-protection of glycine<br>2. synthesis of oxazolones<br>(3.) 4C-alkylation<br>4. hydrolysis<br>5. decarboxylation<br>**1g**: 3-4 workdays: step 1:[6] r.t., 24h, 89-92%; step 2: r.t., 24h, 51%, step 3: r.t. 10 min, 97%; step 4: 105°C, 1h, 99%; **overall yield: 44-45%**	multi-step, labor-intensive, time-consuming, narrow scope of application	4,5
C	Aldehydes, amides, carbamates, imides, lactams, urea	1. three-component coupling<br>**1g**: 135 °C, 2-3h, **91%**	difficulties in the preparation of *N*-protected 1-aminoalkylphosphonium salts, which are derivatives of phosphines substituted with electron-withdrawing substituents (solvent-free methodology)	This work

References:

(1) Mazurkiewicz, R.; Adamek, J.; Październiok-Holewa, A.; Zielińska, K.; Simka, W.; Gajos, A.; Szymura, K. α-Amidoalkylating Agents from *N*-Acyl-α-amino Acids: 1-(*N*-Acylamino)alkyltriphenylphosphonium Salts. *J. Org. Chem.* **2012**, *77*, 1952-1960, DOI: 10.1021/jo202534u.

(2) Adamek, J.; Węgrzyk-Schlieter, A.; Steć, K..; Walczak, K.; Erfurt, K. Michaelis-Arbuzov-Type Reaction of 1-Imidoalkyltriarylphosphonium Salts with Selected Phosphorus Nucleophiles. *Molecules* **2019**, *24*, 3405, DOI: 10.3390/molecules24183405.

(3) Walęcka-Kurczyk, A.; Walczak, K.; Kuźnik, A.; Stecko, S.; Październiok-Holewa, A. The Synthesis of α-Aminophosphonates via Enantioselective Organocatalytic Reaction of 1-(*N*-Acylamino)alkylphosphonium Salts with Dimethyl Phosphite. *Molecules,* **2020**, *25*, 405, DOI: 10.3390/molecules25020405.

(4) Mazurkiewicz, R.; Październiok-Holewa, A.; Grymel, M. Synthesis and decarboxylation of *N*-acyl-

α-triphenylphosphonio-α-amino acids: a new synthesis of α-(*N*-acylamino)alkyltriphenylphosphonium salts. *Tetrahedron Lett.* **2008**, *49*, 1801-1803, DOI: 10.1016/j.tetlet.2008.01.051.

(5) Mazurkiewicz, R.; Pierwocha, A.W. Phosphoranylidene-5(4*H*)-oxazolones – A novel synthesis and properties. *Monatsh. Chem.* **1996**, *127*, 219-225, DOI: 10.1007/BF00807402.

(6) Herbst, R.M.; Shemin, D. Acetylglycine. *Org. Synth.* **1939**, *19*, 4, DOI: 10.15227/orgsyn.019.0004.

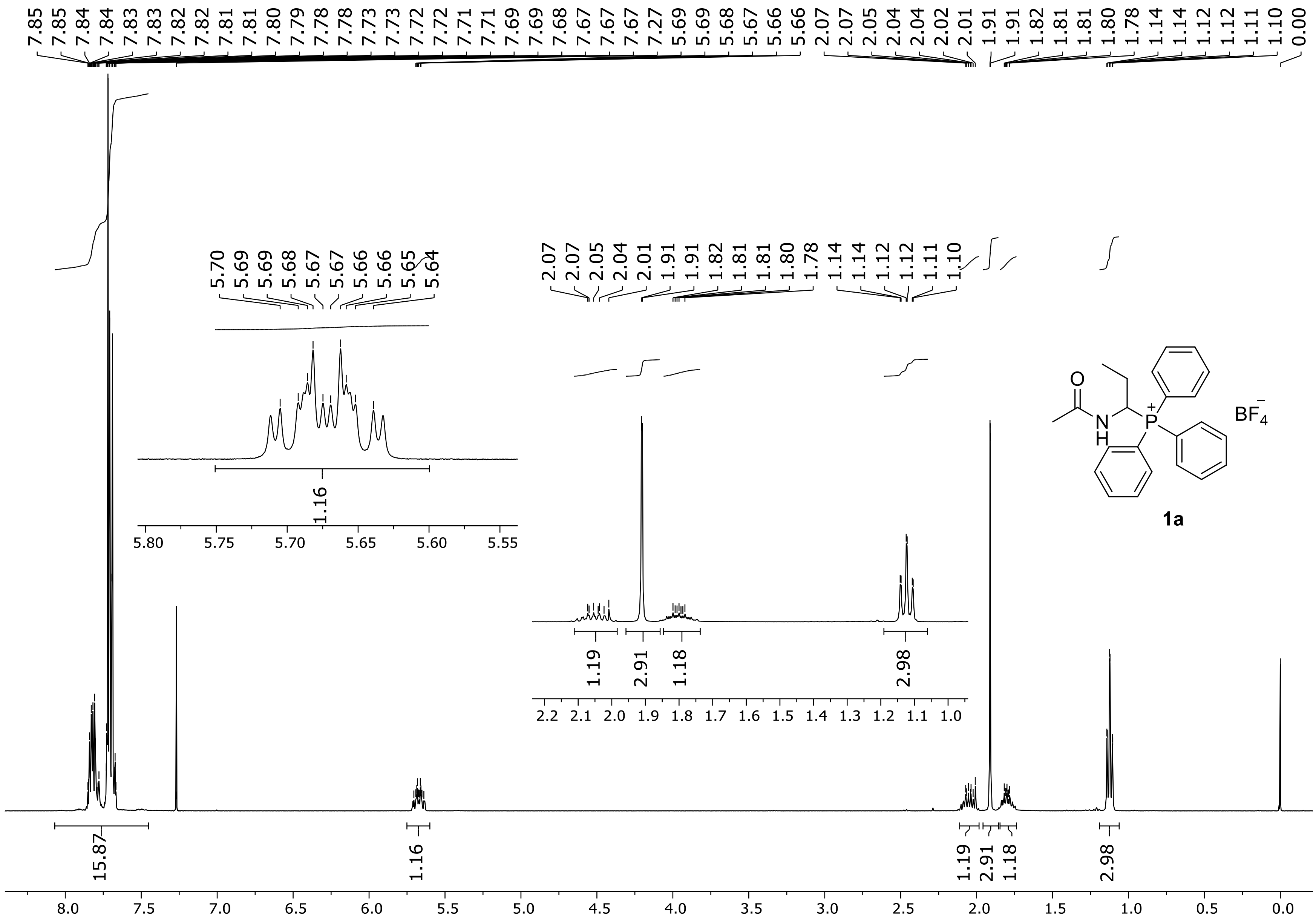

^{1}H NMR spectrum of 1-(*N*-acetylamino)propyltriphenylphosphonium tetrafluoroborate (**1a**); 400 MHz/$CDCl_3$/TMS; δ (ppm).

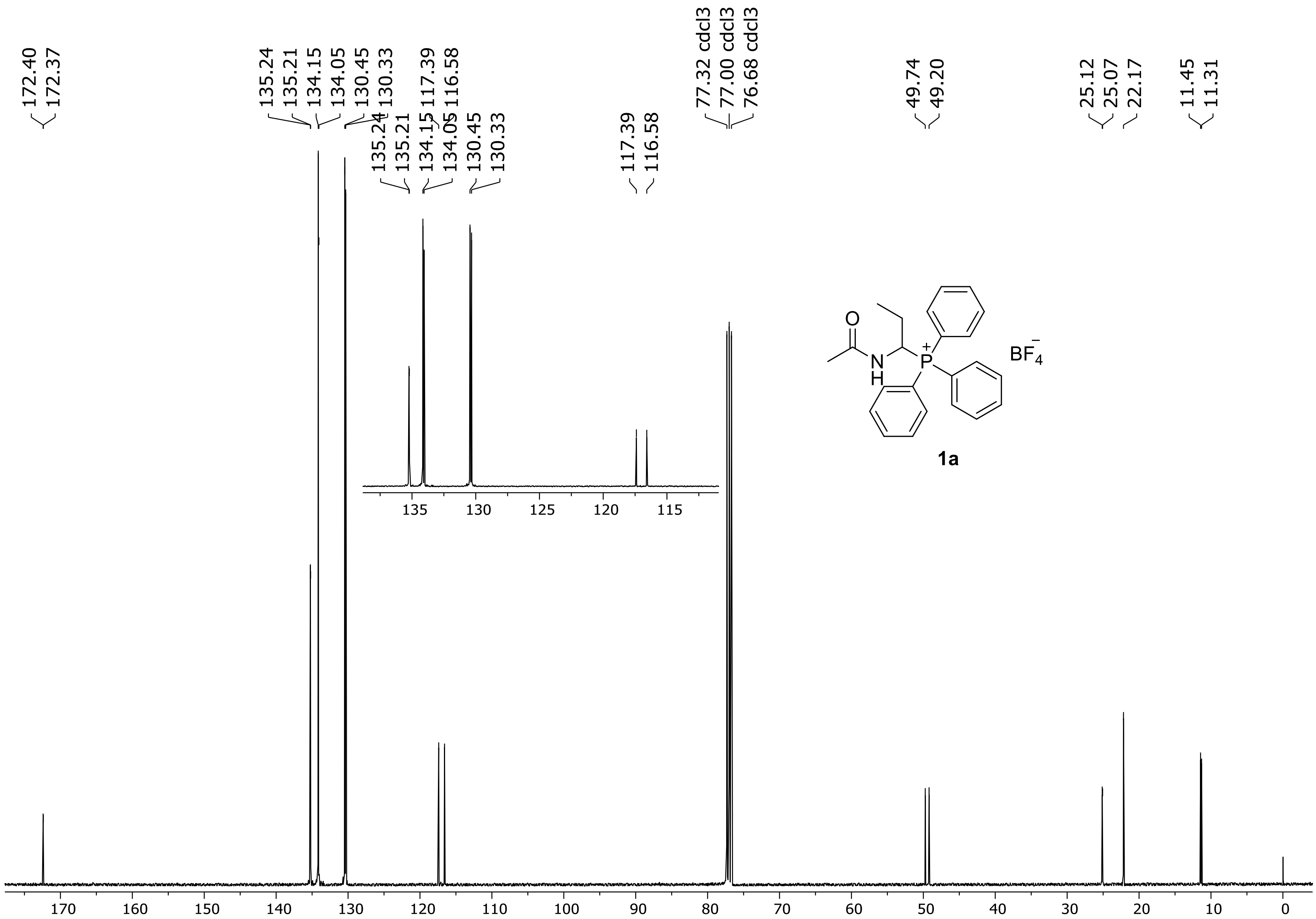

$^{13}C\{^{1}H\}$ NMR spectrum of 1-(*N*-acetylamino)propyltriphenylphosphonium tetrafluoroborate (**1a**); 100 MHz/$CDCl_3$/TMS; δ (ppm).

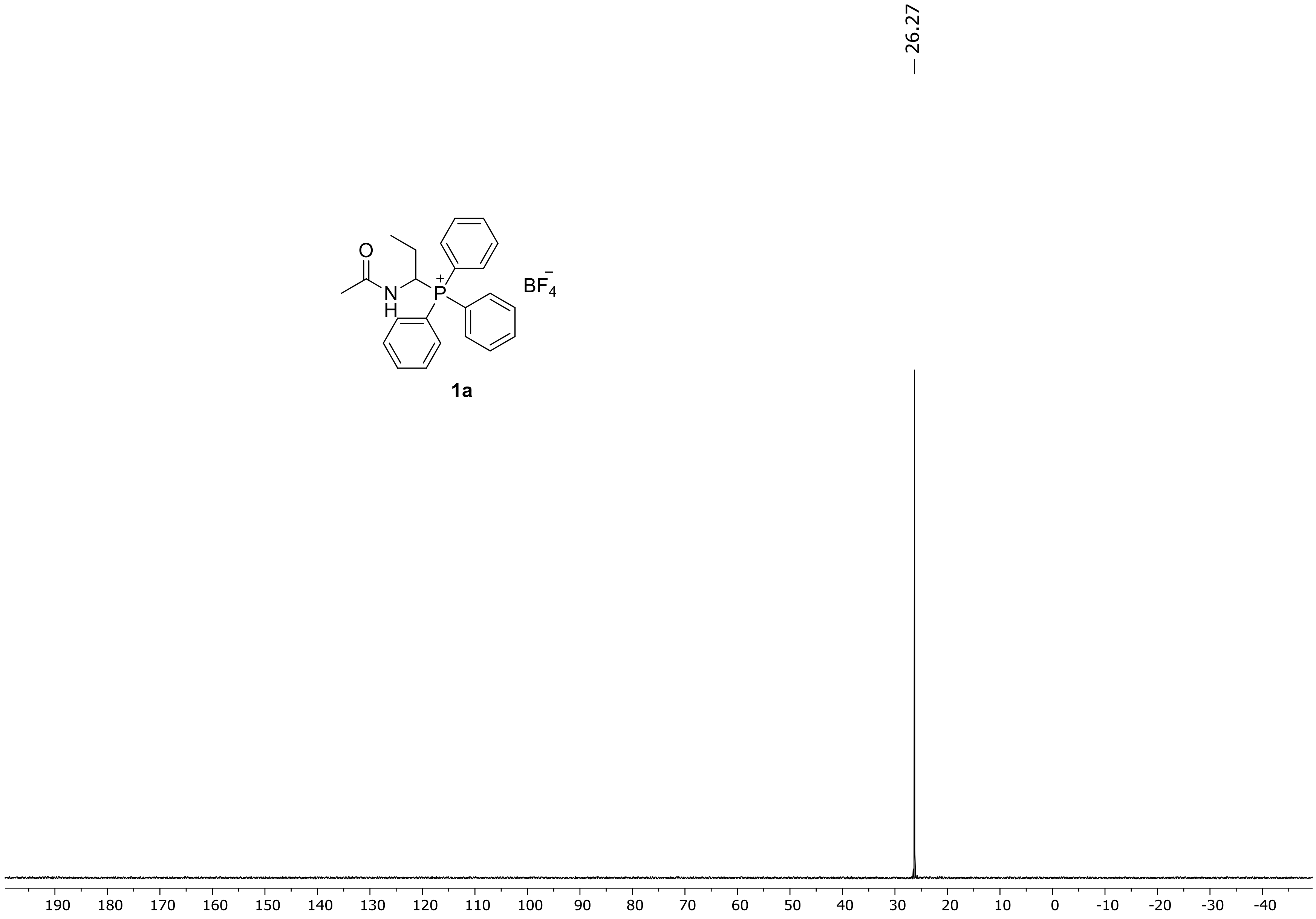

^{31}P NMR spectrum of 1-(*N*-acetylamino)propyltriphenylphosphonium tetrafluoroborate (**1a**); 161.9 MHz/$CDCl_3$; δ (ppm).

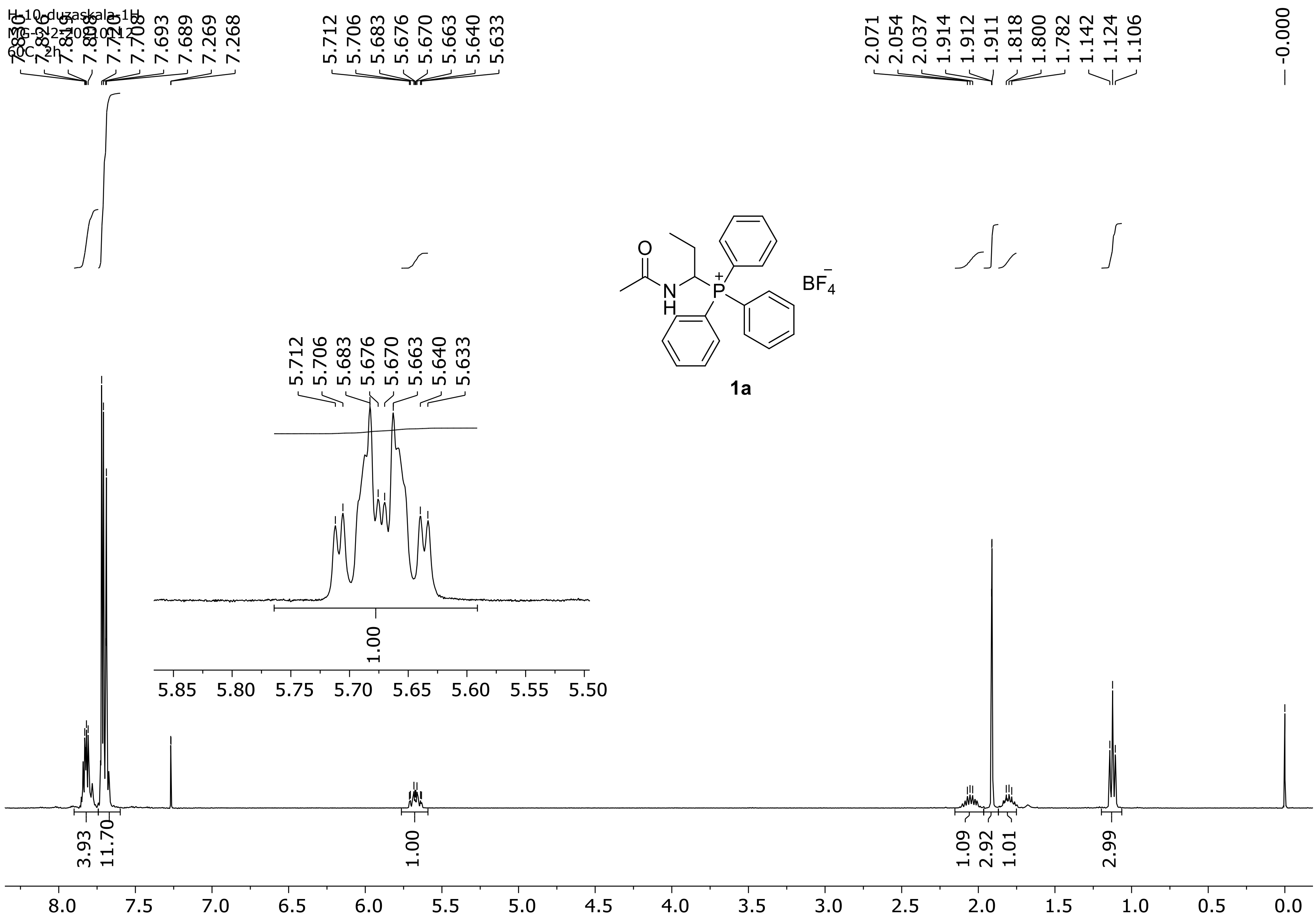

^{1}H NMR spectrum of 1-(*N*-acetylamino)propyltriphenylphosphonium tetrafluoroborate (**1a**); 400 MHz/$CDCl_3$/TMS; δ (ppm) - synthesis on a 20g scale.

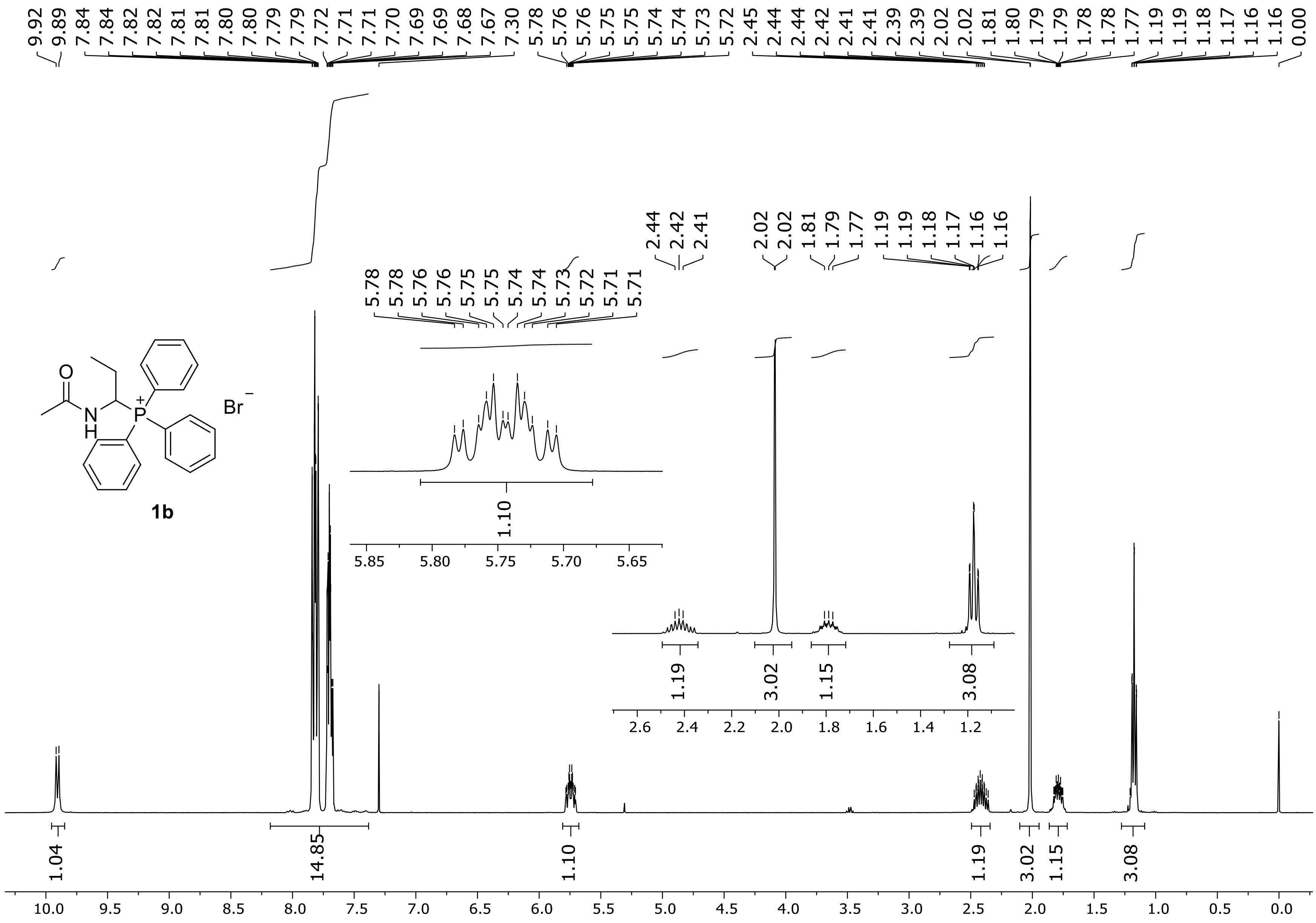

^{1}H NMR spectrum of 1-(*N*-acetylamino)propyltriphenylphosphonium bromide (**1b**); 400 MHz/$CDCl_3$/TMS; δ (ppm).

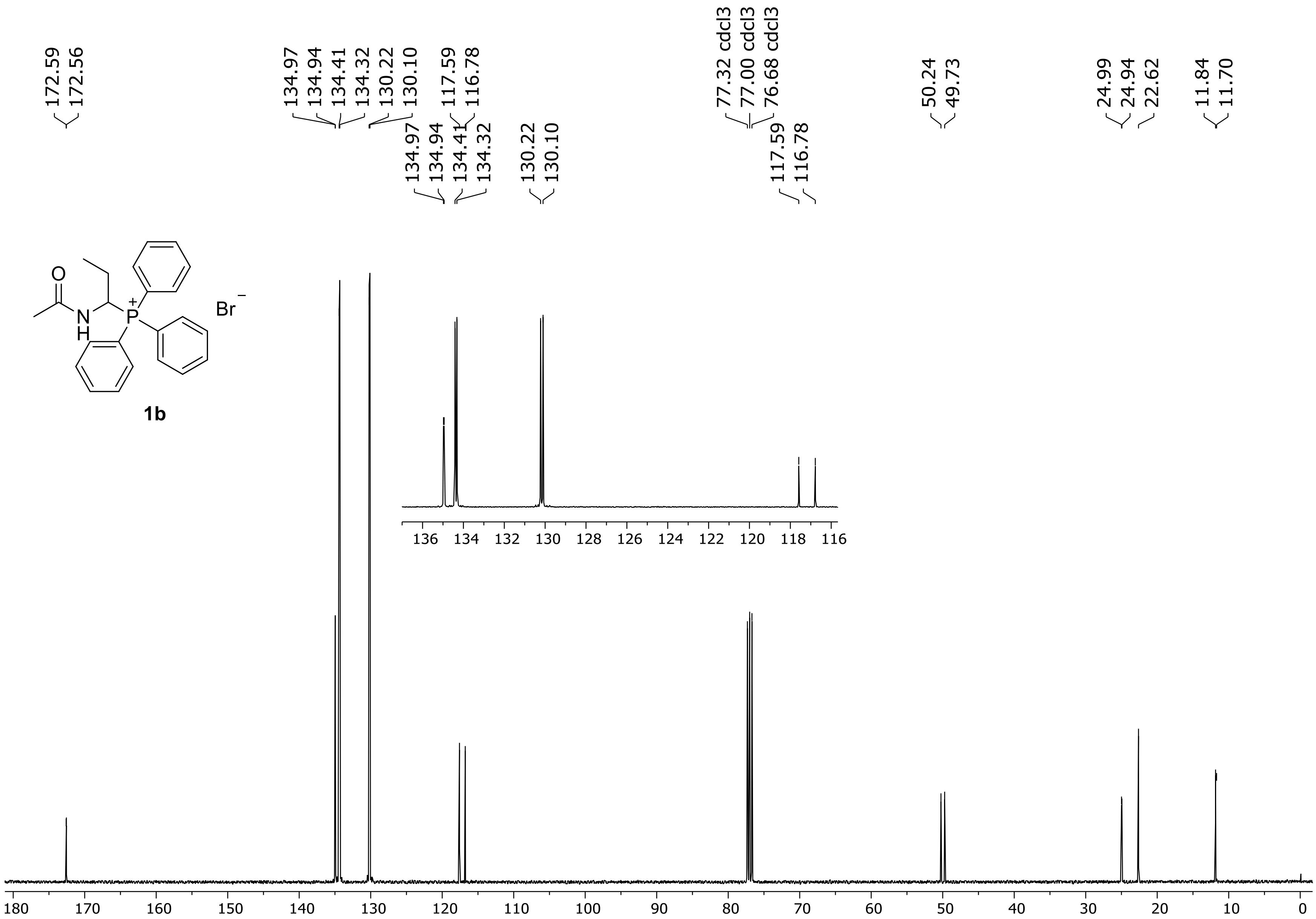

$^{13}C\{^{1}H\}$ NMR spectrum of 1-(*N*-acetylamino)propyltriphenylphosphonium bromide (**1b**); 100 MHz/$CDCl_3$/TMS; δ (ppm).

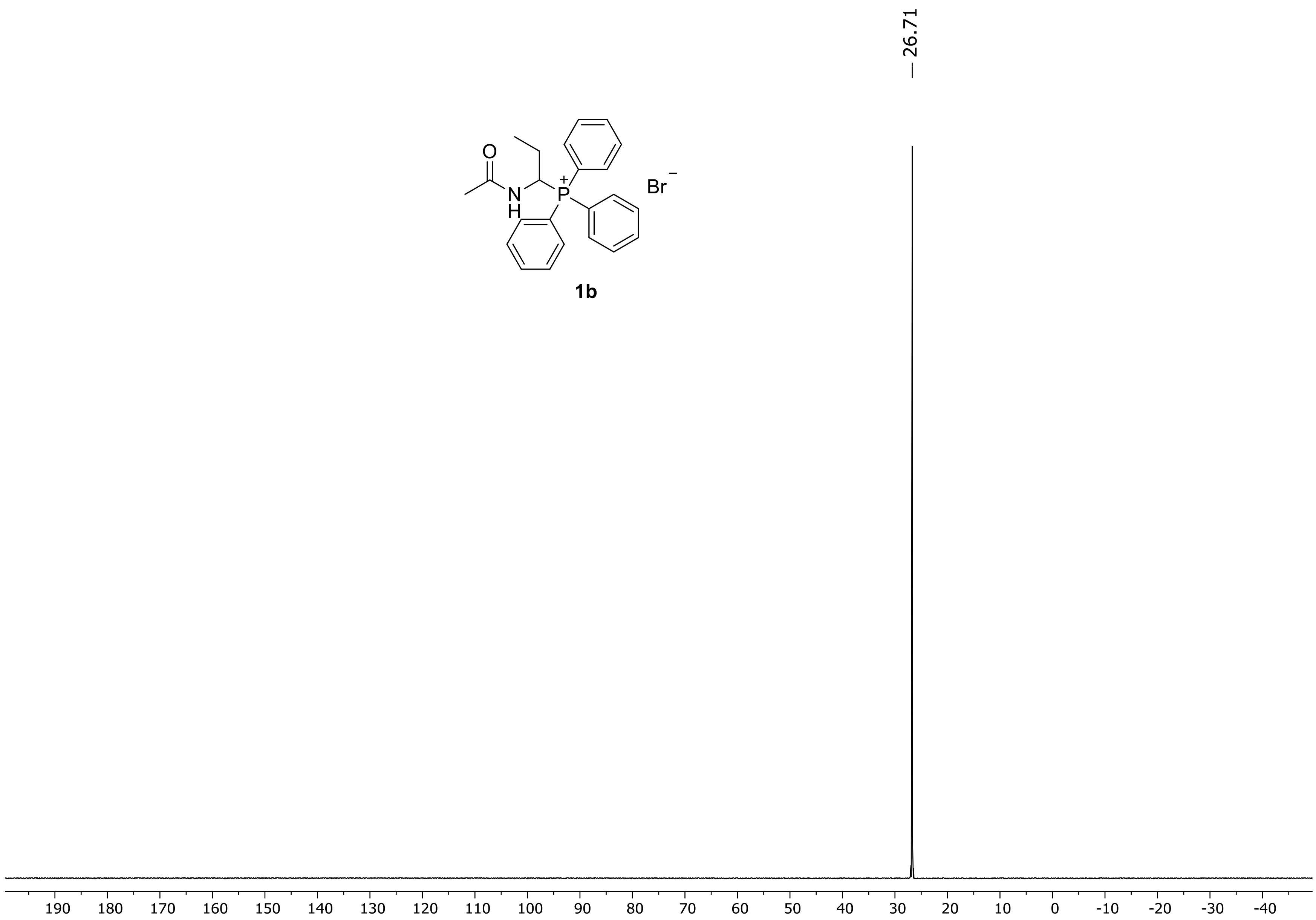

^{31}P NMR spectrum of 1-(*N*-acetylamino)propyltriphenylphosphonium bromide (**1b**); 161.9 MHz/$CDCl_3$; δ (ppm).

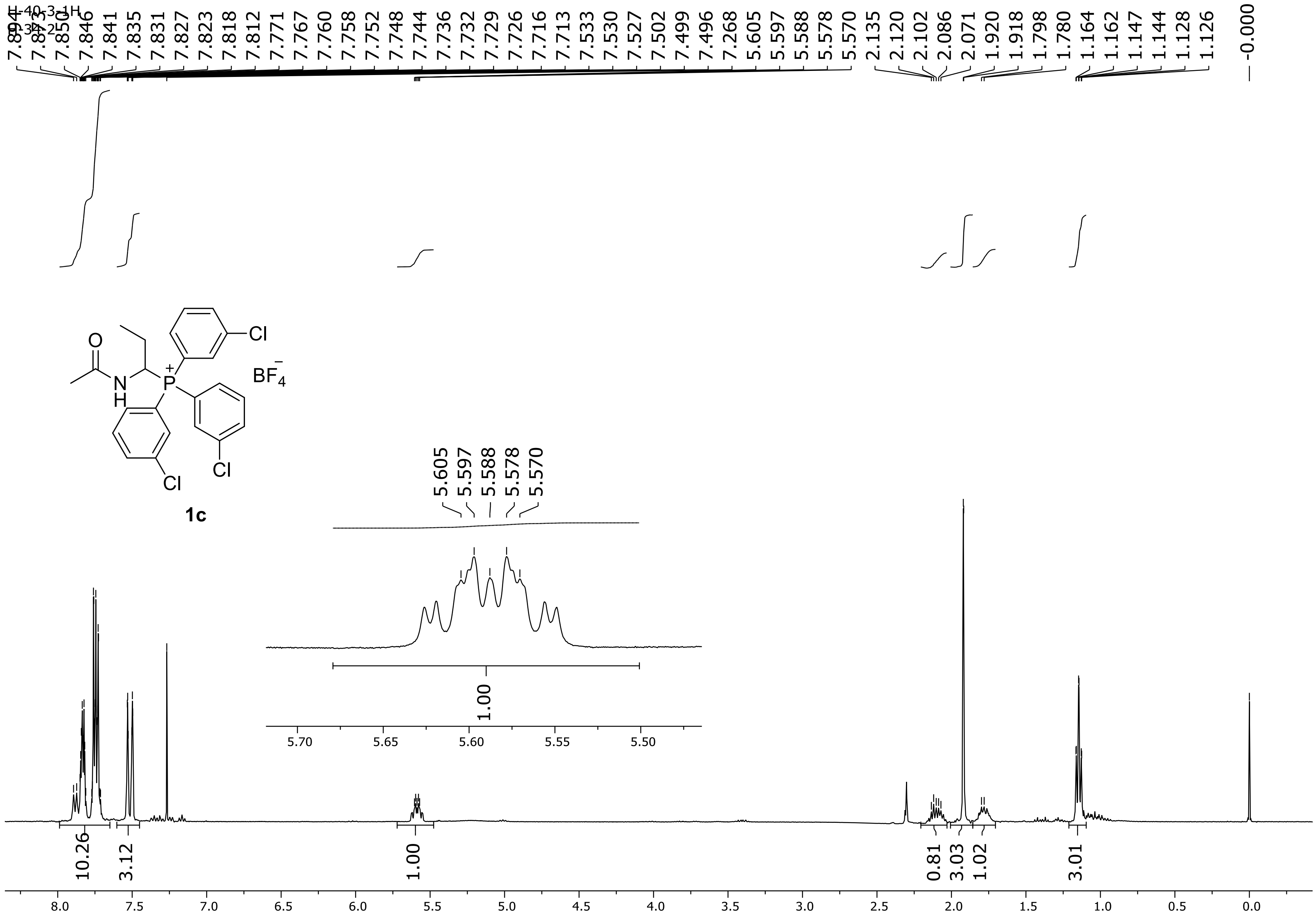

^{1}H NMR spectrum of 1-(*N*-acetylamino)propyltris(3-chlorophenyl)phosphonium tetrafluoroborate (**1c**); 400 MHz/$CDCl_3$/TMS; δ (ppm).

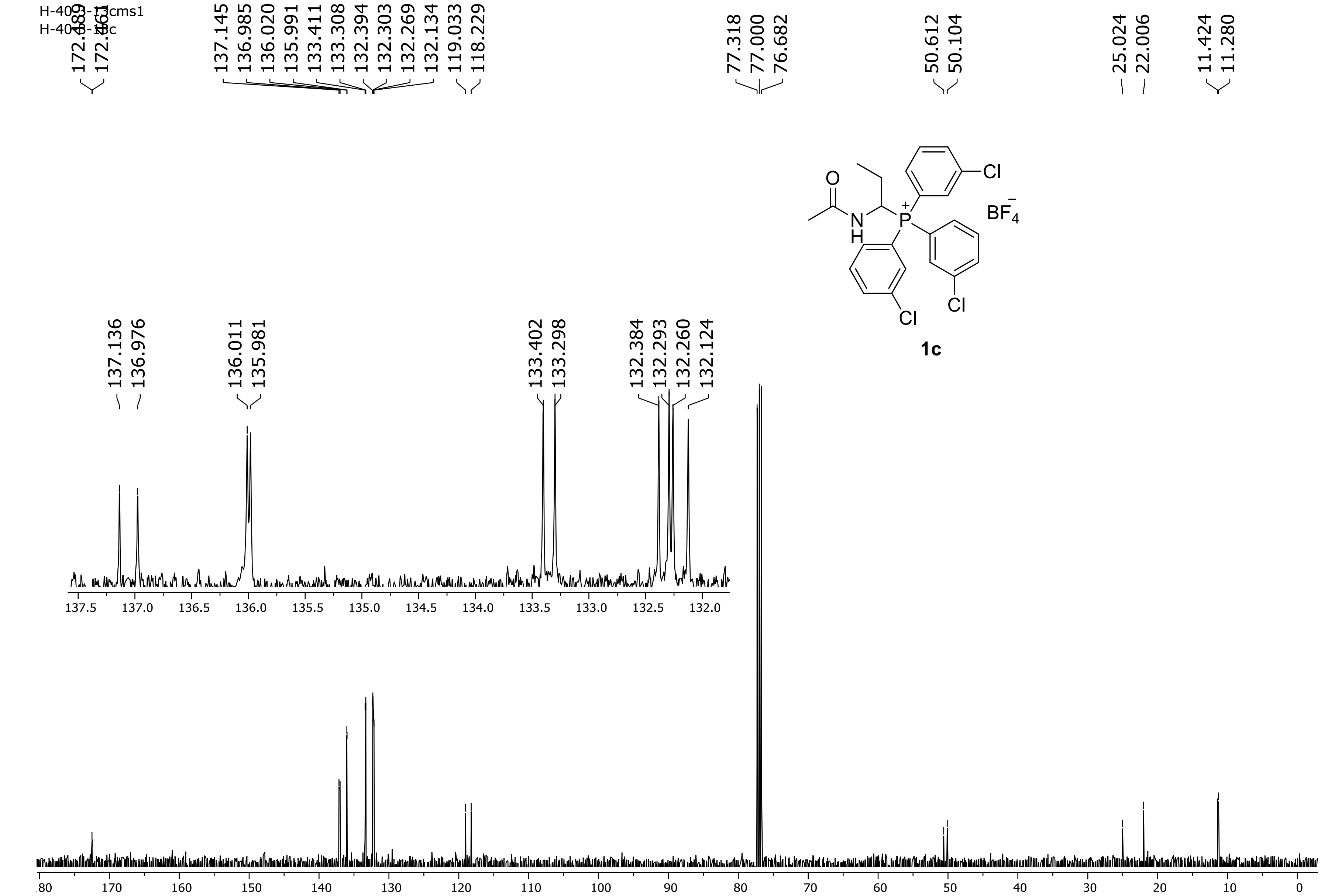

$^{13}C\{^{1}H\}$ NMR spectrum of 1-(*N*-acetylamino)propyltris(3-chlorophenyl)phosphonium tetrafluoroborate (**1c**); 100 MHz/$CDCl_3$/TMS; δ (ppm).

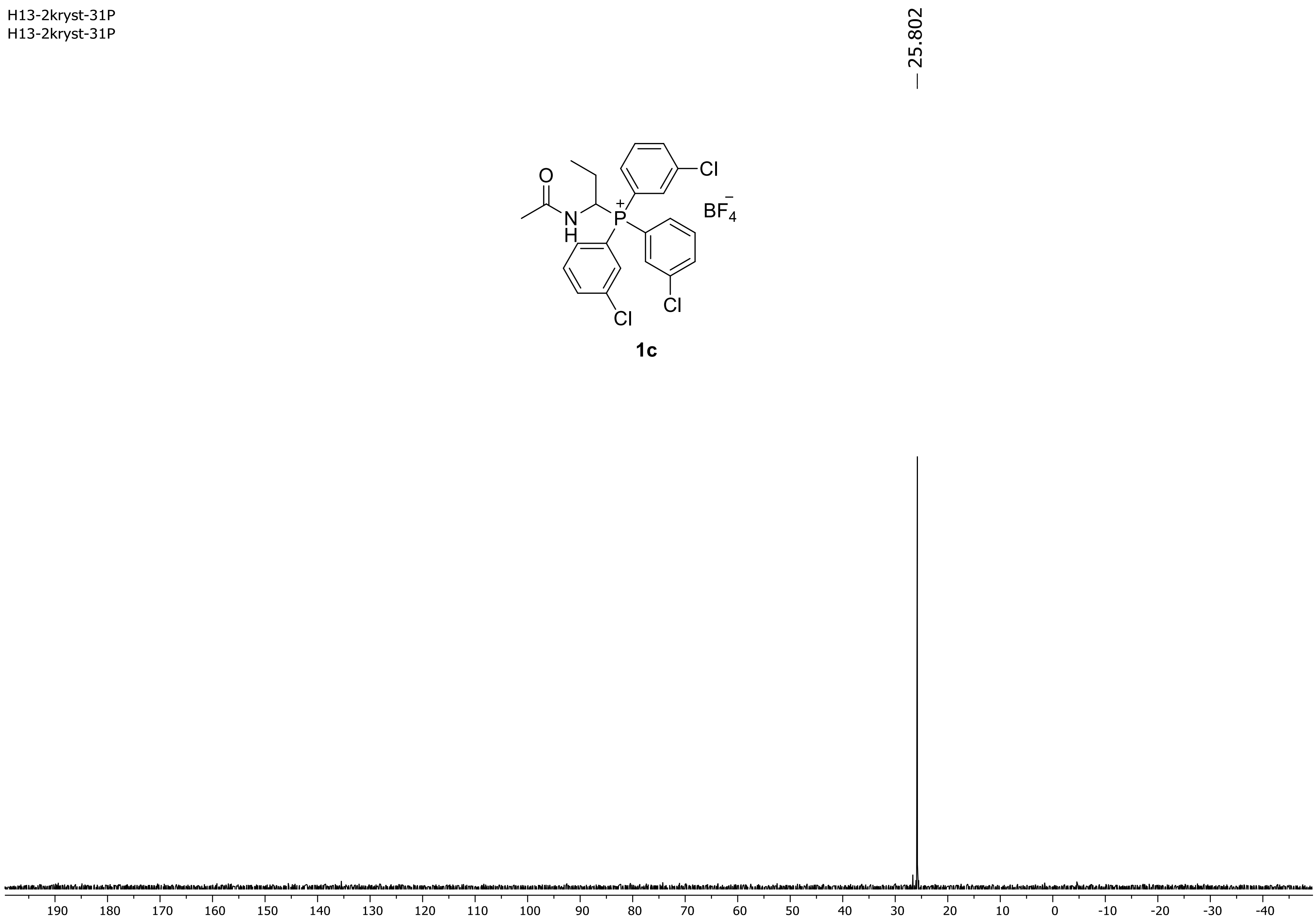

^{31}P NMR spectrum of 1-(*N*-acetylamino)propyltris(3-chlorophenyl)phosphonium tetrafluoroborate (**1c**); 161.9 MHz/$CDCl_3$; δ (ppm).

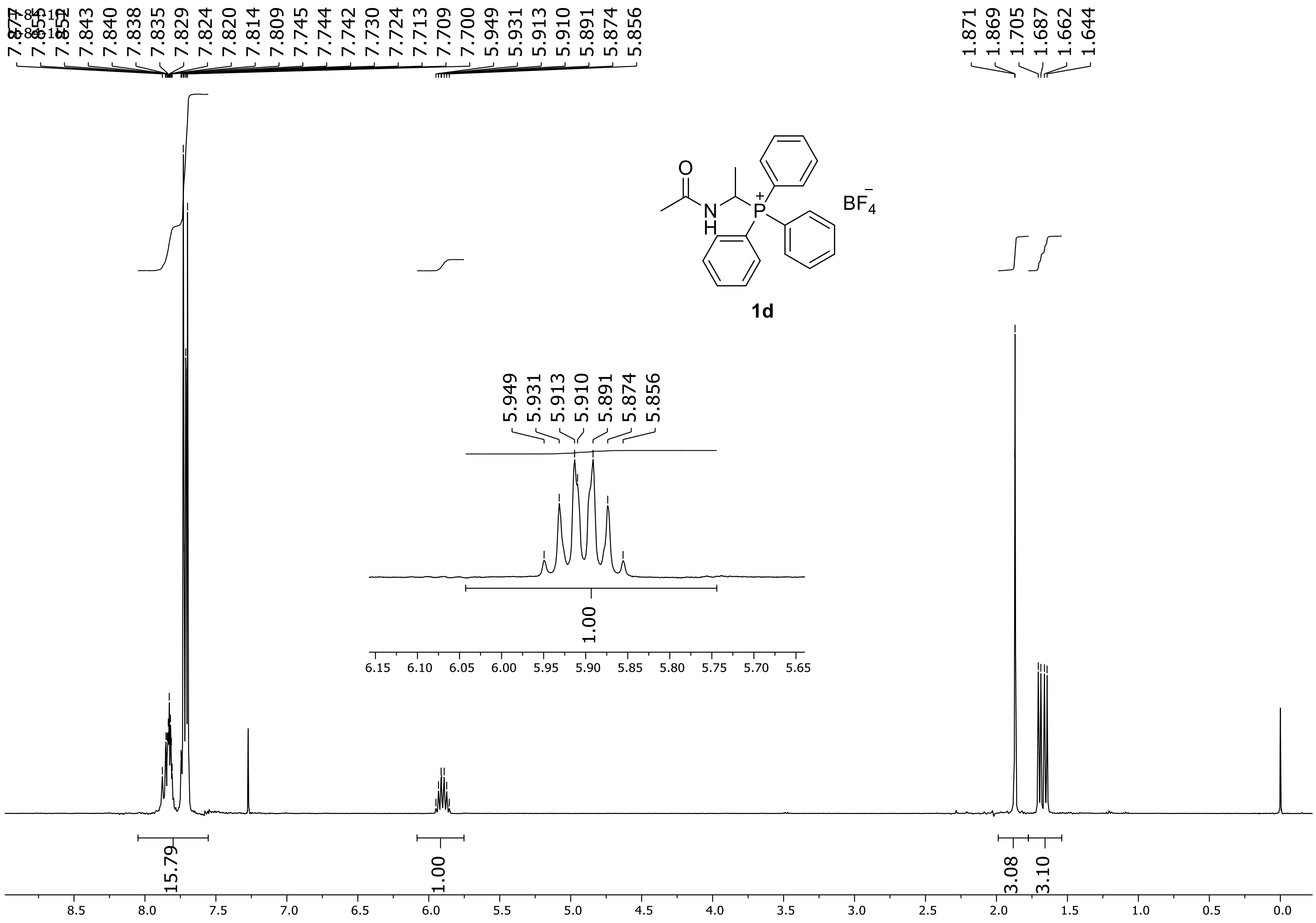

^{1}H NMR spectrum of 1-(*N*-acetylamino)ethyltriphenylphosphonium tetrafluoroborate (**1d**); 400 MHz/$CDCl_3$/TMS; δ (ppm).

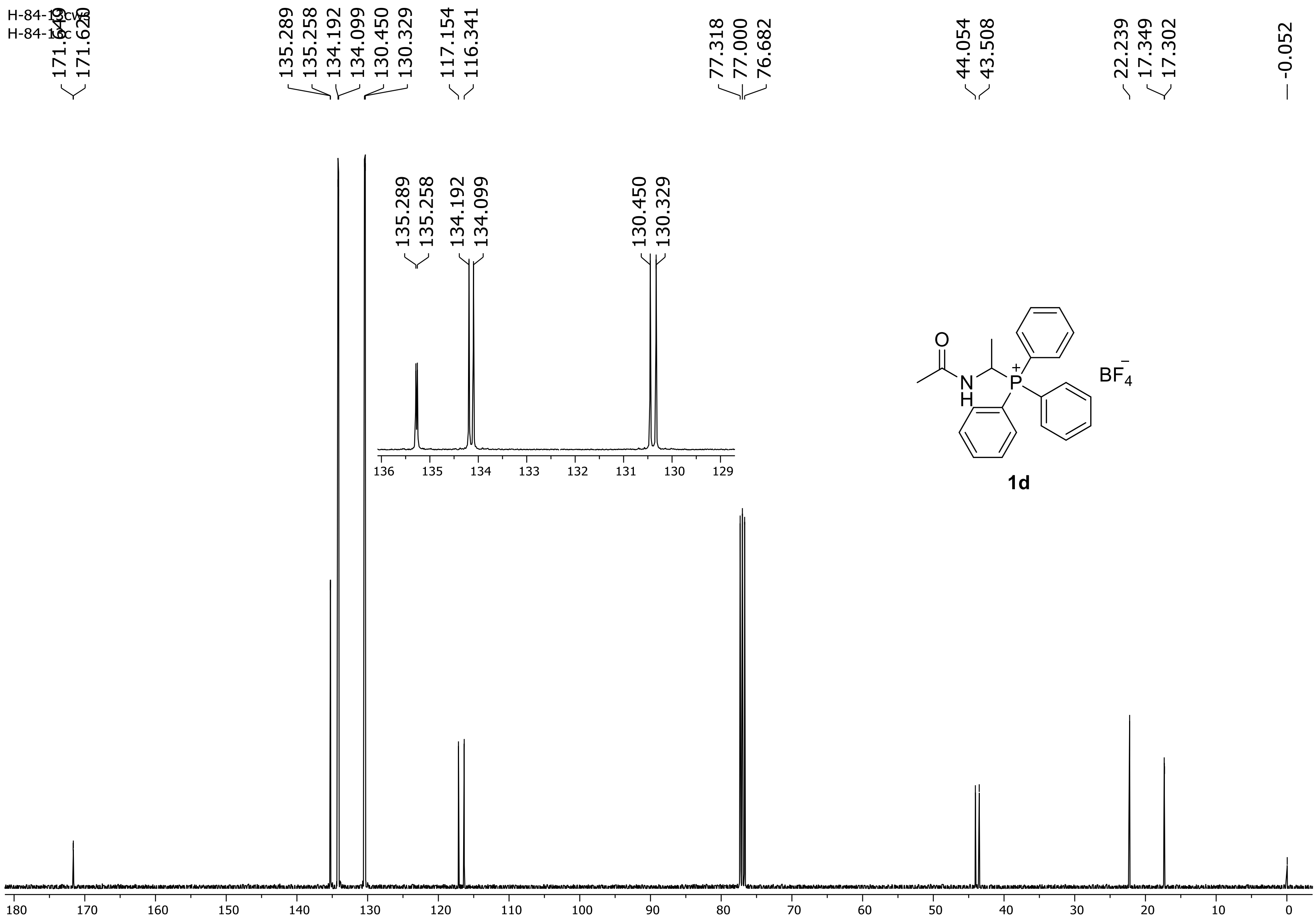

$^{13}C\{^1H\}$ NMR spectrum of 1-(*N*-acetylamino)ethyltriphenylphosphonium tetrafluoroborate (**1d**); 100 MHz/$CDCl_3$/TMS; δ (ppm).

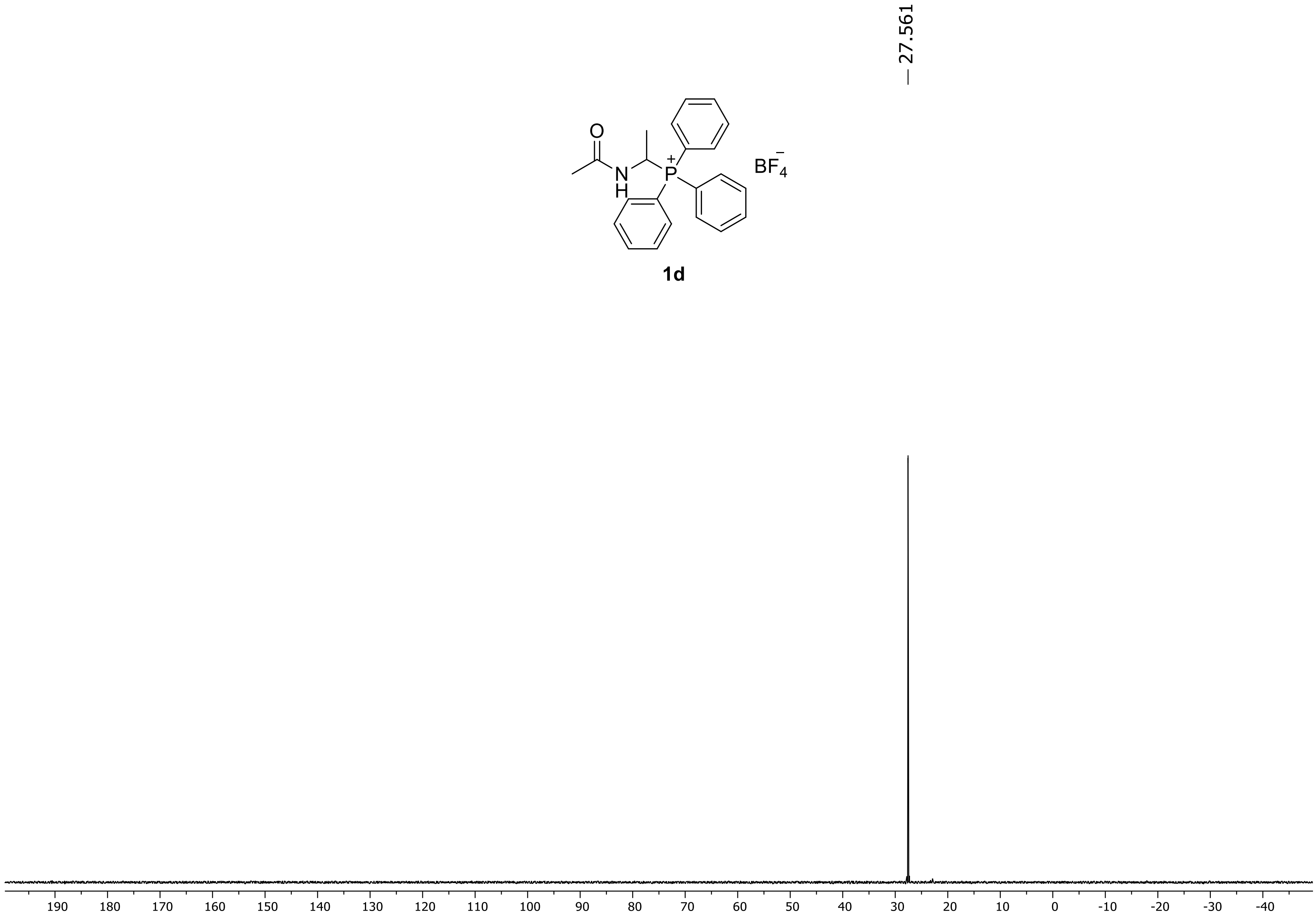

^{31}P NMR spectrum of 1-(*N*-acetylamino)ethyltriphenylphosphonium tetrafluoroborate (**1d**); 161.9 MHz/$CDCl_3$; δ (ppm).

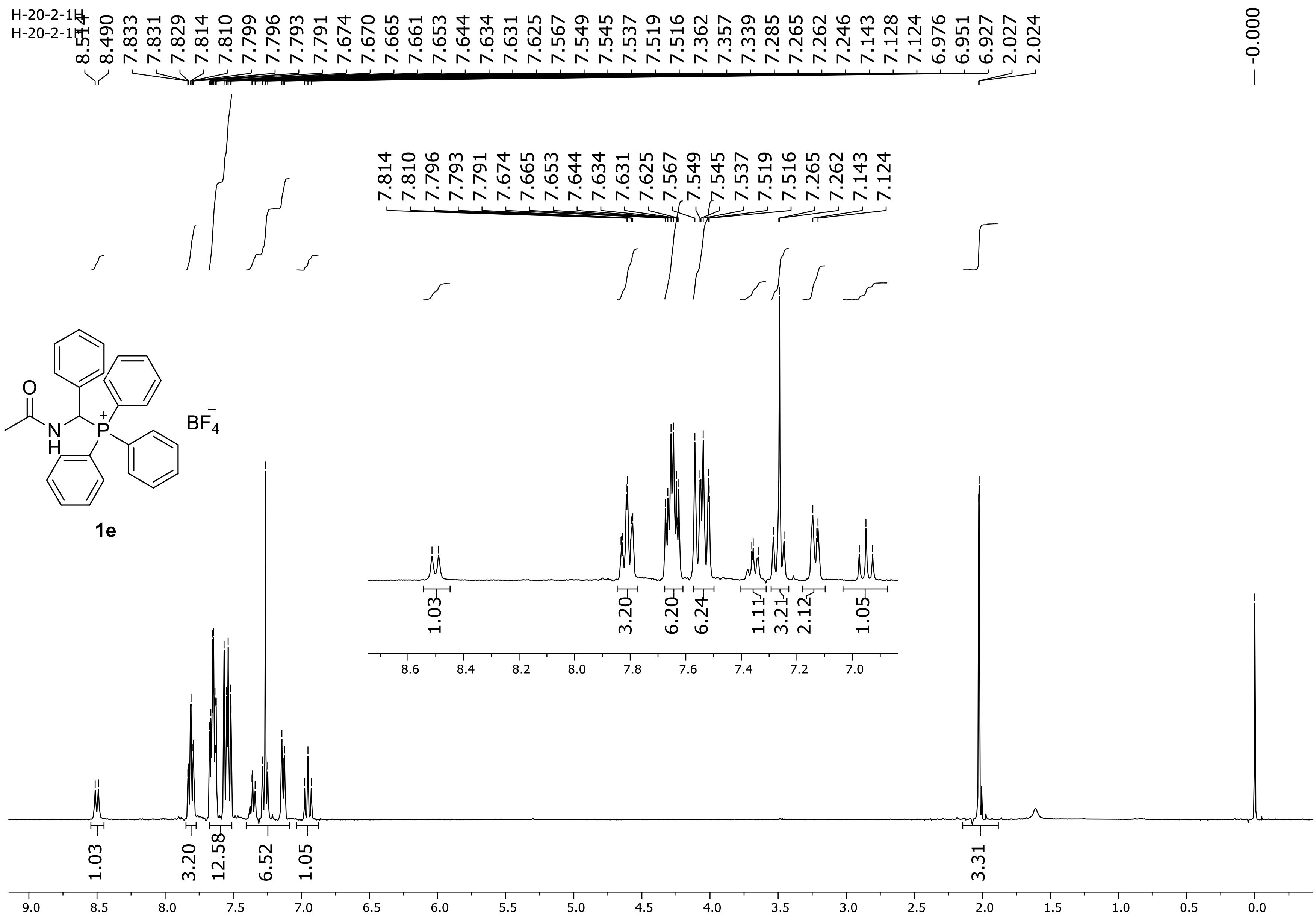

^{1}H NMR spectrum of (*N*-acetylamino)phenylmethyltriphenylphosphonium tetrafluoroborate (**1e**); 400 MHz/$CDCl_3$/TMS; δ (ppm).

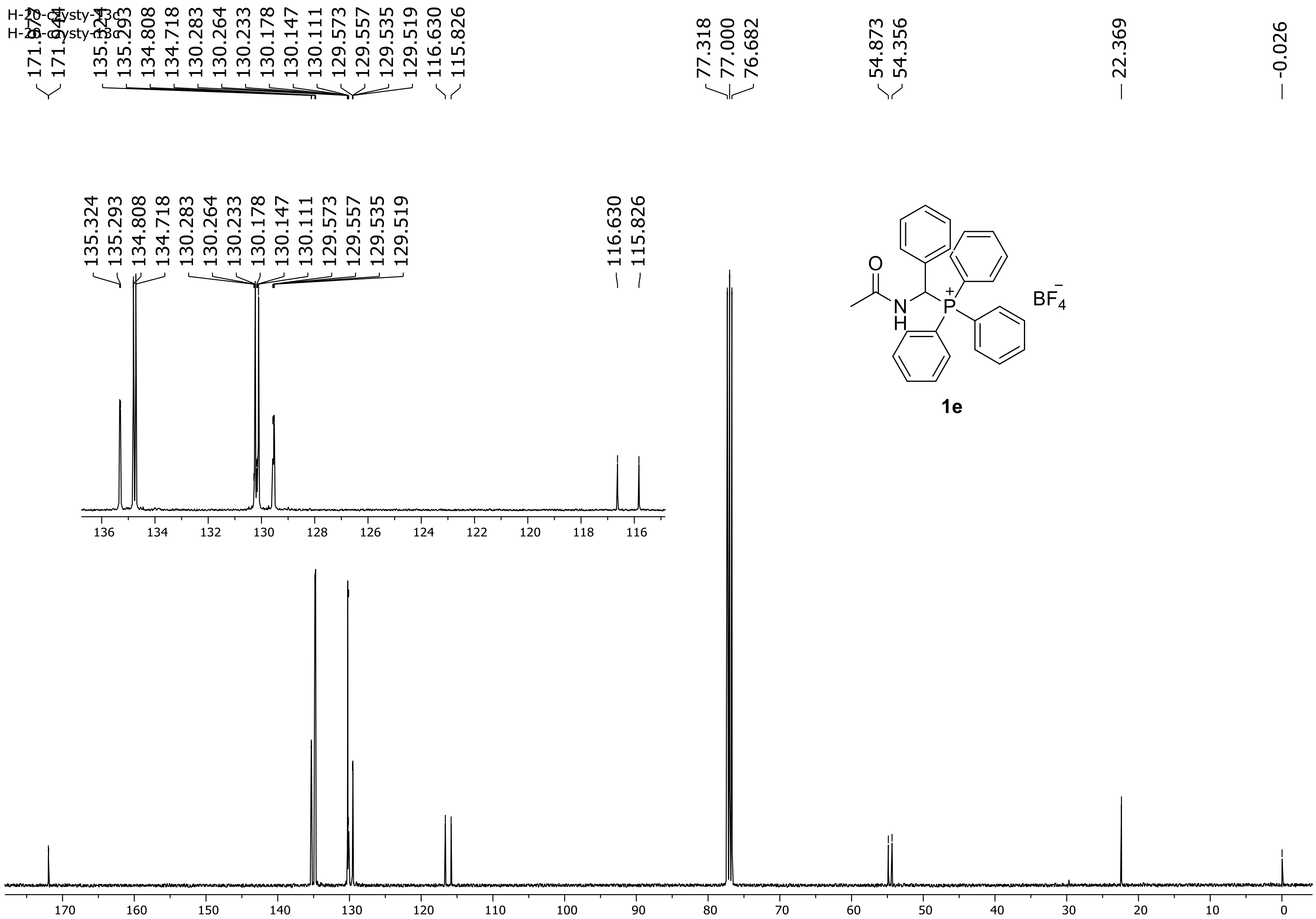

$^{13}C\{^{1}H\}$ NMR spectrum of (*N*-acetylamino)phenylmethyltriphenylphosphonium tetrafluoroborate (**1e**); 100 MHz/$CDCl_3$/TMS; δ (ppm).

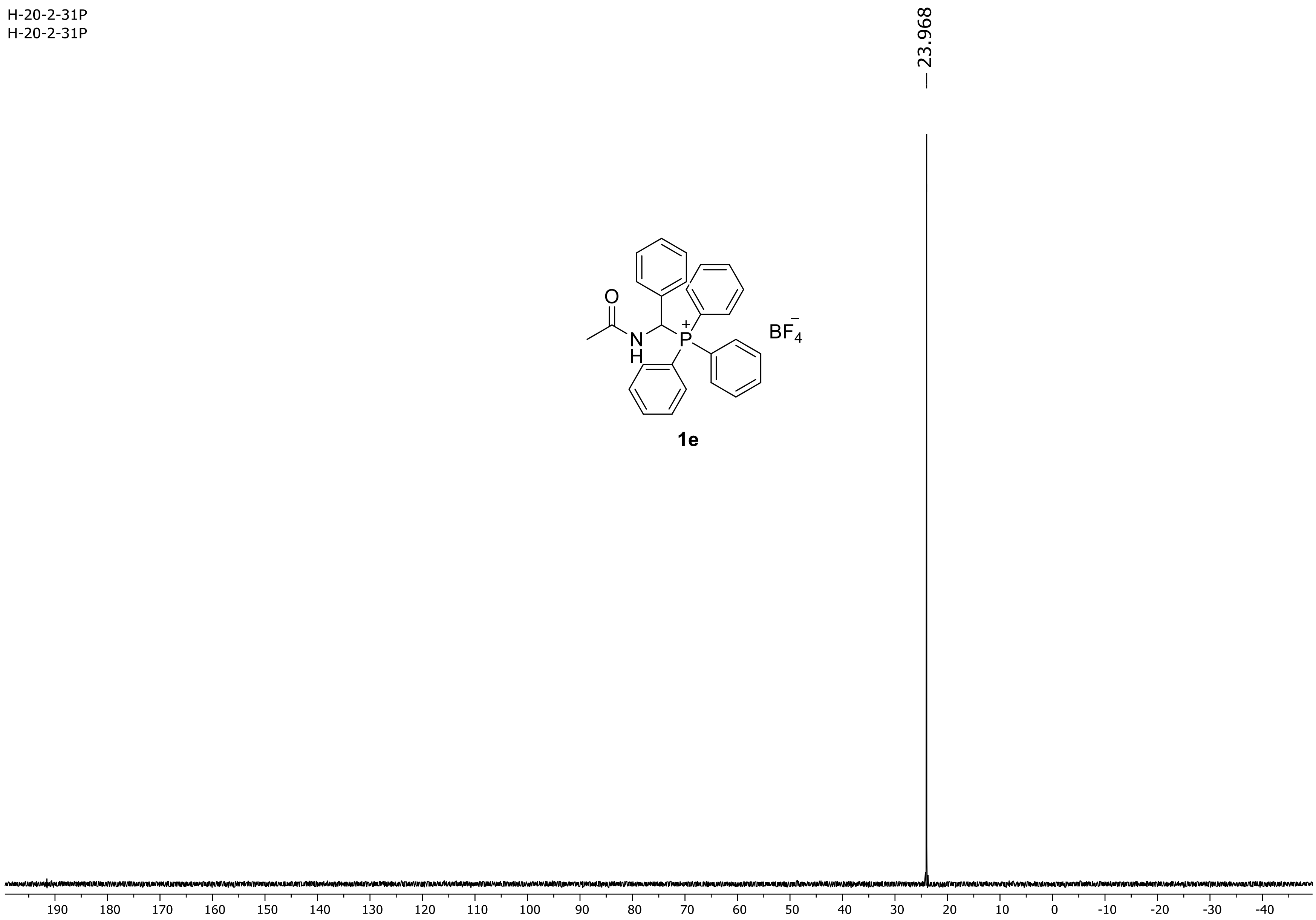

^{31}P NMR spectrum of (*N*-acetylamino)phenylmethyltriphenylphosphonium tetrafluoroborate (**1e**); 161.9 MHz/$CDCl_3$; δ (ppm).

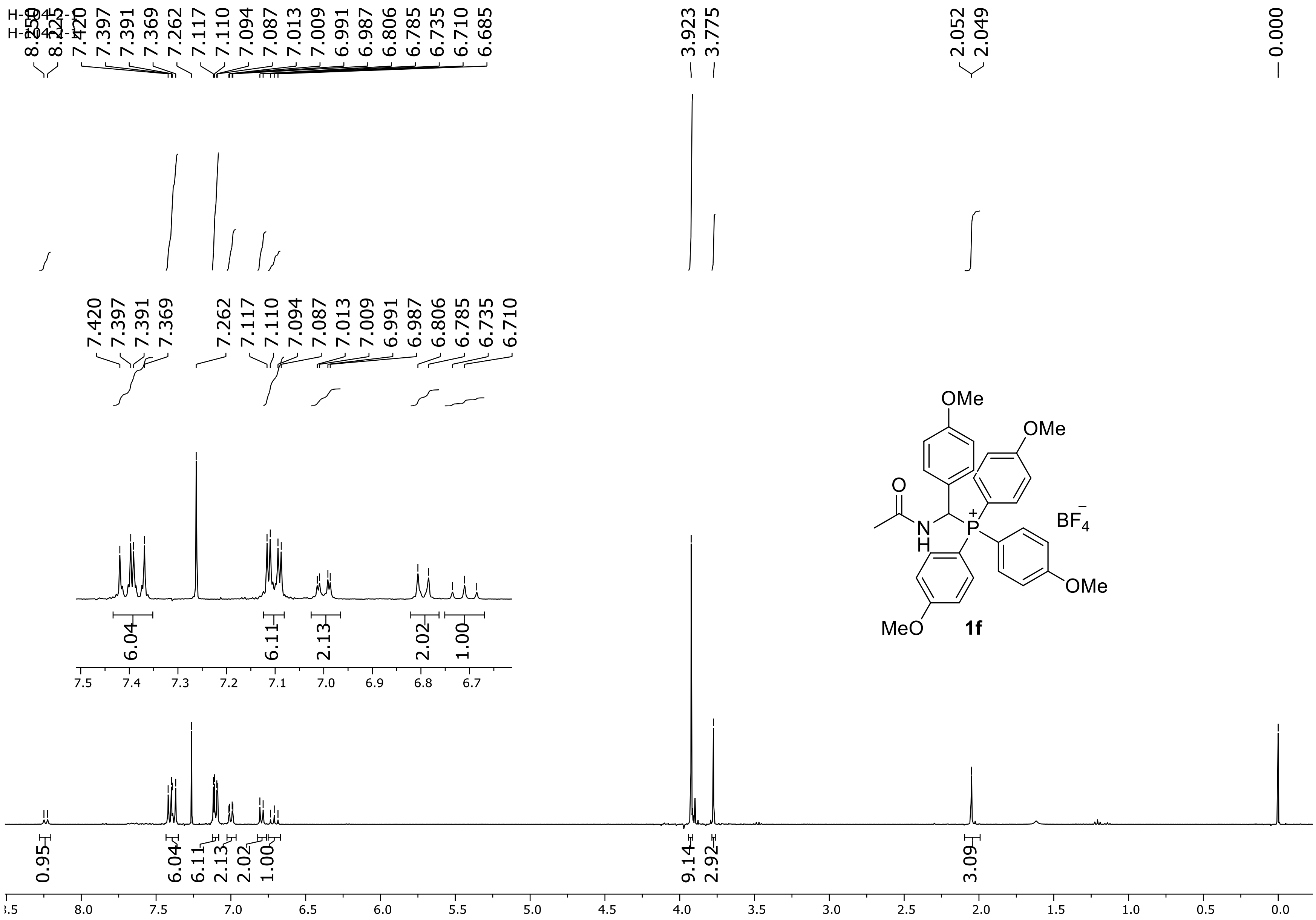

^{1}H NMR spectrum of 1-(*N*-acetylamino)-1-(4-metoxyphenyl)methyltris(4-metoxyphenyl)phosphonium tetrafluoroborate (**1f**); 400 MHz/$CDCl_3$/TMS; δ (ppm).

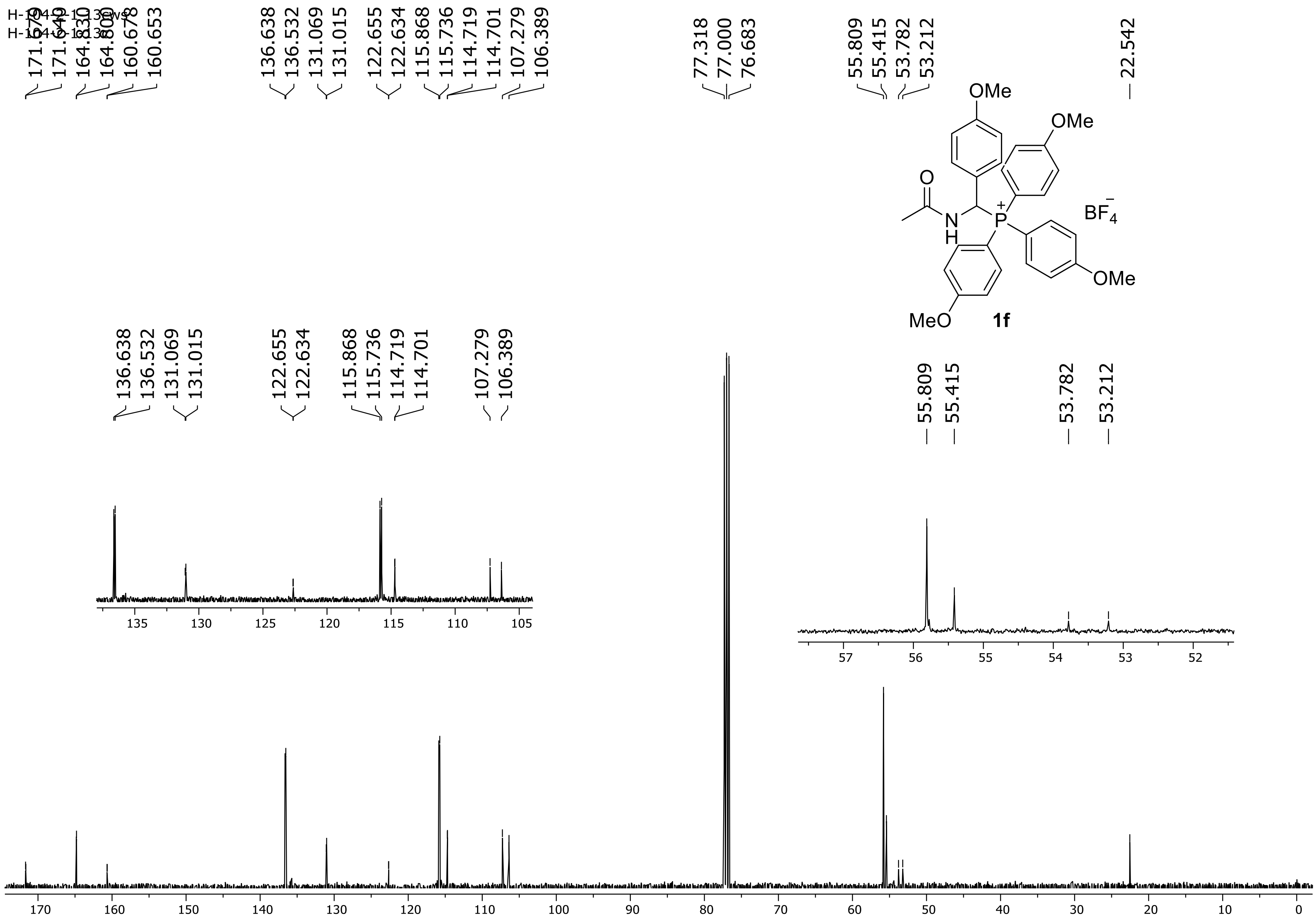

$^{13}C\{^{1}H\}$ NMR spectrum of 1-(*N*-acetylamino)-1-(4-metoxyphenyl)methyltris(4-metoxyphenyl)phosphonium tetrafluoroborate (**1f**); 100 MHz/$CDCl_3$/TMS; δ (ppm).

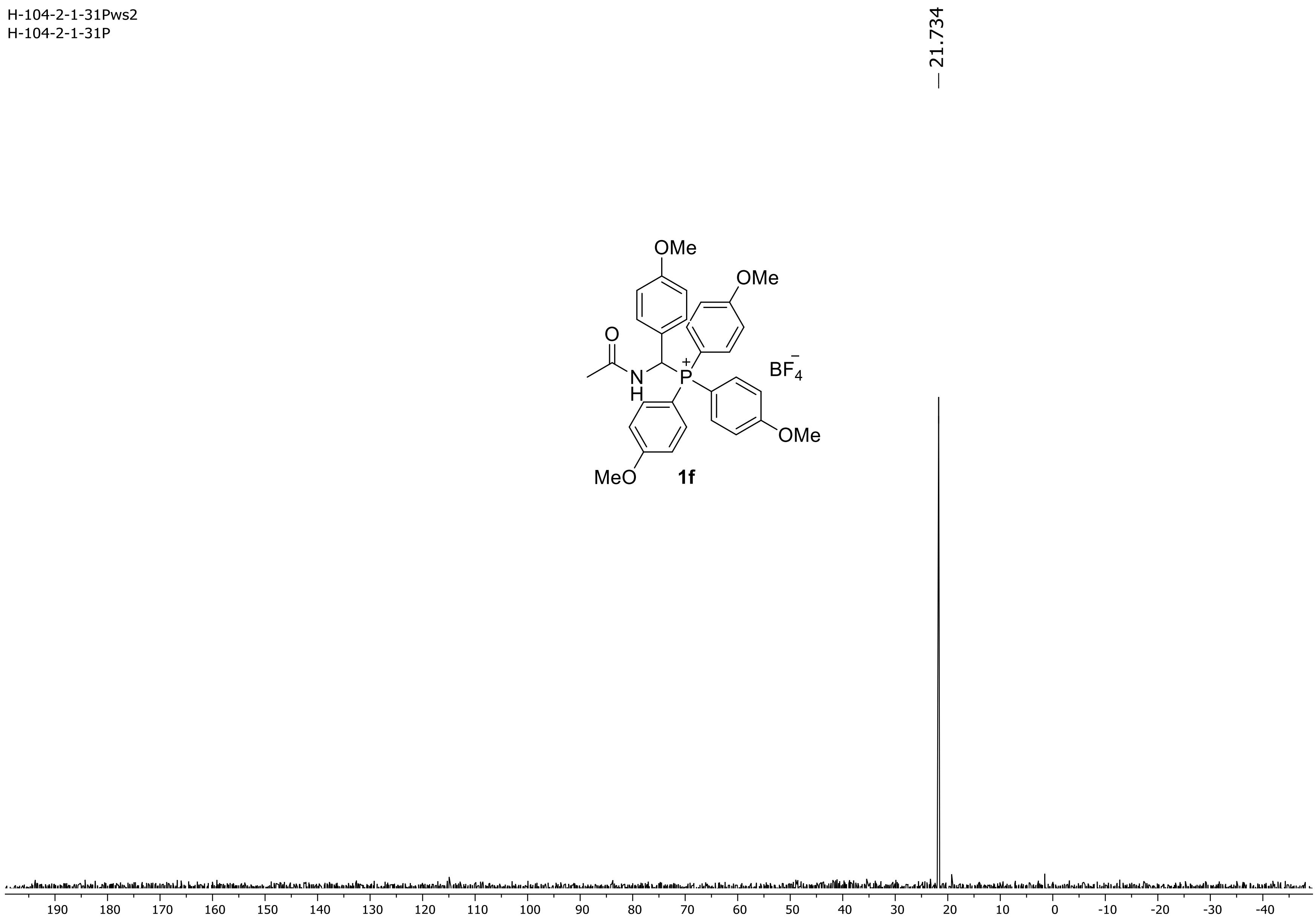

^{31}P NMR spectrum of 1-(*N*-acetylamino)-1-(4-metoxyphenyl)methyltris(4-metoxyphenyl)phosphonium tetrafluoroborate (**1f**); 161.9 MHz/$CDCl_3$; δ (ppm).

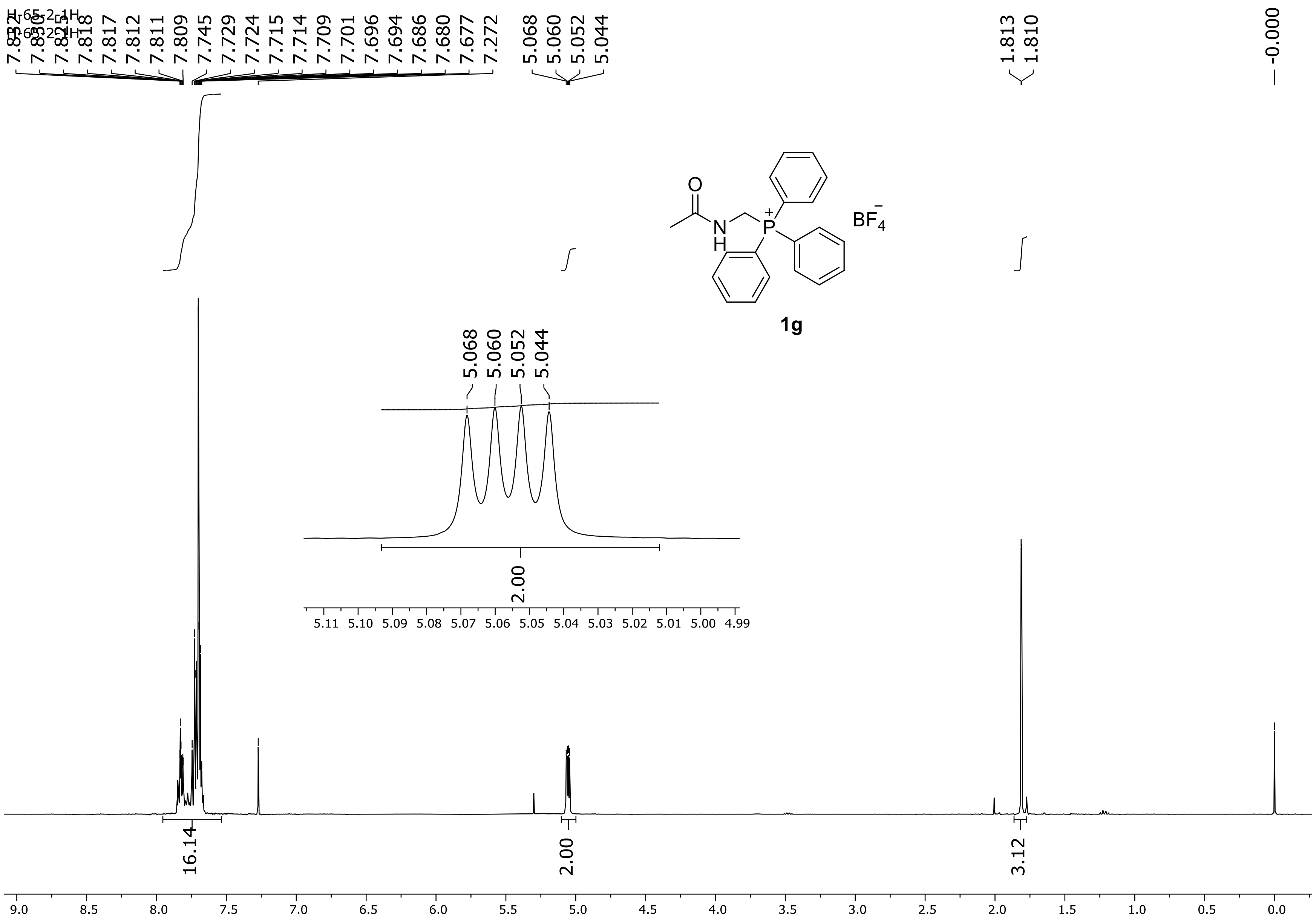

^{1}H NMR spectrum of (*N*-acetylamino)methyltriphenylphosphonium tetrafluoroborate (**1g**); 400 MHz/$CDCl_3$/TMS; δ (ppm).

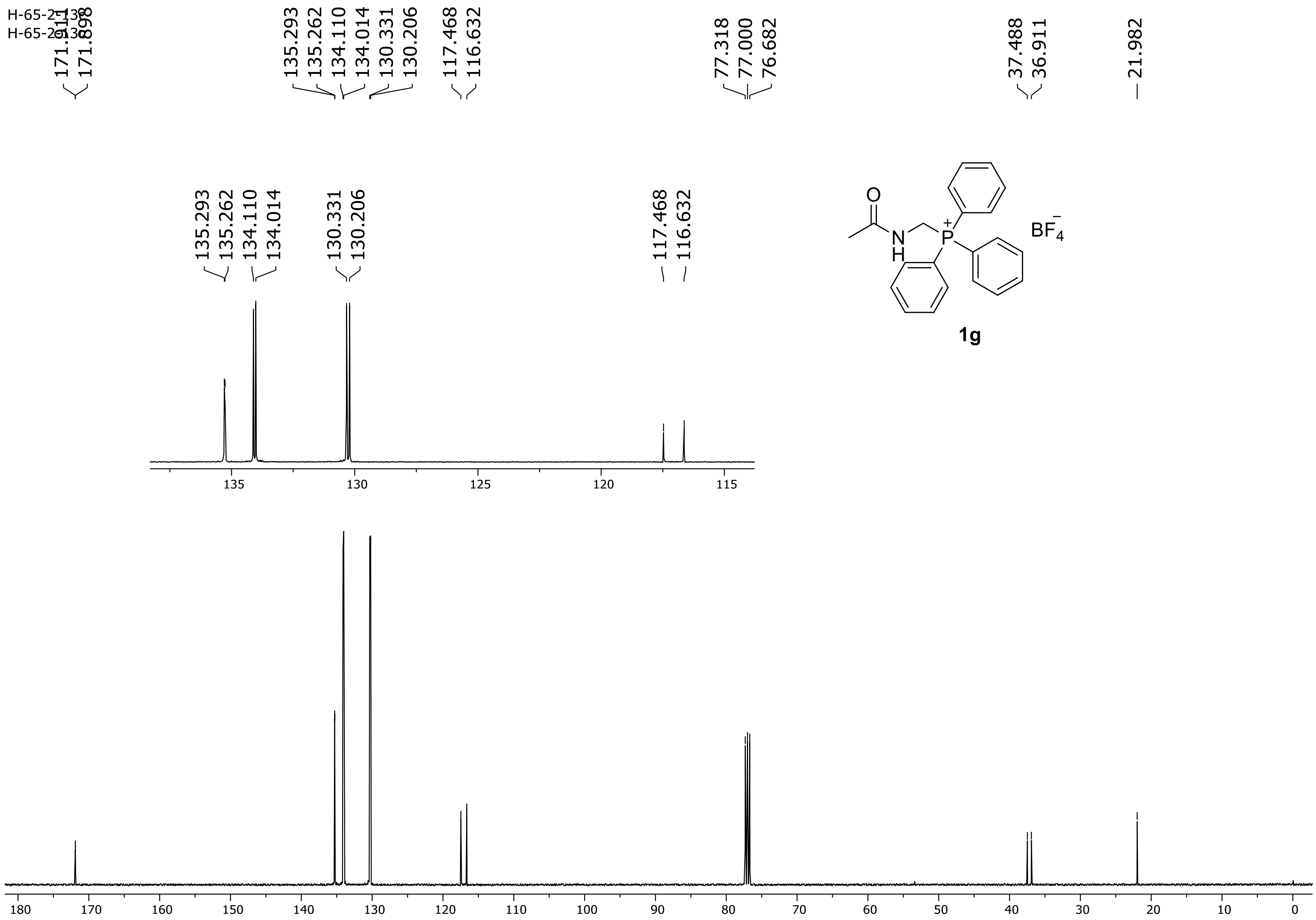

$^{13}C\{^1H\}$ NMR spectrum of (*N*-acetylamino)methyltriphenylphosphonium tetrafluoroborate (**1g**); 100 MHz/$CDCl_3$/TMS; δ (ppm).

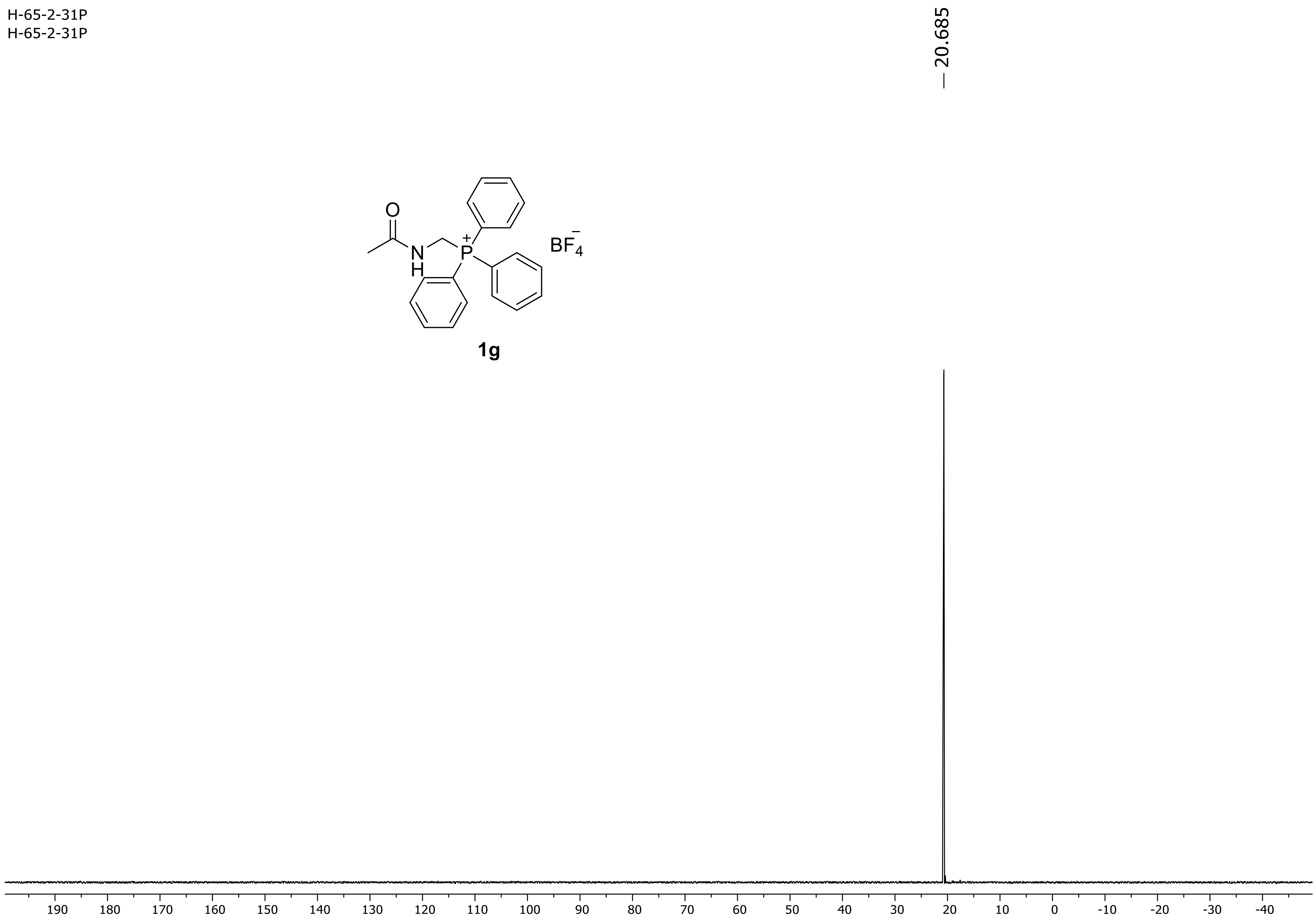

^{31}P NMR spectrum of (*N*-acetylamino)methyltriphenylphosphonium tetrafluoroborate (**1g**); 161.9 MHz/$CDCl_3$; δ (ppm).

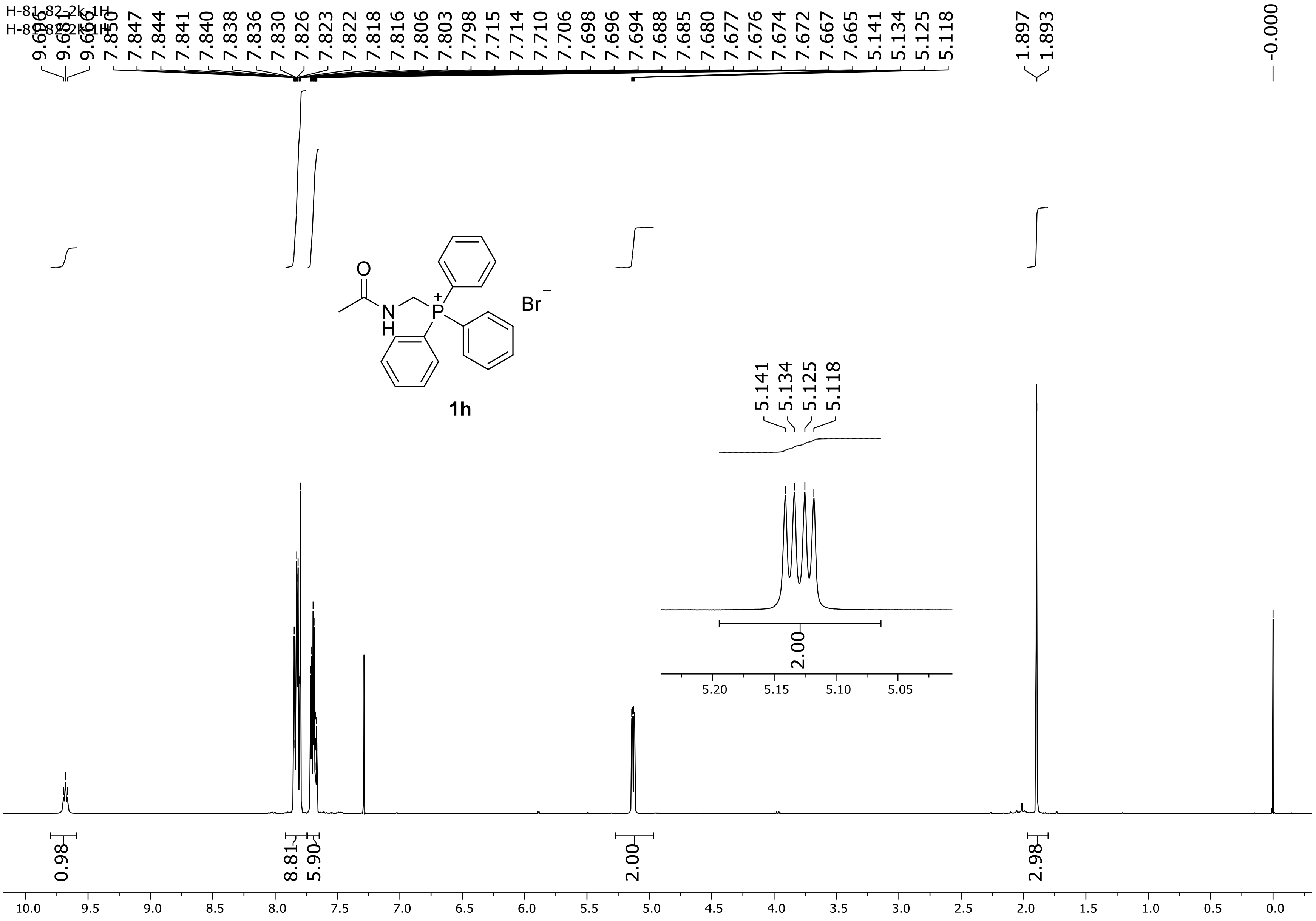

^{1}H NMR spectrum of (*N*-acetylamino)methyltriphenylphosphonium bromide (**1h**); 400 MHz/$CDCl_3$/TMS; δ (ppm).

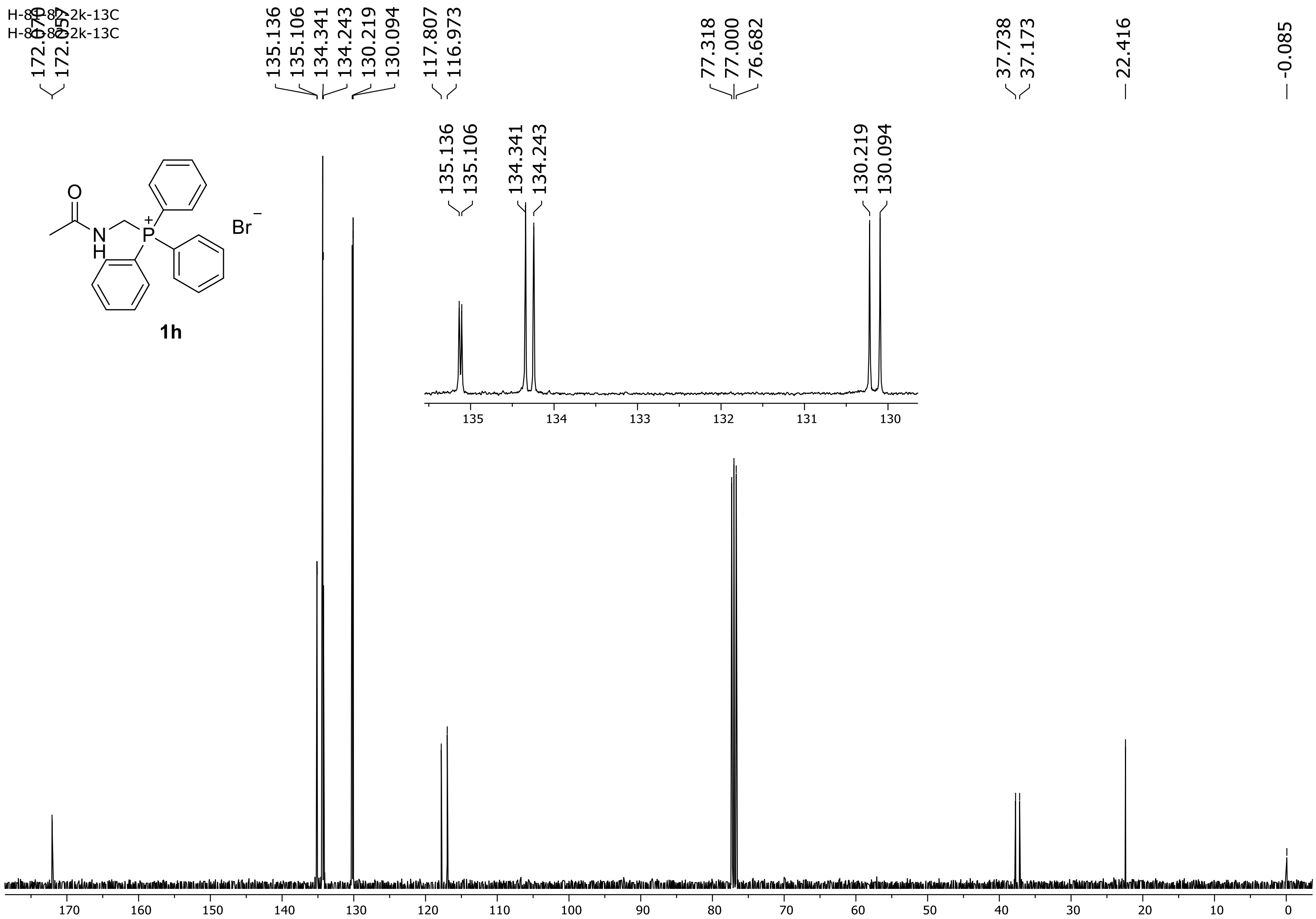

$^{13}C\{^{1}H\}$ NMR spectrum of (*N*-acetylamino)methyltriphenylphosphonium bromide (**1h**); 100 MHz/$CDCl_3$/TMS; δ (ppm).

H-81-31P
H-81-31P

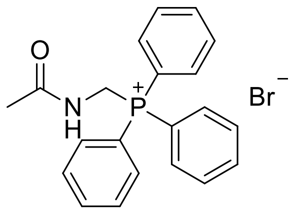

1h

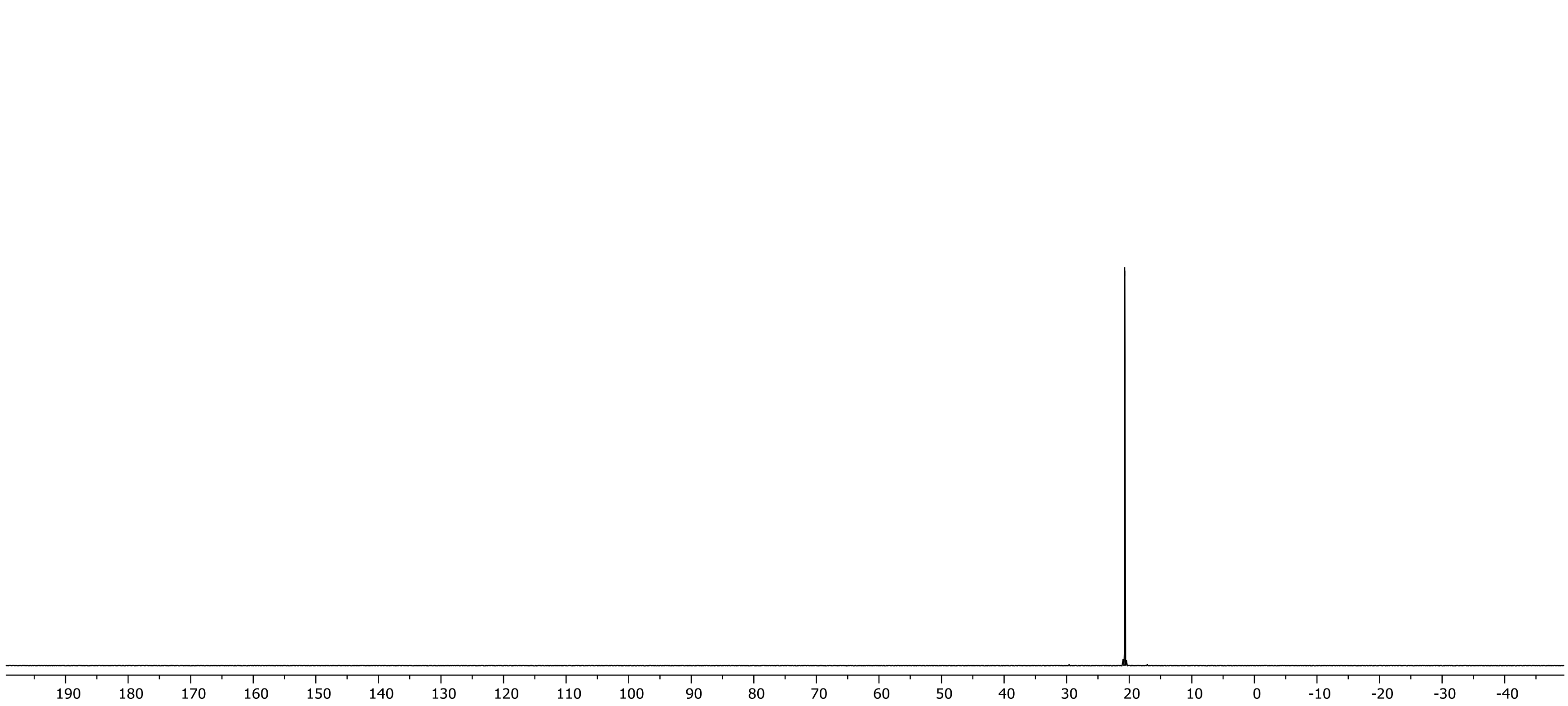

^{31}P NMR spectrum of (*N*-acetylamino)methyltriphenylphosphonium bromide (**1h**); 161.9 MHz/$CDCl_3$; δ (ppm).

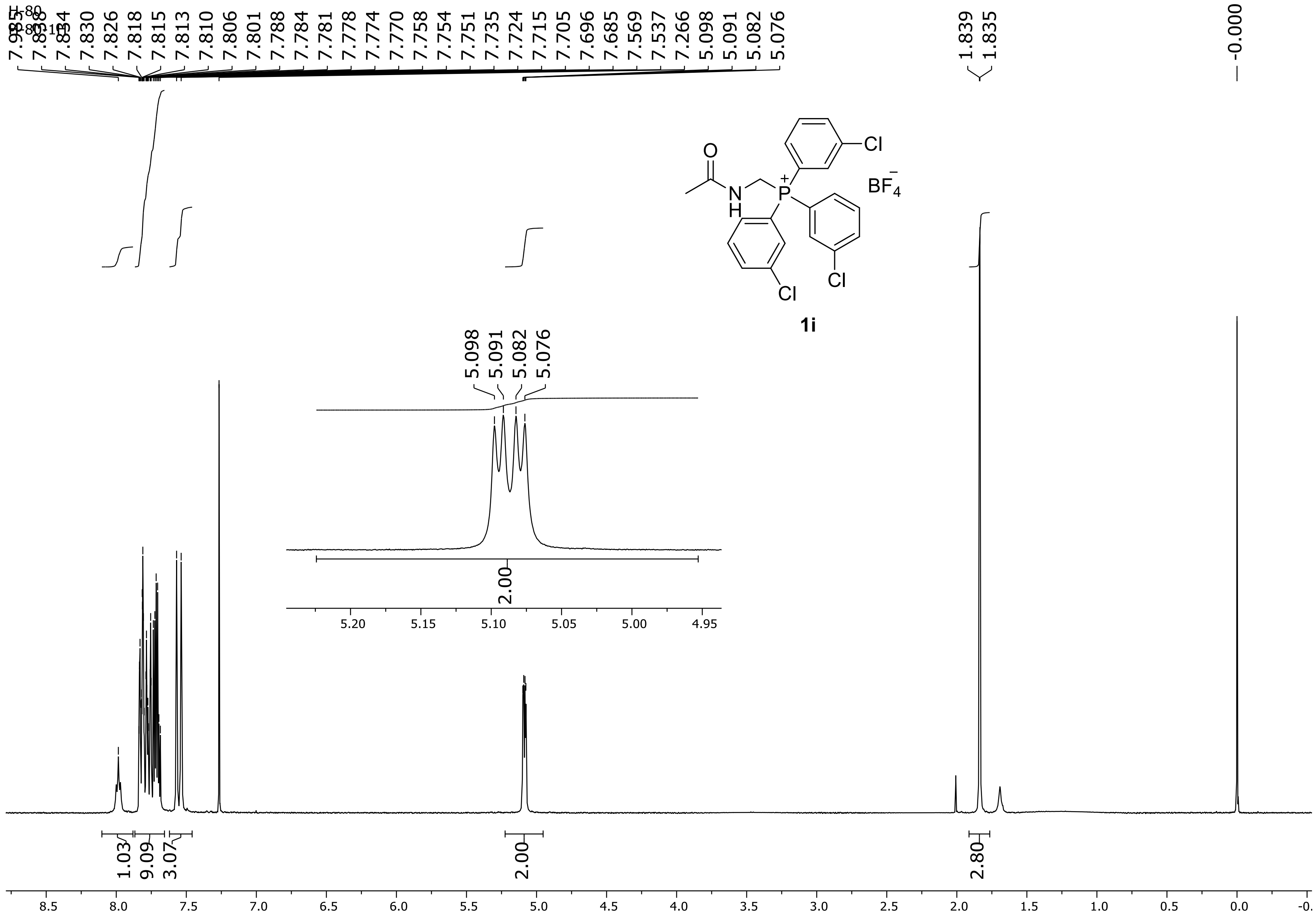

^{1}H NMR spectrum of (*N*-acetylamino)methyltris(3-chlorophenyl)phosphonium tetrafluoroborate (**1i**); 400 MHz/$CDCl_3$/TMS; δ (ppm).

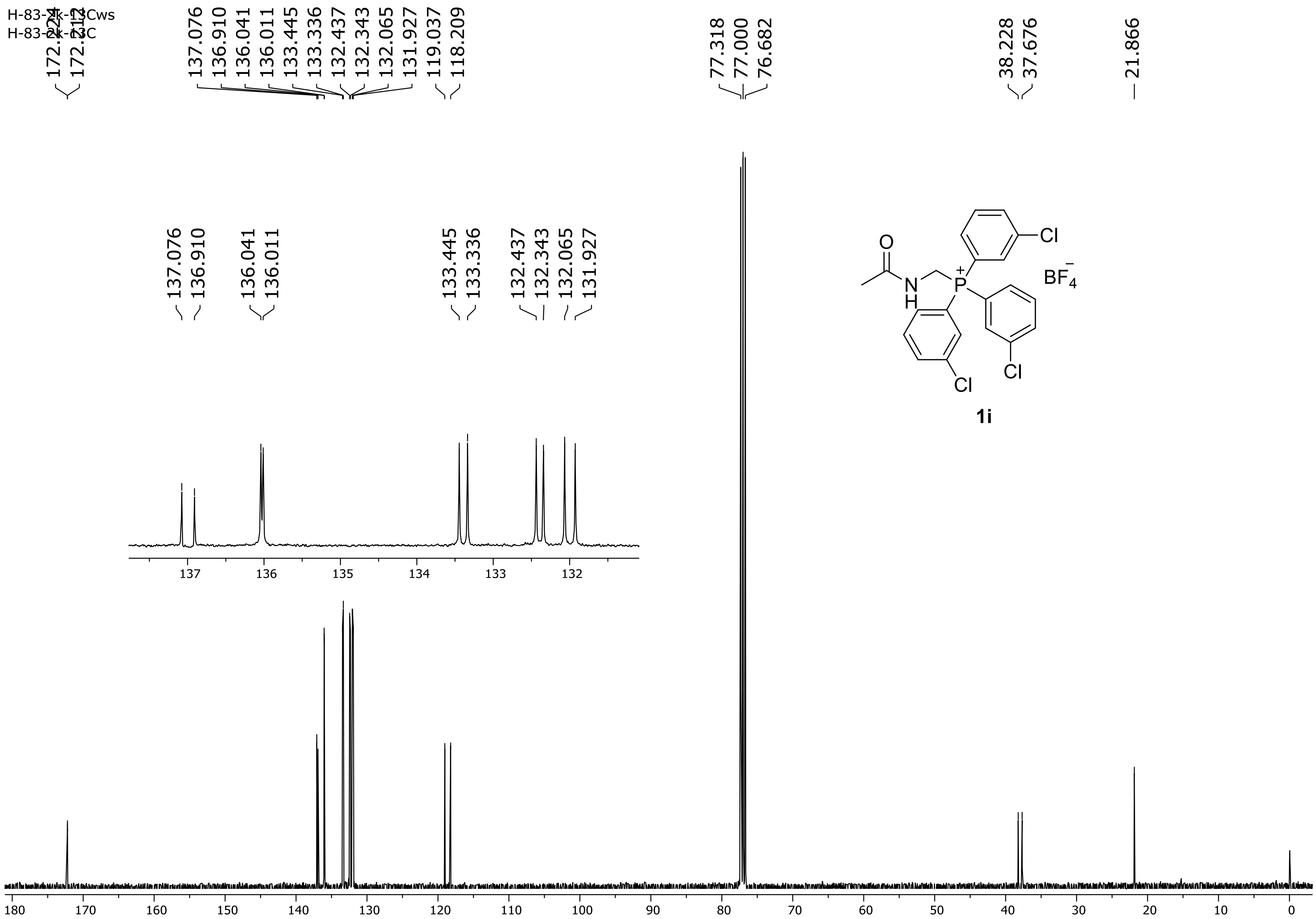

$^{13}C\{^1H\}$ NMR spectrum of (*N*-acetylamino)methyltris(3-chlorophenyl)phosphonium tetrafluoroborate (**1i**); 100 MHz/$CDCl_3$/TMS; δ (ppm).

H-80-31P
H-80-31P

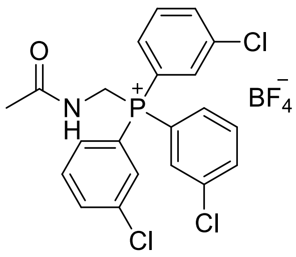

1i

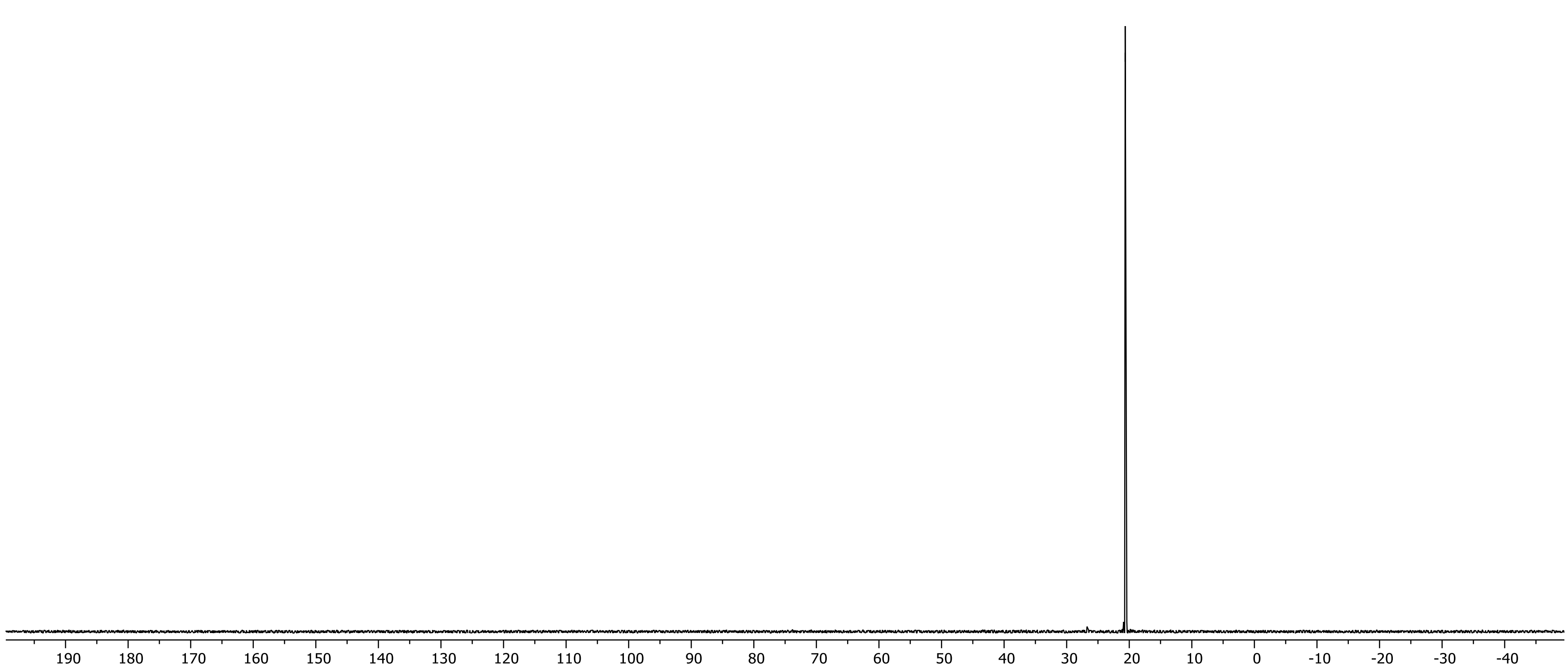

^{31}P NMR spectrum of (*N*-acetylamino)methyltris(3-chlorophenyl)phosphonium tetrafluoroborate (**1i**); 161.9 MHz/$CDCl_3$; δ (ppm).

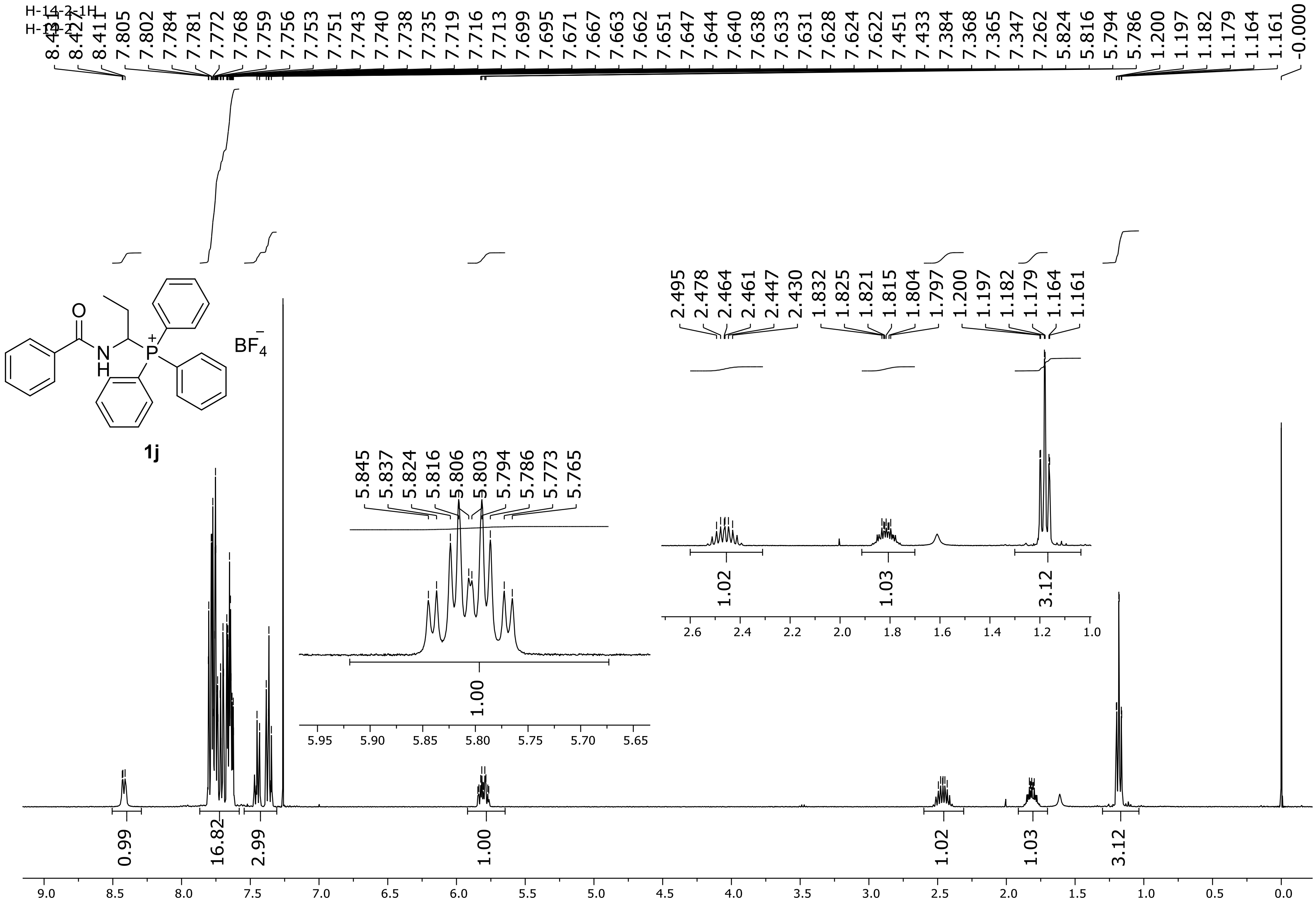

^{1}H NMR spectrum of 1-(*N*-benzoylamino)propyltriphenylphosphonium tetrafluoroborate (**1j**); 400 MHz/$CDCl_3$/TMS; δ (ppm).

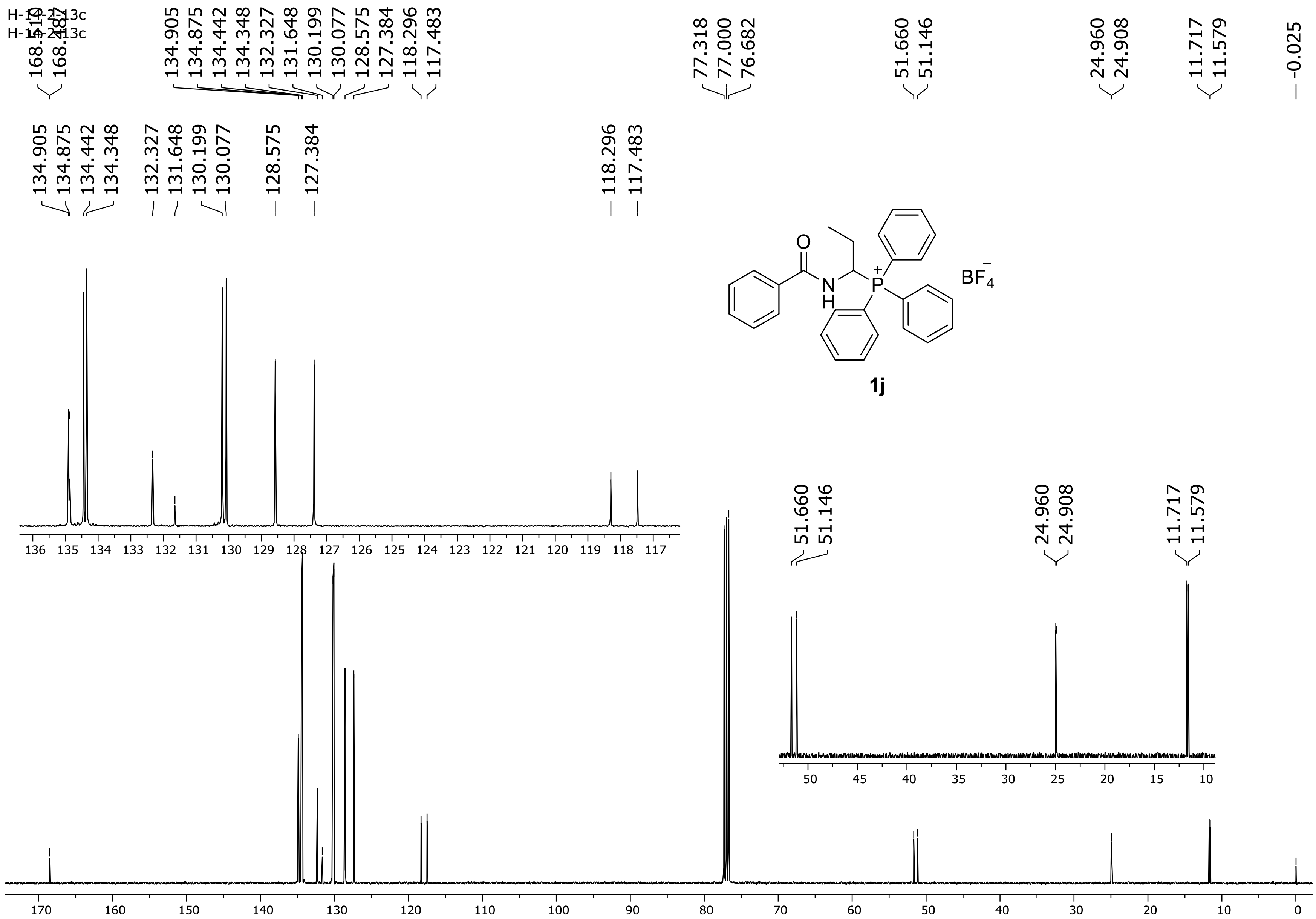

$^{13}C\{^{1}H\}$ NMR spectrum of 1-(*N*-benzoylamino)propyltriphenylphosphonium tetrafluoroborate (**1j**); 100 MHz/$CDCl_3$/TMS; δ (ppm).

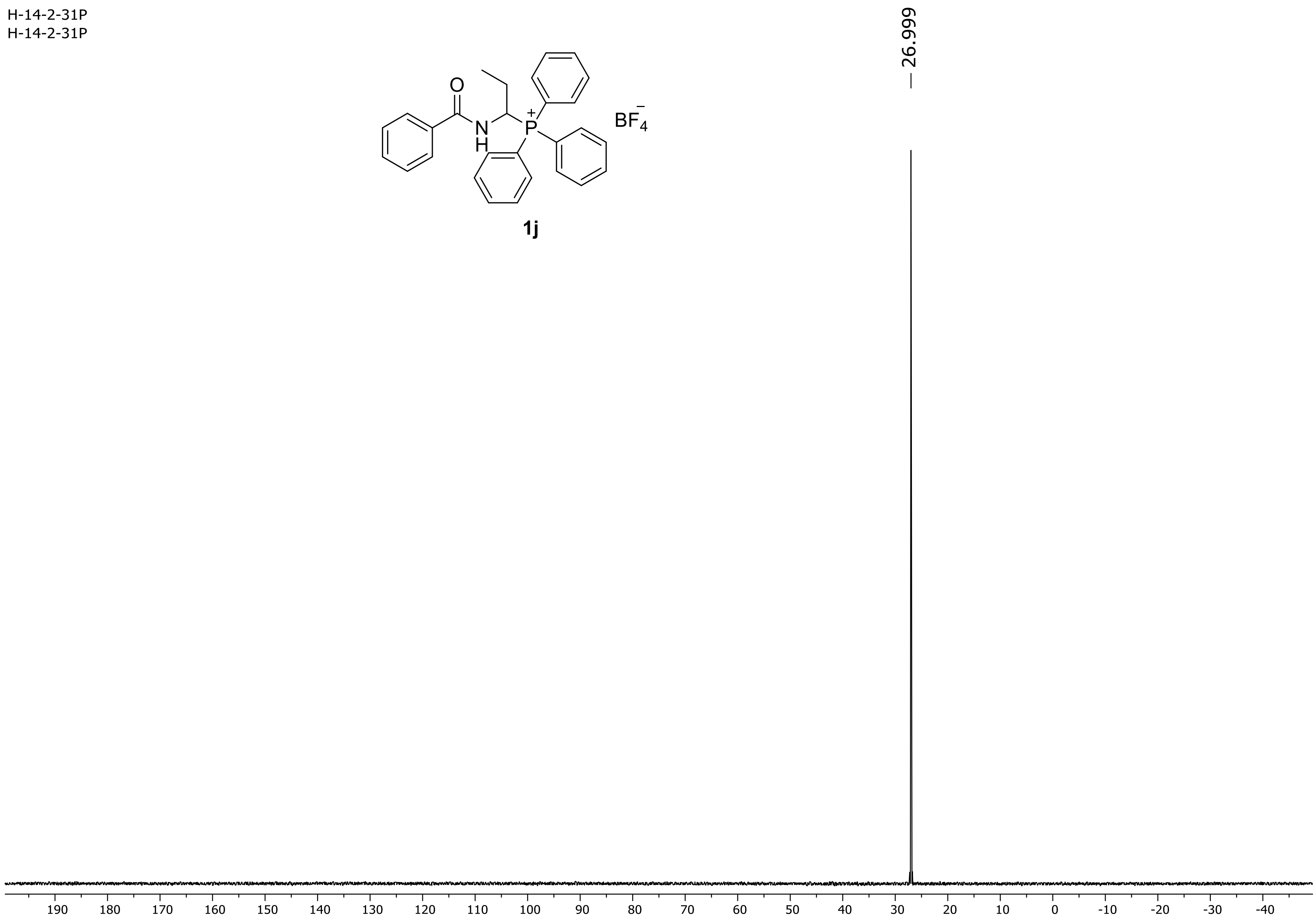

^{31}P NMR spectrum of 1-(*N*-benzoylamino)propyltriphenylphosphonium tetrafluoroborate (**1j**); 161.9 MHz/$CDCl_3$; δ (ppm).

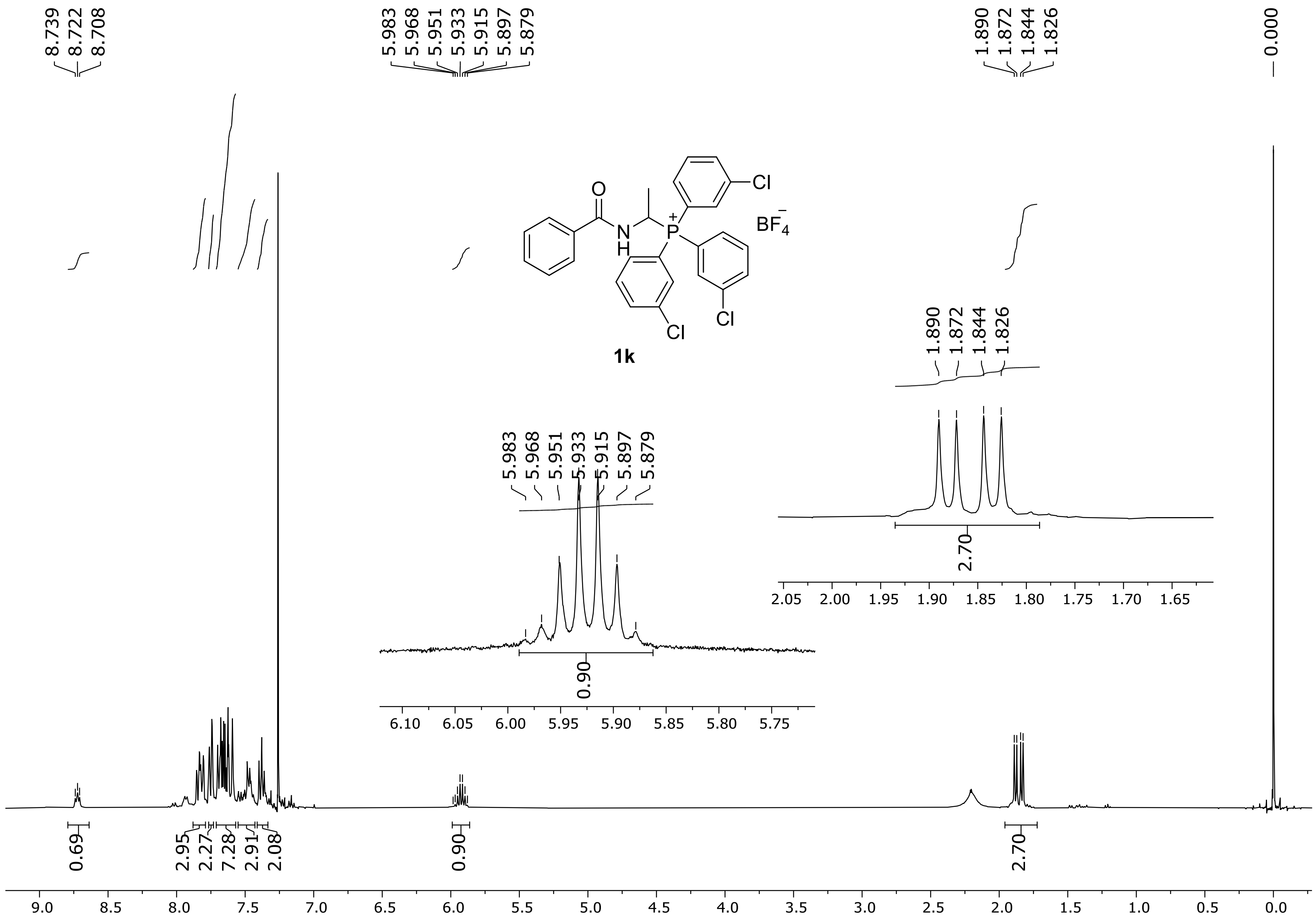

^{1}H NMR spectrum of 1-(*N*-benzoylamino)ethyltris(3-chlorophenyl)phosphonium tetrafluoroborate (**1k**); 400 MHz/$CDCl_3$/TMS; δ (ppm).

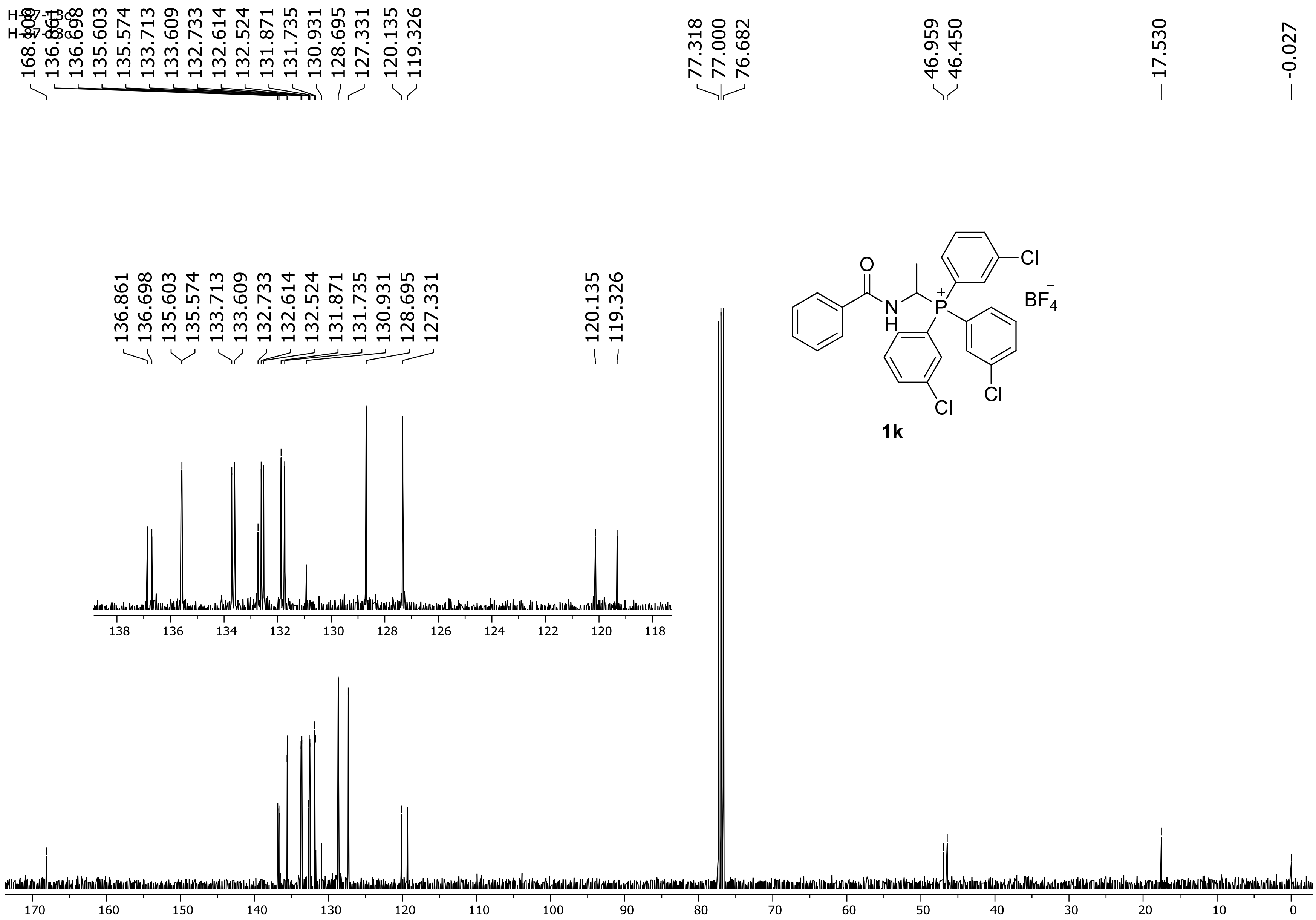

$^{13}C\{^{1}H\}$ NMR spectrum of 1-(*N*-benzoylamino)ethyltris(3-chlorophenyl)phosphonium tetrafluoroborate (**1k**); 100 MHz/$CDCl_3$/TMS; δ (ppm).

H-87-31P
H-87-31P

27.682

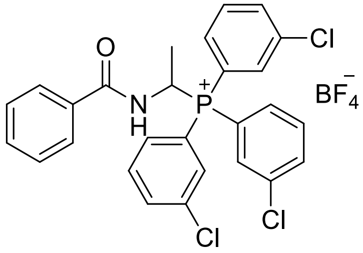

1k

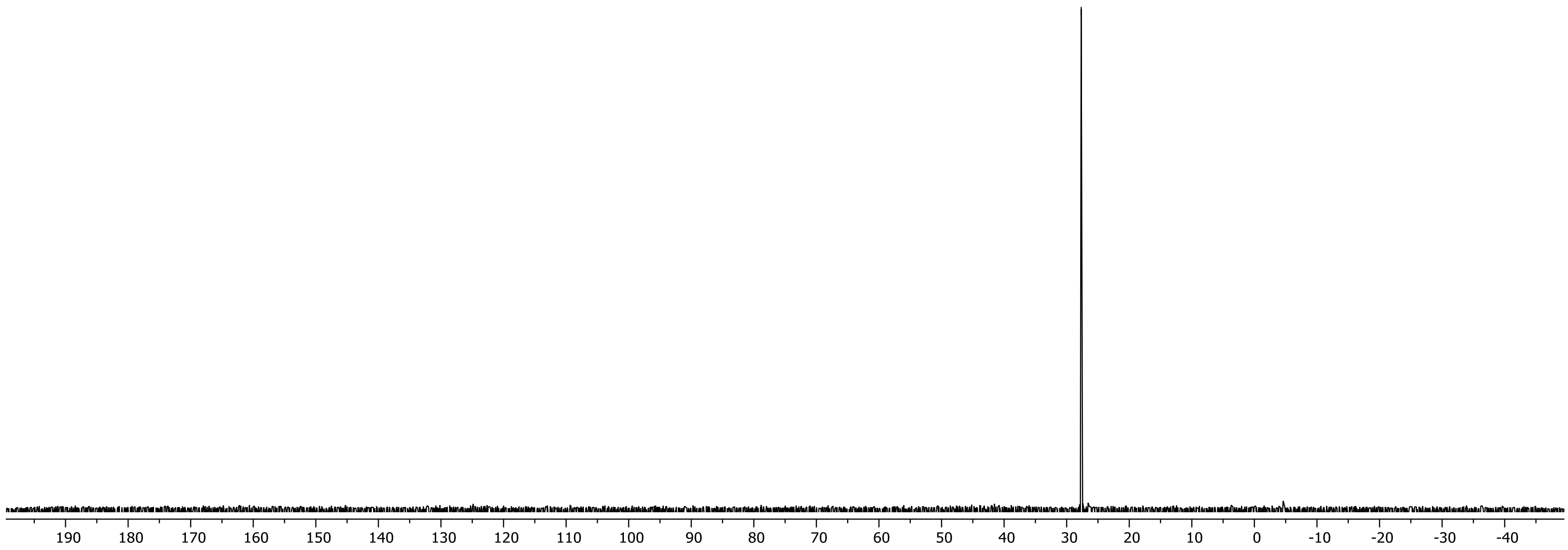

^{31}P NMR spectrum of 1-(*N*-benzoylamino)ethyltris(3-chlorophenyl)phosphonium tetrafluoroborate (**1k**); 161.9 MHz/$CDCl_3$; δ (ppm).

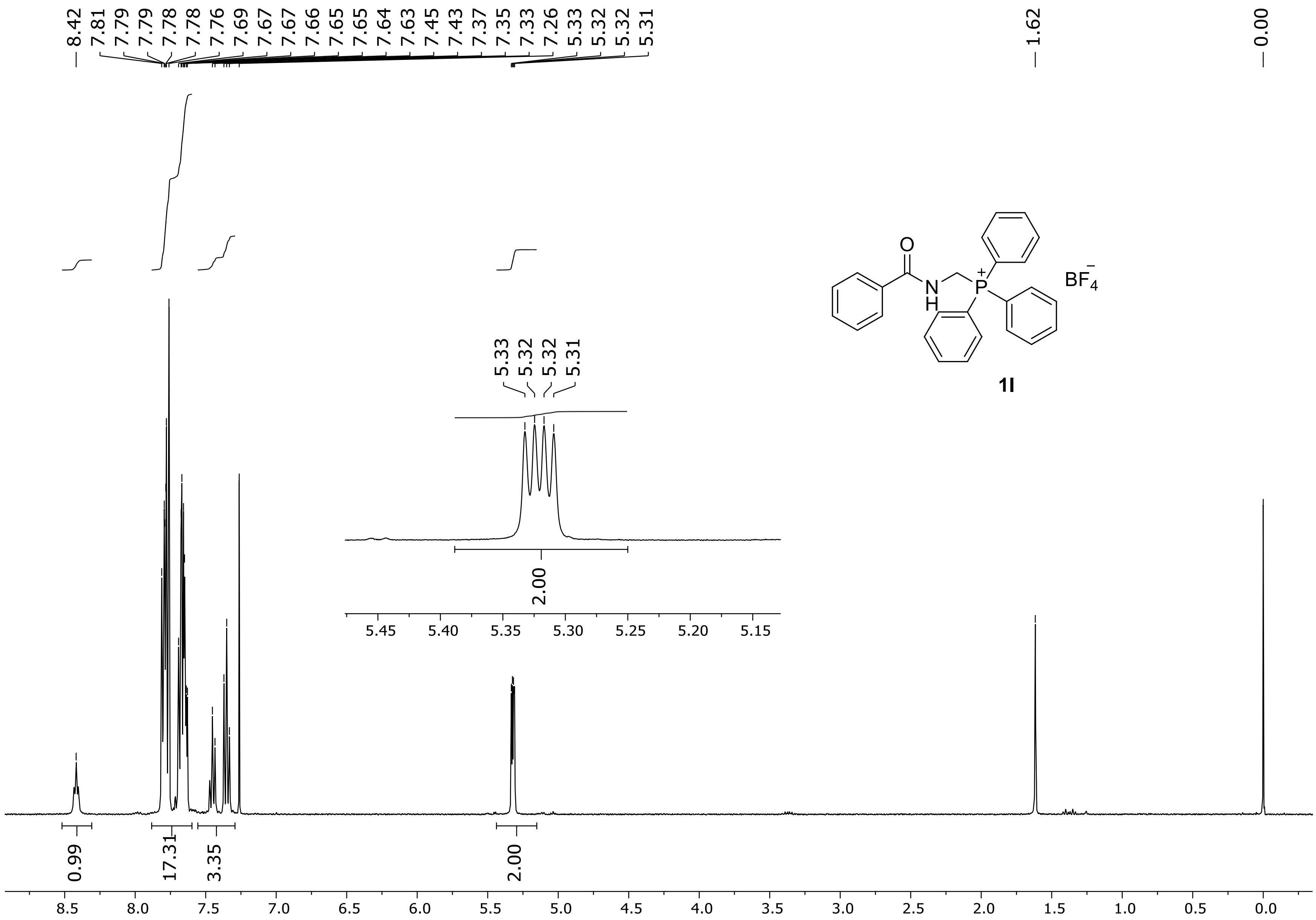

^{1}H NMR spectrum of (*N*-benzoylamino)methyltriphenylphosphonium tetrafluoroborate (**1l**); 400 MHz/$CDCl_3$/TMS; δ (ppm).

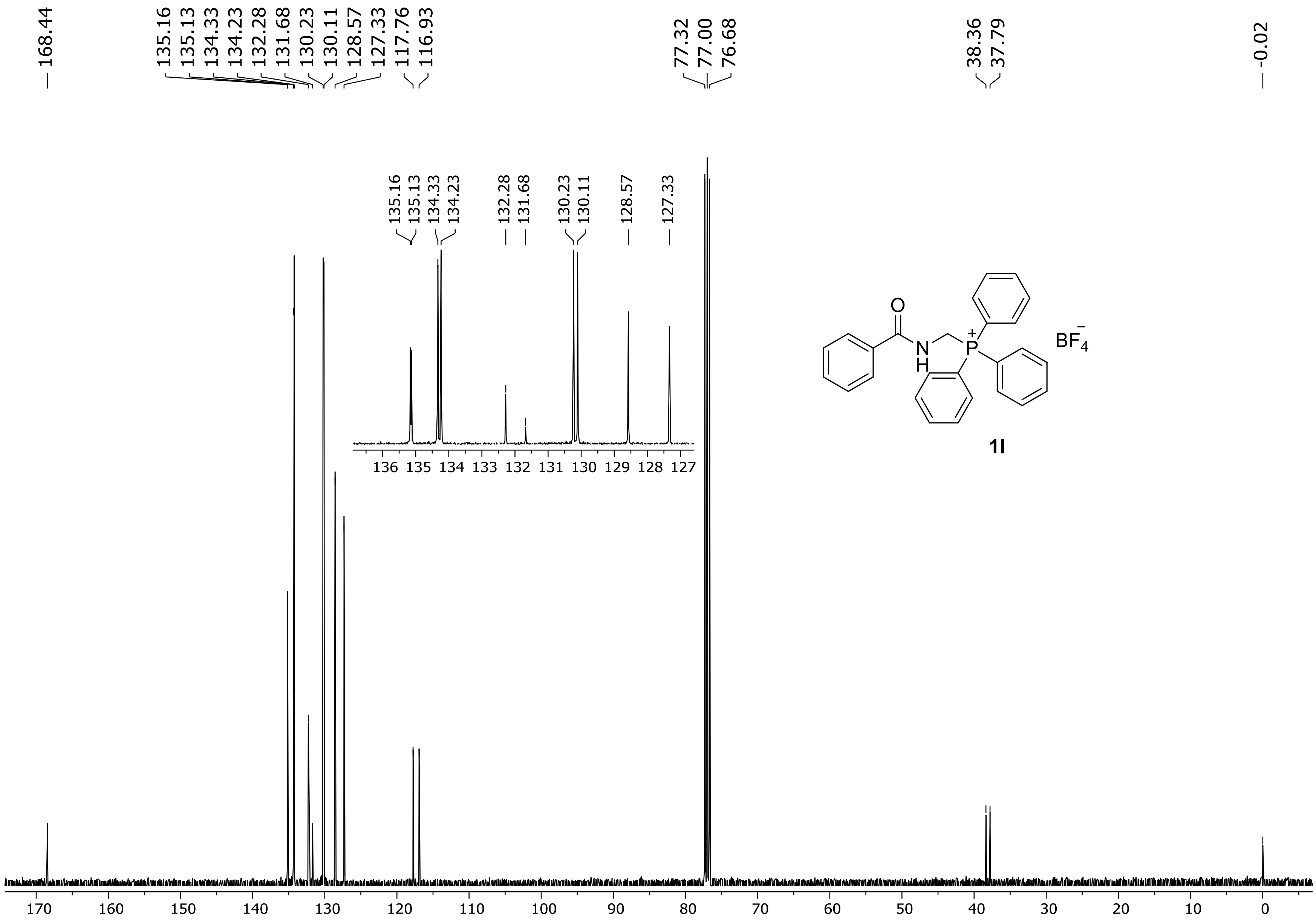

$^{13}C\{^{1}H\}$ NMR spectrum of (*N*-benzoylamino)methyltriphenylphosphonium tetrafluoroborate (**1l**); 100 MHz/$CDCl_3$/TMS; δ (ppm).

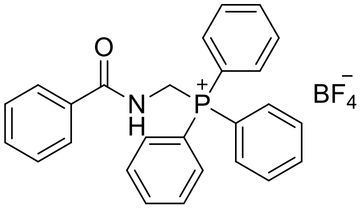

1l

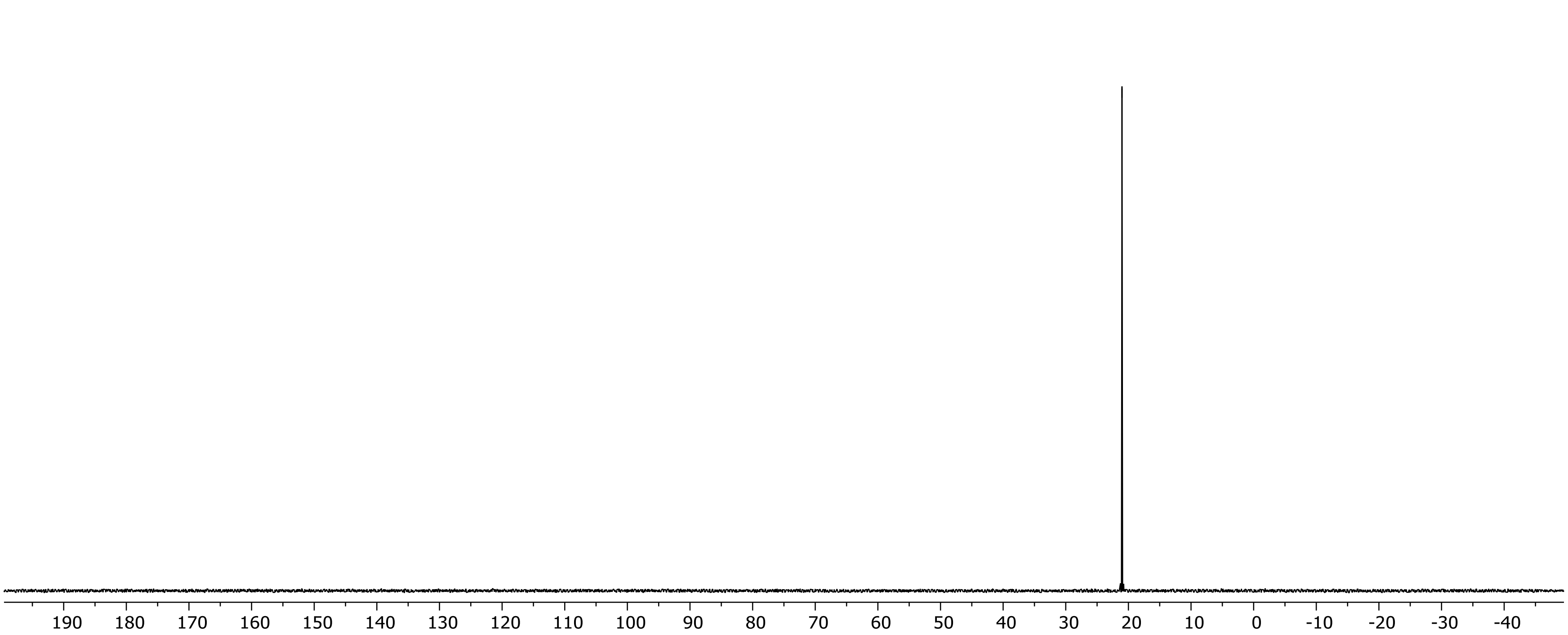

^{31}P NMR spectrum of (*N*-benzoylamino)methyltriphenylphosphonium tetrafluoroborate (**1l**); 161.9 MHz/$CDCl_3$; δ (ppm).

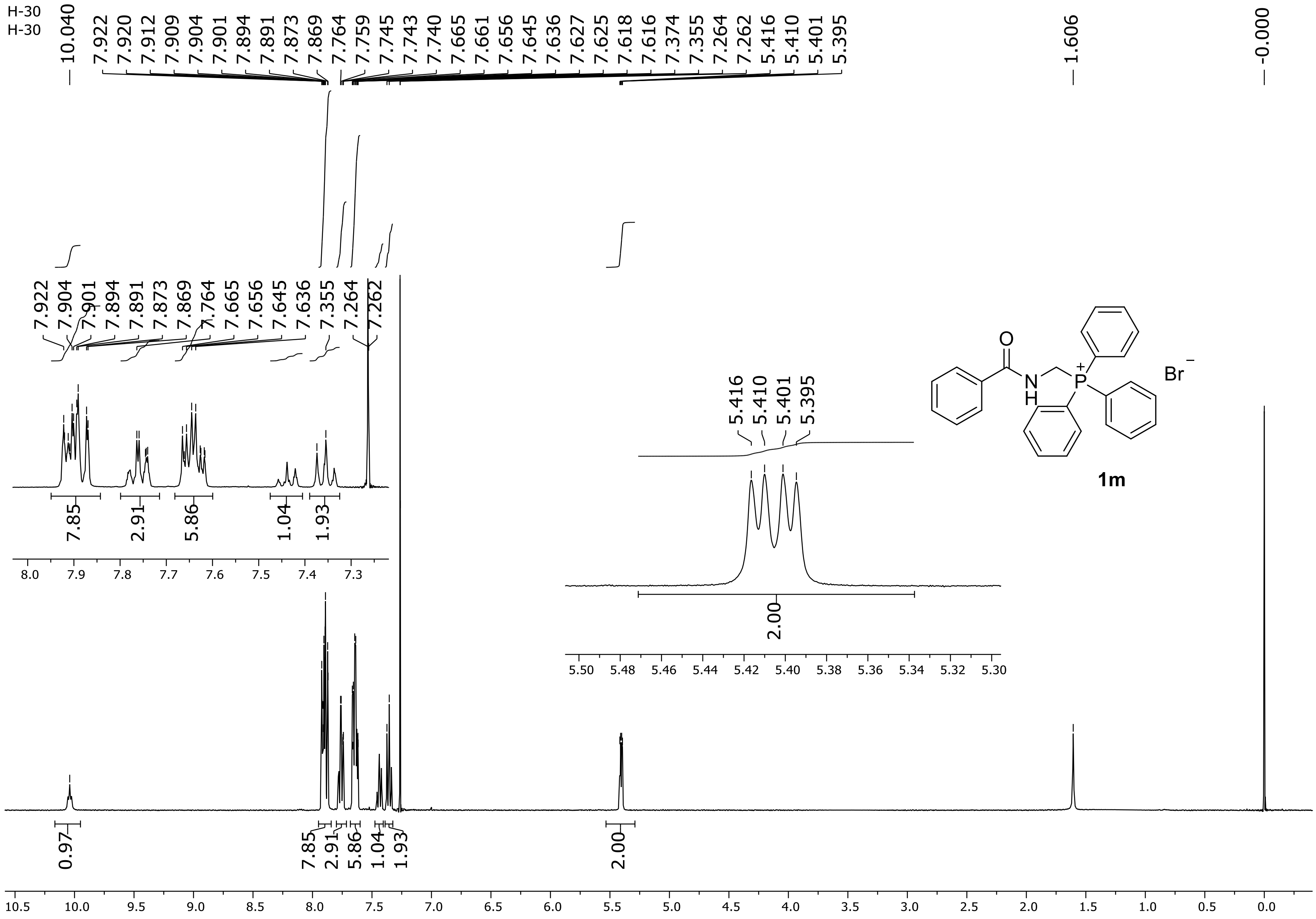

^{1}H NMR spectrum of (*N*-benzoylamino)methyltriphenylphosphonium bromide (**1m**); 400 MHz/$CDCl_3$/TMS; δ (ppm).

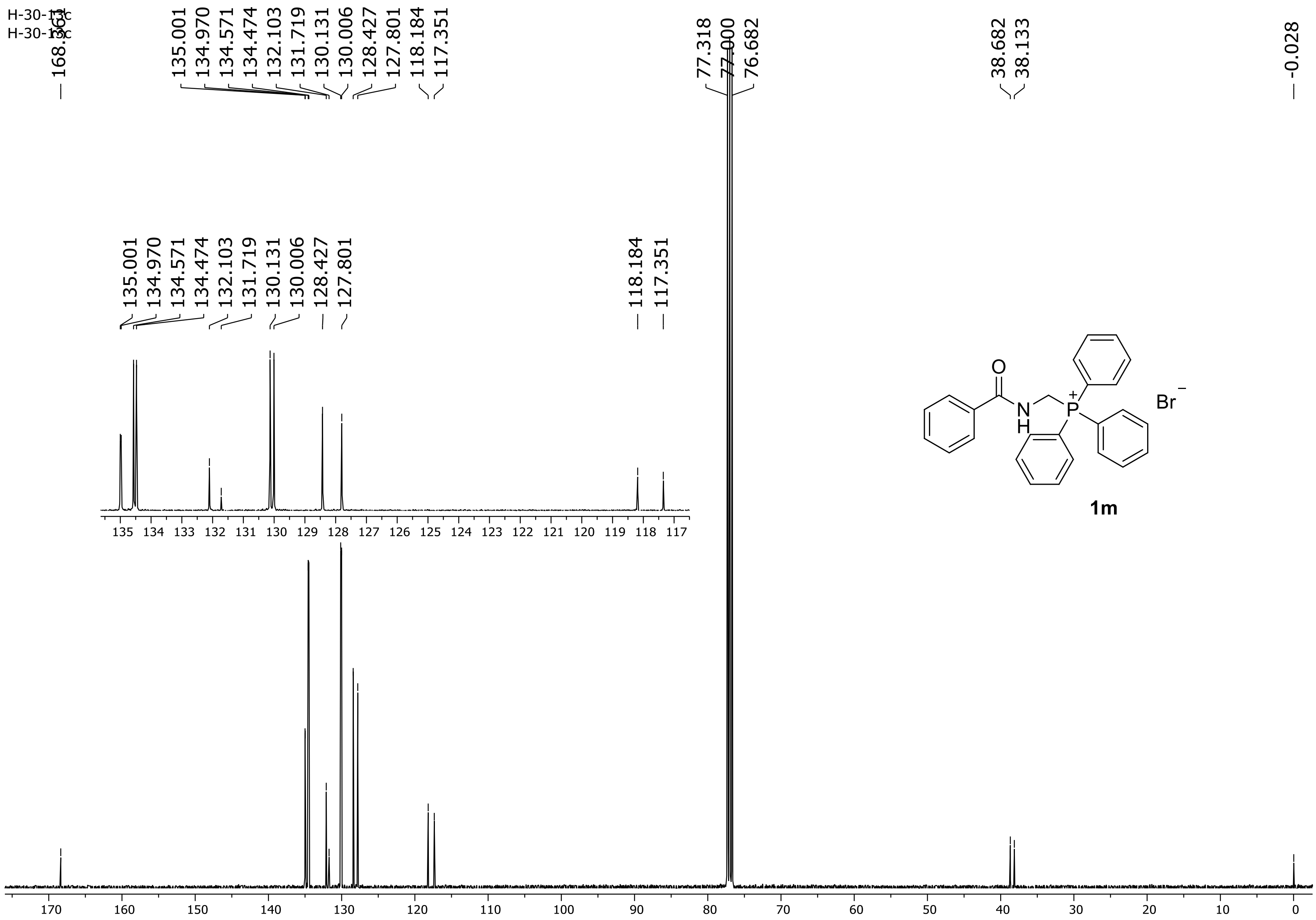

$^{13}C\{^{1}H\}$ NMR spectrum of (*N*-benzoylamino)methyltriphenylphosphonium bromide (**1m**); 100 MHz/$CDCl_3$/TMS; δ (ppm).

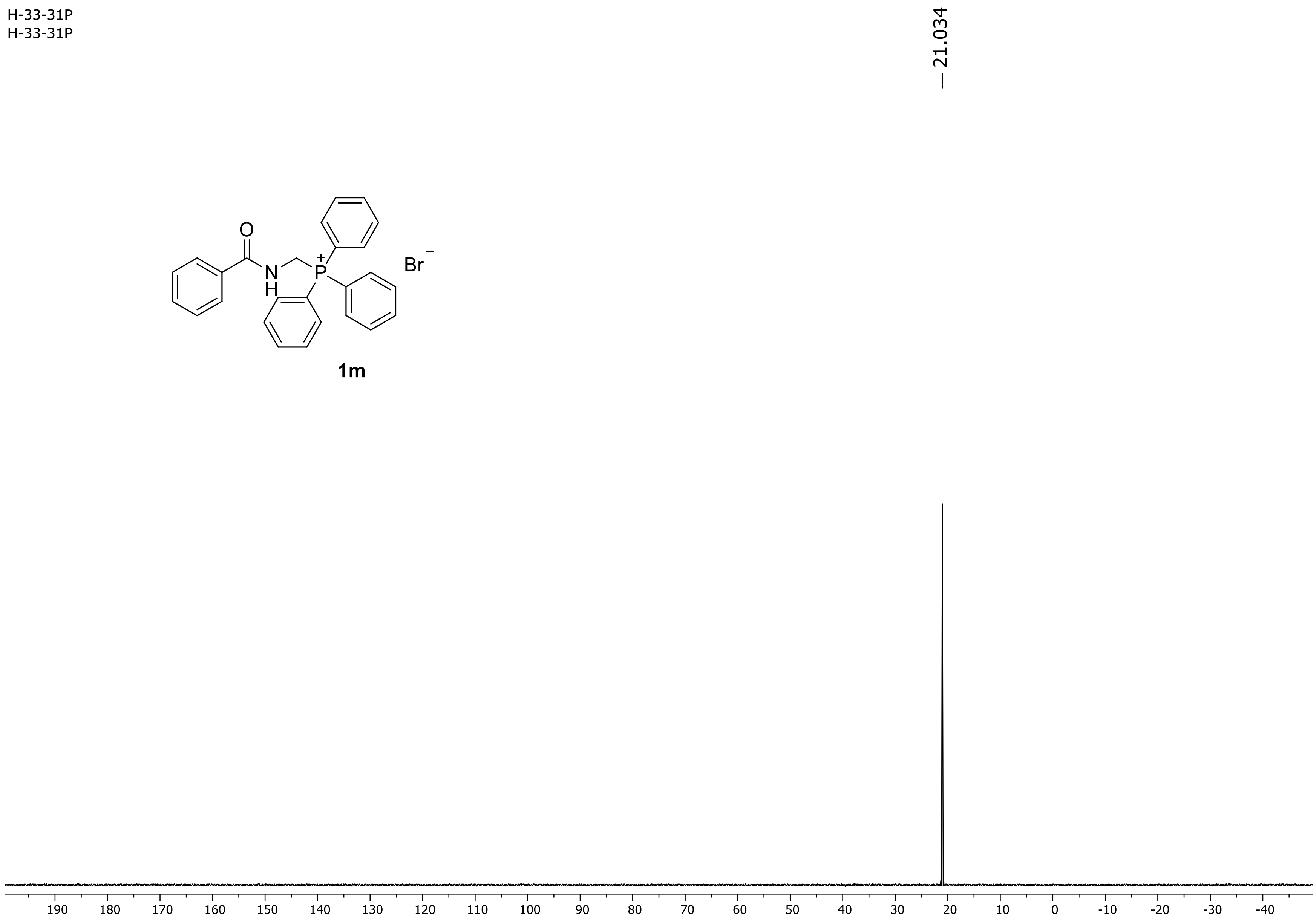

^{31}P NMR spectrum of (*N*-benzoylamino)methyltriphenylphosphonium bromide (**1m**); 161.9 MHz/$CDCl_3$; δ (ppm).

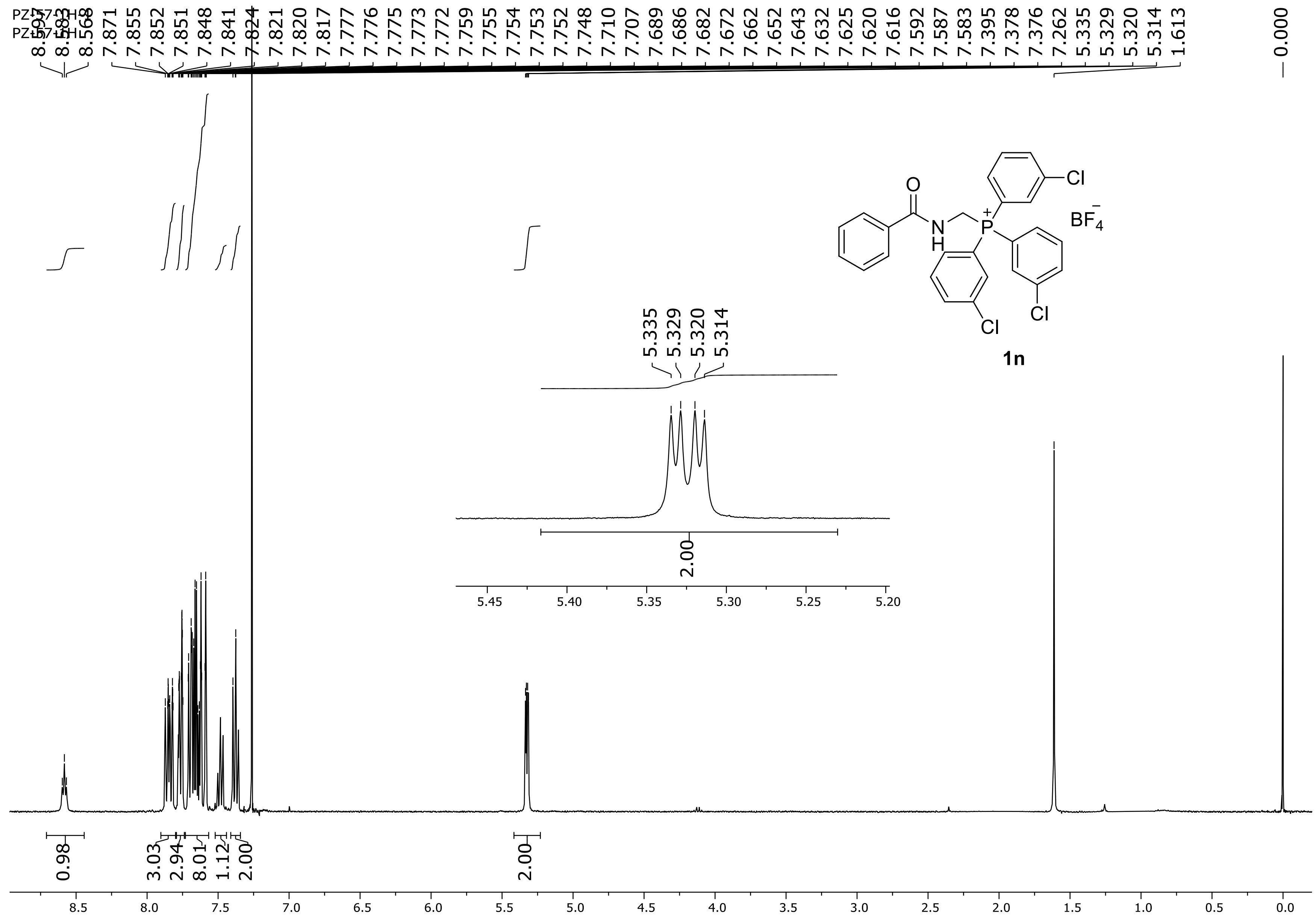

^{1}H NMR spectrum of (*N*-benzoylamino)methyltris(3-chlorophenyl)phosphonium tetrafluoroborate (**1n**); 400 MHz/$CDCl_3$/TMS; δ (ppm).

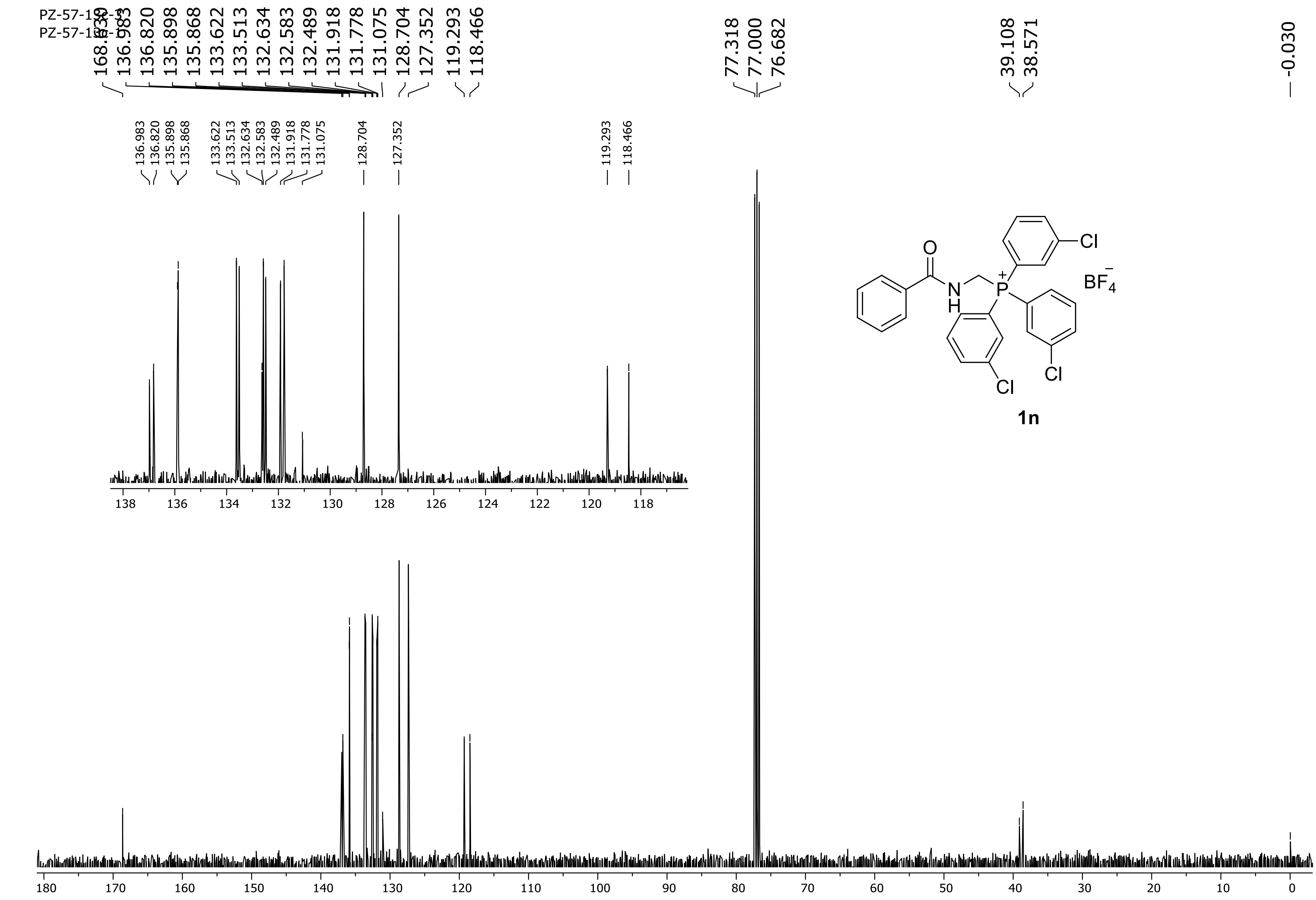

$^{13}C\{^{1}H\}$ NMR spectrum of (*N*-benzoylamino)methyltris(3-chlorophenyl)phosphonium tetrafluoroborate (**1n**); 100 MHz/$CDCl_3$/TMS; δ (ppm).

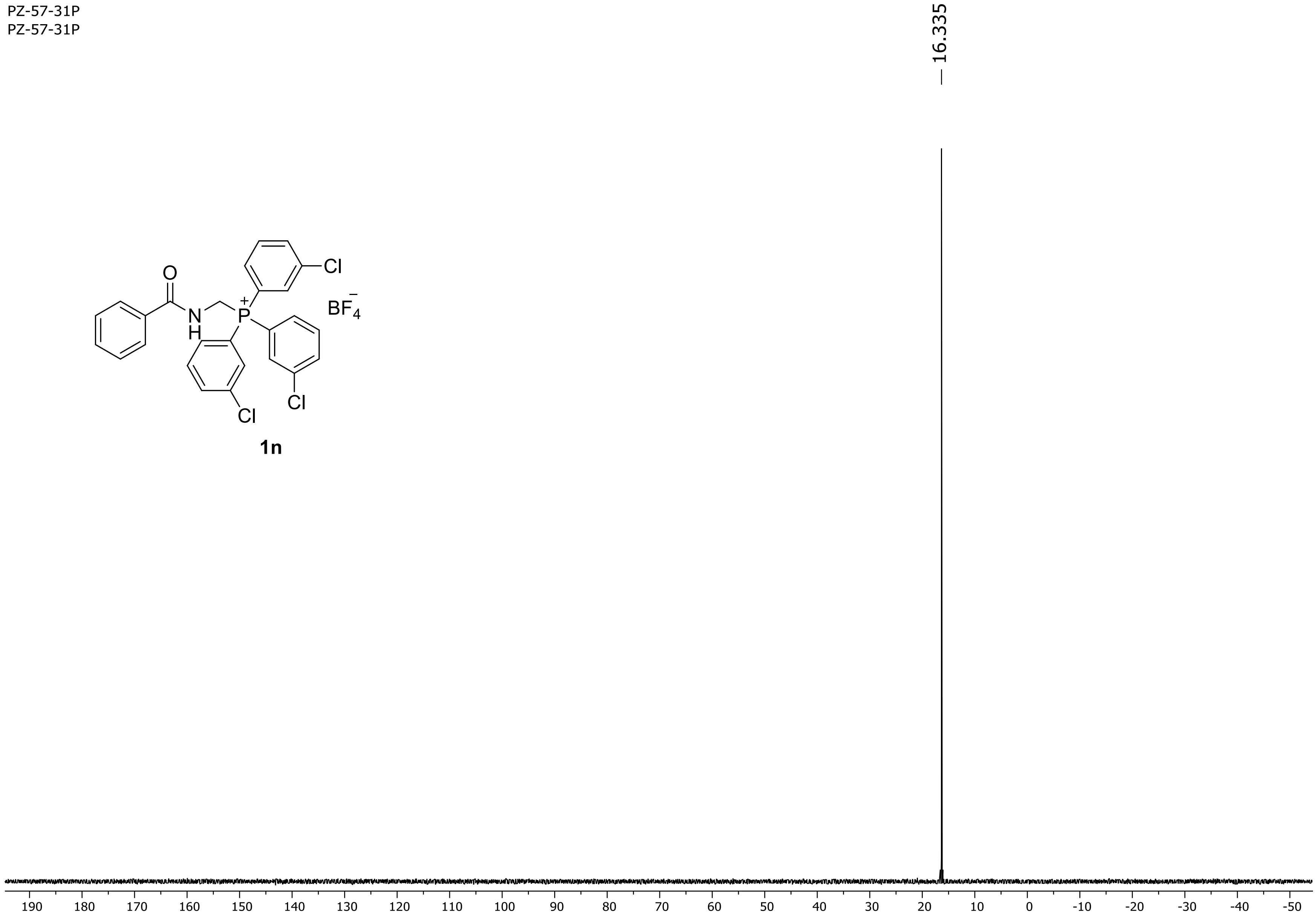

1n

^{31}P NMR spectrum of (*N*-benzoylamino)methyltris(3-chlorophenyl)phosphonium tetrafluoroborate (**1n**); 161.9 MHz/CDCl$_3$; *δ* (ppm).

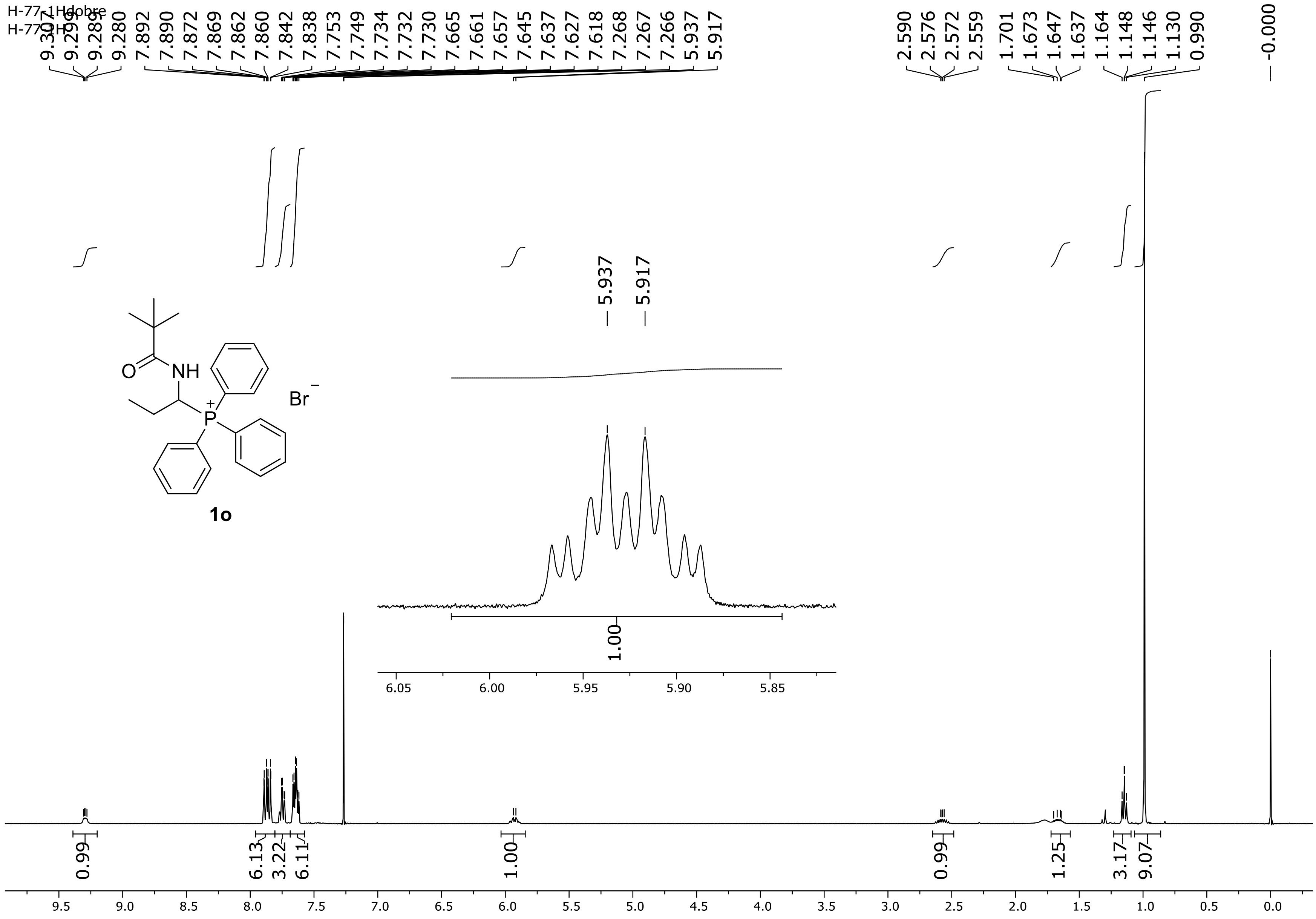

^{1}H NMR spectrum of 1-(*N*-pivaloylamino)propyltriphenylphosphonium bromide (**1o**); 400 MHz/$CDCl_3$/TMS; δ (ppm).

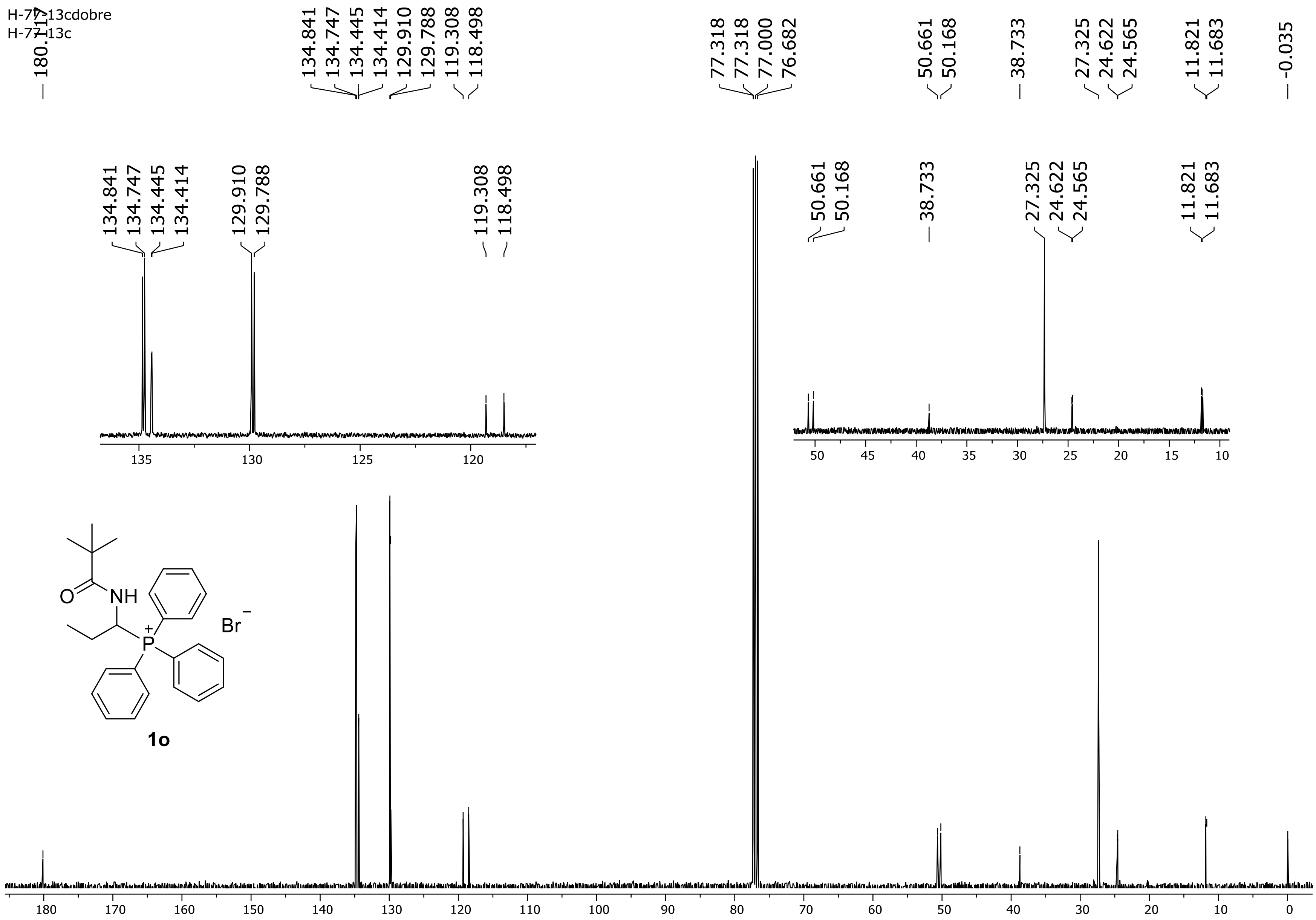

$^{13}C\{^{1}H\}$ NMR spectrum of 1-(*N*-pivaloylamino)propyltriphenylphosphonium bromide (**1o**); 100 MHz/$CDCl_3$/TMS; *δ* (ppm).

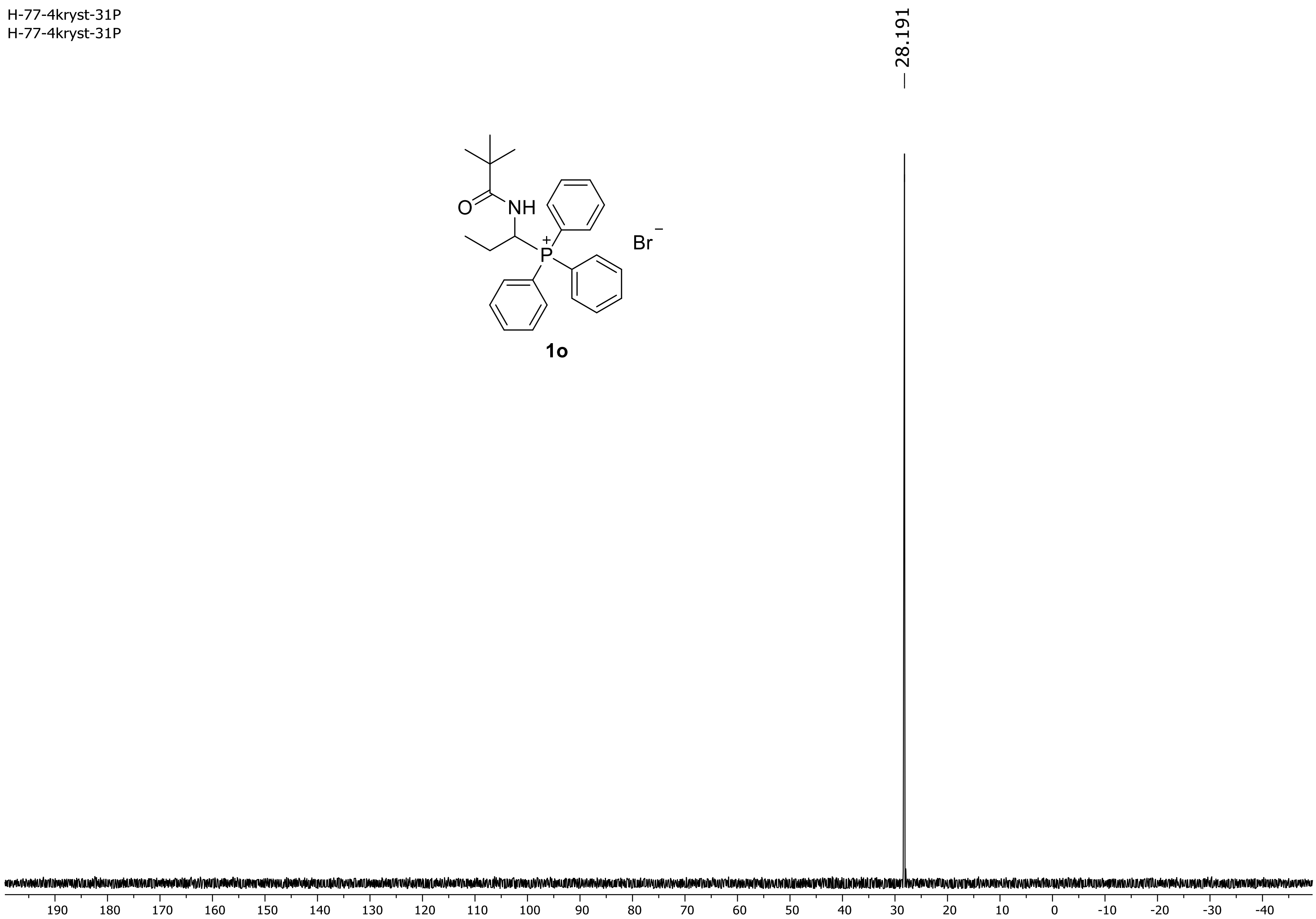

^{31}P NMR spectrum of 1-(*N*-pivaloylamino)propyltriphenylphosphonium bromide (**1o**); 161.9 MHz/$CDCl_3$; δ (ppm).

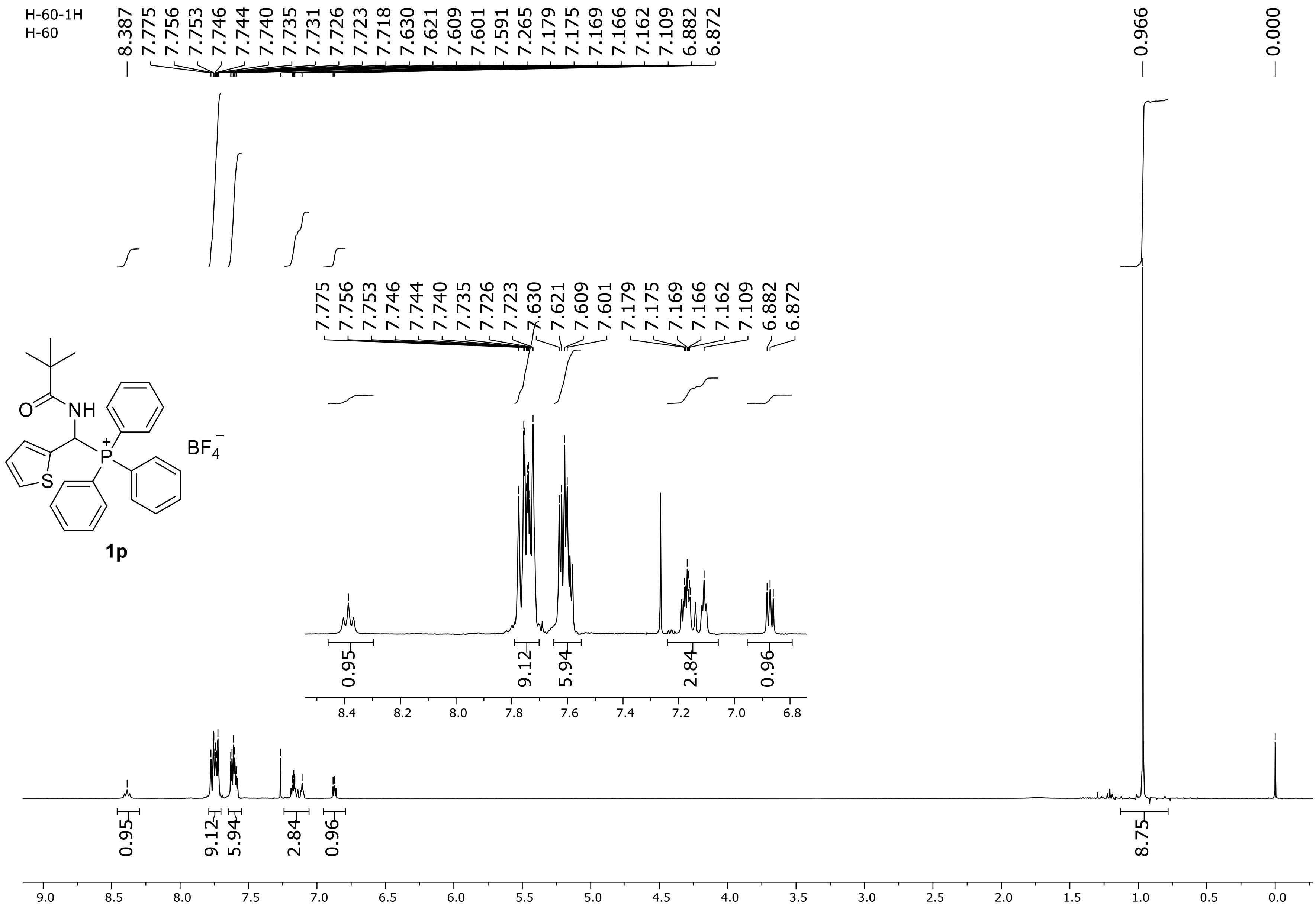

^{1}H NMR spectrum of 1-(*N*-pivaloylamino)-1-(2-thienyl)methyltriphenylphosphonium tetrafluoroborate (**1p**); 400 MHz/$CDCl_3$/TMS; δ (ppm).

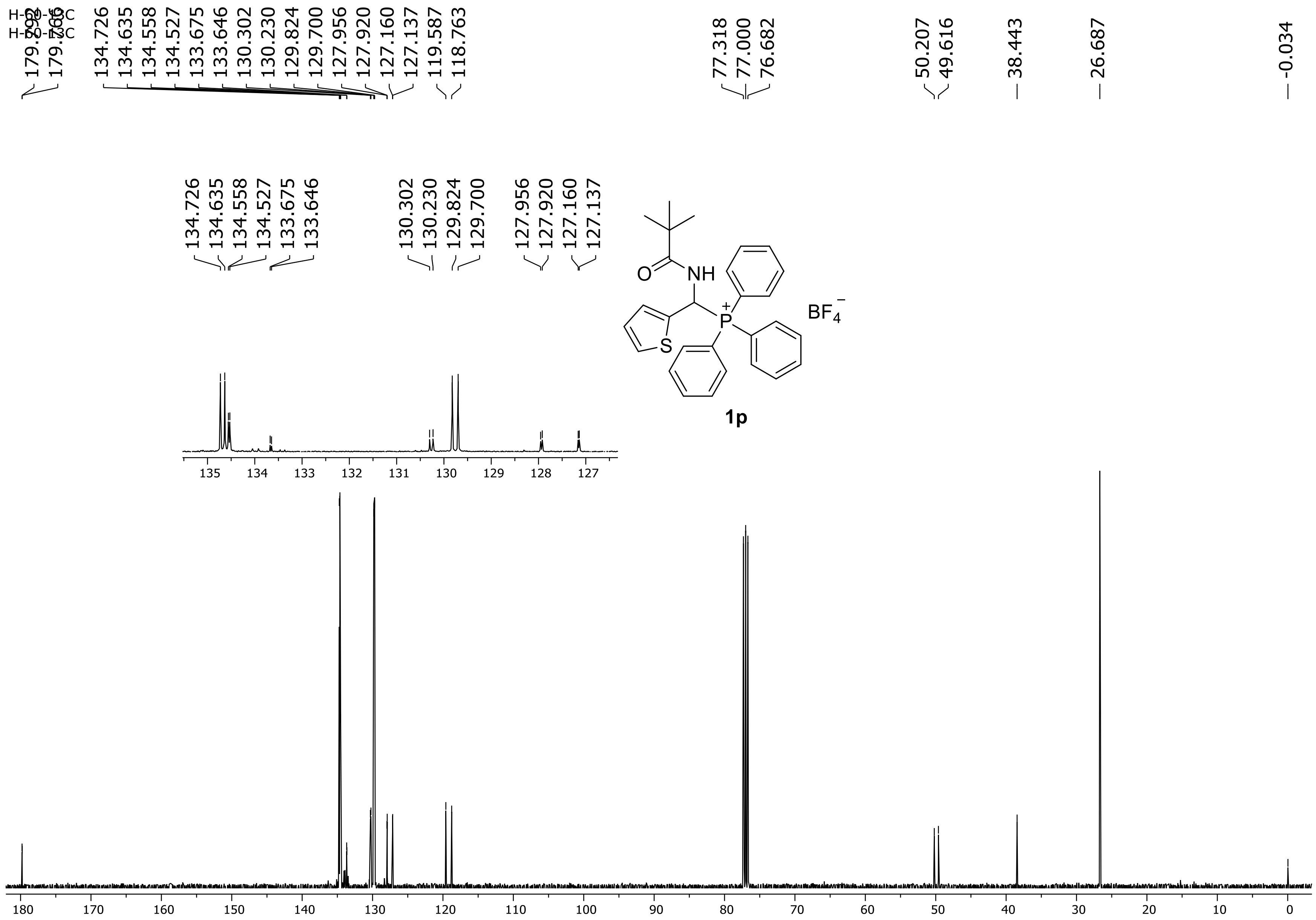

$^{13}C\{^1H\}$ NMR spectrum of 1-(*N*-pivaloylamino)-1-(2-thienyl)methyltriphenylphosphonium tetrafluoroborate (**1p**); 100 MHz/$CDCl_3$/TMS; δ (ppm).

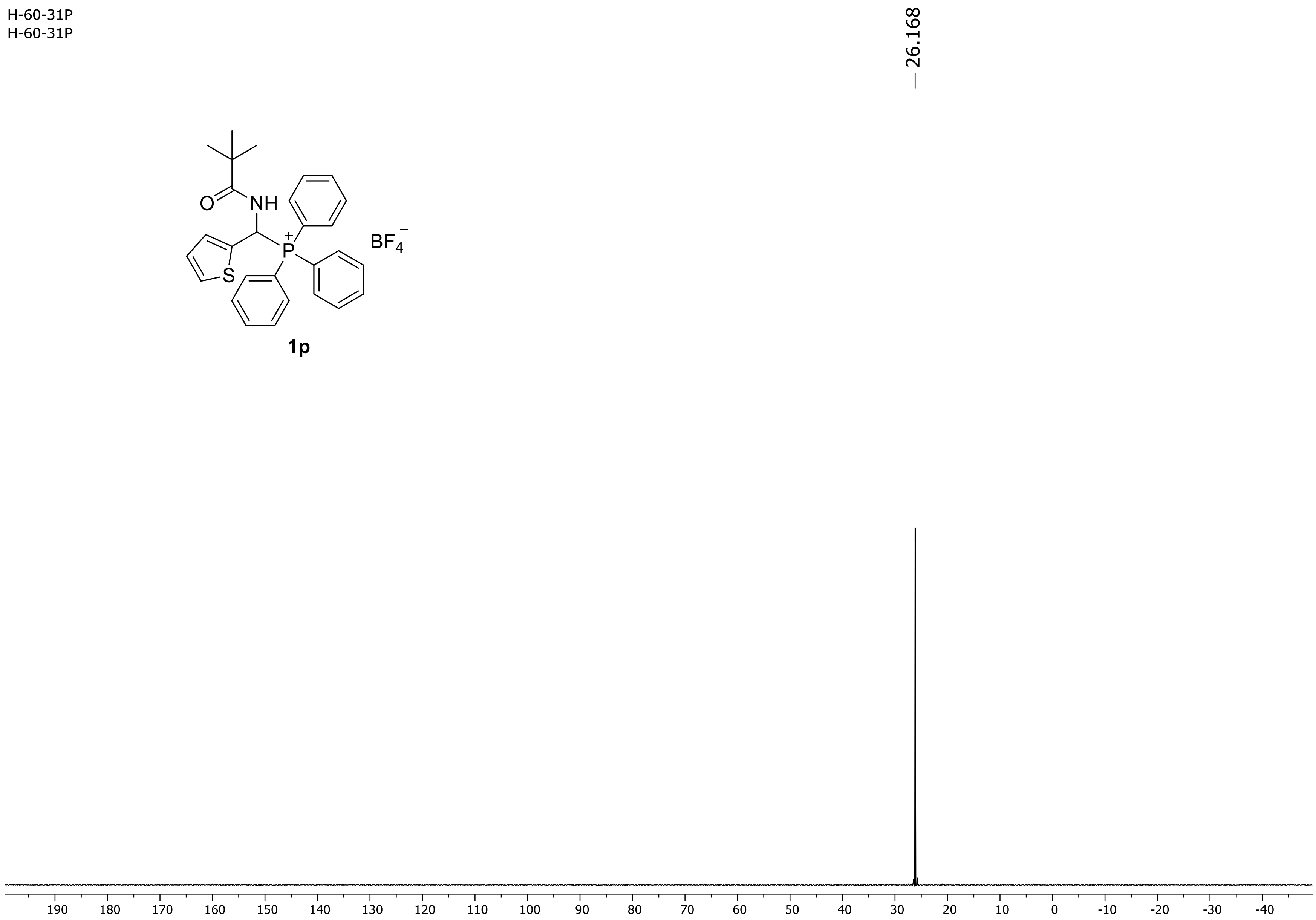

^{31}P NMR spectrum of 1-(*N*-pivaloylamino)-1-(2-thienyl)methyltriphenylphosphonium tetrafluoroborate (**1p**); 161.9 MHz/$CDCl_3$; δ (ppm).

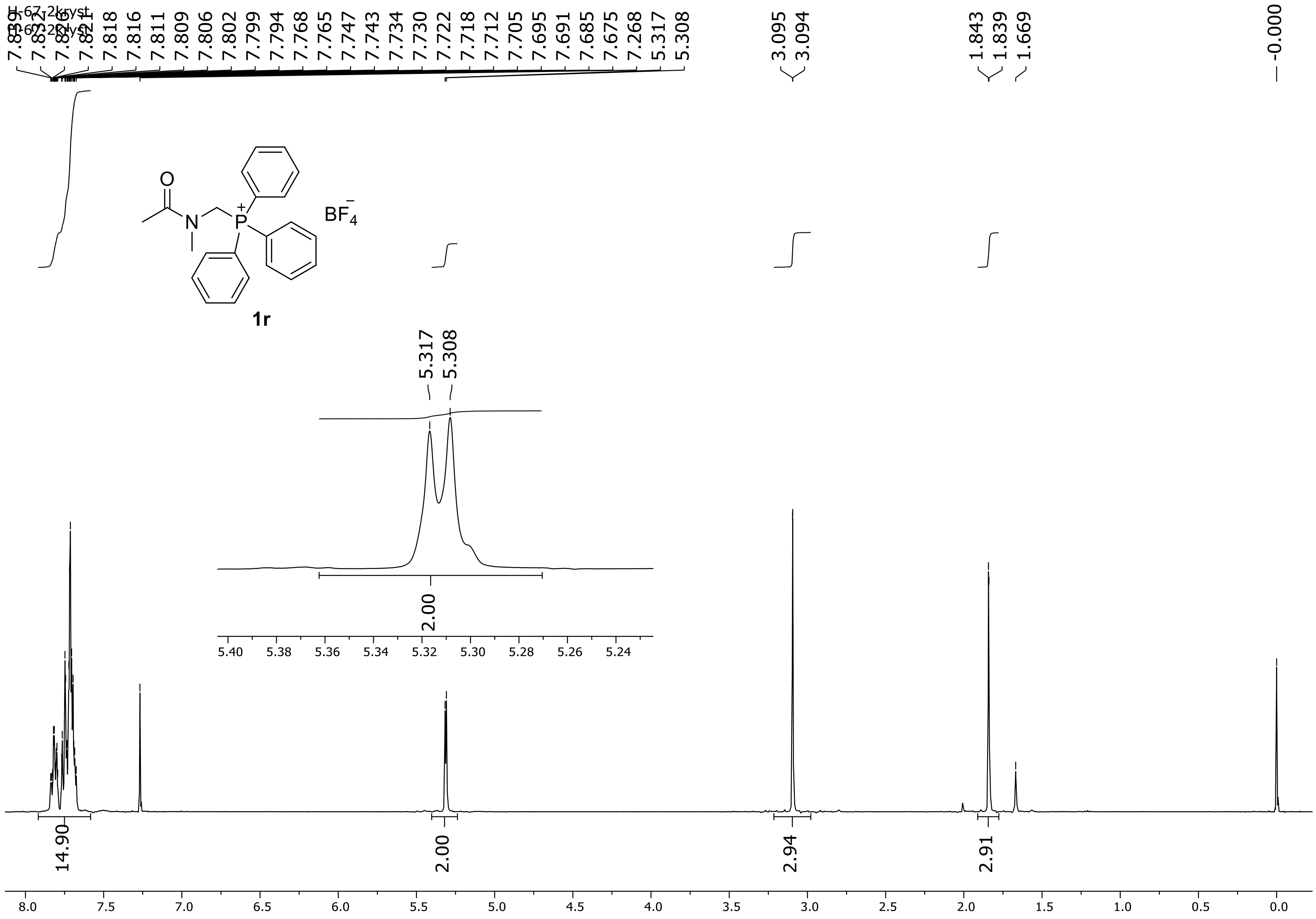

^{1}H NMR spectrum of *N*-(*N*-methylacetylamino)methyltriphenylphosphonium tetrafluoroborate (**1r**); 400 MHz/$CDCl_3$/TMS; δ (ppm).

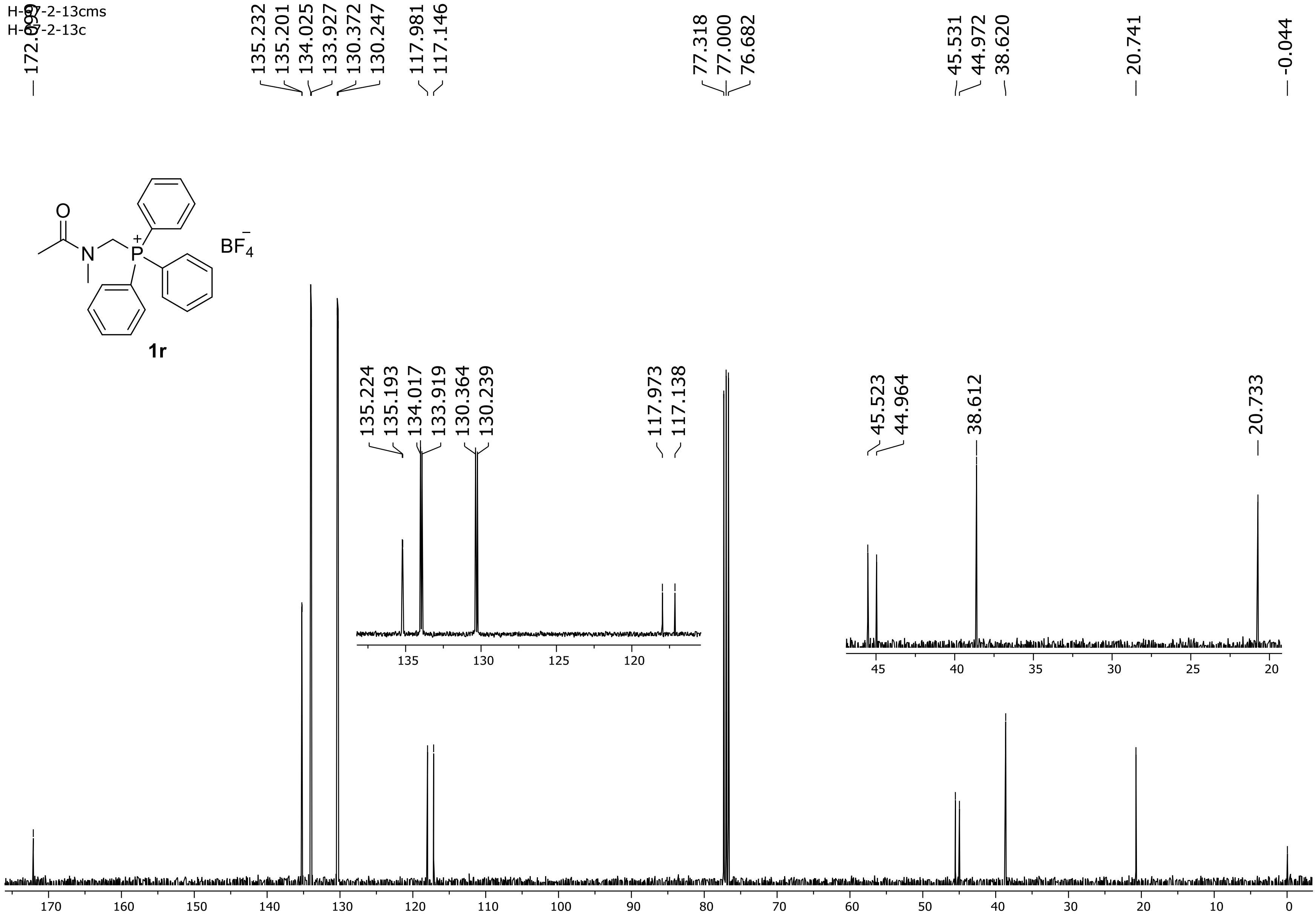

$^{13}C\{^1H\}$ NMR spectrum of *N*-(*N*-methylacetylamino)methyltriphenylphosphonium tetrafluoroborate (**1r**); 100 MHz/$CDCl_3$/TMS; δ (ppm).

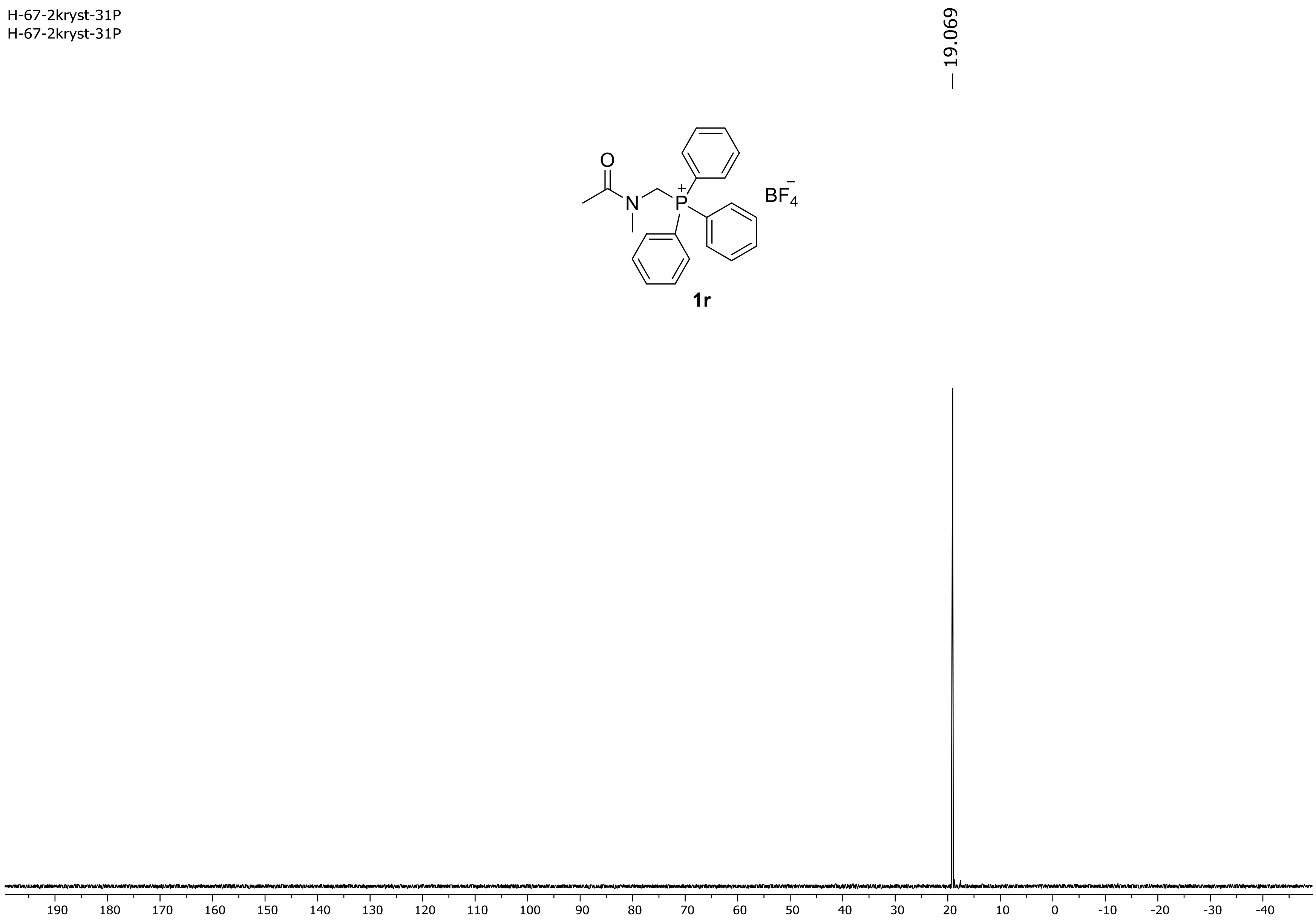

^{31}P NMR spectrum of *N*-(*N*-methylacetylamino)methyltriphenylphosphonium tetrafluoroborate (**1r**); 161.9 MHz/$CDCl_3$; δ (ppm).

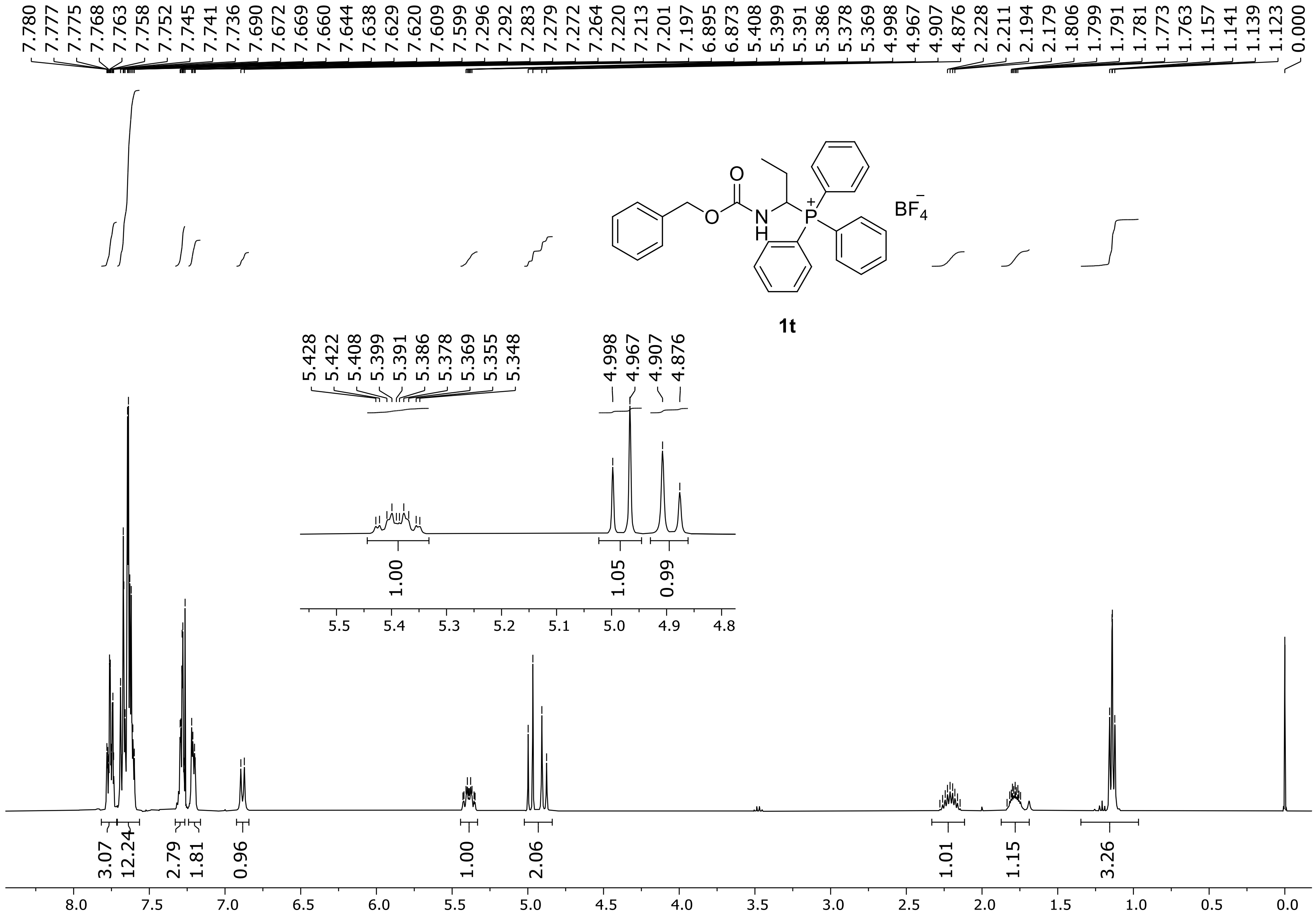

^{1}H NMR spectrum of 1-(*N*-benzyloxycarbonylamino)propyltriphenylphosphonium tetrafluoroborate (**1t**); 400 MHz/$CDCl_3$/TMS; δ (ppm).

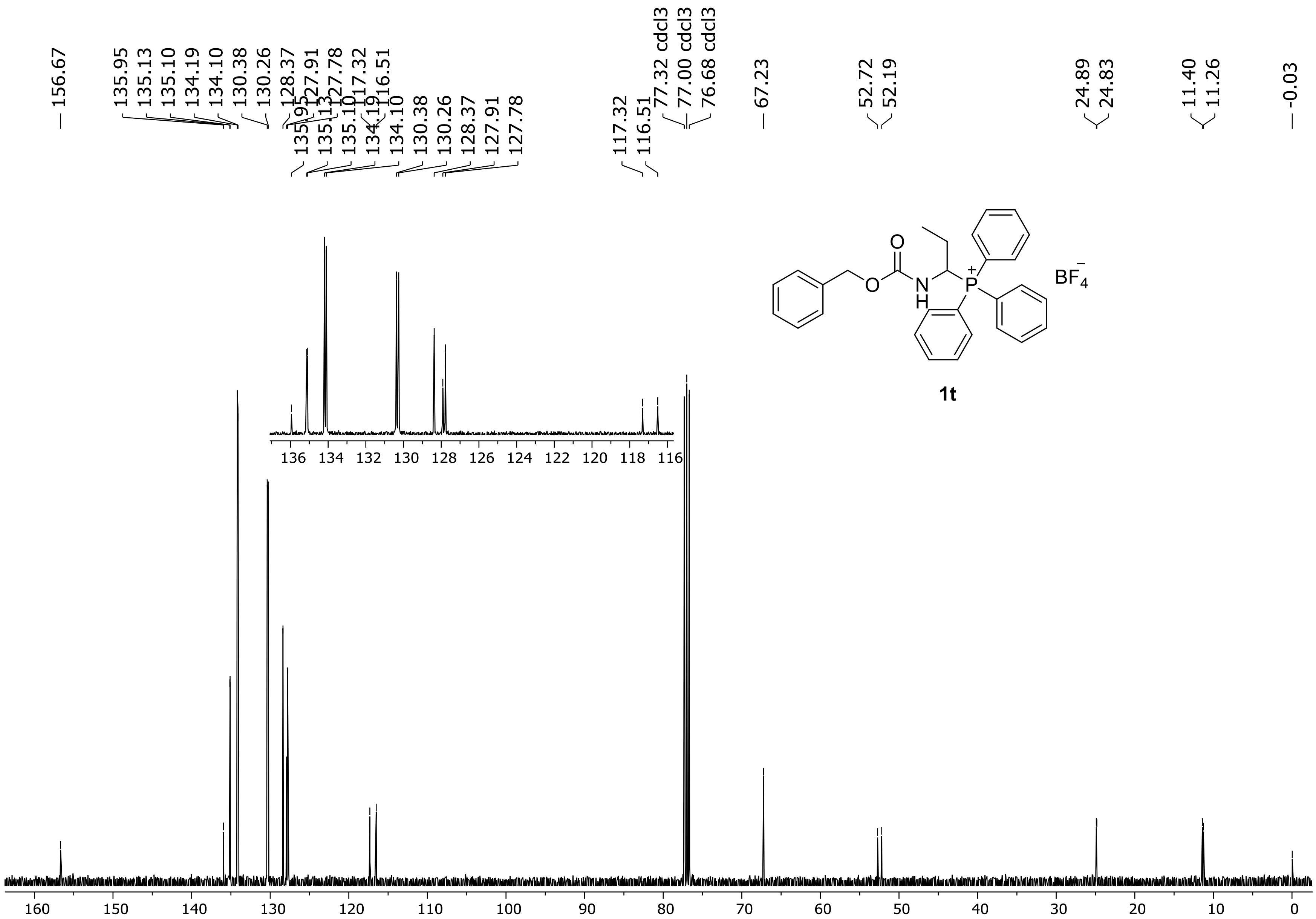

$^{13}C\{^{1}H\}$ NMR spectrum of 1-(*N*-benzyloxycarbonylamino)propyltriphenylphosphonium tetrafluoroborate (**1t**); 100 MHz/$CDCl_3$/TMS; δ (ppm).

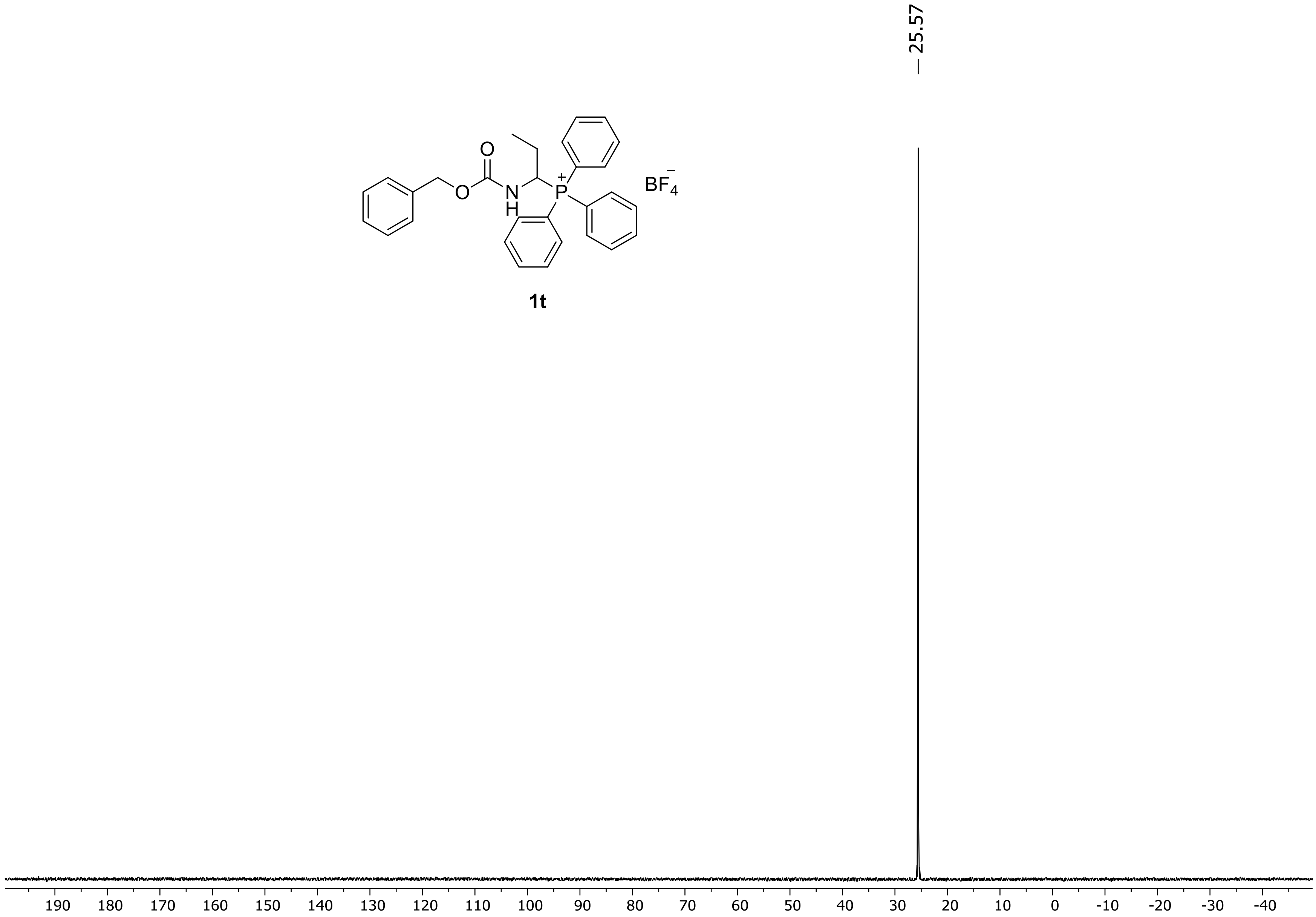

^{31}P NMR spectrum of 1-(*N*-benzyloxycarbonylamino)propyltriphenylphosphonium tetrafluoroborate (**1t**); 161.9 MHz/$CDCl_3$; δ (ppm).

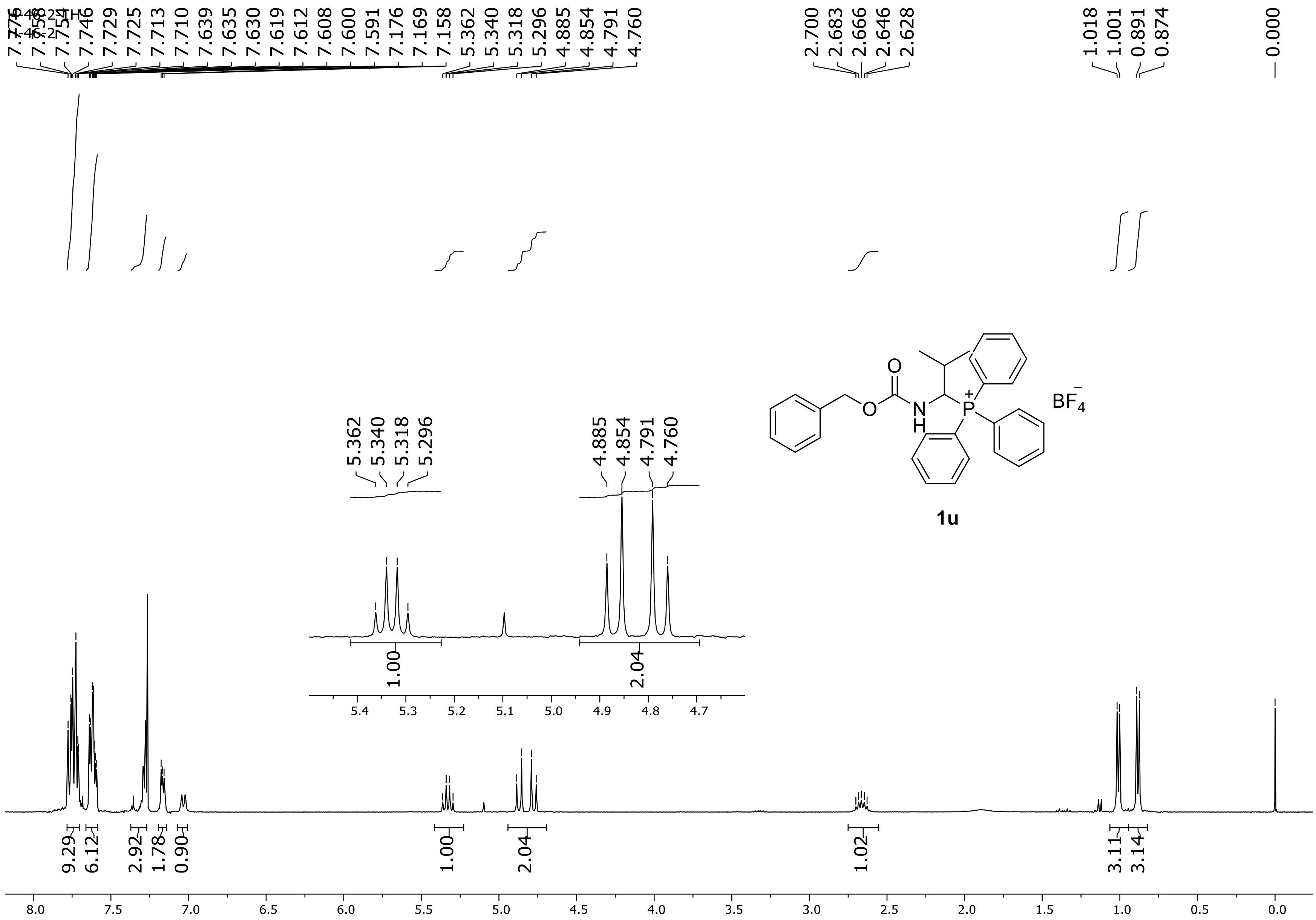

^{1}H NMR spectrum of 1-(*N*-benzyloxycarbonylamino)-2-methylpropyltriphenylphosphonium tetrafluoroborate (**1u**); 400 MHz/CDCl$_3$/TMS; δ (ppm).

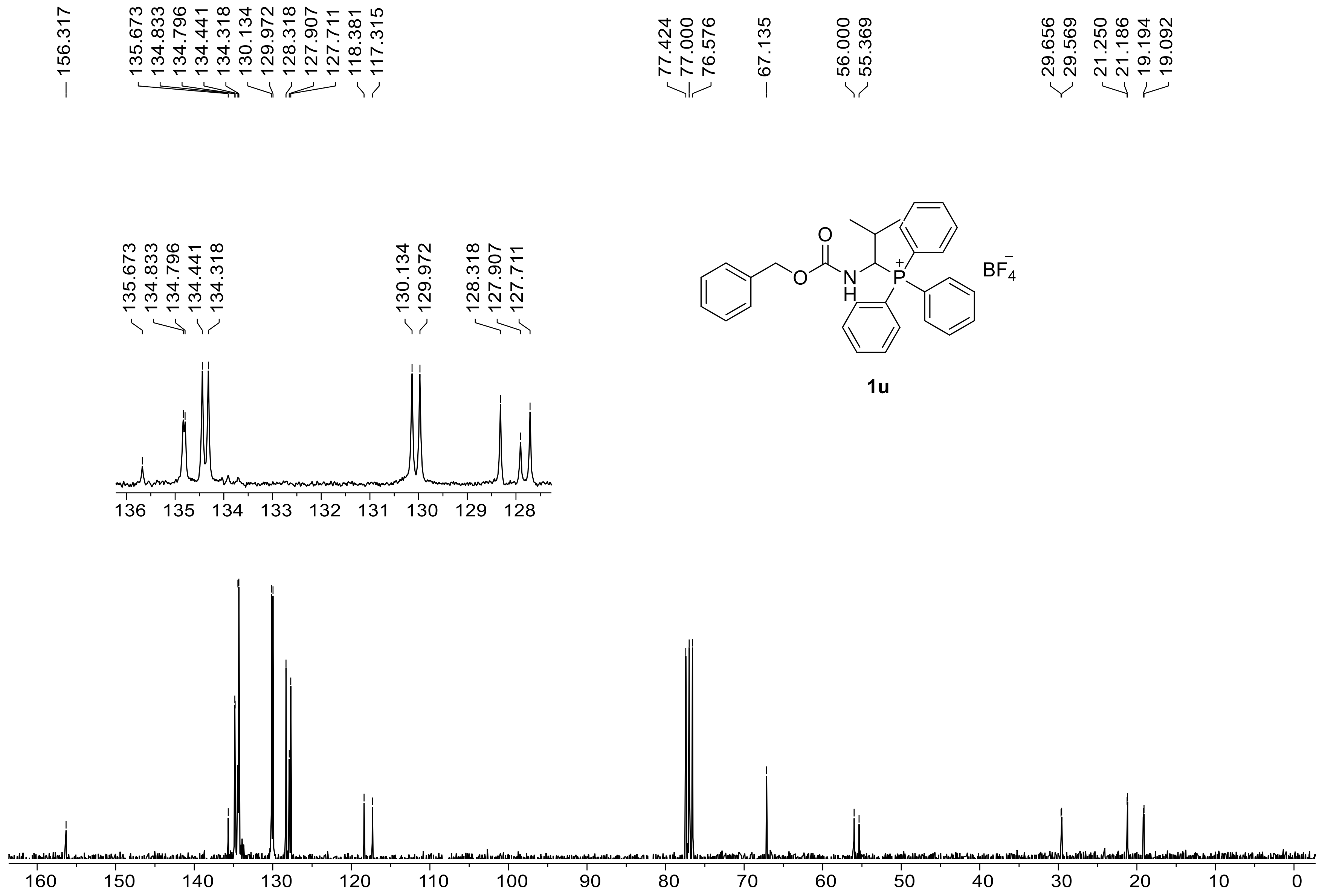

$^{13}C\{^{1}H\}$ NMR spectrum of 1-(*N*-benzyloxycarbonylamino)-2-methylpropyltriphenylphosphonium tetrafluoroborate (**1u**); 100 MHz/$CDCl_3$/TMS; δ (ppm).

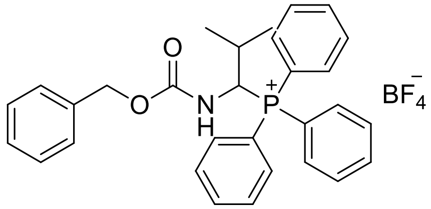

1u

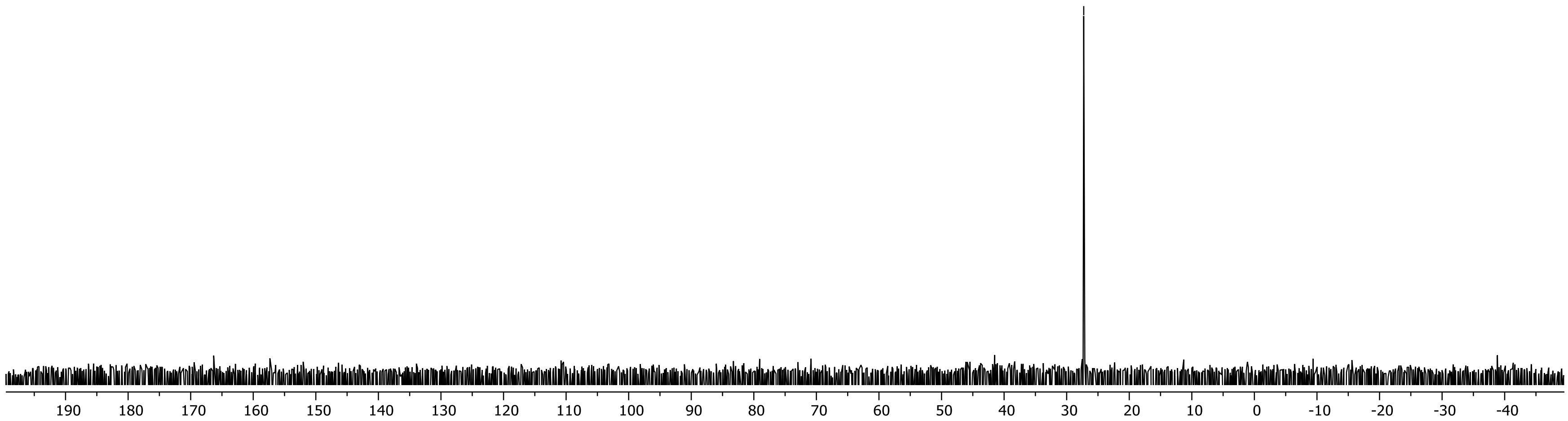

^{31}P NMR spectrum of 1-(*N*-benzyloxycarbonylamino)-2-methylpropyltriphenylphosphonium tetrafluoroborate (**1u**); 161.9 MHz/$CDCl_3$; δ (ppm).

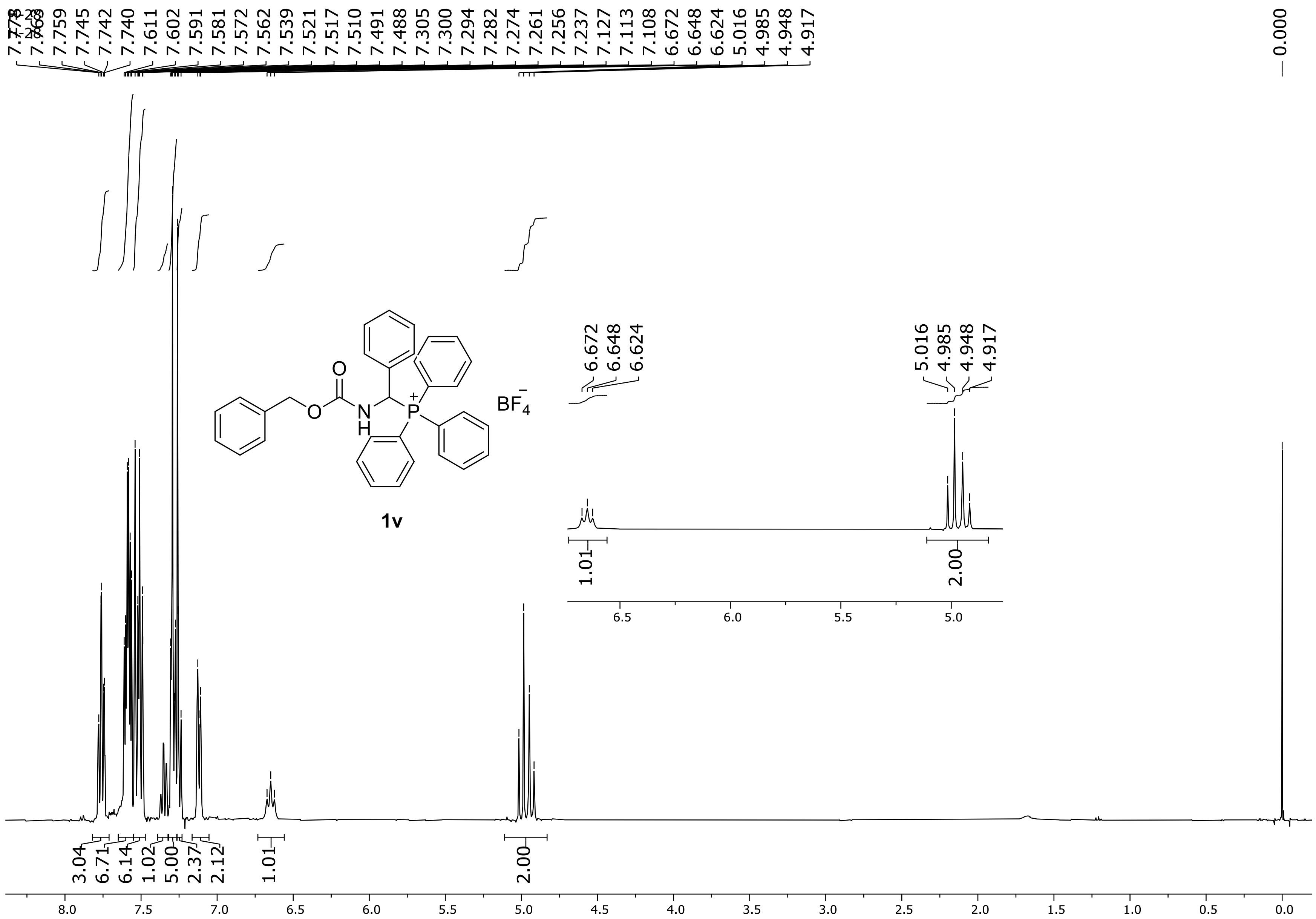

^{1}H NMR spectrum of (*N*-benzyloxycarbonylamino)phenylmethyltriphenylphosphonium tetrafluoroborate (**1v**); 400 MHz/$CDCl_3$/TMS; δ (ppm).

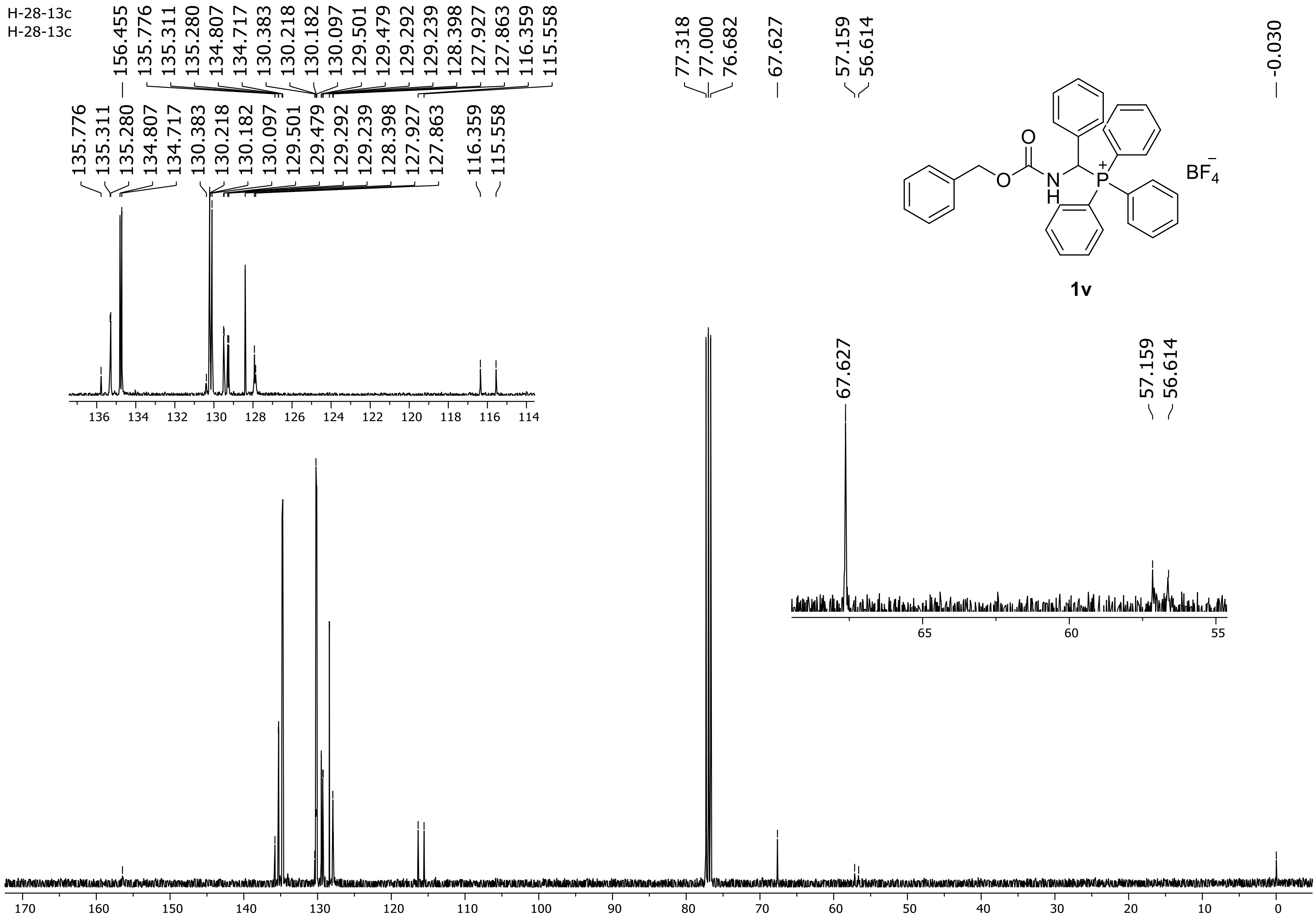

$^{13}C\{^{1}H\}$ NMR spectrum of (*N*-benzyloxycarbonylamino)phenylmethyltriphenylphosphonium tetrafluoroborate (**1v**); 100 MHz/$CDCl_3$/TMS; δ (ppm).

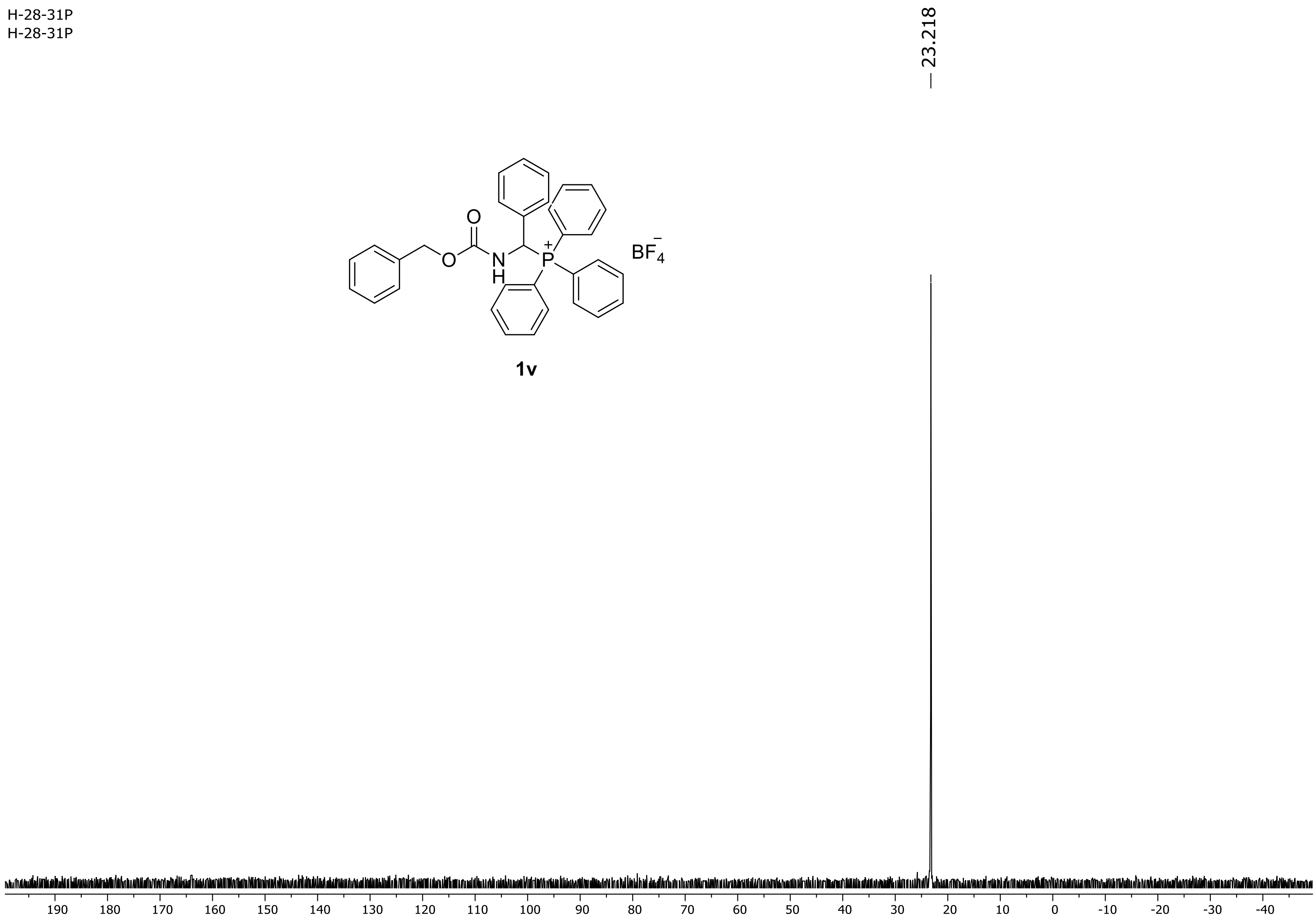

^{31}P NMR spectrum of (*N*-benzyloxycarbonylamino)phenylmethyltriphenylphosphonium tetrafluoroborate (**1v**); 161.9 MHz/$CDCl_3$; δ (ppm).

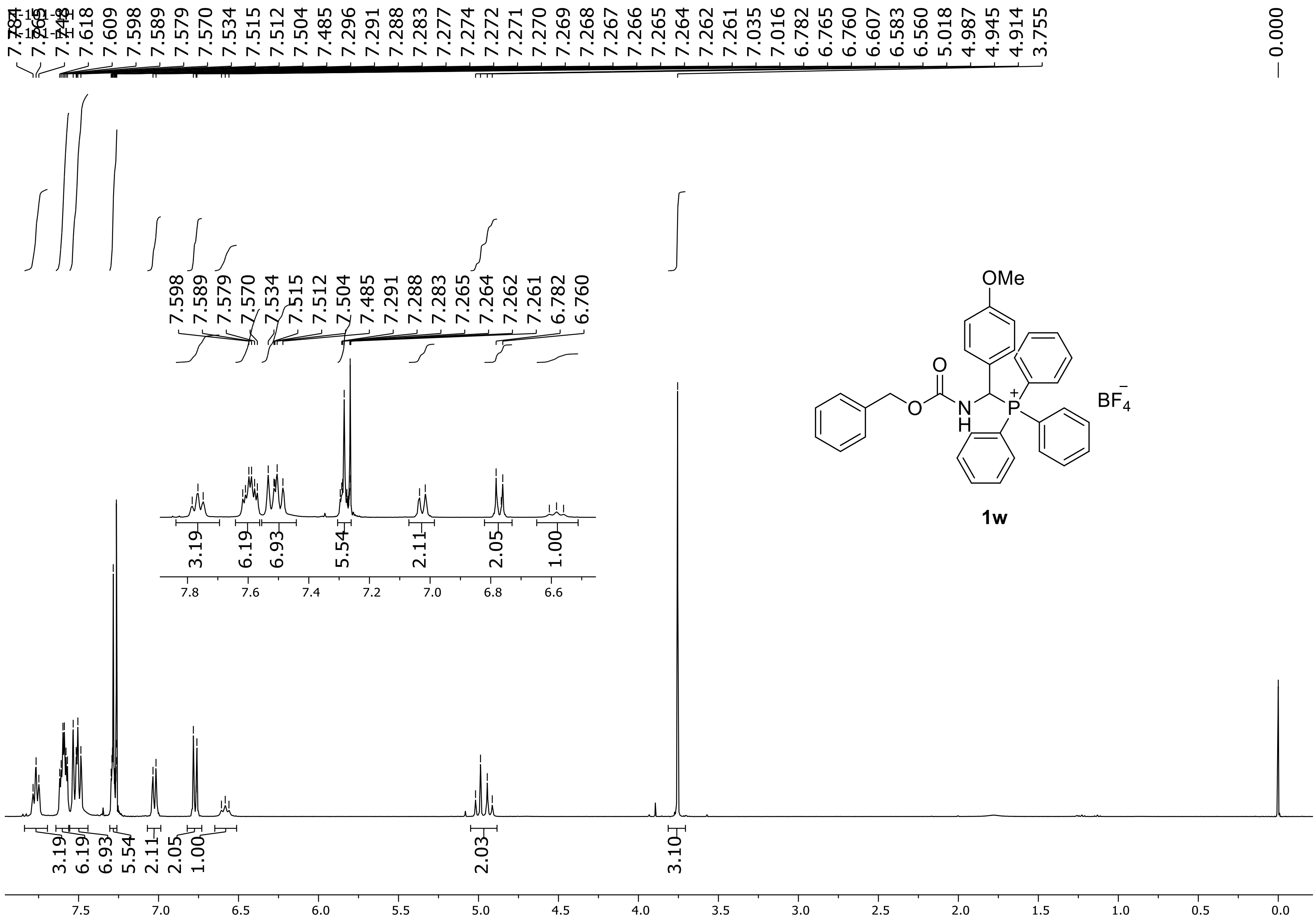

^{1}H NMR spectrum of 1-(*N*-benzyloxycarbonylamino)-1-(4-methoxyphenyl)methyltriphenylphosphonium tetrafluoroborate (**1w**); 400 MHz/$CDCl_3$/TMS; δ (ppm).

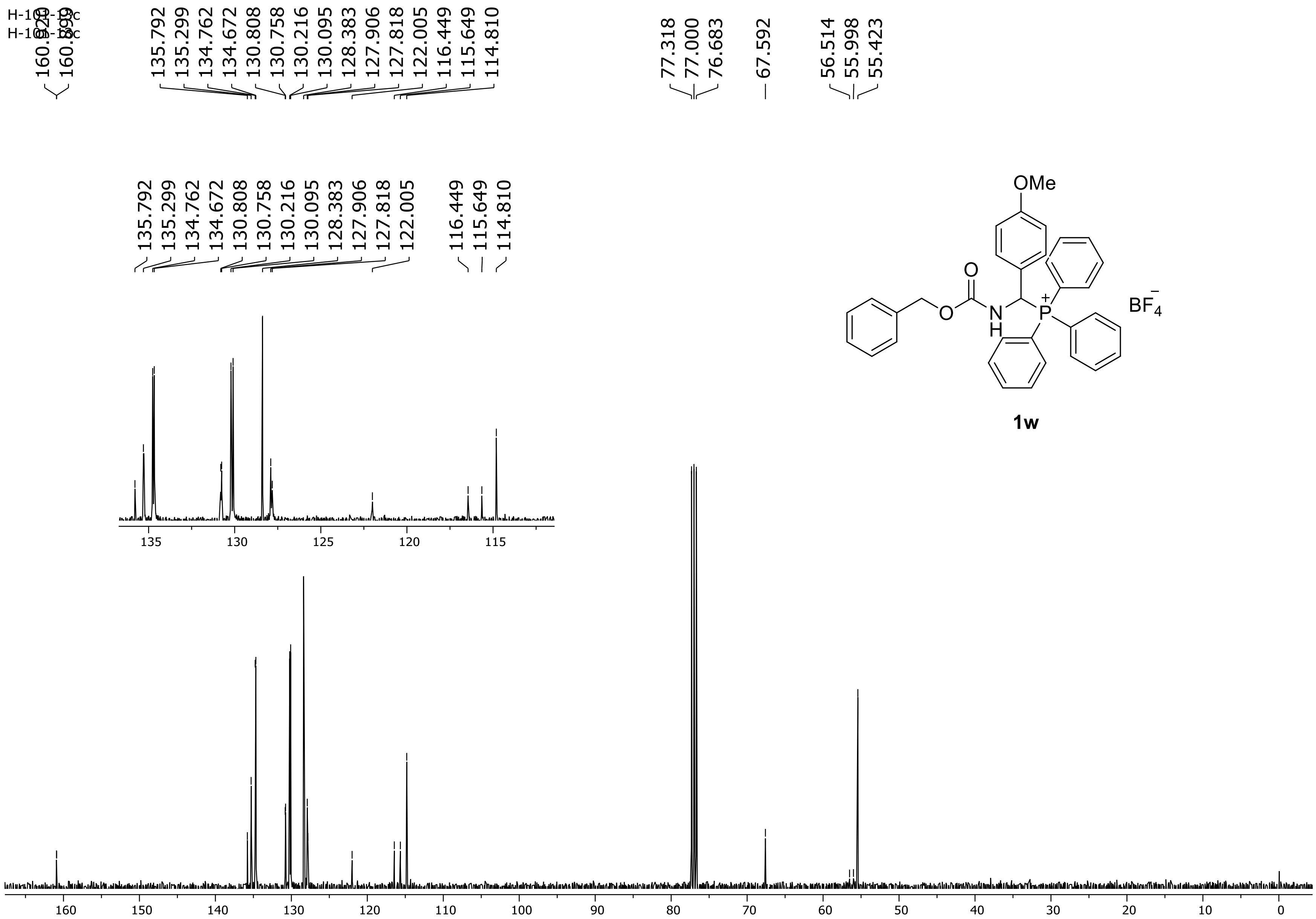

$^{13}C\{^{1}H\}$ NMR spectrum of 1-(*N*-benzyloxycarbonylamino)-1-(4-methoxyphenyl)methyltriphenylphosphonium tetrafluoroborate (**1w**); 100 MHz/$CDCl_3$/TMS; δ (ppm).

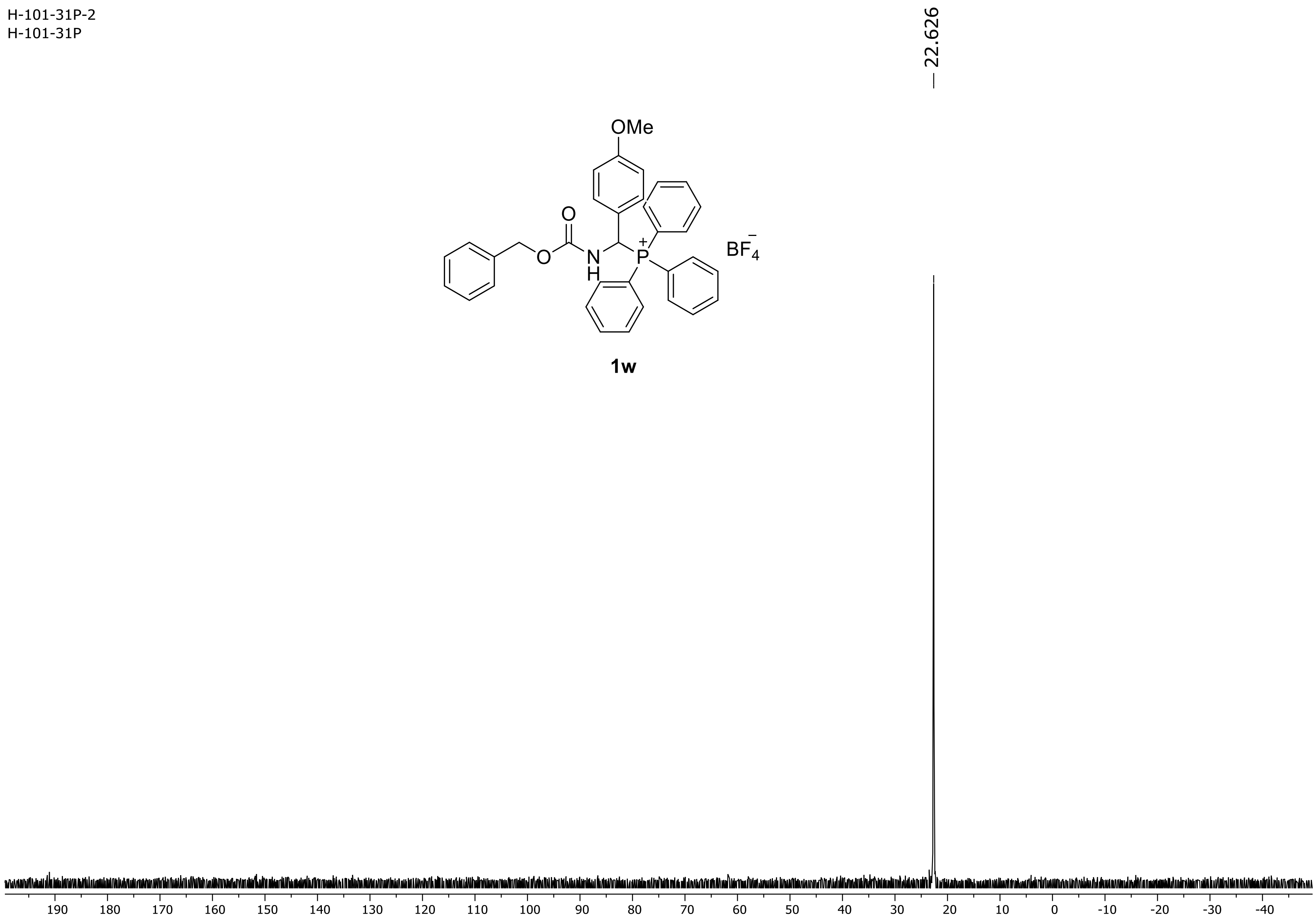

^{31}P NMR spectrum of 1-(*N*-benzyloxycarbonylamino)-1-(4-methoxyphenyl)methyltriphenylphosphonium tetrafluoroborate (**1w**); 161.9 MHz/$CDCl_3$; δ (ppm).

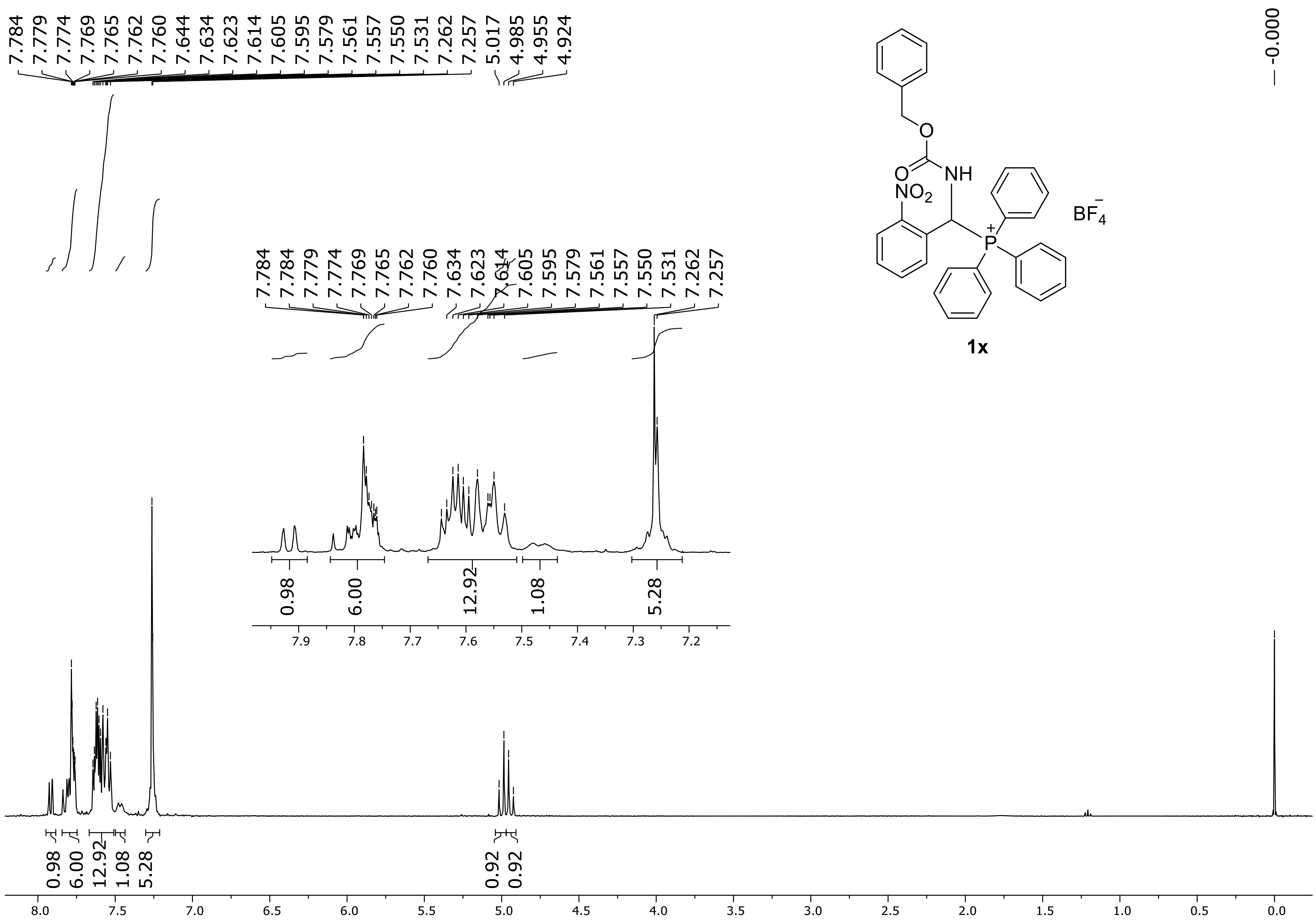

^{1}H NMR spectrum of 1-(*N*-benzyloxycarbonylamino)-1-(2-nitrophenyl)methyltriphenylphosphonium tetrafluoroborate (**1x**); 400 MHz/$CDCl_3$/TMS; δ (ppm).

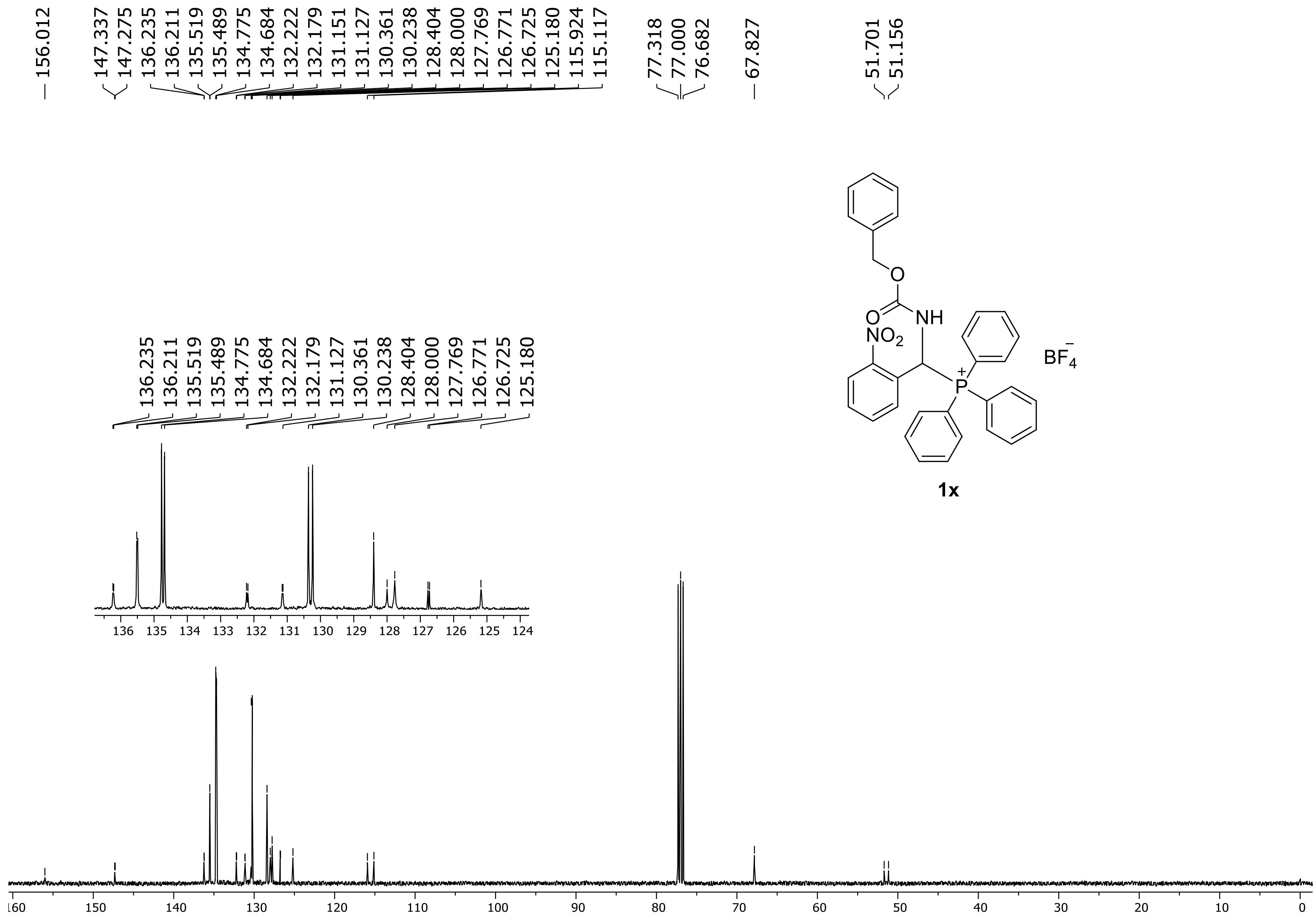

$^{13}C\{^{1}H\}$ NMR spectrum of 1-(*N*-benzyloxycarbonylamino)-1-(2-nitrophenyl)methyltriphenylphosphonium tetrafluoroborate (**1x**); 100 MHz/$CDCl_3$/TMS; δ (ppm).

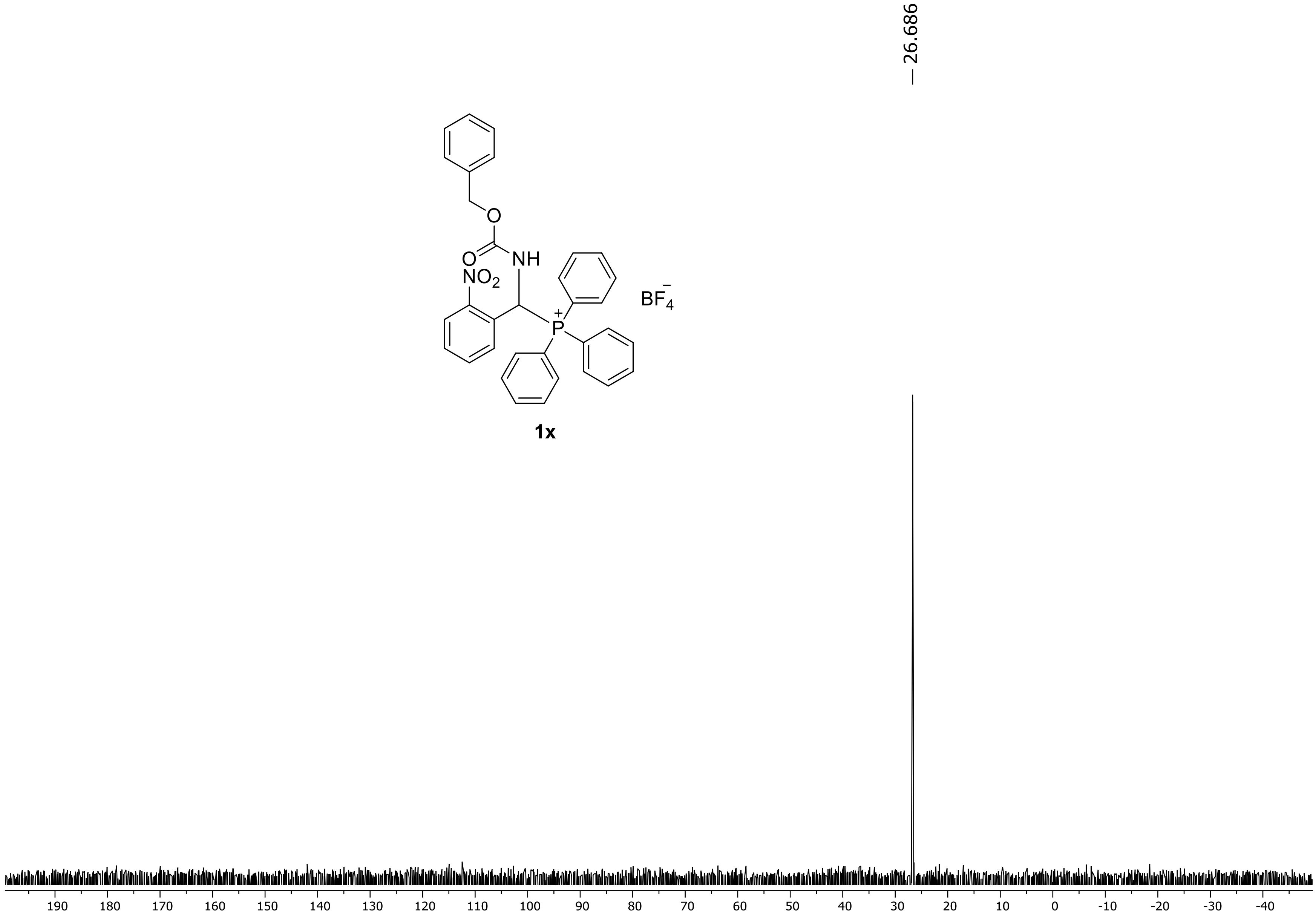

^{31}P NMR spectrum of 1-(*N*-benzyloxycarbonylamino)-1-(2-nitrophenyl)methyltriphenylphosphonium tetrafluoroborate (**1x**); 161.9 MHz/$CDCl_3$; δ (ppm).

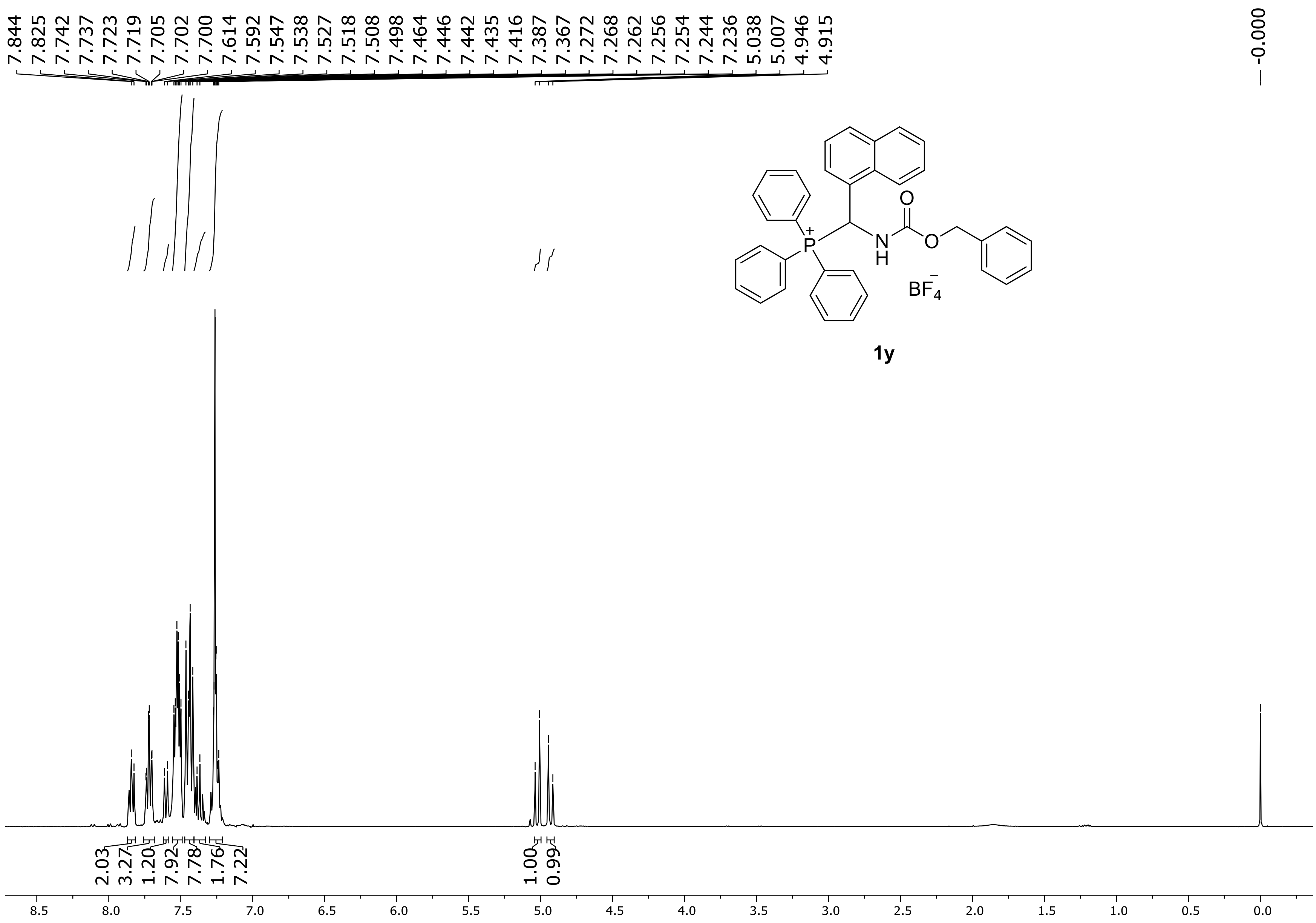

^{1}H NMR spectrum of 1-(*N*-benzyloxycarbonylamino)-1-(1-naphthyl)methyltriphenylphosphonium tetrafluoroborate (**1y**); 400 MHz/$CDCl_3$/TMS; δ (ppm).

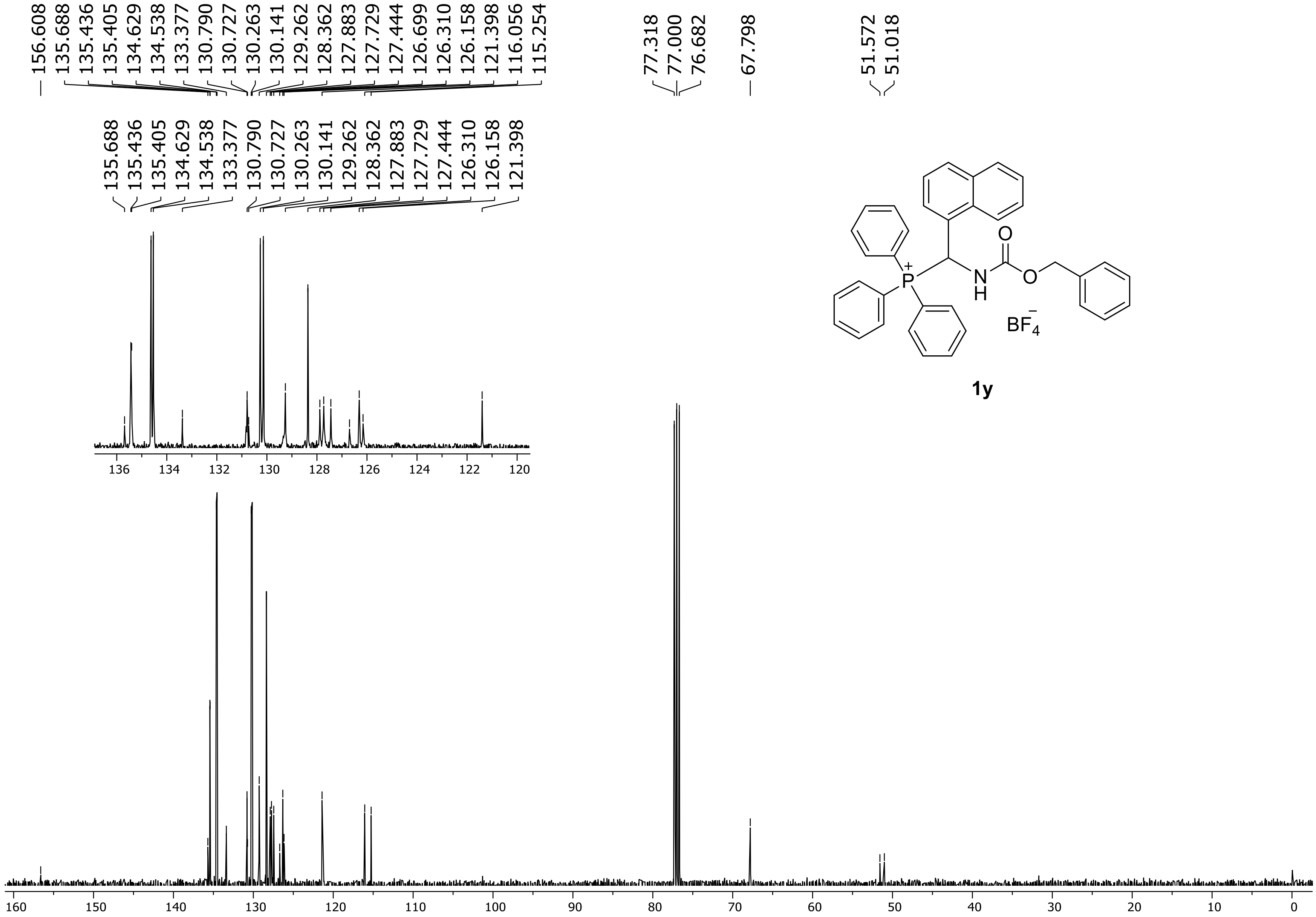

$^{13}C\{^1H\}$ NMR spectrum of 1-(*N*-benzyloxycarbonylamino)-1-(1-naphthyl)methyltriphenylphosphonium tetrafluoroborate (**1y**); 100 MHz/$CDCl_3$/TMS; δ (ppm).

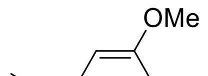

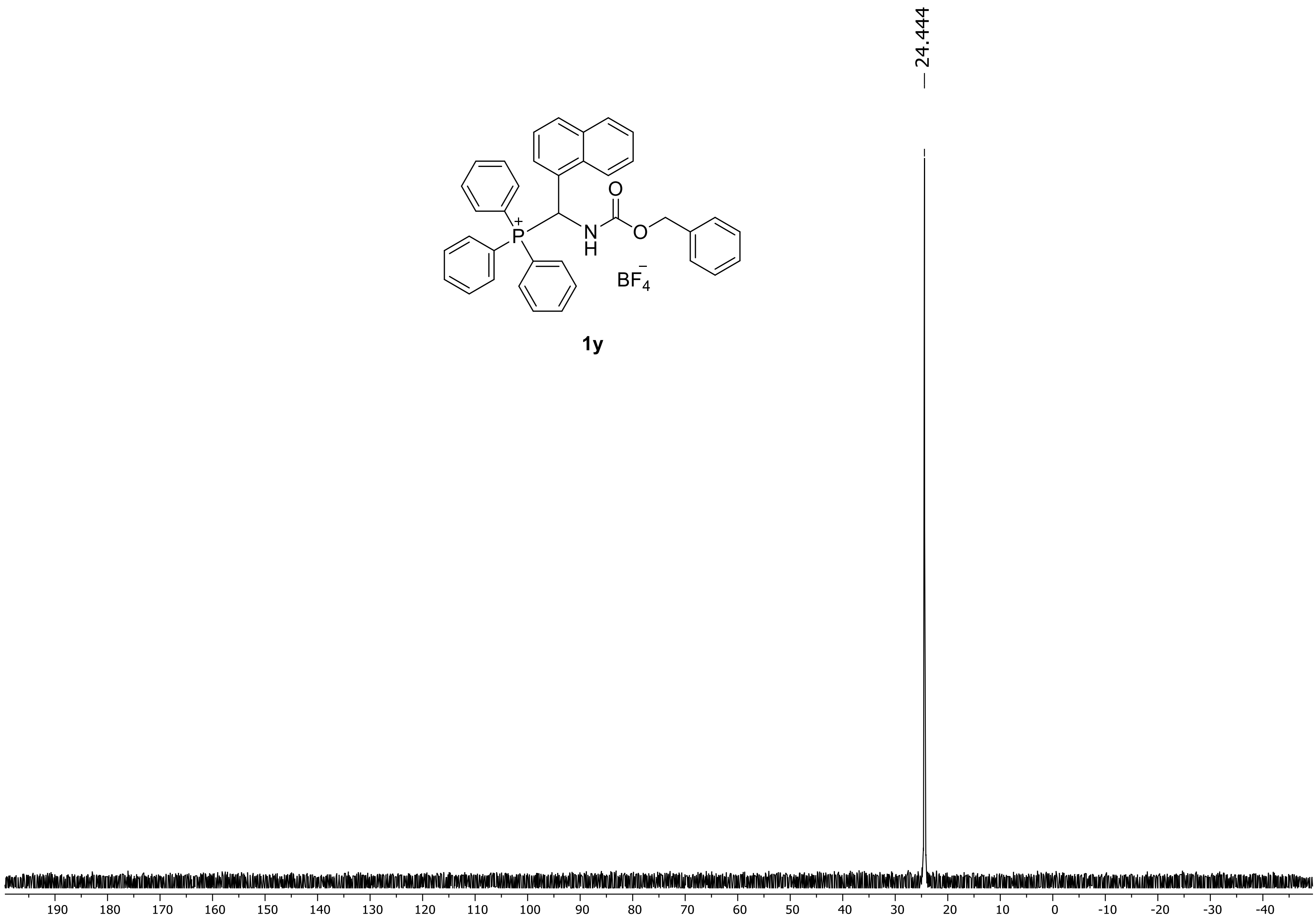

^{31}P NMR spectrum of 1-(*N*-benzyloxycarbonylamino)-1-(1-naphthyl)methyltriphenylphosphonium tetrafluoroborate (**1y**); 161.9 MHz/$CDCl_3$; δ (ppm).

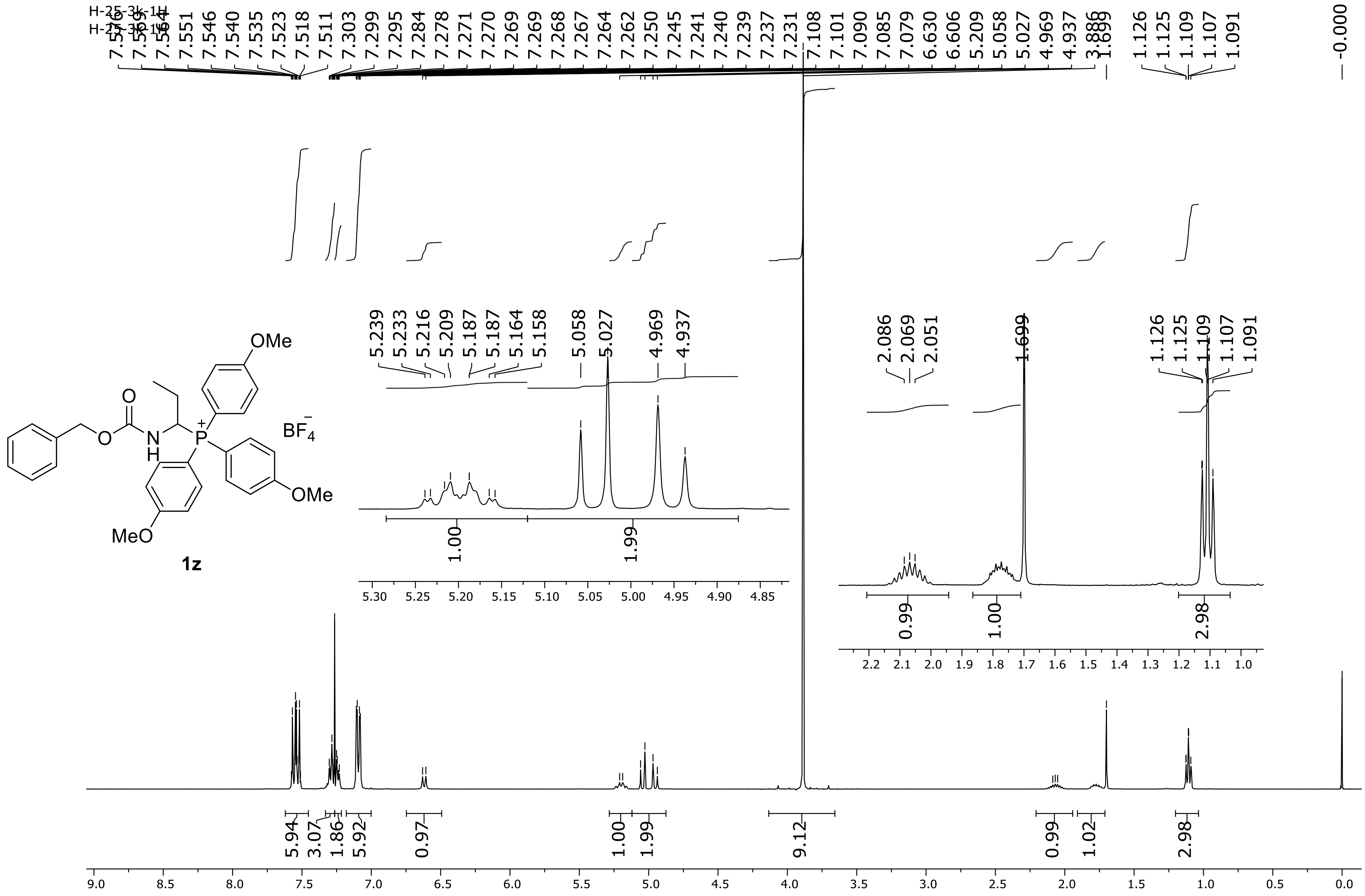

^{1}H NMR spectrum of 1-(*N*-benzyloxycarbonylamino)propyltris(4-methoxyphenyl)phosphonium tetrafluoroborate (**1z**); 400 MHz/$CDCl_3$/TMS; δ (ppm).

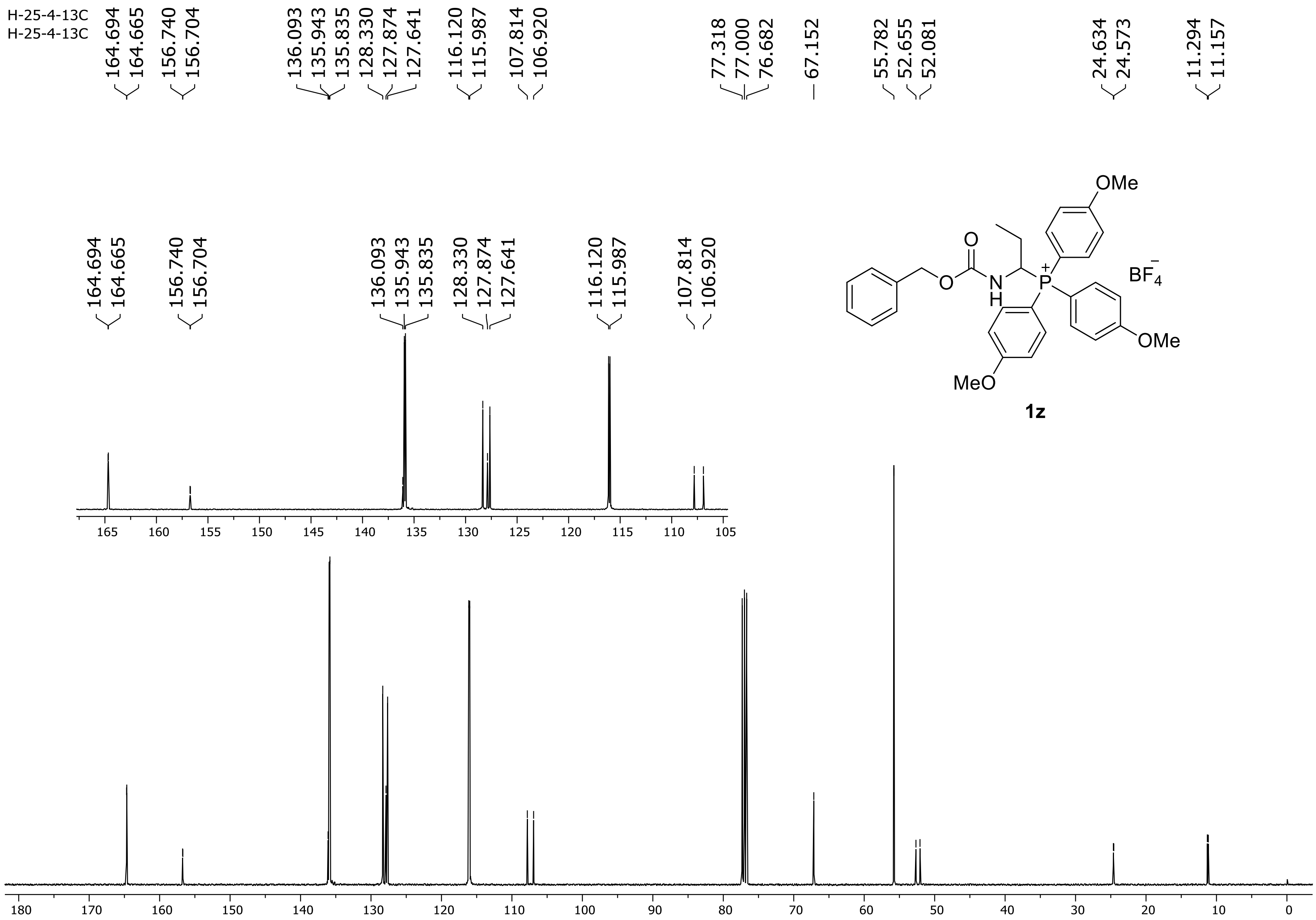

$^{13}C\{^1H\}$ NMR spectrum of 1-(*N*-benzyloxycarbonylamino)propyltris(4-methoxyphenyl)phosphonium tetrafluoroborate (**1z**); 100 MHz/$CDCl_3$/TMS; δ (ppm).

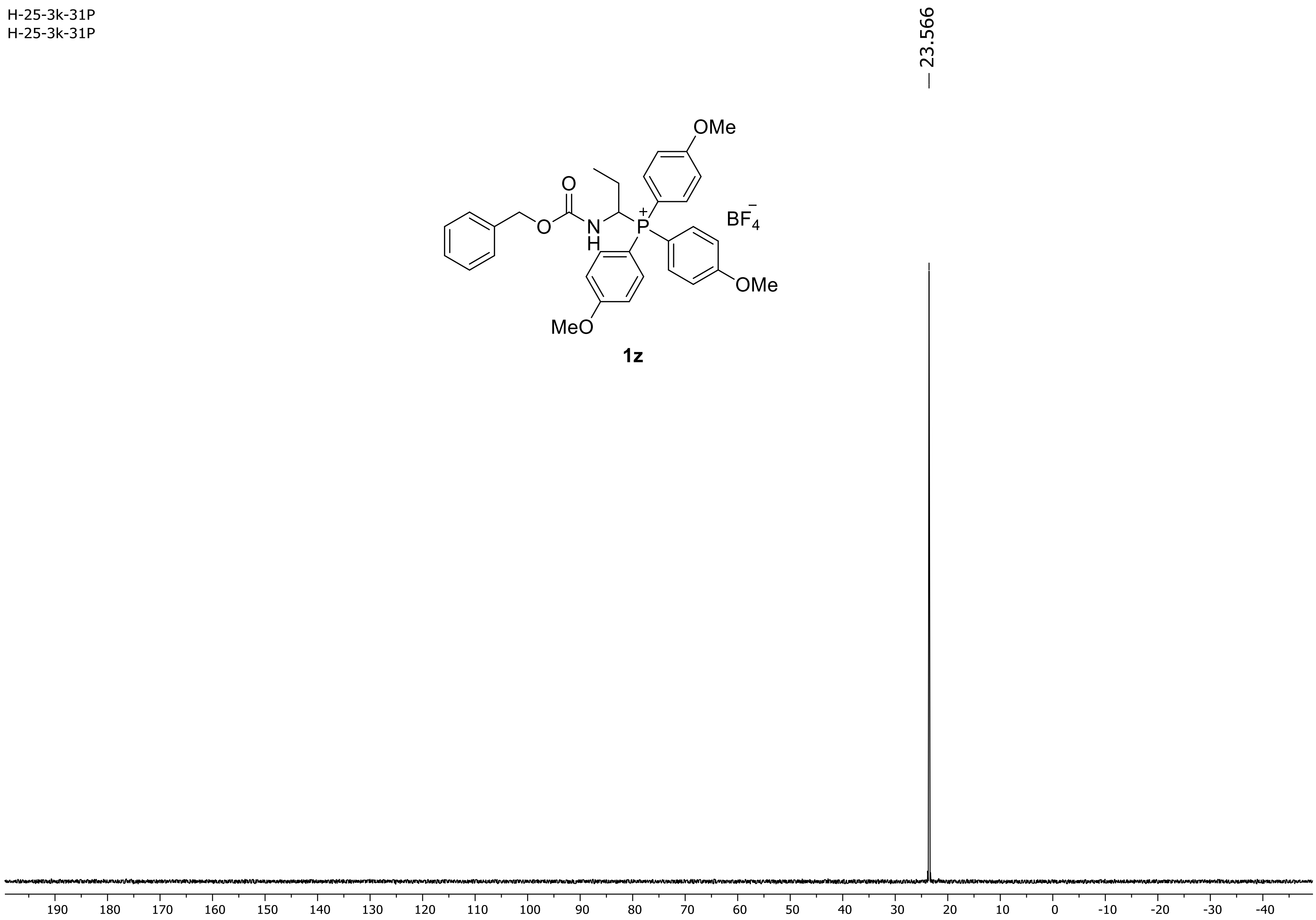

^{31}P NMR spectrum of 1-(*N*-benzyloxycarbonylamino)propyltris(4-methoxyphenyl)phosphonium tetrafluoroborate (**1z**); 161.9 MHz/$CDCl_3$; δ (ppm).

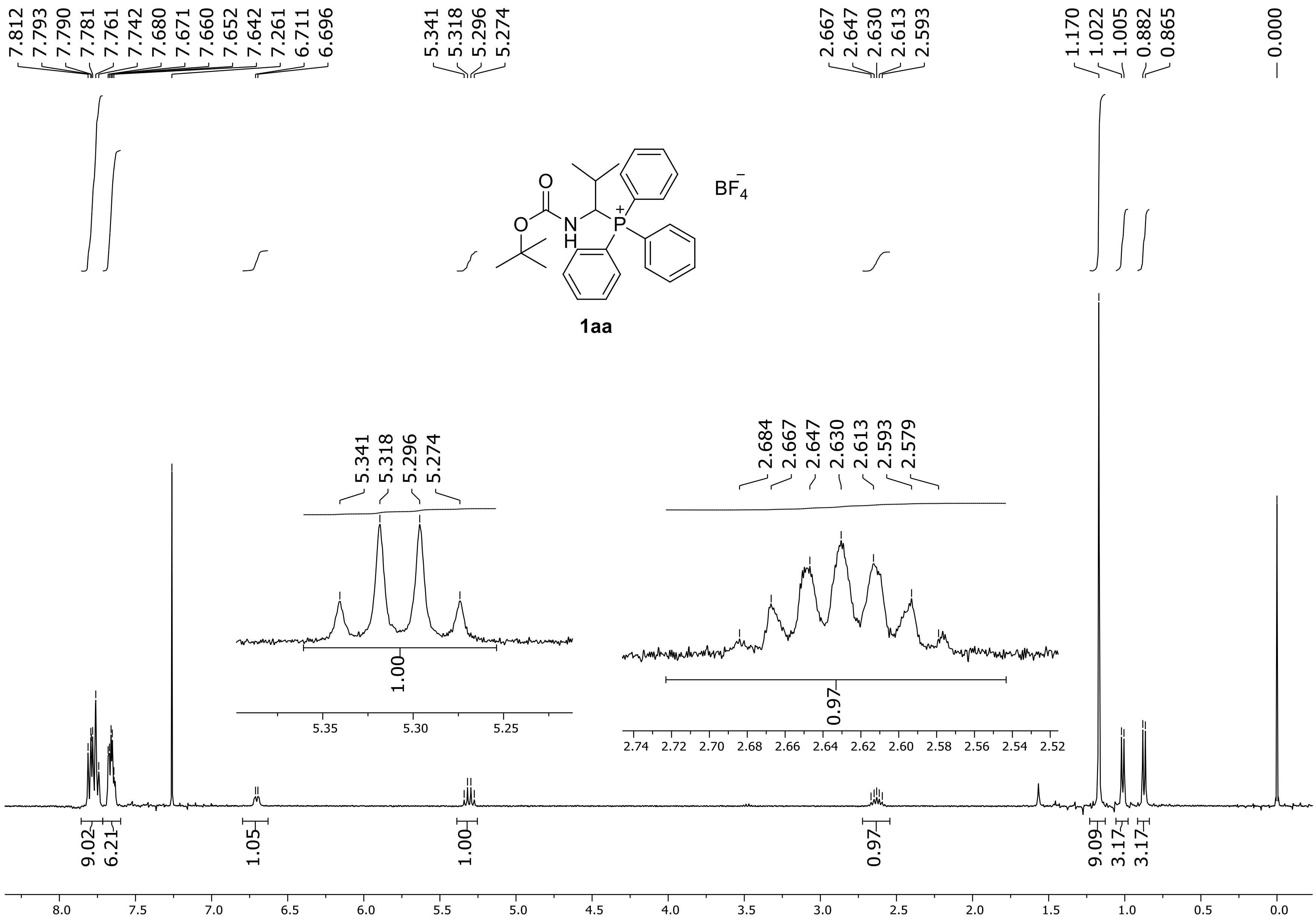

^{1}H NMR spectrum of 1-(*N*-tert-butoxycarbonylamino)-2-methylpropyltriphenylphosphonium tetrafluoroborate (**1aa**); 400 MHz/$CDCl_3$/TMS; δ (ppm).

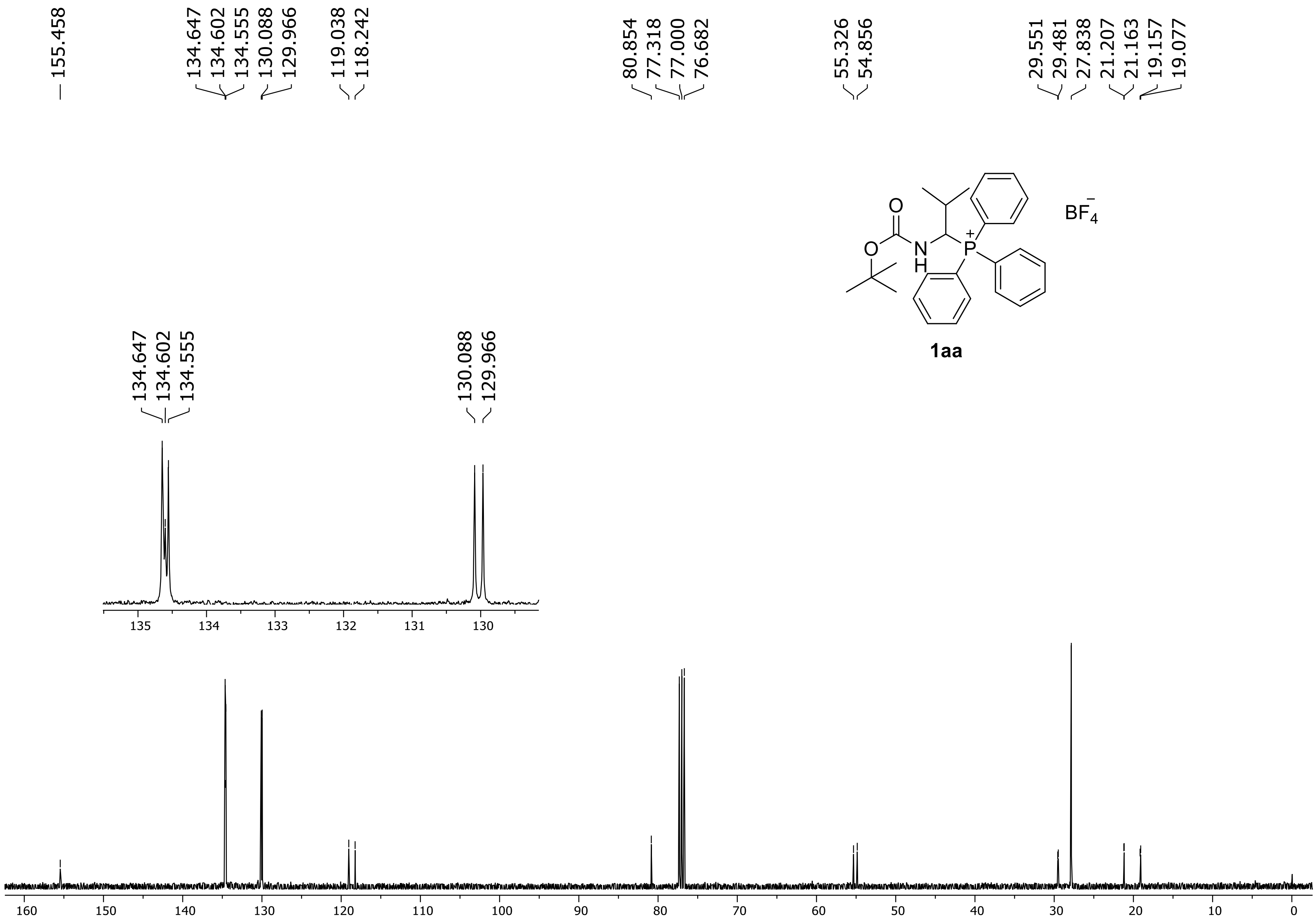

$^{13}C\{^{1}H\}$ NMR spectrum of 1-(*N*-tert-butoxycarbonylamino)-2-methylpropyltriphenylphosphonium tetrafluoroborate (**1aa**); 100 MHz/$CDCl_3$/TMS; δ (ppm).

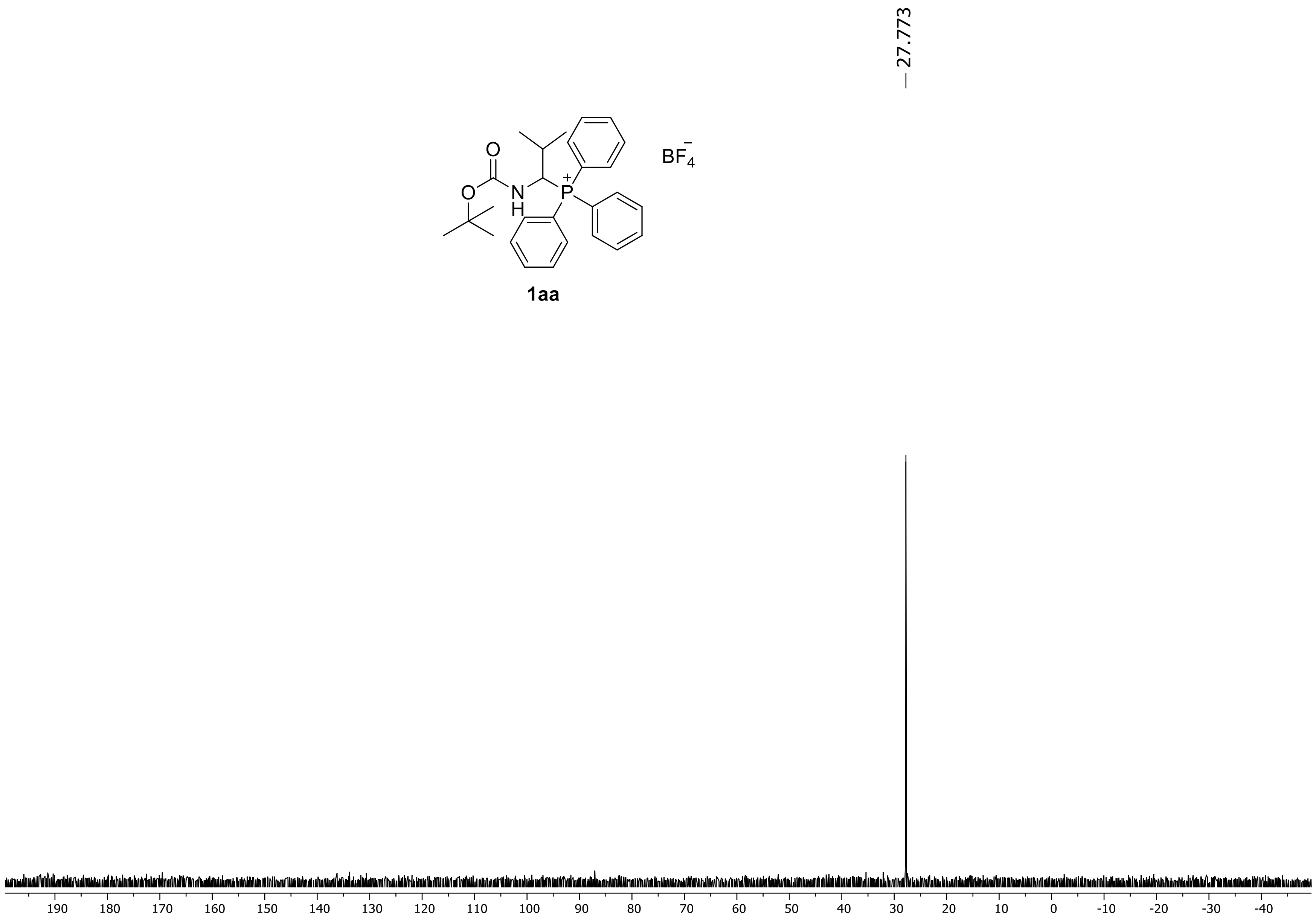

^{31}P NMR spectrum of 1-(*N*-tert-butoxycarbonylamino)-2-methylpropyltriphenylphosphonium tetrafluoroborate (**1aa**); 161.9 MHz/$CDCl_3$; δ (ppm).

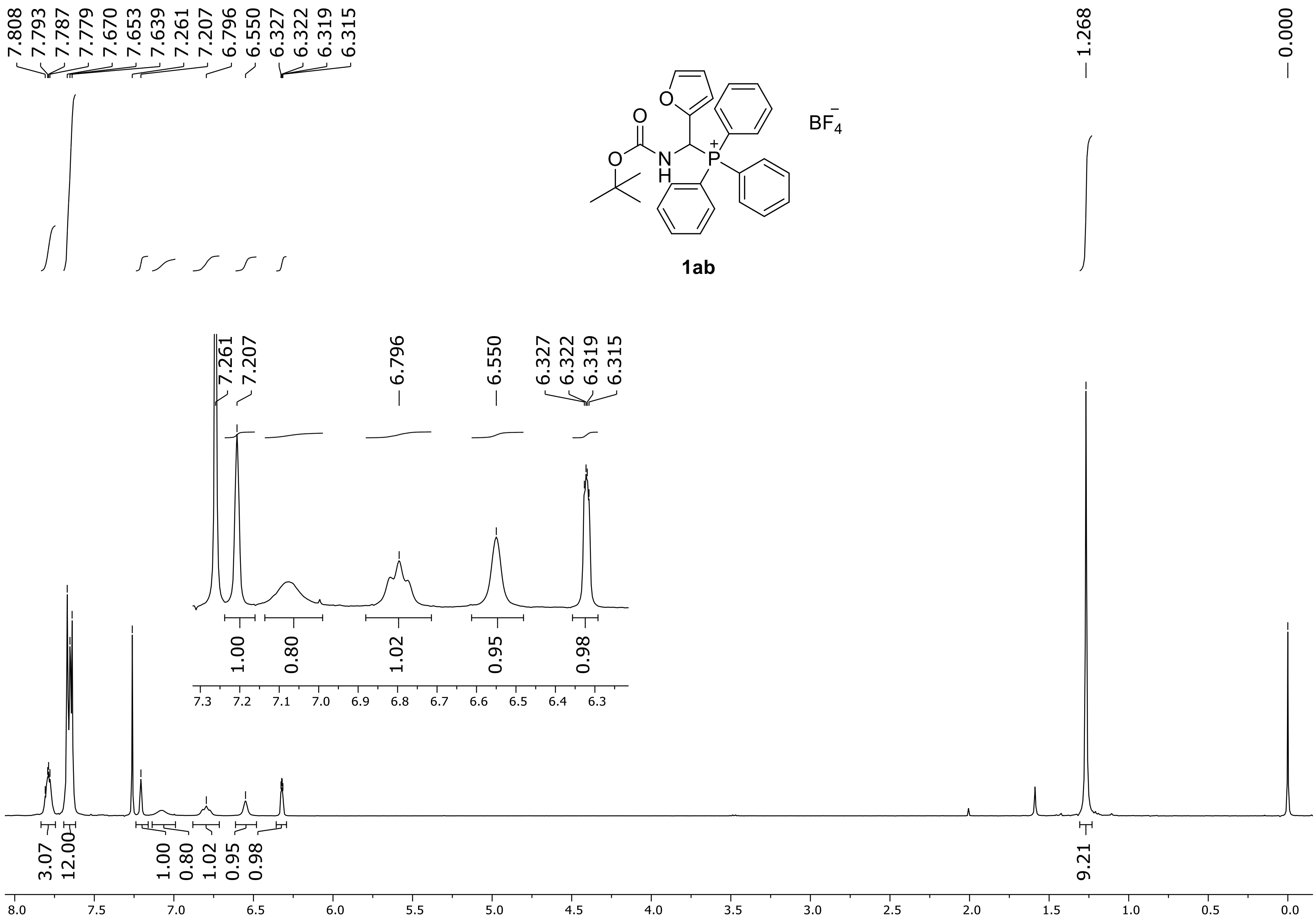

^{1}H NMR spectrum of 1-(*N*-tert-butoxycarbonylamino)-1-(2-furyl)methyltriphenylphosphonium tetrafluoroborate (**1ab**); 400 MHz/$CDCl_3$/TMS; δ (ppm).

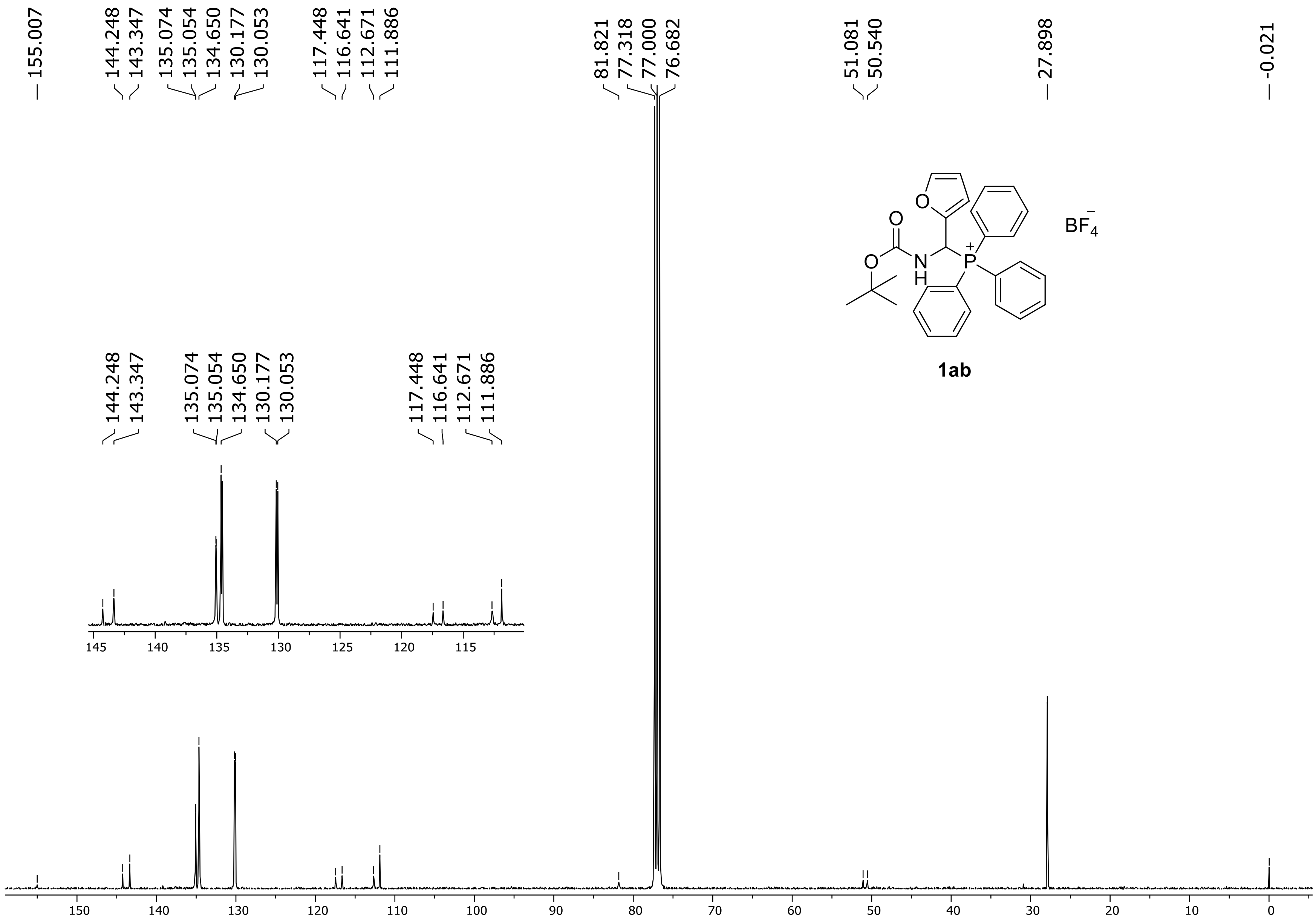

$^{13}C\{^{1}H\}$ NMR spectrum of 1-(*N*-tert-butoxycarbonylamino)-1-(2-furyl)methyltriphenylphosphonium tetrafluoroborate (**1ab**); 100 MHz/$CDCl_3$/TMS; δ (ppm).

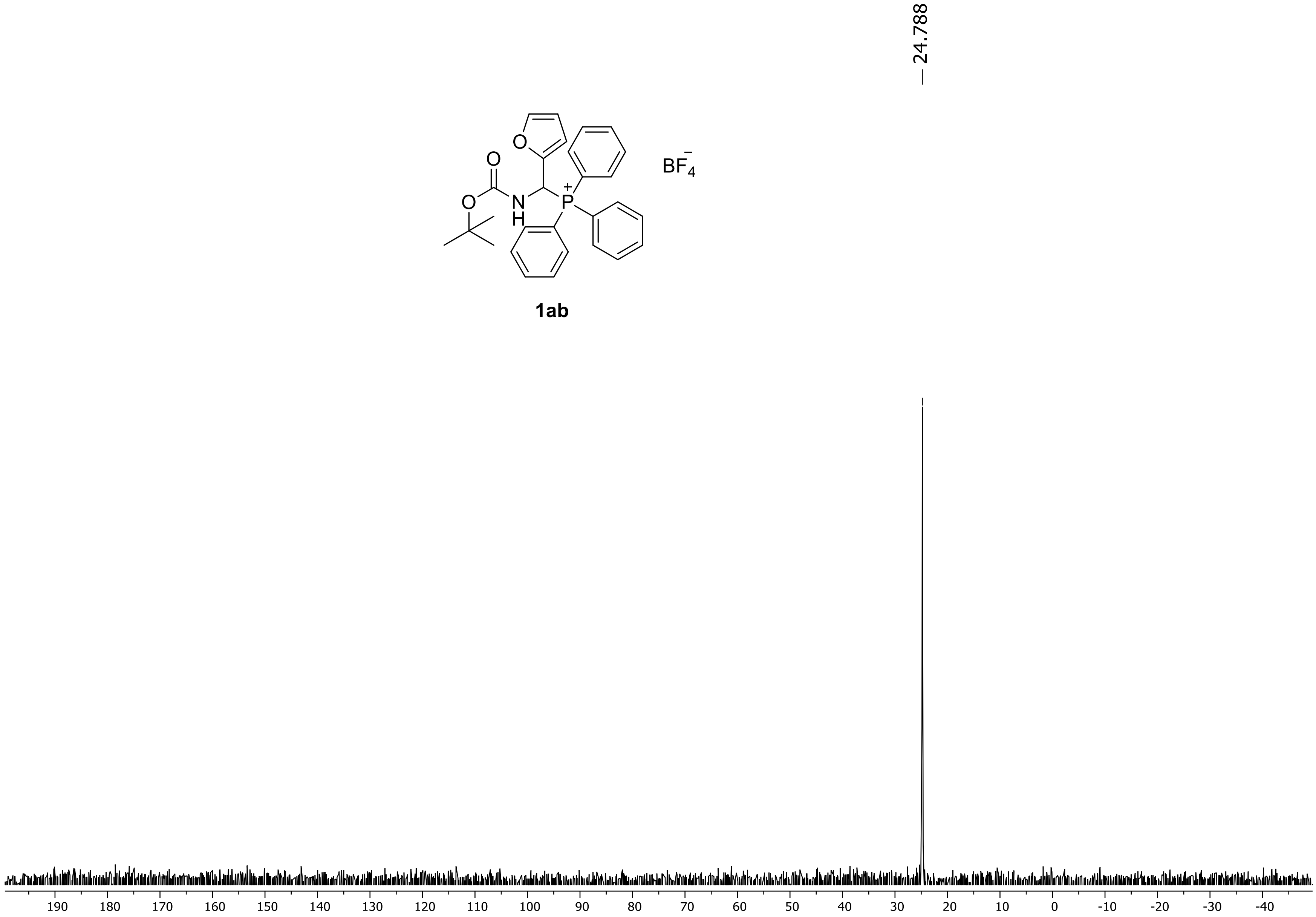

^{31}P NMR spectrum of 1-(*N*-tert-butoxycarbonylamino)-1-(2-furyl)methyltriphenylphosphonium tetrafluoroborate (**1ab**); 161.9 MHz/$CDCl_3$; δ (ppm).

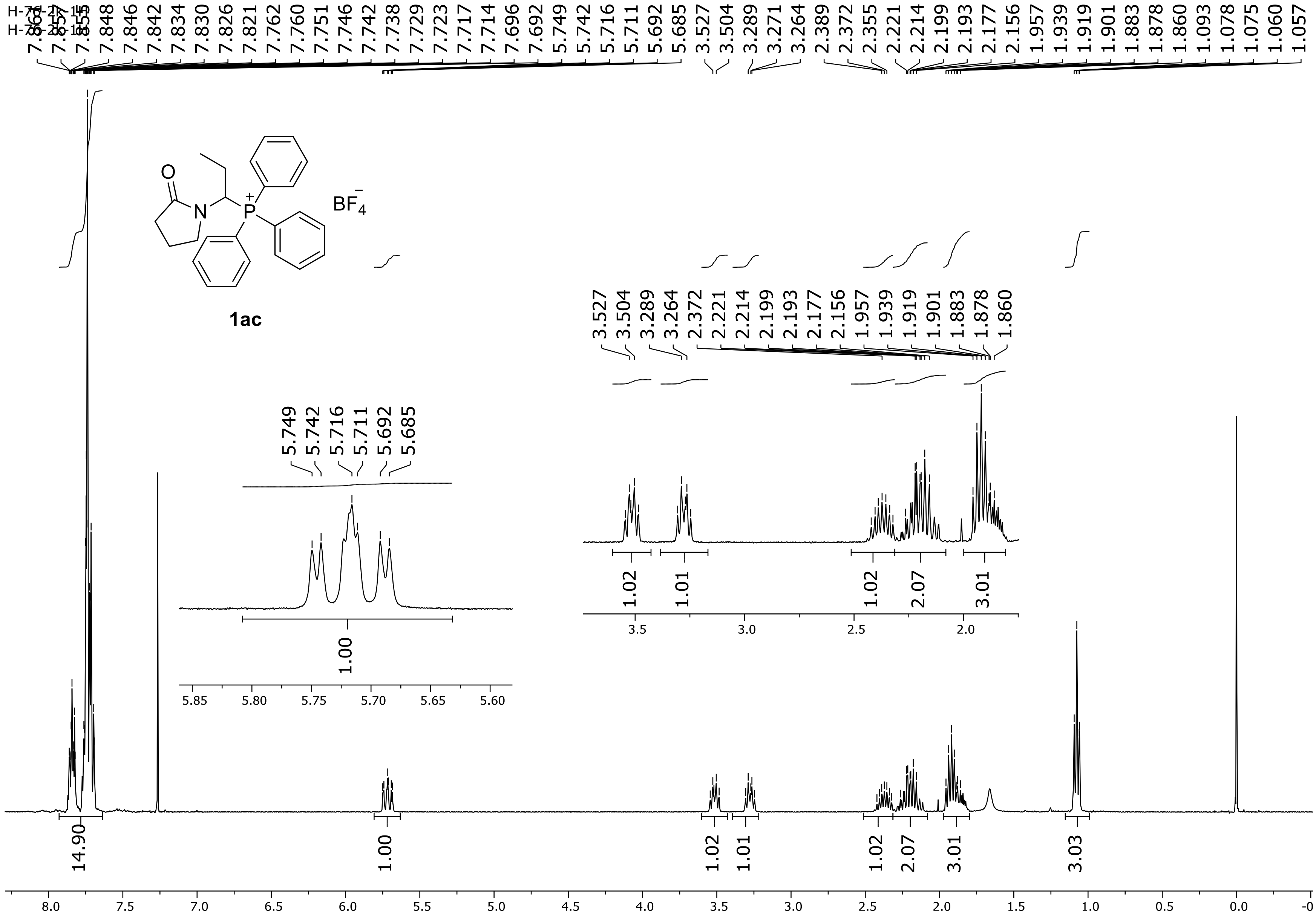

^{1}H NMR spectrum of 1-(2-oxopyrrolidin-1-yl)propyltriphenylphosphonium tetrafluoroborate (**1ac**); 400 MHz/$CDCl_3$/TMS; *δ* (ppm).

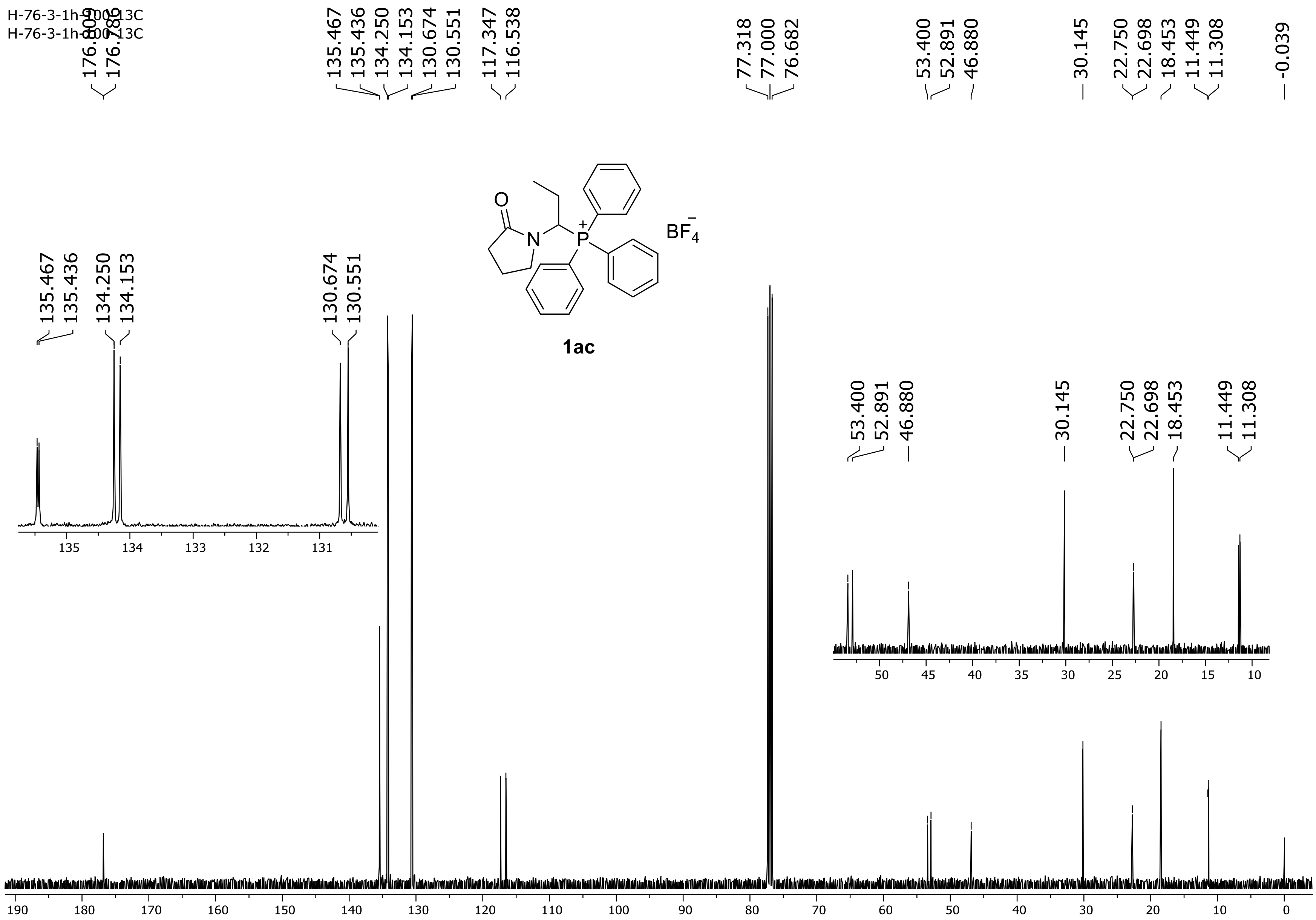

$^{13}C\{^1H\}$ NMR spectrum of 1-(2-oxopyrrolidin-1-yl)propyltriphenylphosphonium tetrafluoroborate (**1ac**); 100 MHz/$CDCl_3$/TMS; δ (ppm).

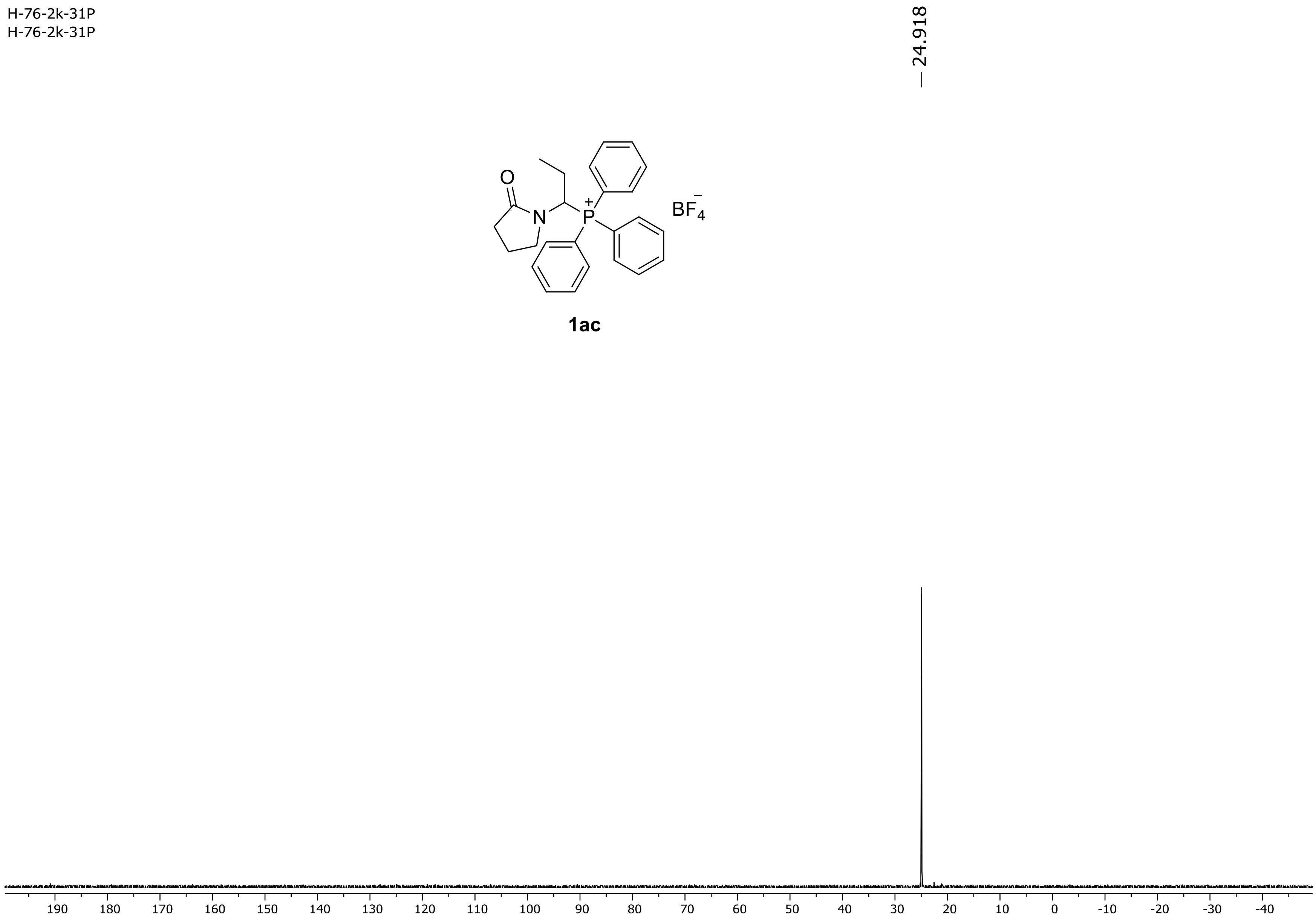

^{31}P NMR spectrum of 1-(2-oxopyrrolidin-1-yl)propyltriphenylphosphonium tetrafluoroborate (**1ac**); 161.9 MHz/$CDCl_3$; δ (ppm).

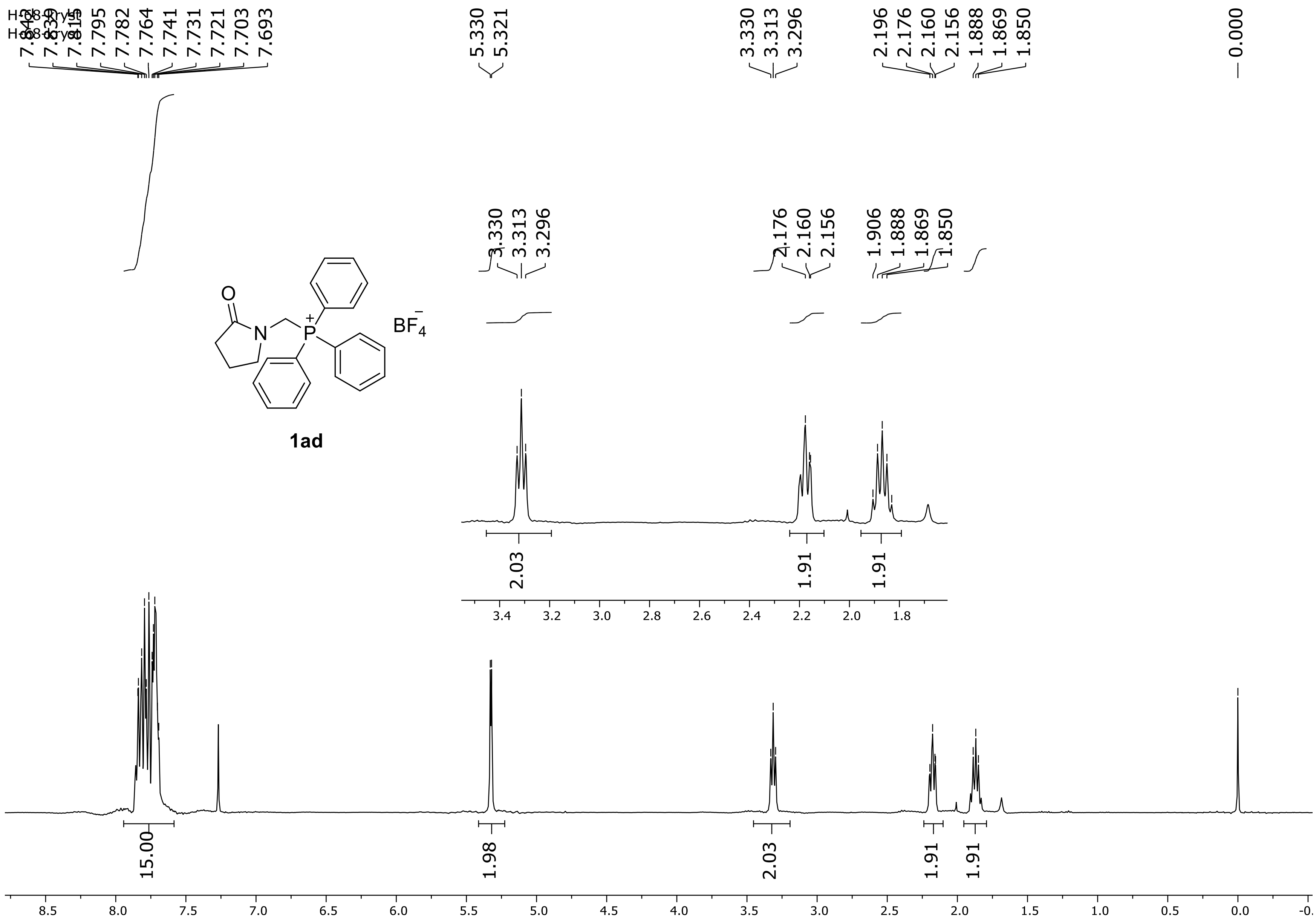

^{1}H NMR spectrum of 1-(2-oxopyrrolidin-1-yl)methyltriphenylphosphonium tetrafluoroborate (**1ad**); 400 MHz/CDCl$_3$/TMS; δ (ppm).

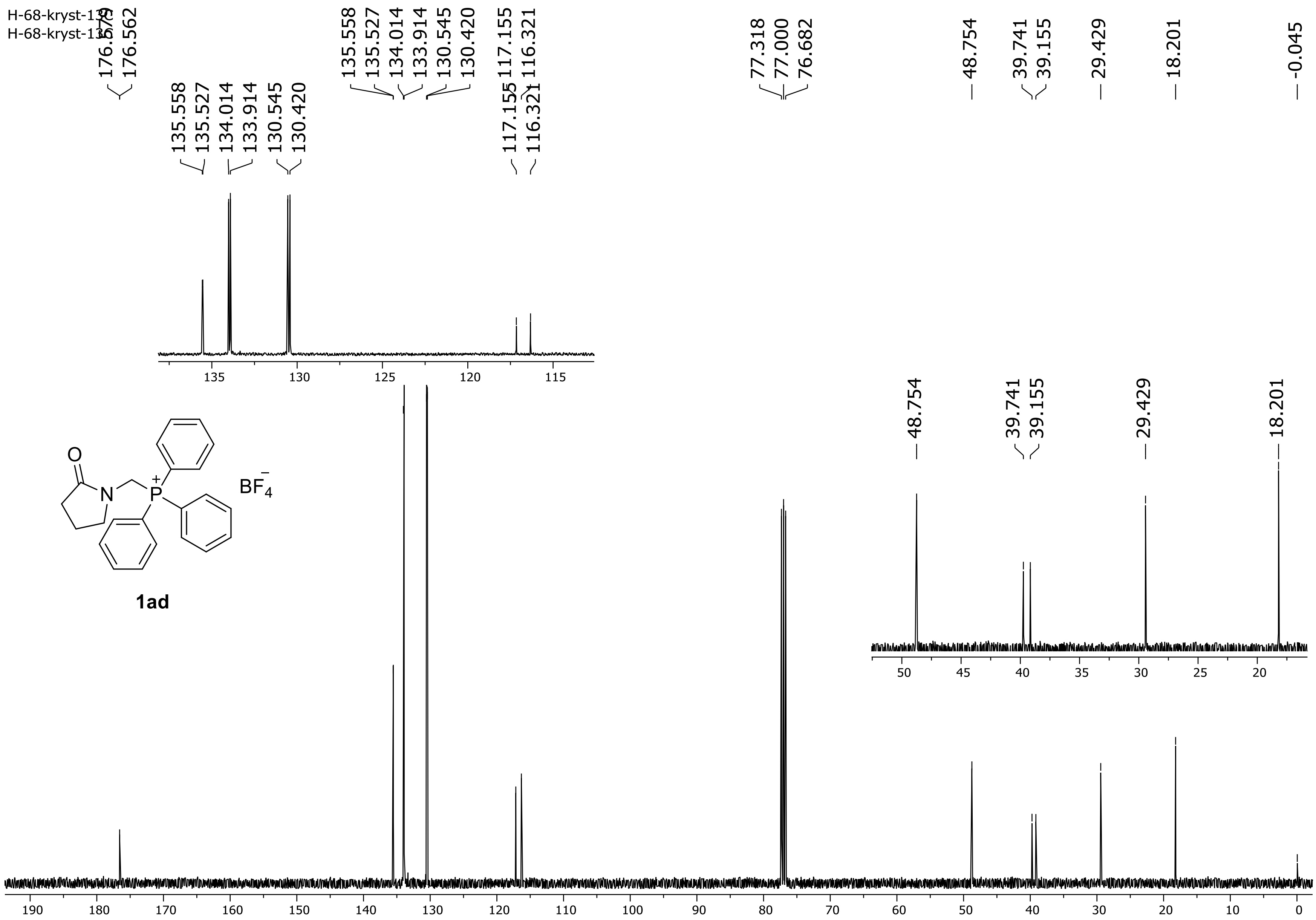

$^{13}C\{^1H\}$ NMR spectrum of 1-(2-oxopyrrolidin-1-yl)methyltriphenylphosphonium tetrafluoroborate (**1ad**); 100 MHz/$CDCl_3$/TMS; *δ* (ppm).

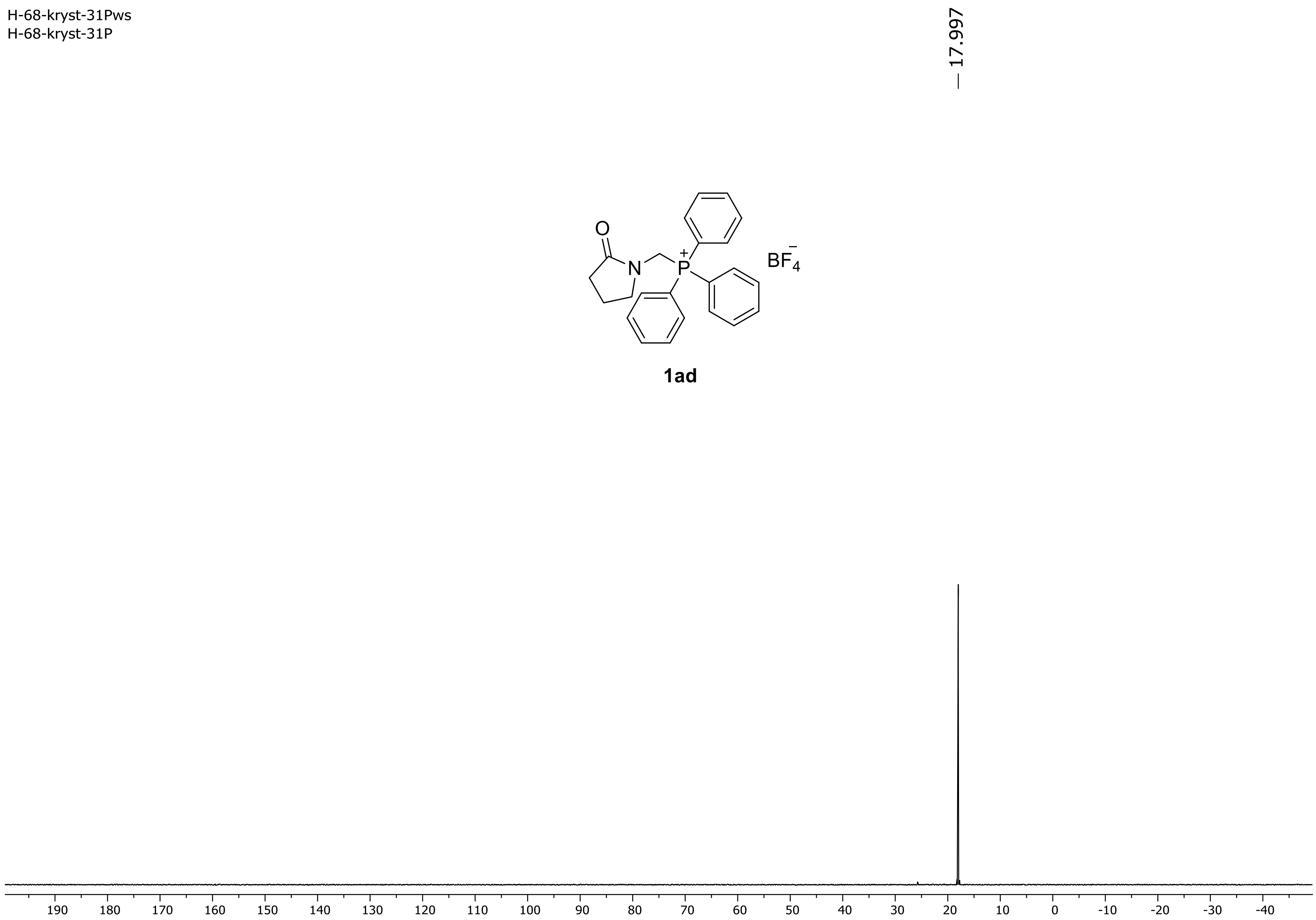

^{31}P NMR spectrum of 1-(2-oxopyrrolidin-1-yl)methyltriphenylphosphonium tetrafluoroborate (**1ad**); 161.9 MHz/CDCl$_3$; δ (ppm).

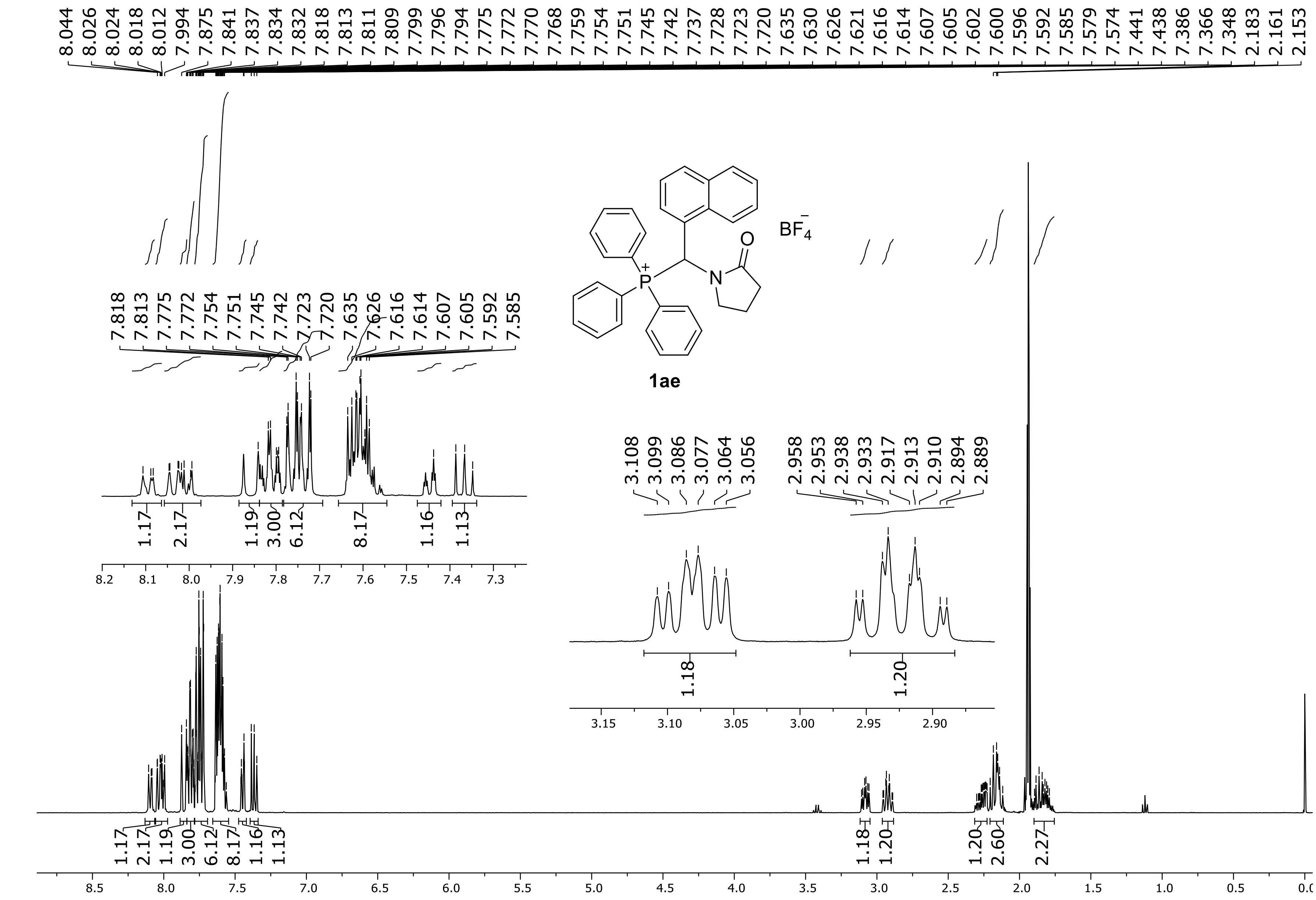

^{1}H NMR spectrum of 1-(2-oxopyrrolidin-1-yl)-1-(1-naphthyl)methyltriphenylphosphonium tetrafluoroborate (**1ae**); 400 MHz/CD_3CN/TMS; δ (ppm).

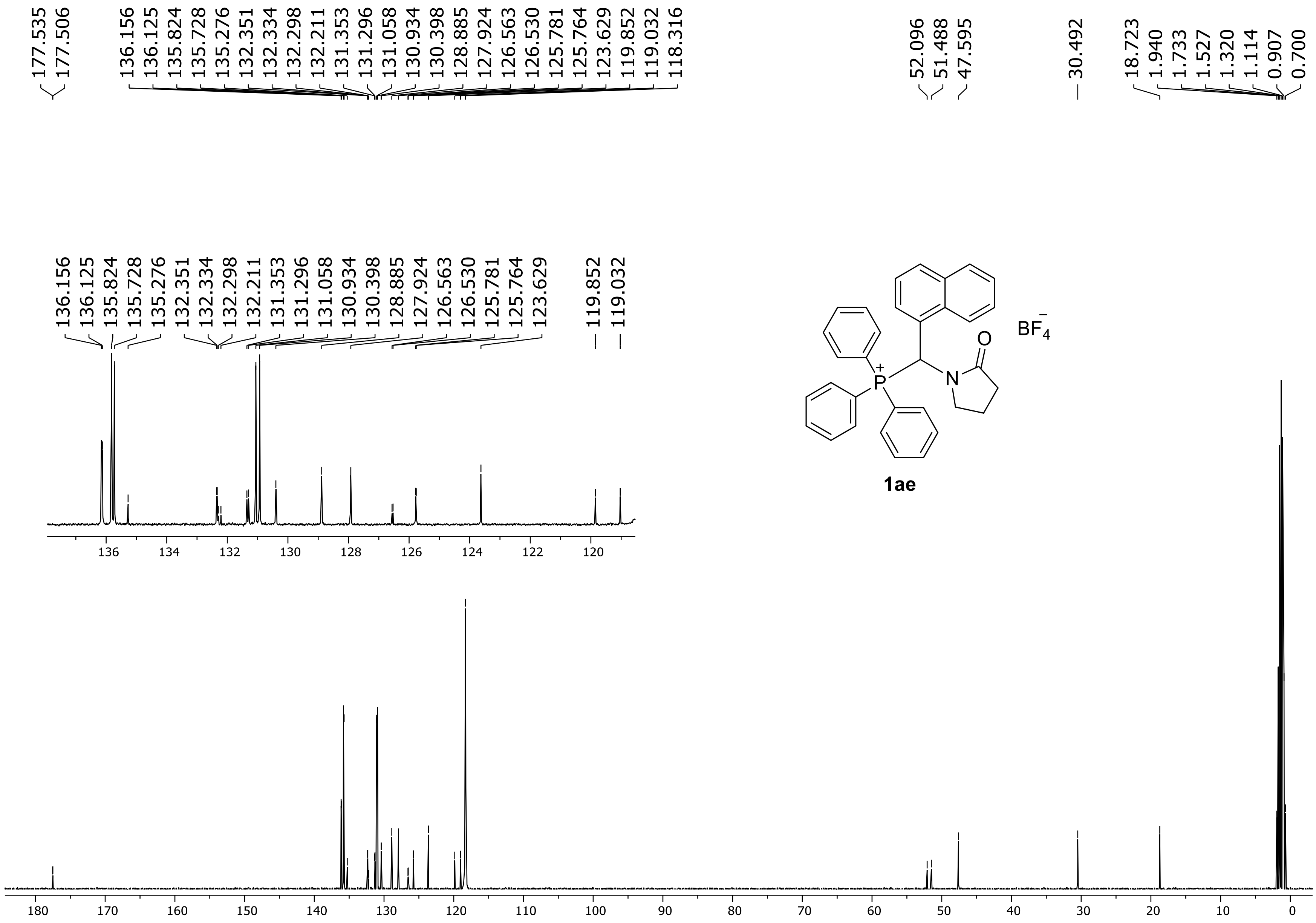

$^{13}C\{^{1}H\}$ NMR spectrum of 1-(2-oxopyrrolidin-1-yl)-1-(1-naphthyl)methyltriphenylphosphonium tetrafluoroborate (**1ae**); 100 MHz/CD_3CN/TMS; δ (ppm).

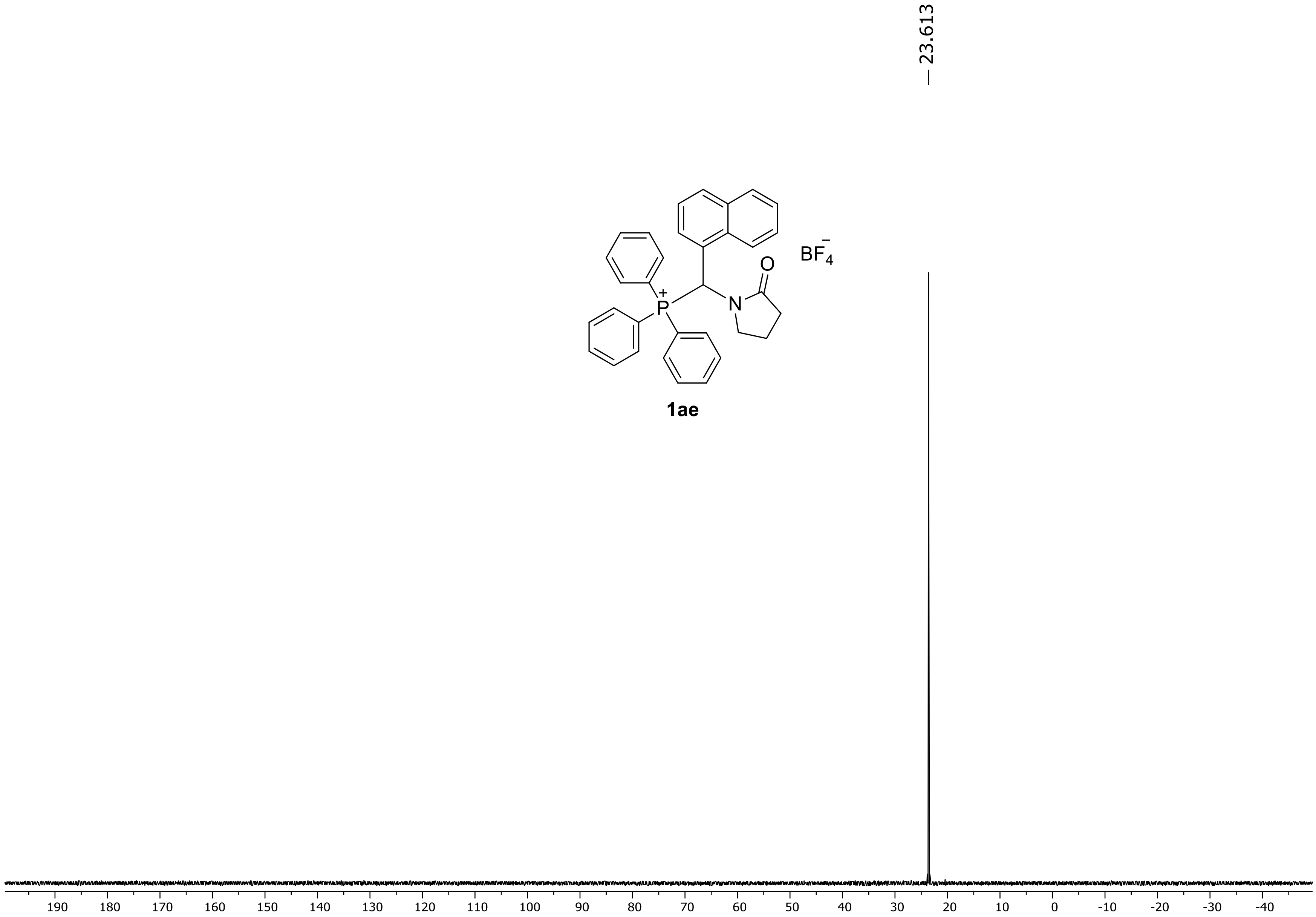

^{31}P NMR spectrum of 1-(2-oxopyrrolidin-1-yl)-1-(1-naphthyl)methyltriphenylphosphonium tetrafluoroborate (**1ae**); 161.9 MHz/CD_3CN; δ (ppm).

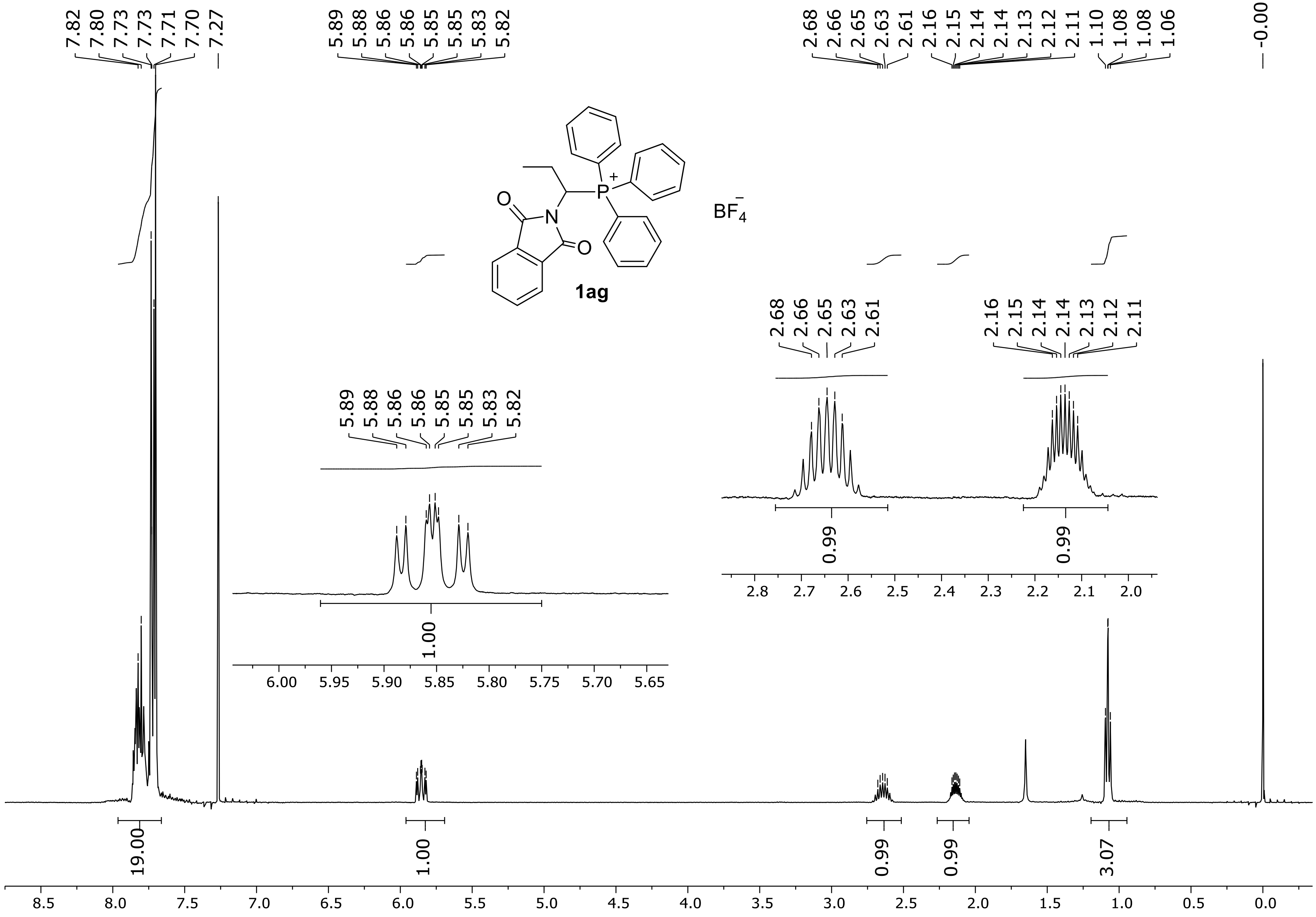

^{1}H NMR spectrum of 1-(*N*-phthalimido)propyltriphenylphosphonium tetrafluoroborate (**1ag**); 400 MHz/CDCl₃/TMS; δ (ppm).

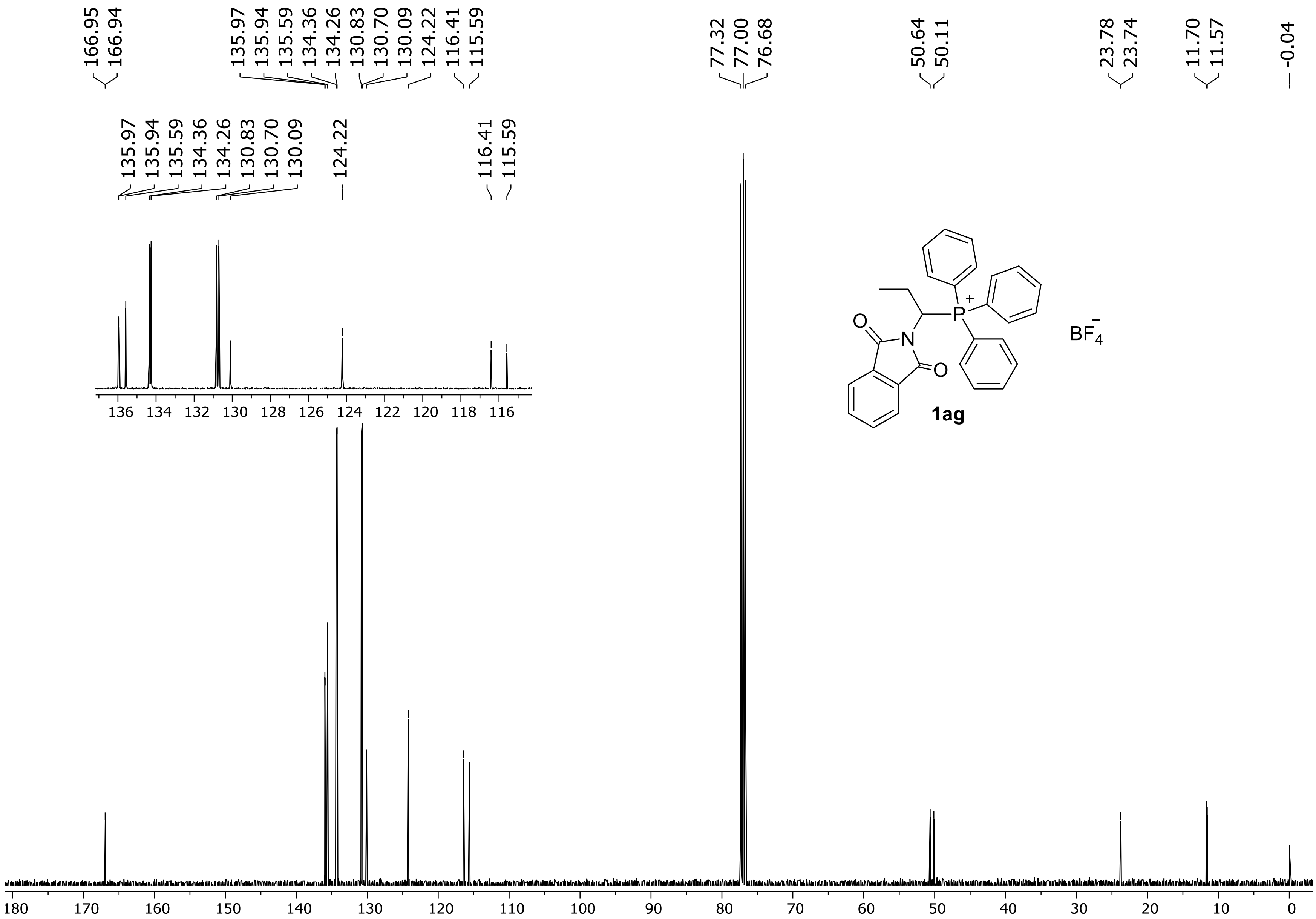

$^{13}C\{^1H\}$ NMR spectrum of 1-(*N*-phthalimido)propyltriphenylphosphonium tetrafluoroborate (**1ag**); 100 MHz/$CDCl_3$/TMS; δ (ppm).

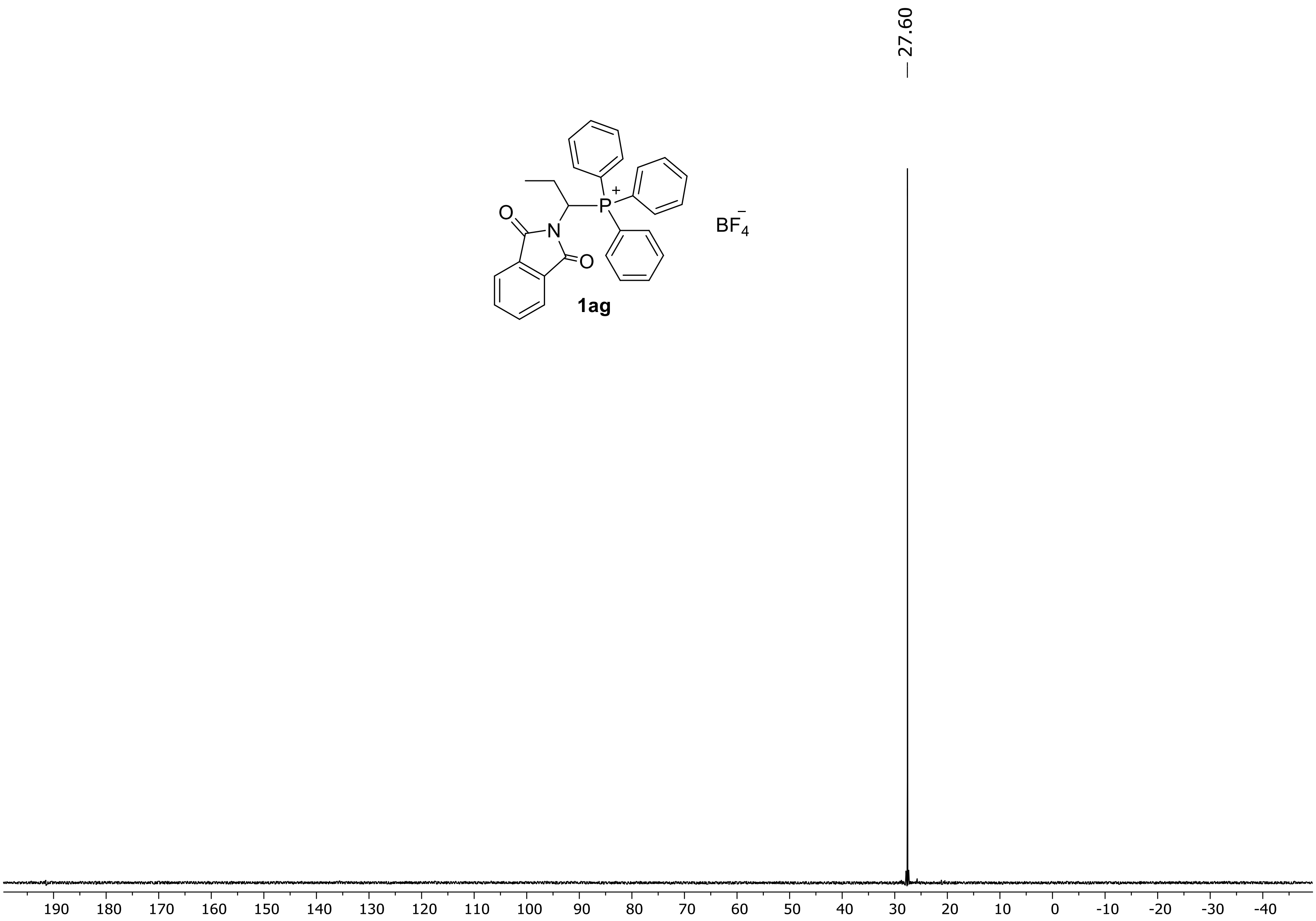

^{31}P NMR spectrum of 1-(*N*-phthalimido)propyltriphenylphosphonium tetrafluoroborate (**1ag**); 161.9 MHz/$CDCl_3$; δ (ppm).

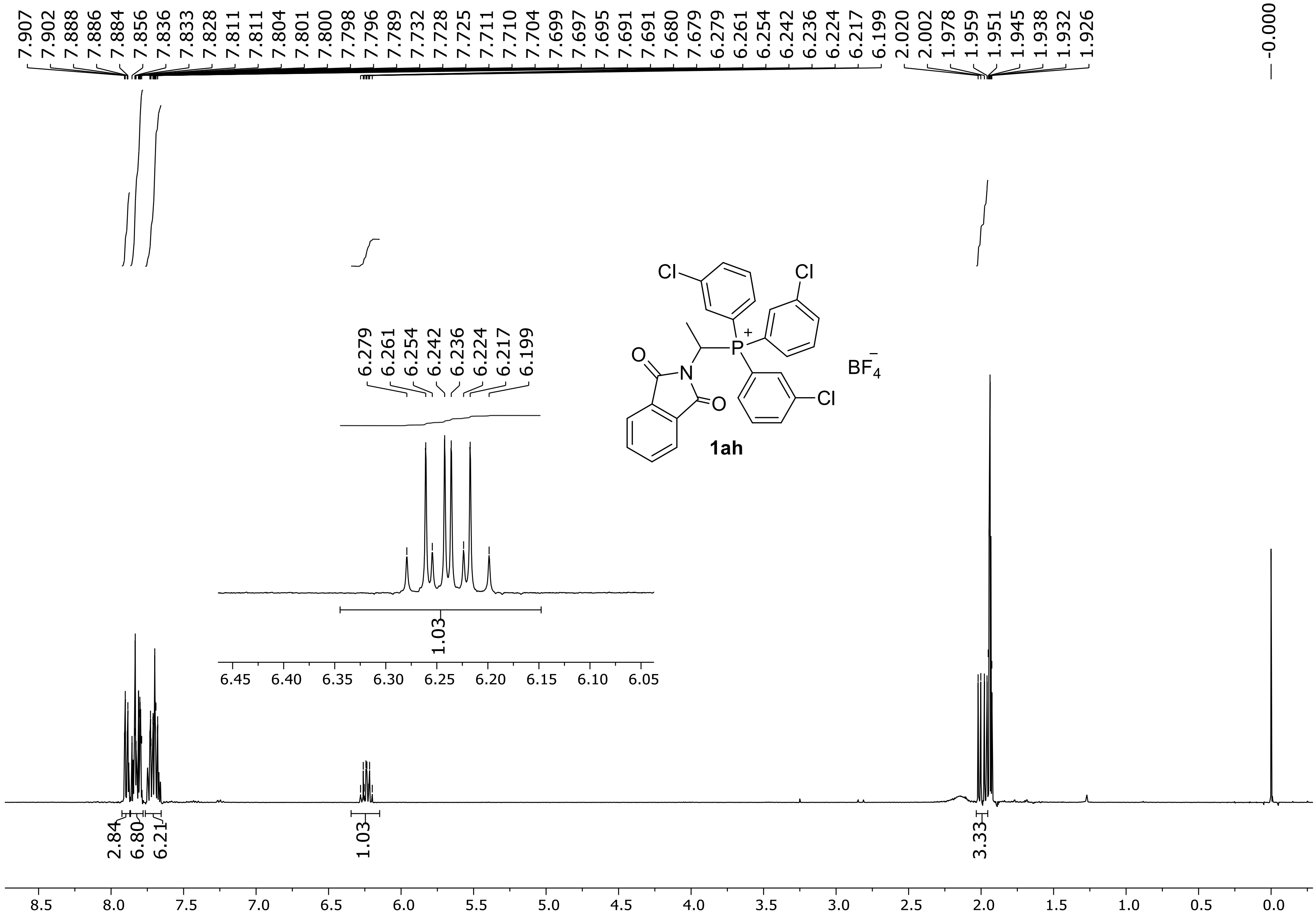

^{1}H NMR spectrum of 1-(*N*-phthalimido)ethyltris(3-chlorophenyl)phosphonium tetrafluoroborate (**1ah**); 400 MHz/CD_3CN/TMS; δ (ppm).

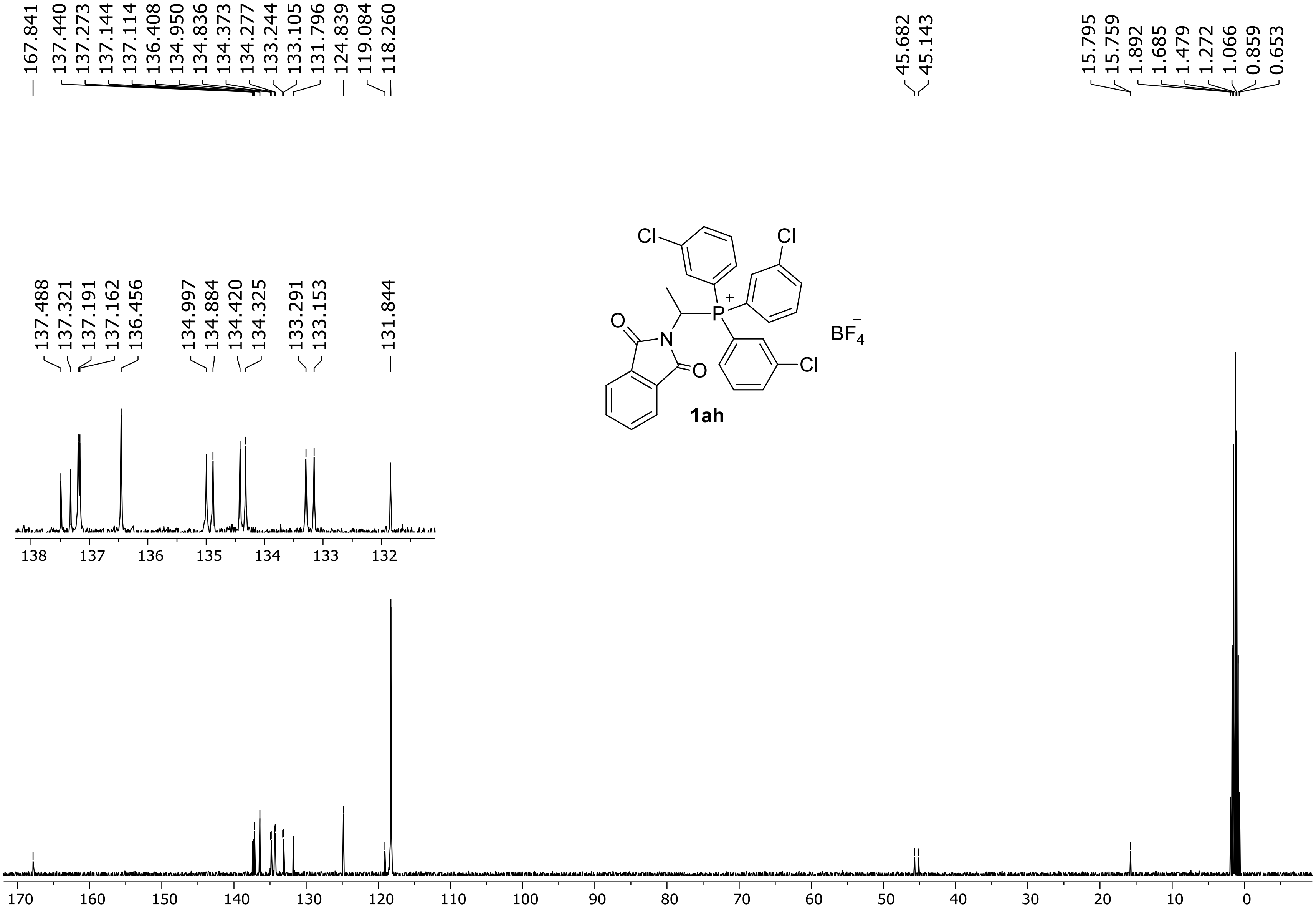

$^{13}C\{^{1}H\}$ NMR spectrum of 1-(*N*-phthalimido)ethyltris(3-chlorophenyl)phosphonium tetrafluoroborate (**1ah**); 100 MHz/CD_3CN/TMS; δ (ppm).

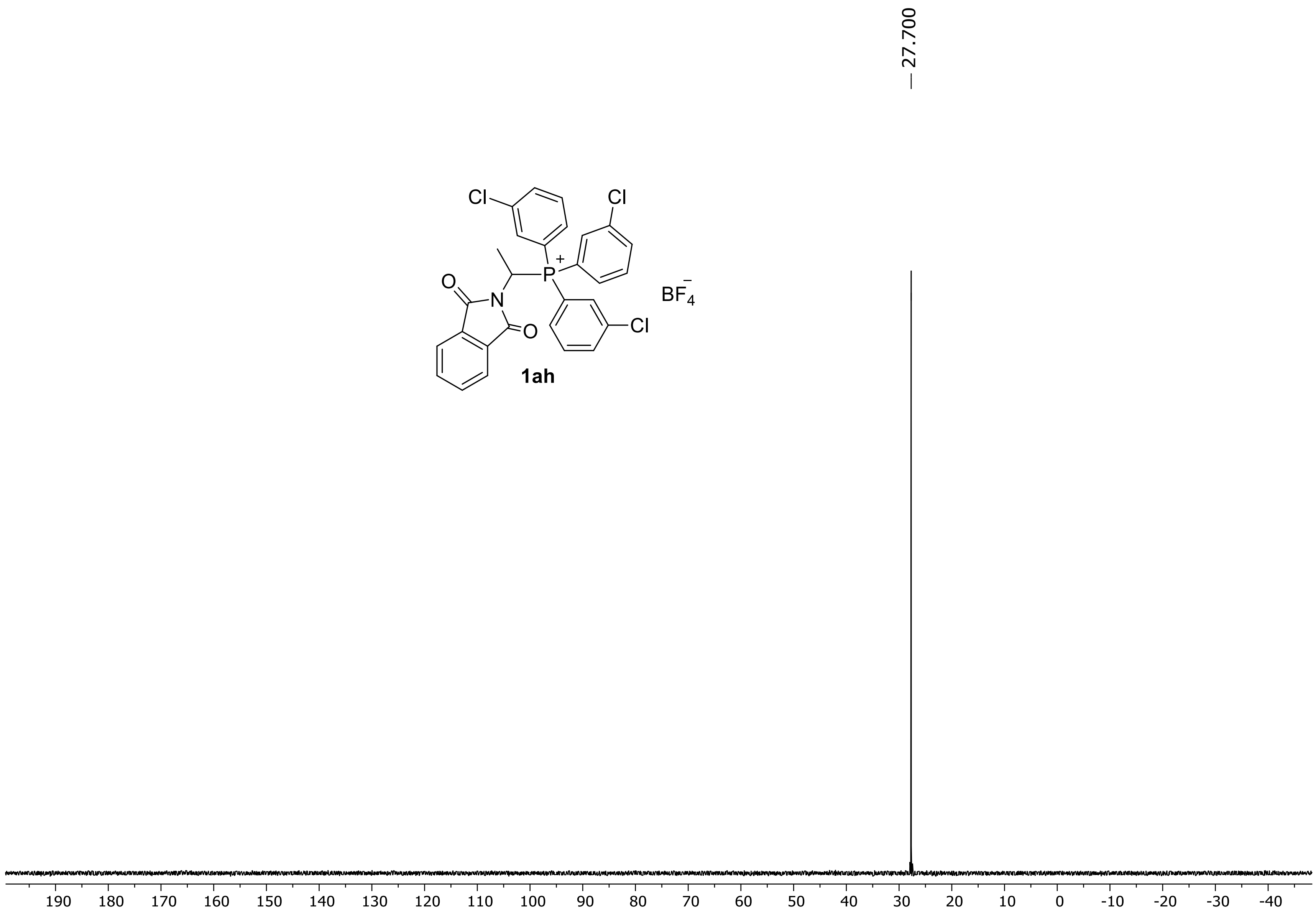

^{31}P NMR spectrum of 1-(*N*-phthalimido)ethyltris(3-chlorophenyl)phosphonium tetrafluoroborate (**1ah**); 161.9 MHz/CD_3CN; δ (ppm).

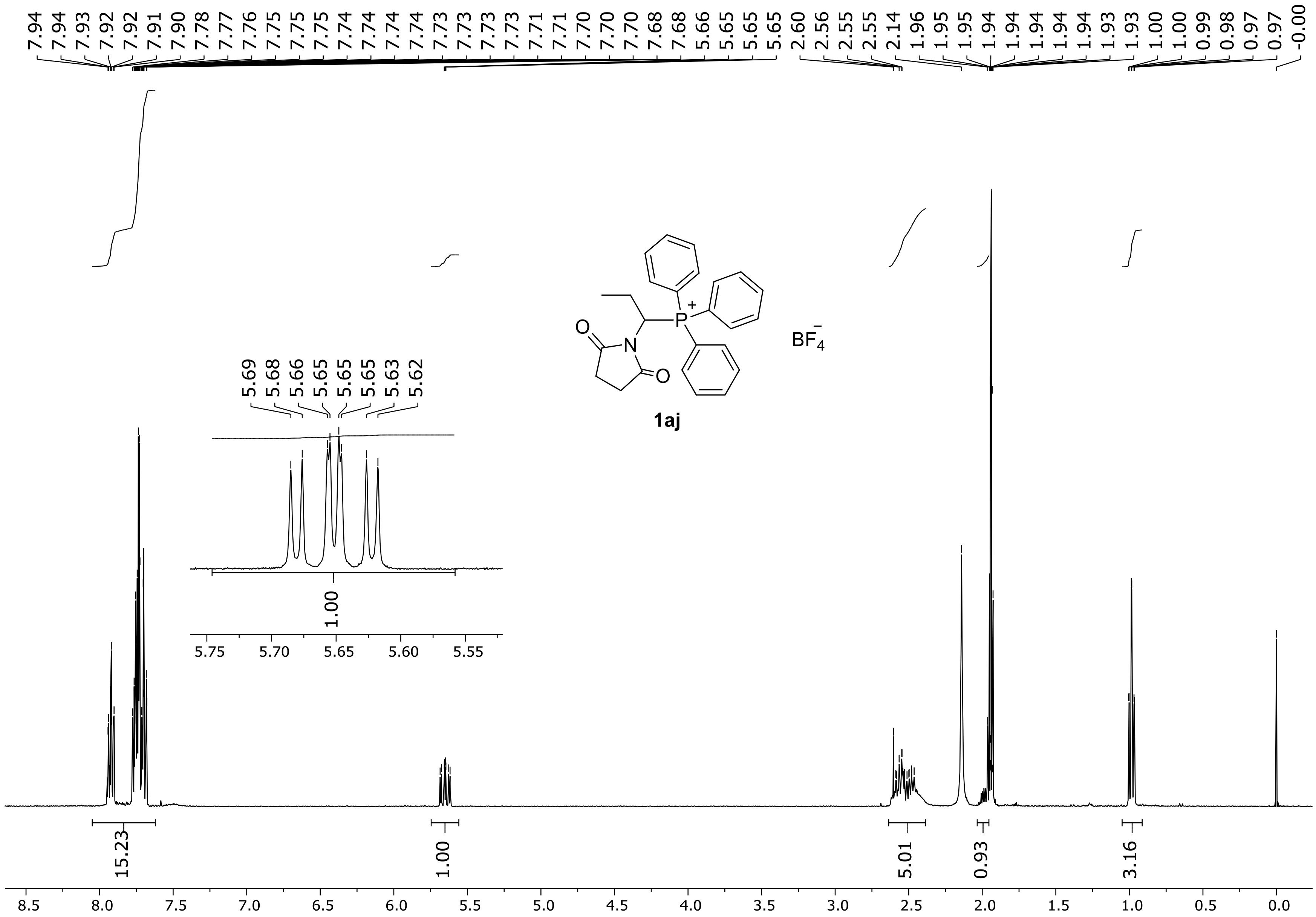

^{1}H NMR spectrum of 1-(*N*-succinimido)propyltriphenylphosphonium tetrafluoroborate (**1aj**); 400 MHz/CD_3CN/TMS; δ (ppm).

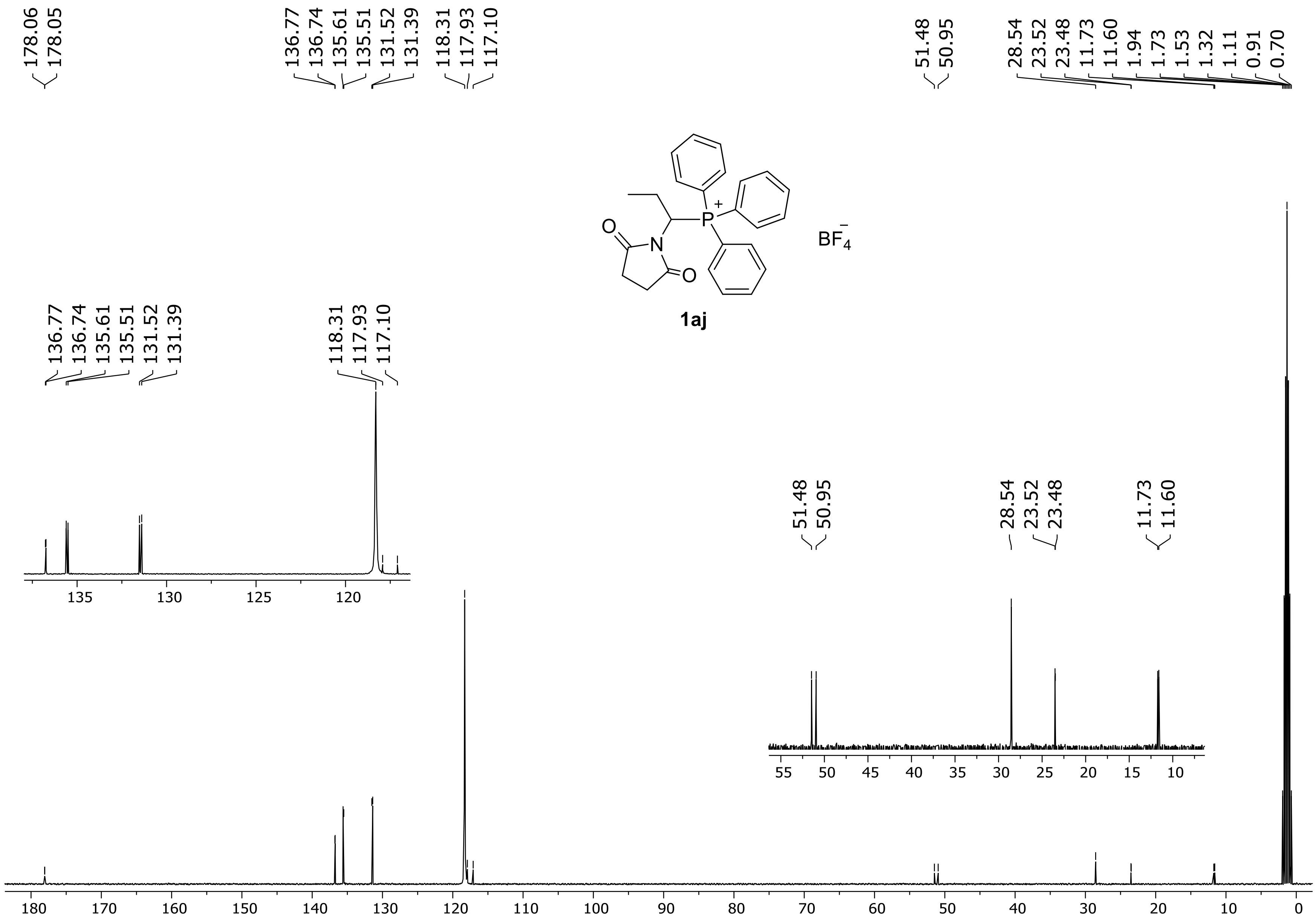

$^{13}C\{^{1}H\}$ NMR spectrum of 1-(*N*-succinimido)propyltriphenylphosphonium tetrafluoroborate (**1aj**); 100 MHz/CD_3CN/TMS; δ (ppm).

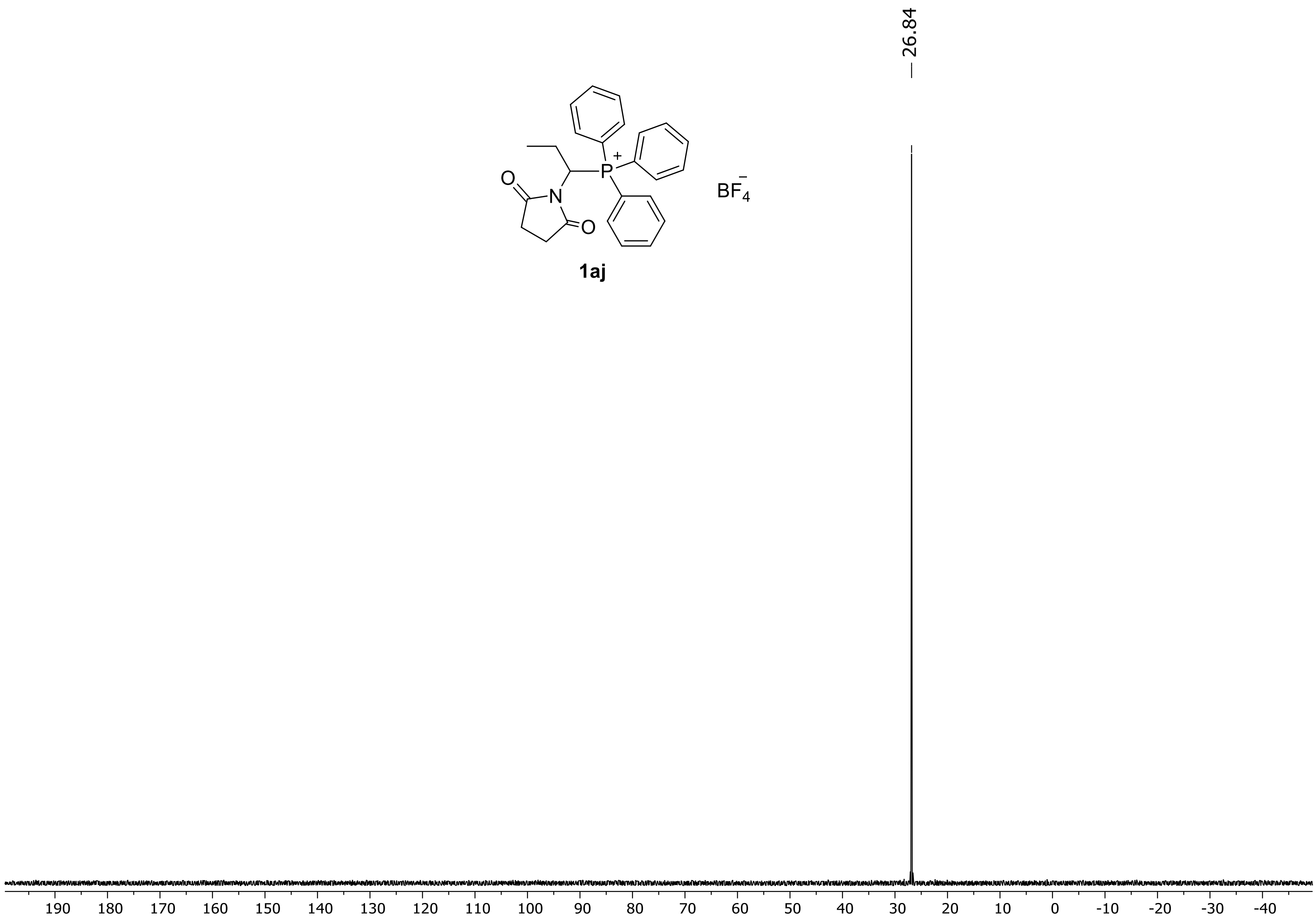

^{31}P NMR spectrum of 1-(*N*-succinimido)propyltriphenylphosphonium tetrafluoroborate (**1aj**); 161.9 MHz/CD_3CN; δ (ppm).

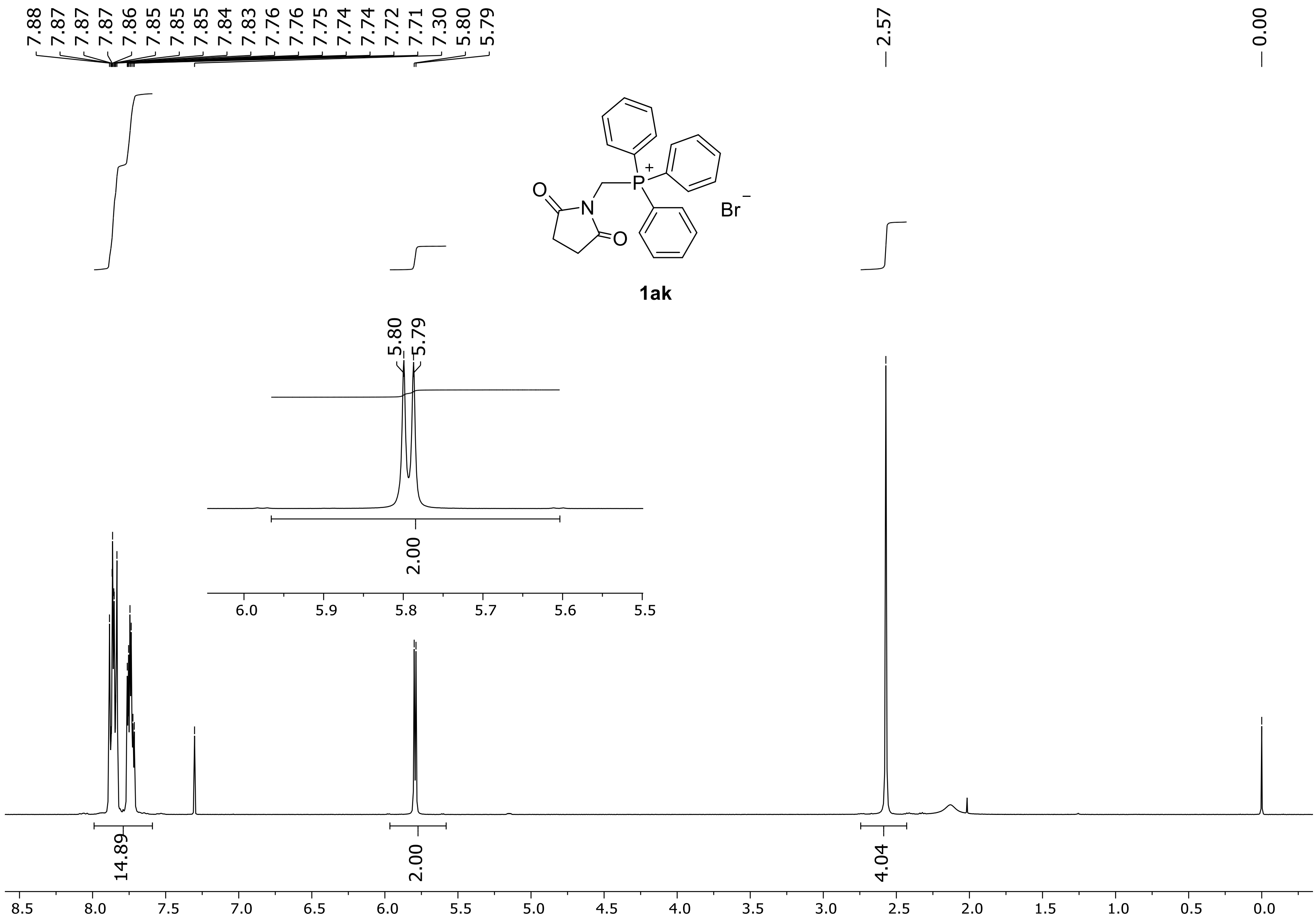

^{1}H NMR spectrum of 1-(*N*-succinimido)methyltriphenylphosphonium bromide (**1ak**); 400 MHz/$CDCl_3$/TMS; δ (ppm).

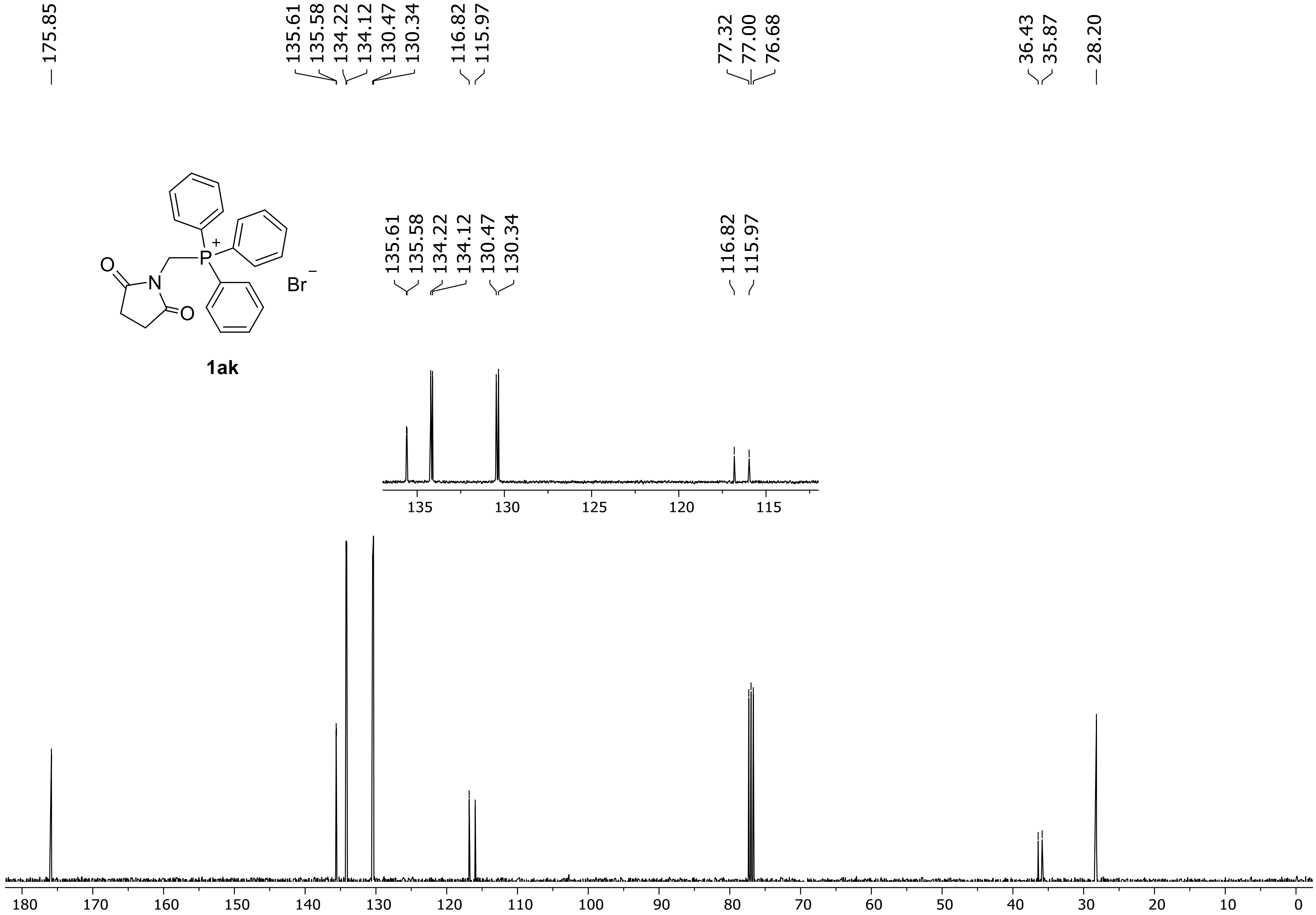

$^{13}C\{^1H\}$ NMR spectrum of 1-(*N*-succinimido)methyltriphenylphosphonium bromide (**1ak**); 100 MHz/$CDCl_3$/TMS; δ (ppm).

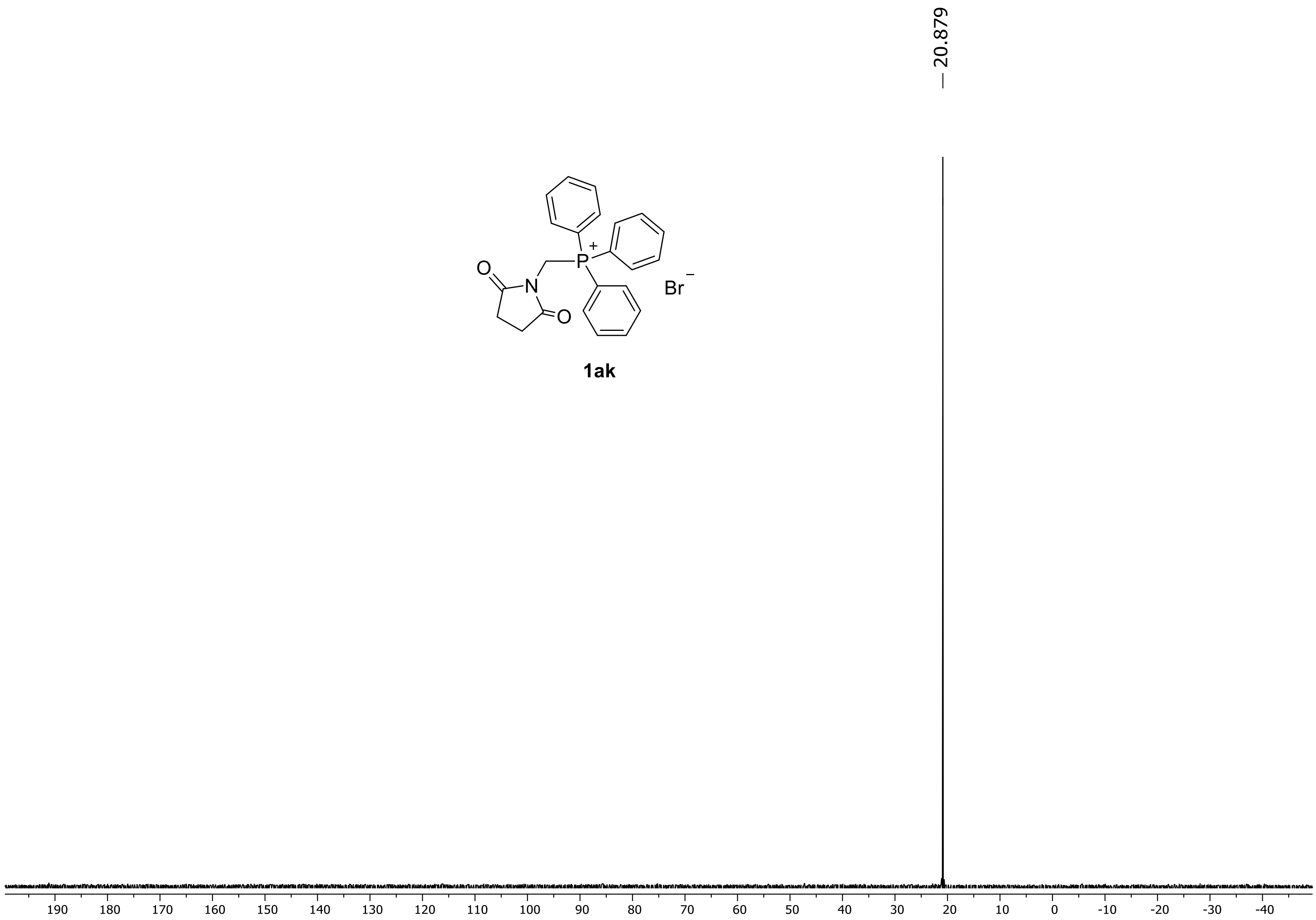

^{31}P NMR spectrum of 1-(*N*-succinimido)methyltriphenylphosphonium bromide (**1ak**); 161.9 MHz/$CDCl_3$; δ (ppm).

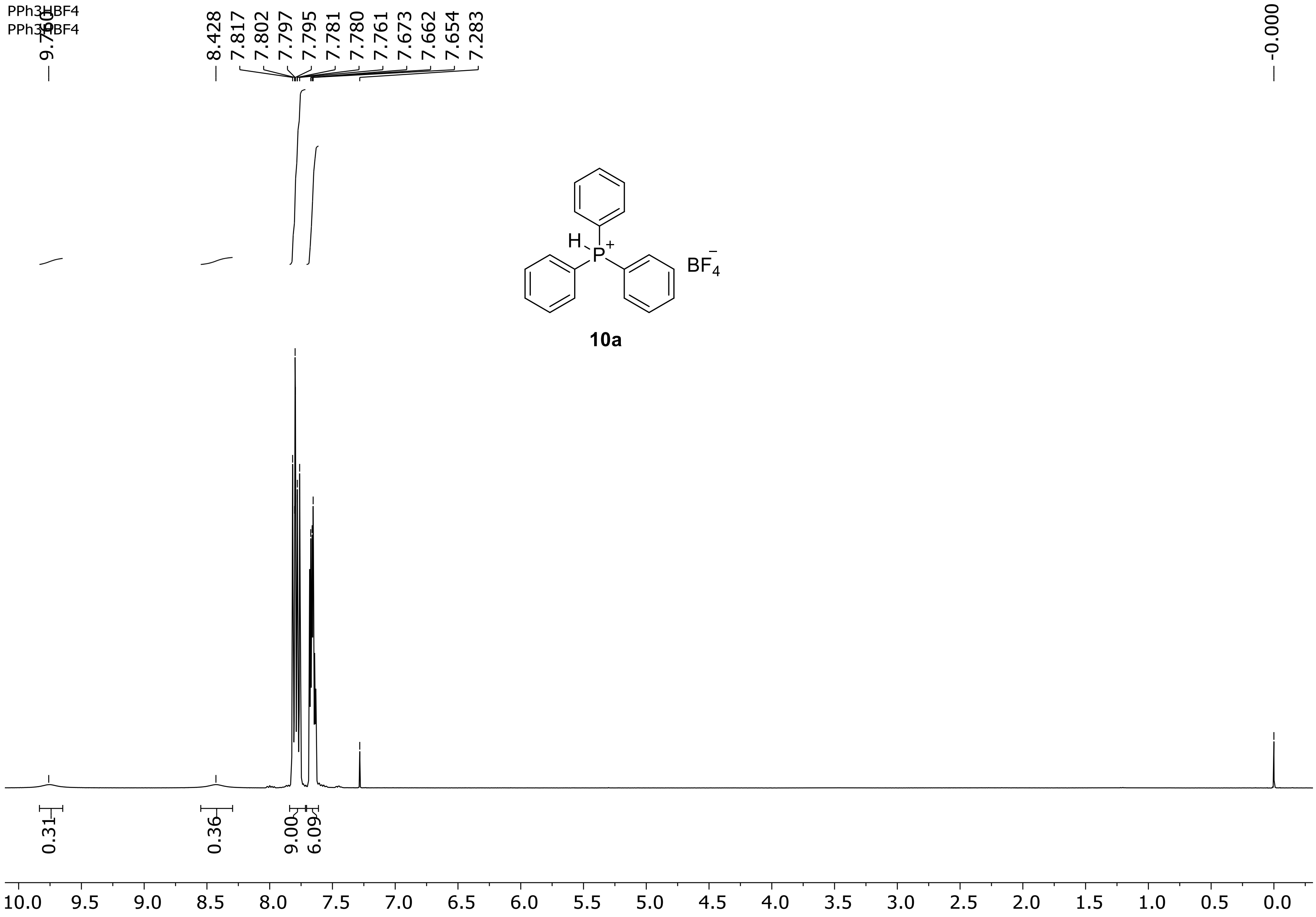

^{1}H NMR spectrum of triphenylphosphonium tetrafluoroborate (**10a**); 400 MHz/$CDCl_3$/TMS; δ (ppm).

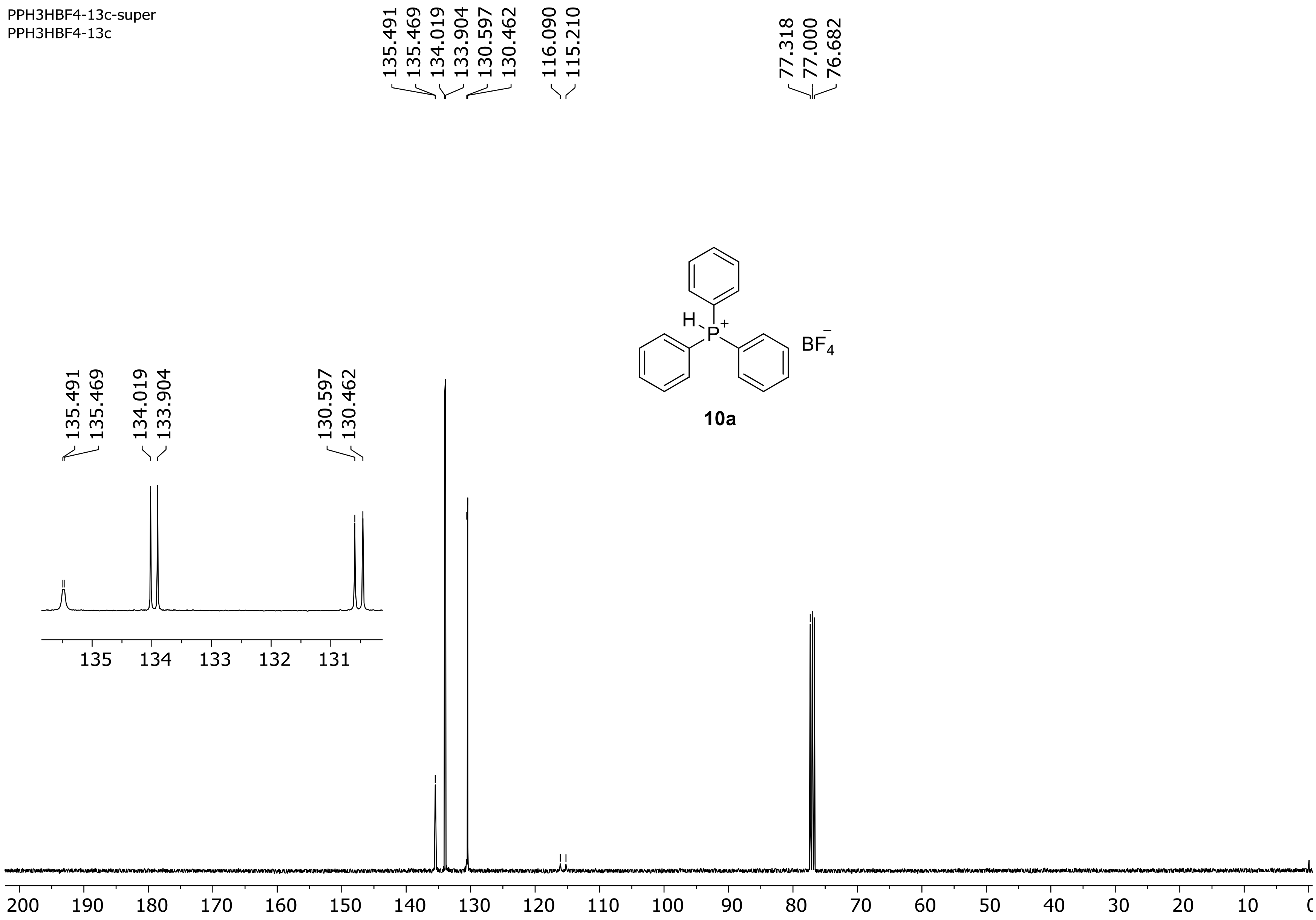

$^{13}C\{^{1}H\}$ NMR spectrum of triphenylphosphonium tetrafluoroborate (**10a**); 100 MHz/$CDCl_3$/TMS; δ (ppm).

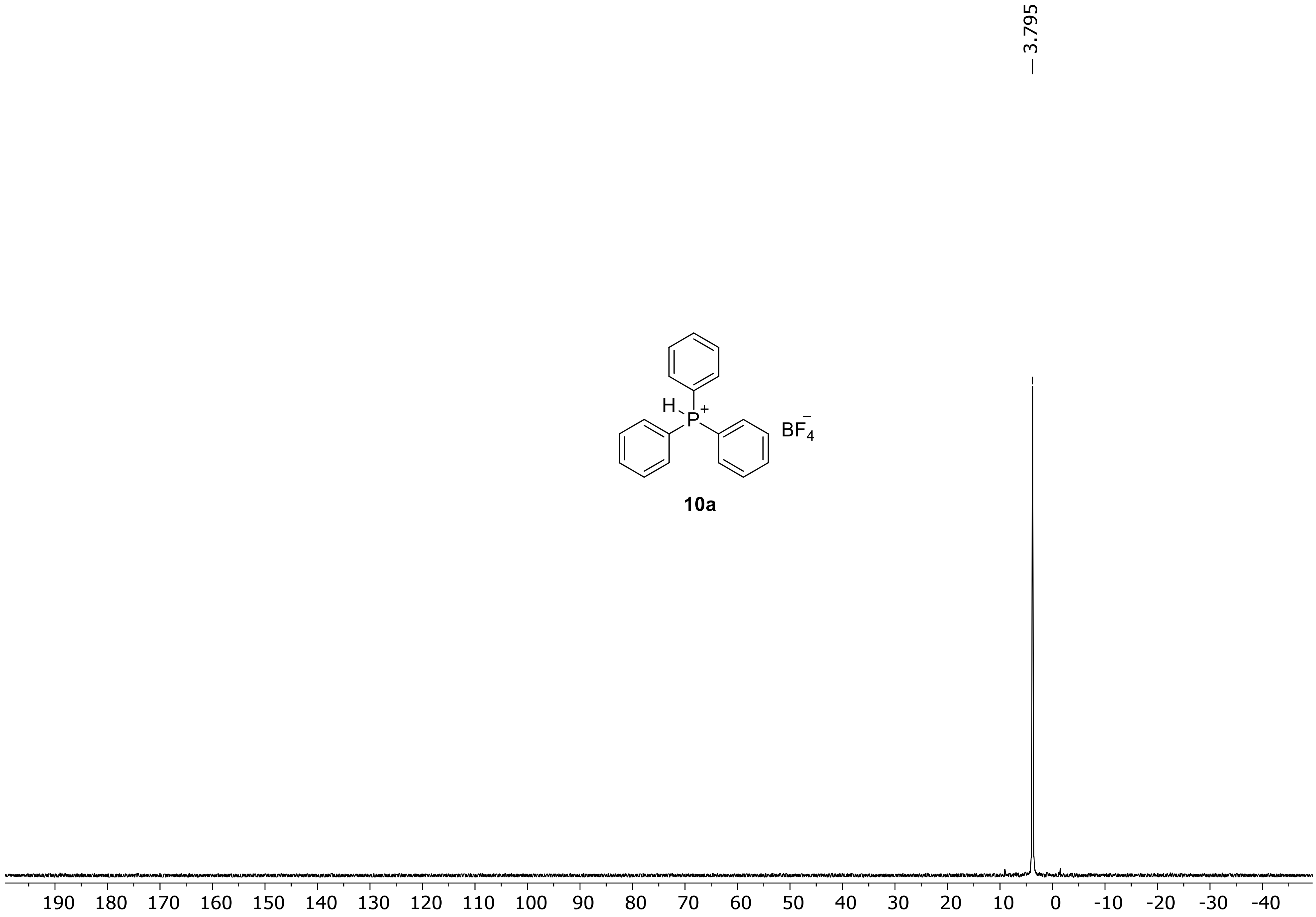

^{31}P NMR spectrum of triphenylphosphonium tetrafluoroborate (**10a**); 161.9 MHz/$CDCl_3$; δ (ppm).

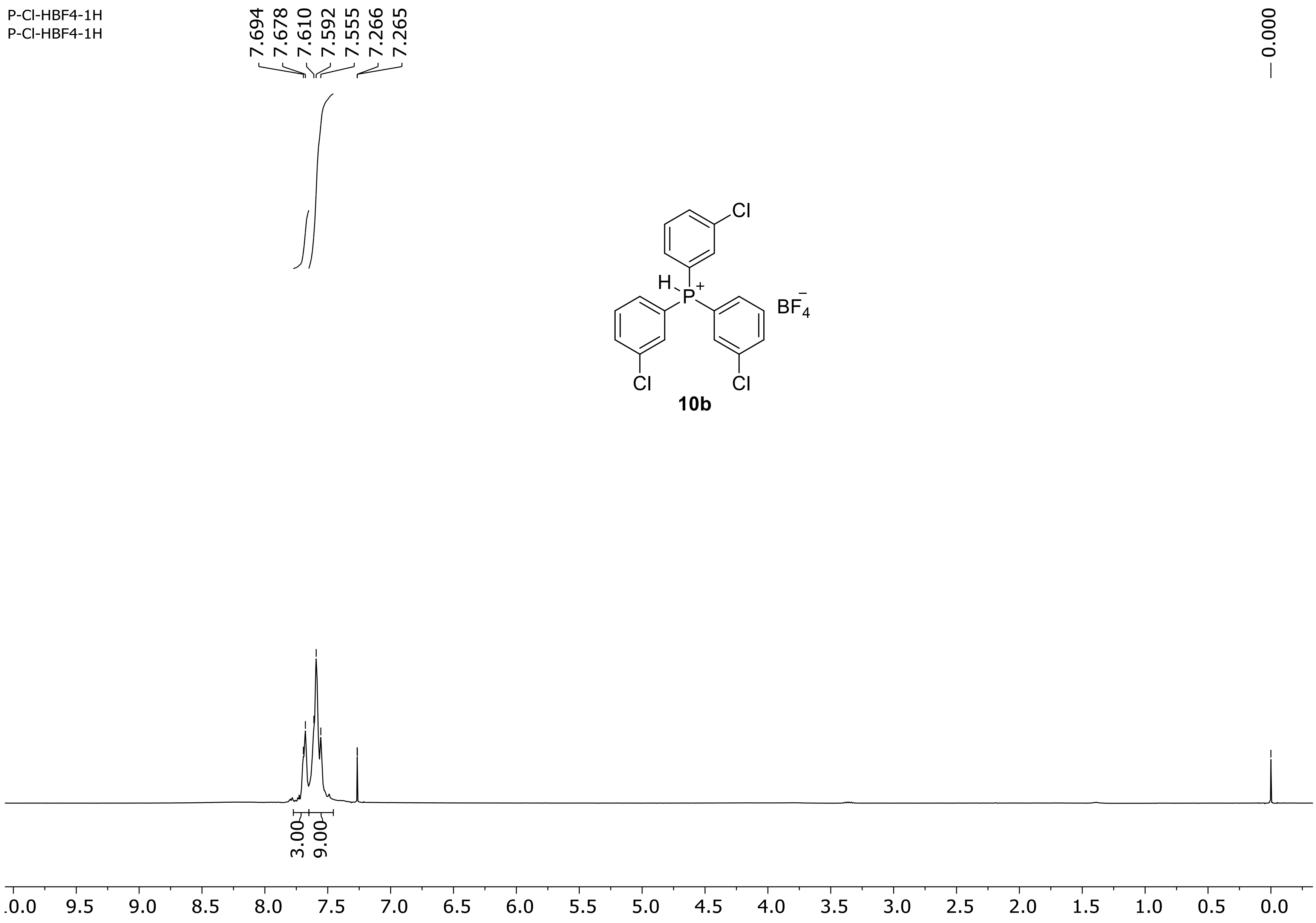

^{1}H NMR spectrum of tris(3-chlorophenyl)phosphonium tetrafluoroborate (**10b**); 400 MHz/$CDCl_3$/TMS; δ (ppm).

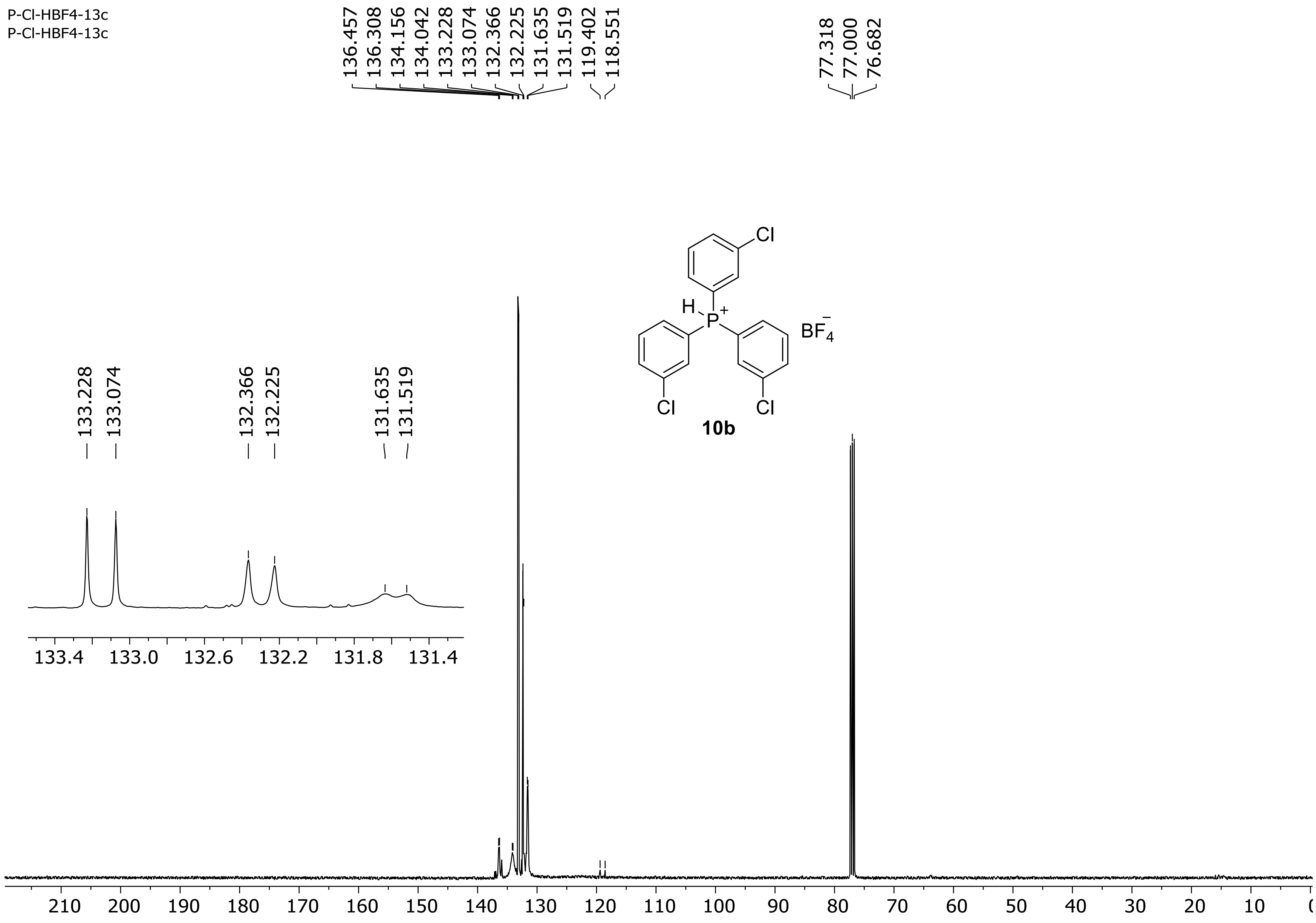

$^{13}C\{^1H\}$ NMR spectrum of tris(3-chlorophenyl)phosphonium tetrafluoroborate (**10b**); 100 MHz/$CDCl_3$/TMS; δ (ppm).

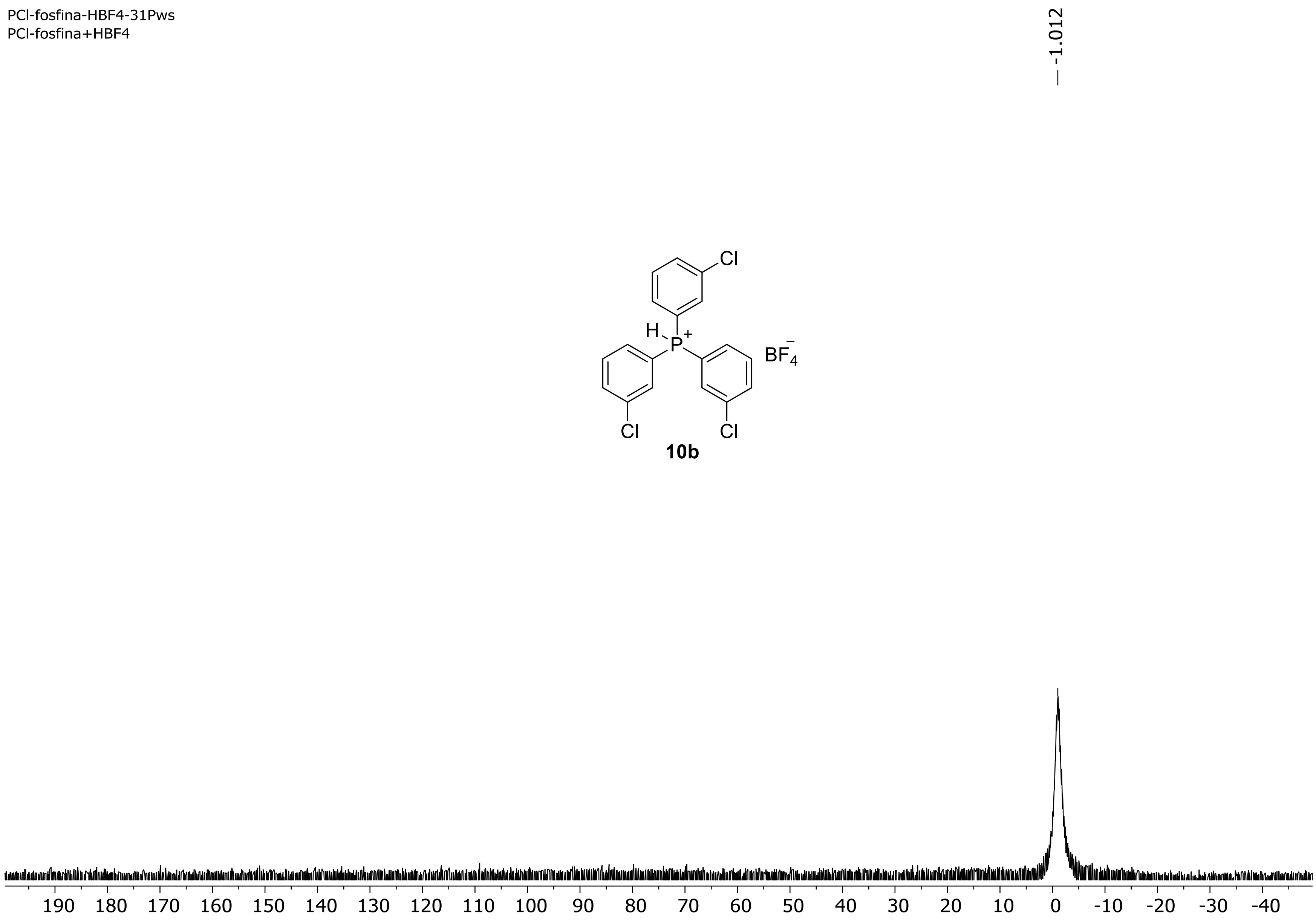

^{31}P NMR spectrum of tris(3-chlorophenyl)phosphonium tetrafluoroborate (**10b**); 161.9 MHz/$CDCl_3$; δ (ppm).

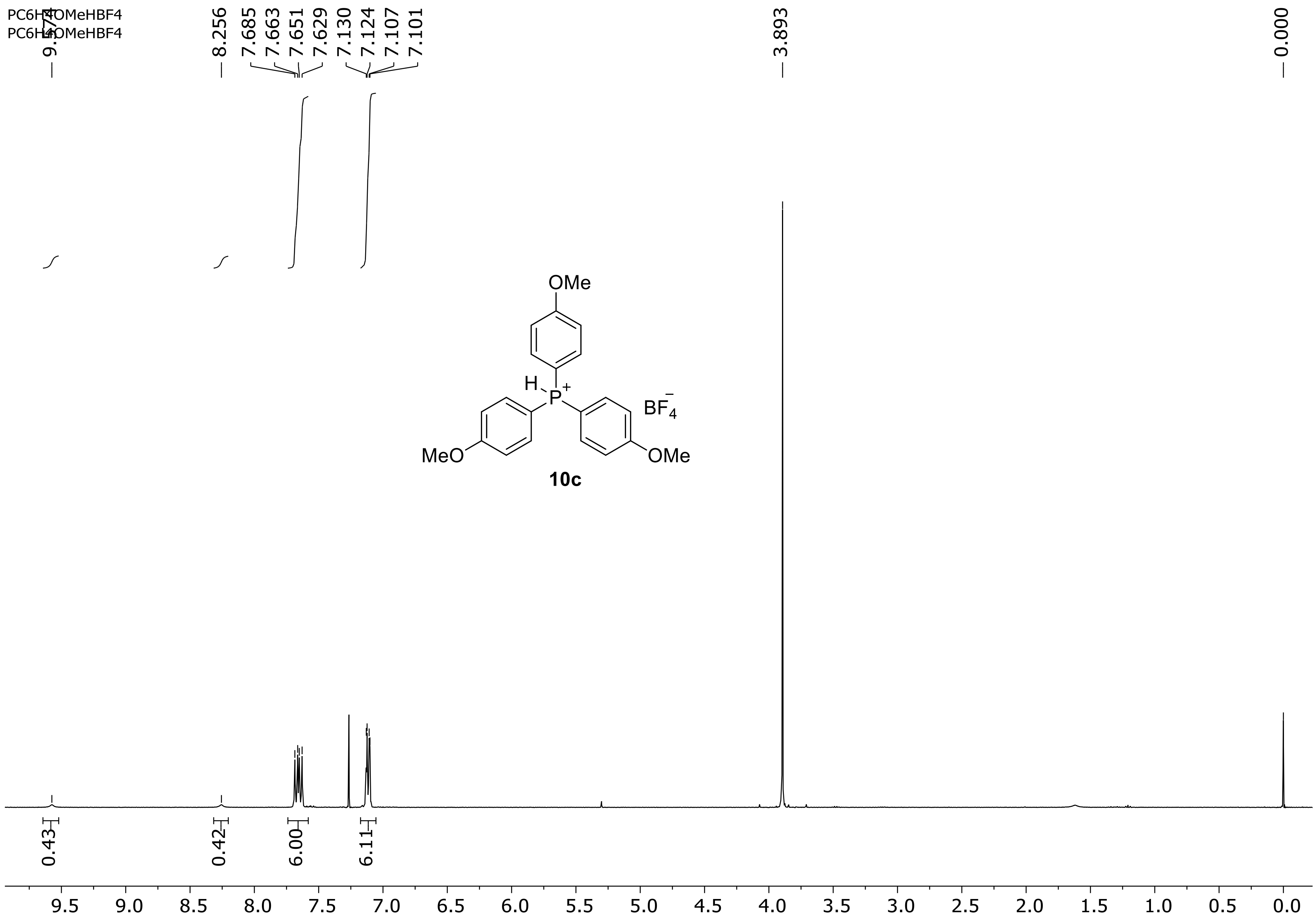

^{1}H NMR spectrum of tris(4-methoxyphenyl)phosphonium tetrafluoroborate (**10c**); 400 MHz/$CDCl_3$/TMS; δ (ppm).

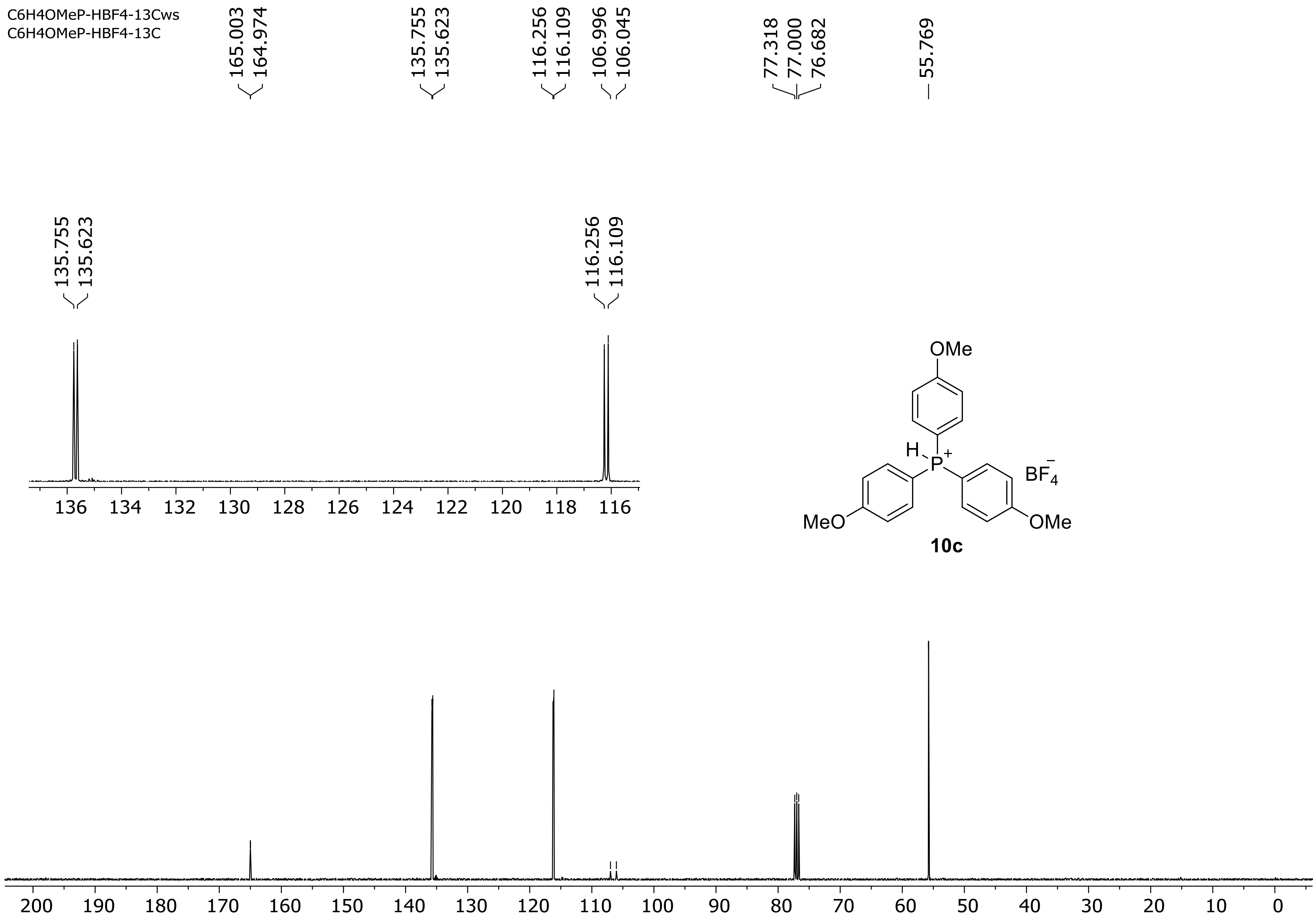

$^{13}C\{^{1}H\}$ NMR spectrum of tris(4-methoxyphenyl)phosphonium tetrafluoroborate (**10c**); 100 MHz/$CDCl_3$/TMS; δ (ppm).

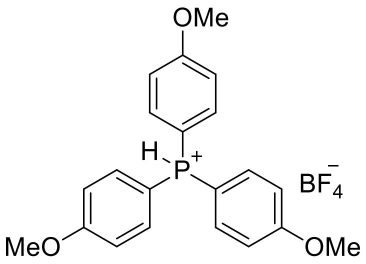

10c

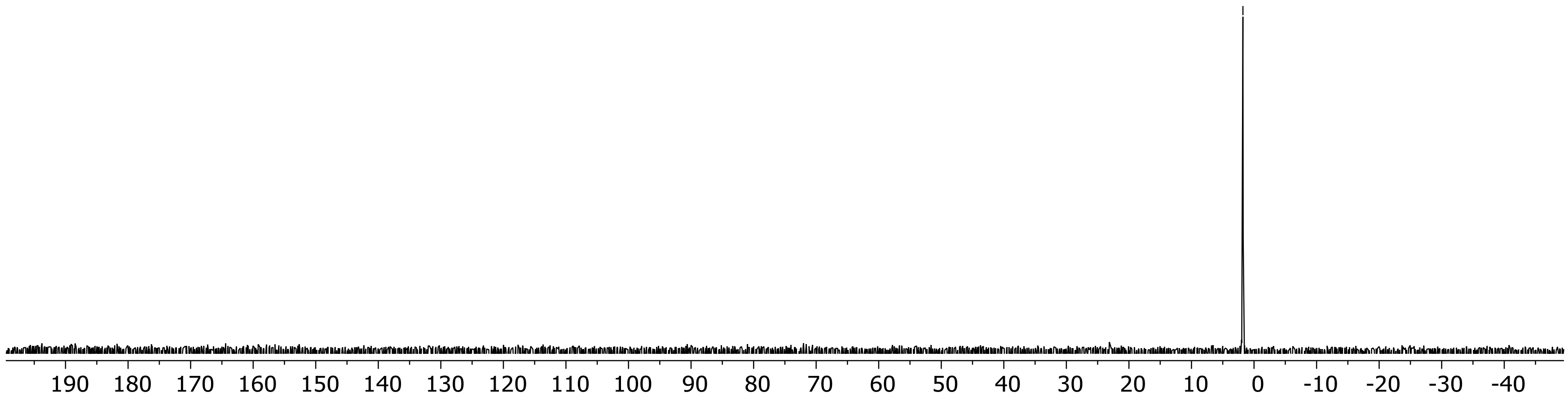

^{31}P NMR spectrum of tris(4-methoxyphenyl)phosphonium tetrafluoroborate (**10c**); 161.9 MHz/$CDCl_3$; δ (ppm).

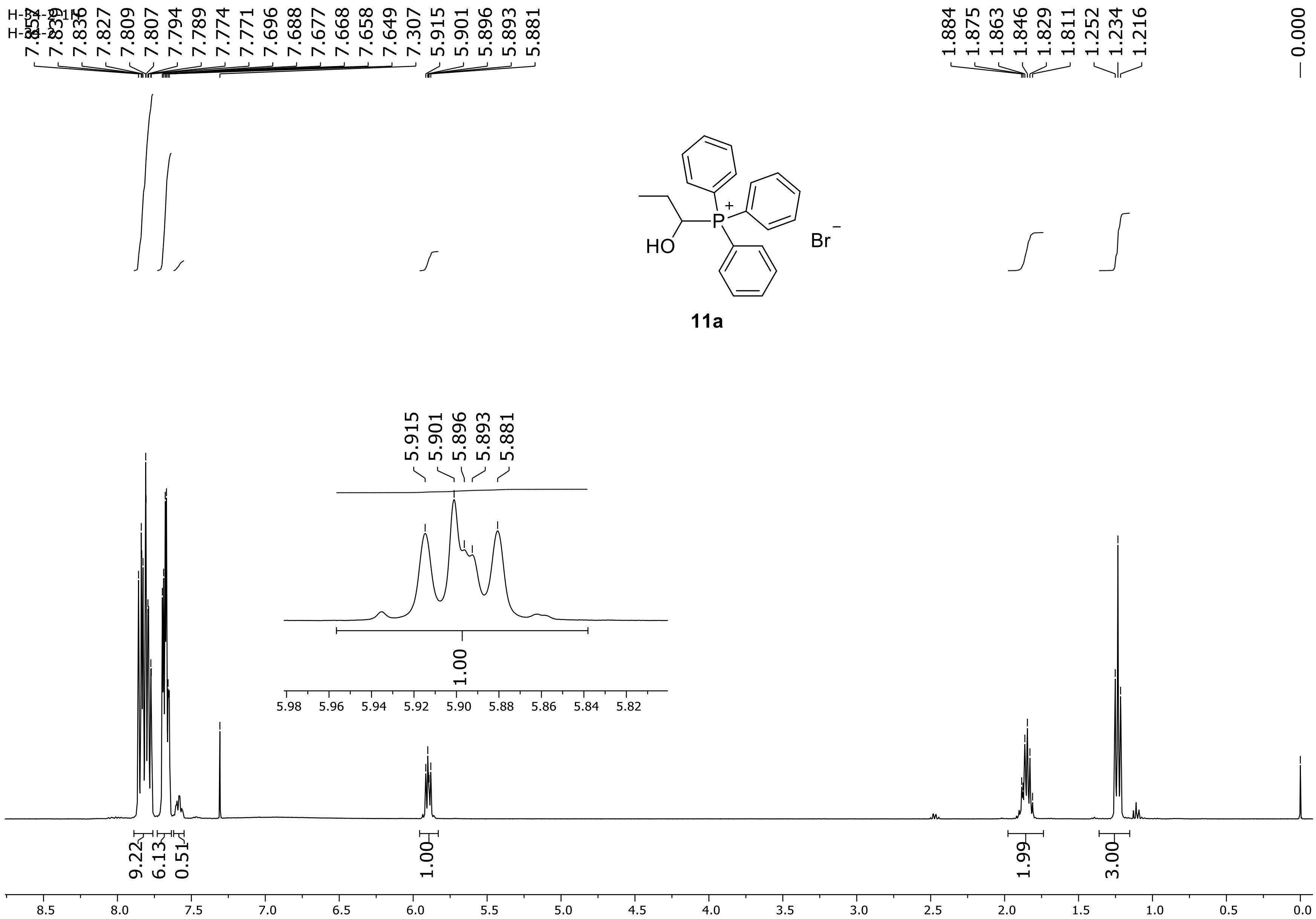

11a

^{1}H NMR spectrum of 1-hydroxypropyltriphenylphosphonium bromide (**11a**); 400 MHz/$CDCl_3$/TMS; δ (ppm).

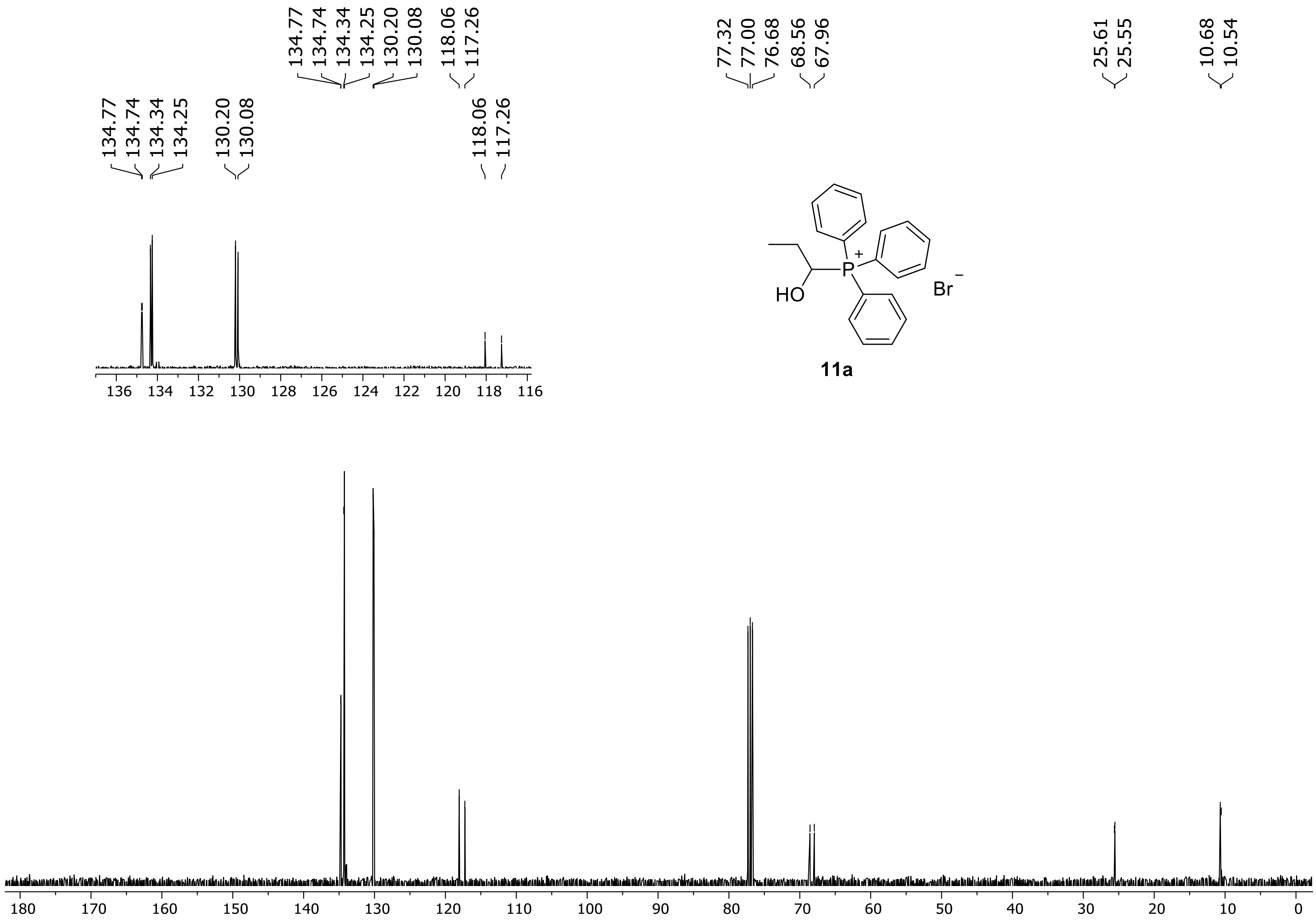

$^{13}C\{^1H\}$ NMR spectrum of 1-hydroxypropyltriphenylphosphonium bromide (**11a**); 100 MHz/$CDCl_3$/TMS; δ (ppm).

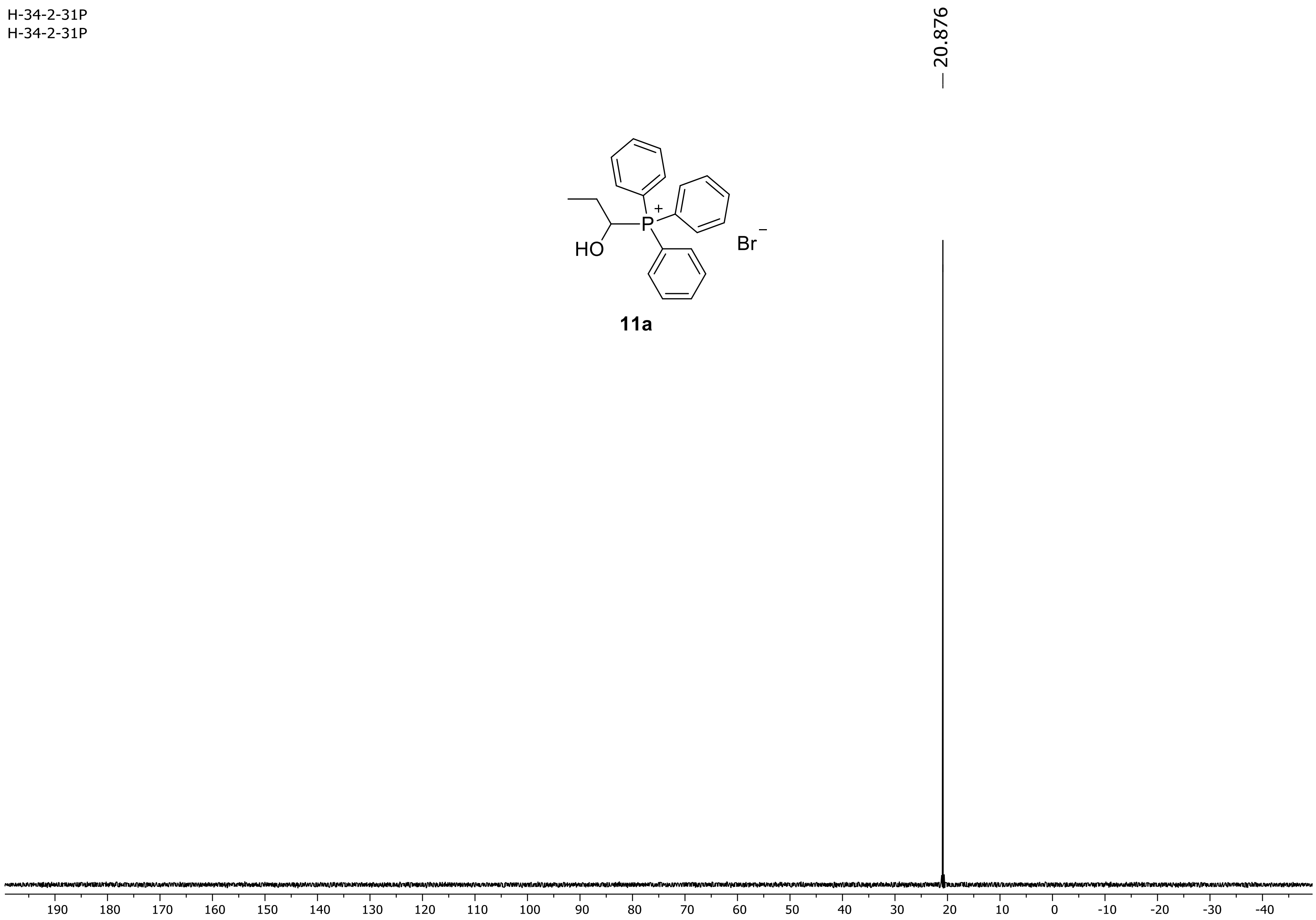

^{31}P NMR spectrum of 1-hydroxypropyltriphenylphosphonium bromide (**11a**); 161.9 MHz/$CDCl_3$; δ (ppm).

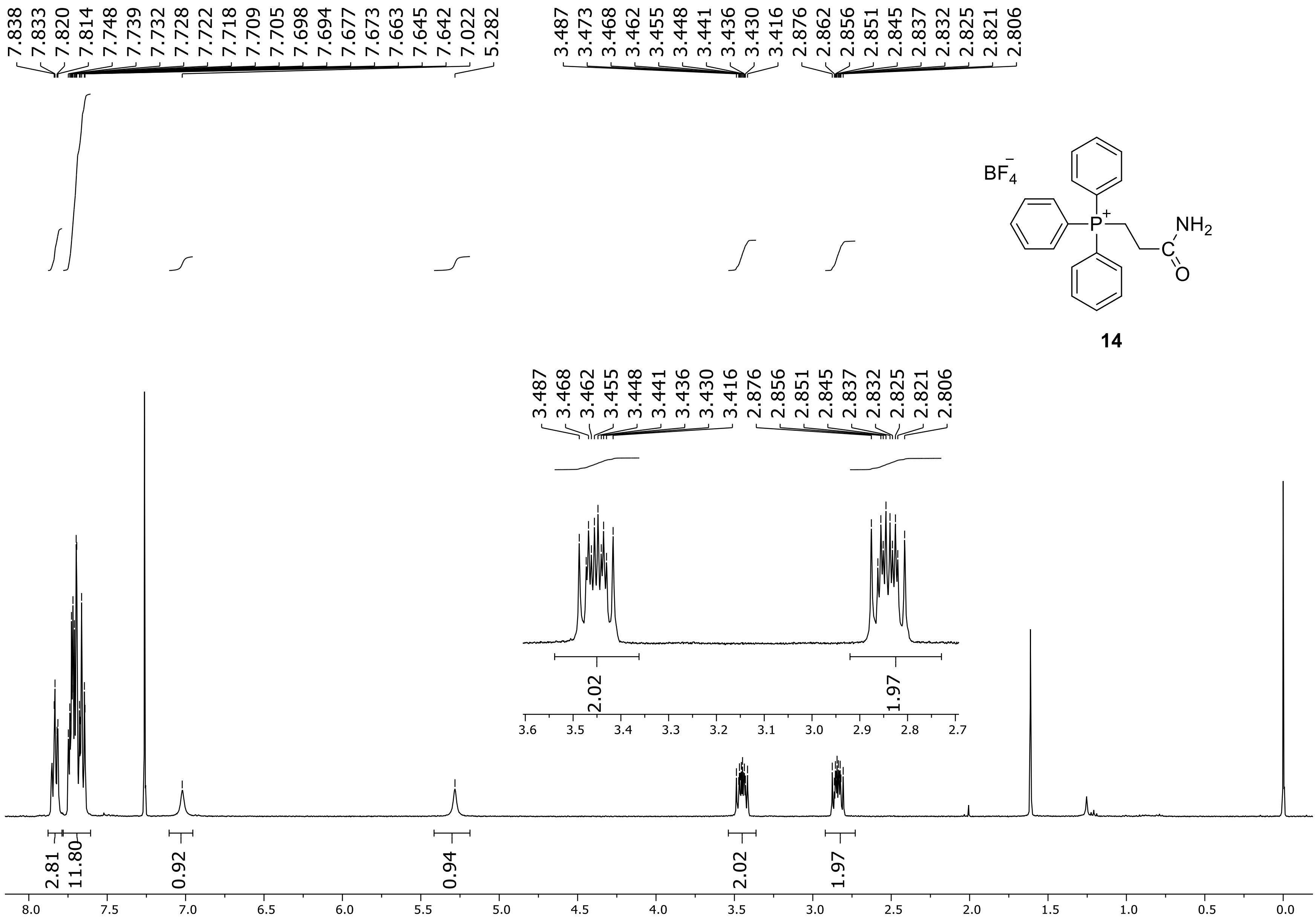

^{1}H NMR spectrum of 2-carbamoyletyhyltriphenylphosphonium tetrafluoroborate (**14**); 400 MHz/$CDCl_3$/TMS; δ (ppm).

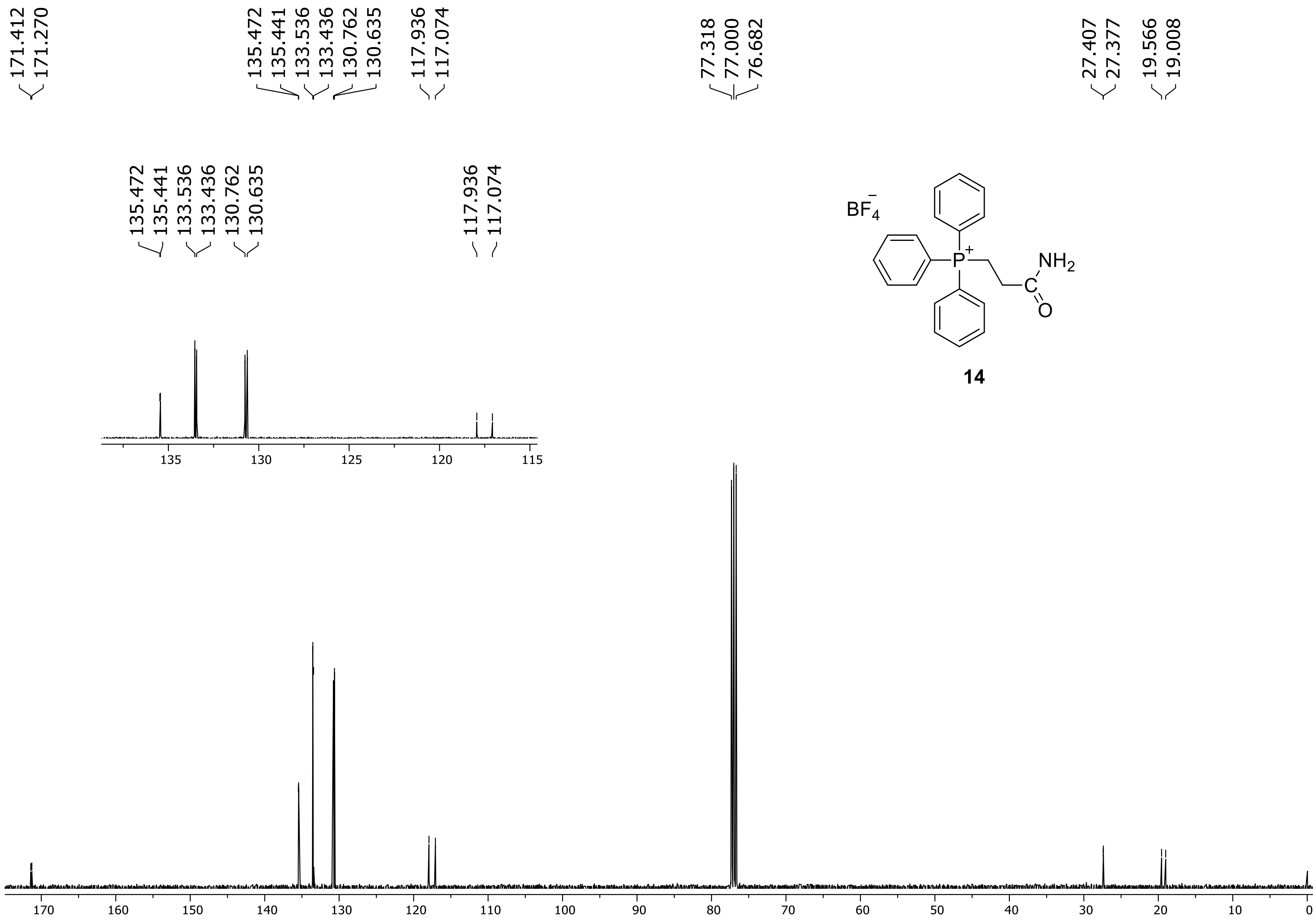

$^{13}C\{^{1}H\}$ NMR spectrum of 2-carbamoyletyhyltriphenylphosphonium tetrafluoroborate (**14**); 100 MHz/$CDCl_3$/TMS; δ (ppm).

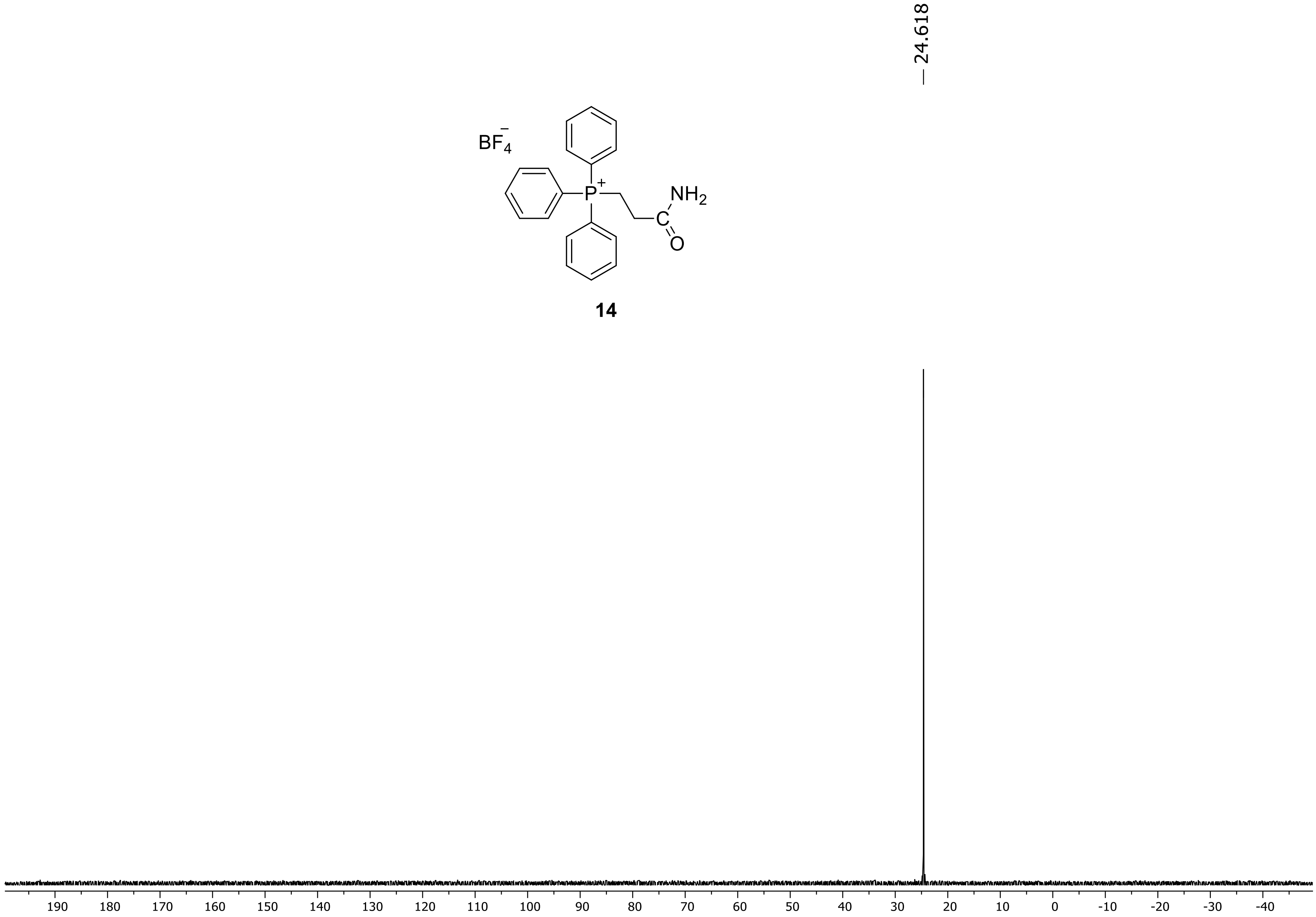

^{31}P NMR spectrum of 2-carbamoyletyhyltriphenylphosphonium tetrafluoroborate (**14**); 161.9 MHz/$CDCl_3$; δ (ppm).

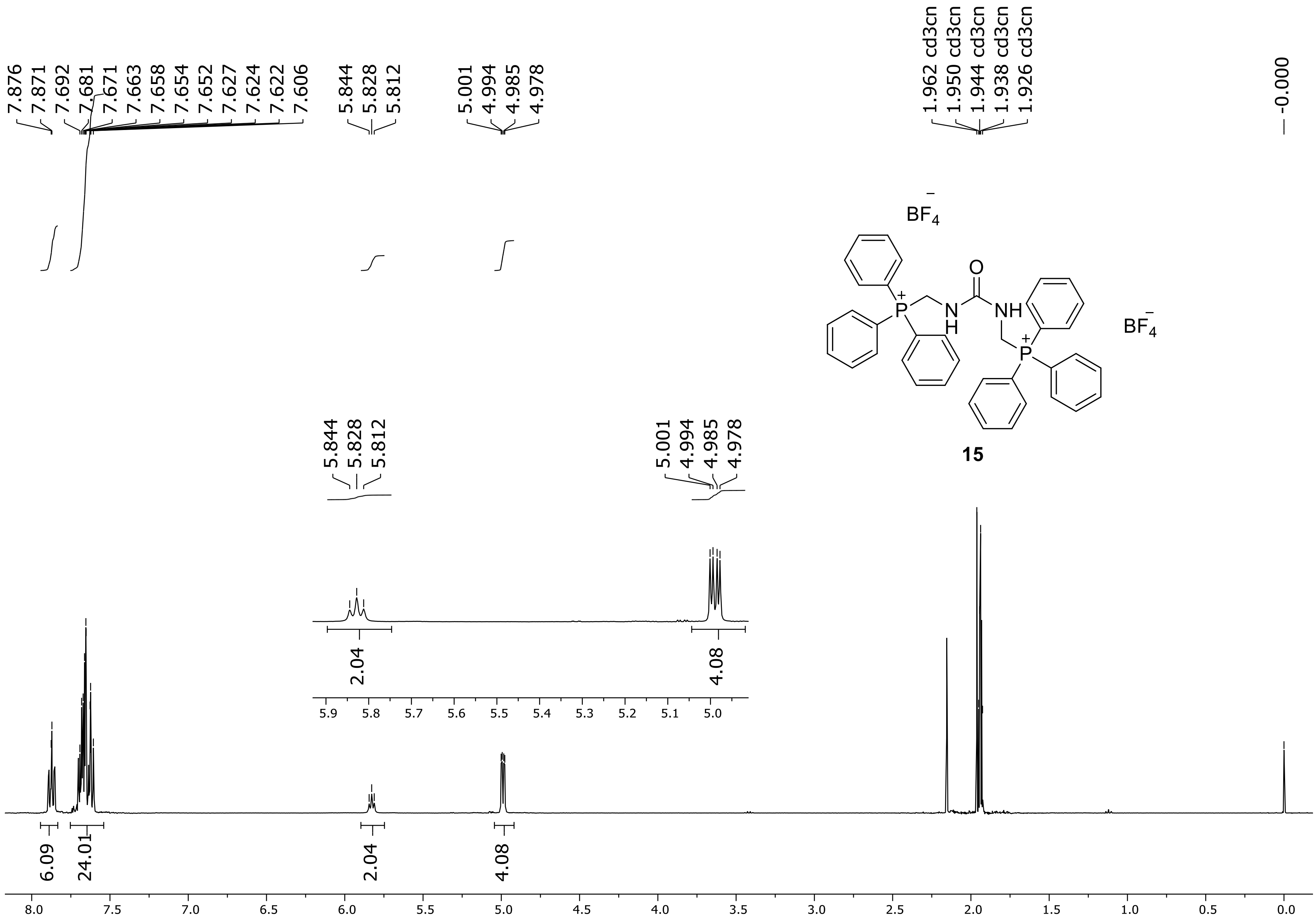

^{1}H NMR spectrum of 1,1'-(carbonyldimino)bis(methyltriphenylphosphonium) bis(tetrafluoroborate) (**15**); 400 MHz/CD_3CN/TMS; δ (ppm).

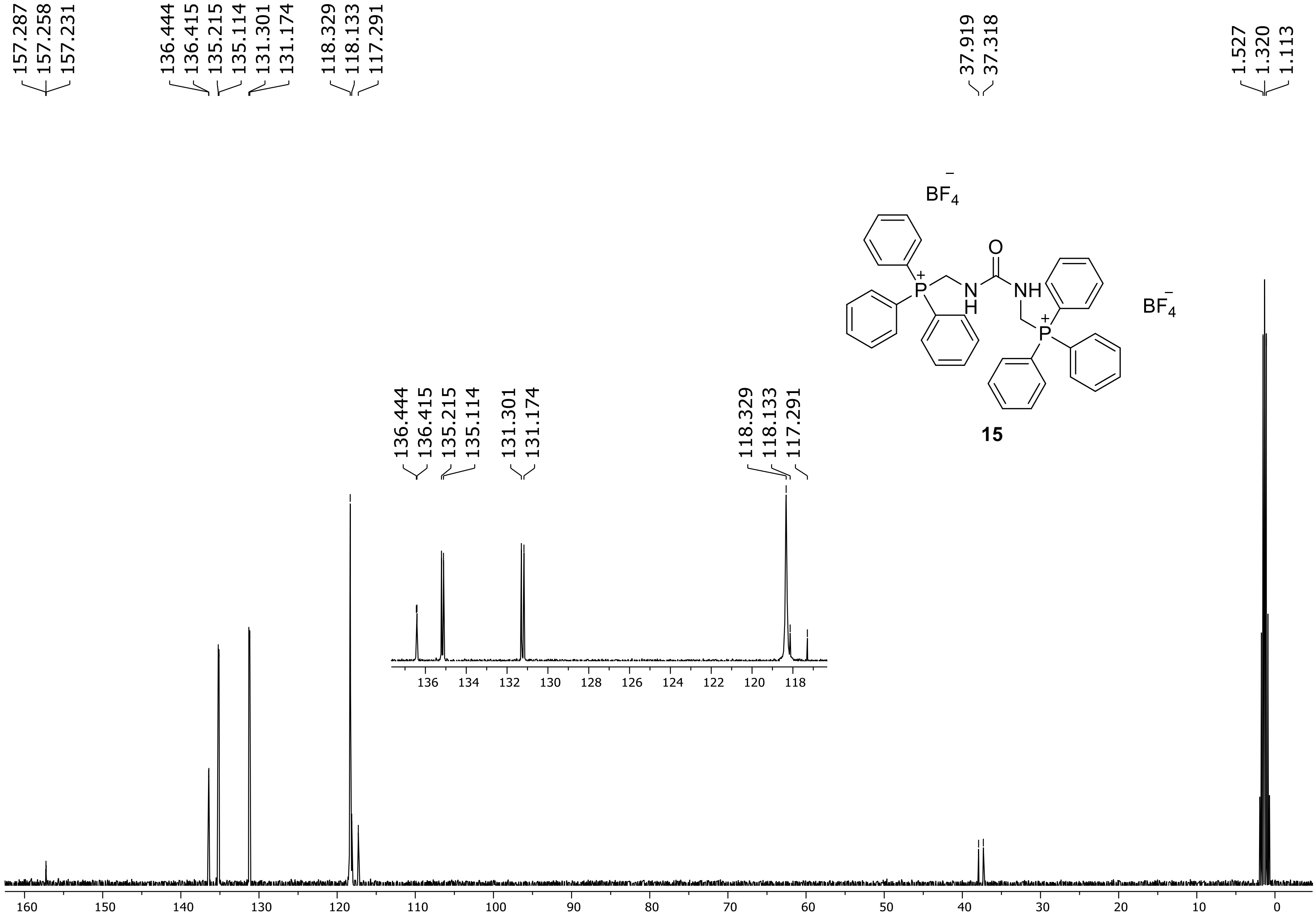

$^{13}C\{^{1}H\}$ NMR spectrum of 1,1’-(carbonyldimino)bis(methyltriphenylphosphonium) bis(tetrafluoroborate) (**15**); 100 MHz/CD_3CN/TMS; δ (ppm).

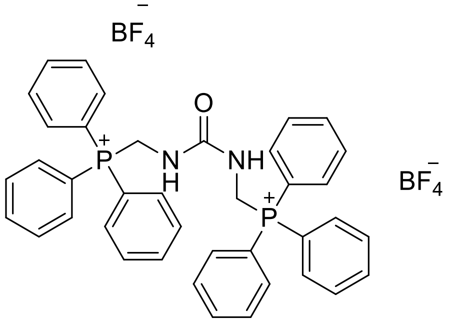

15

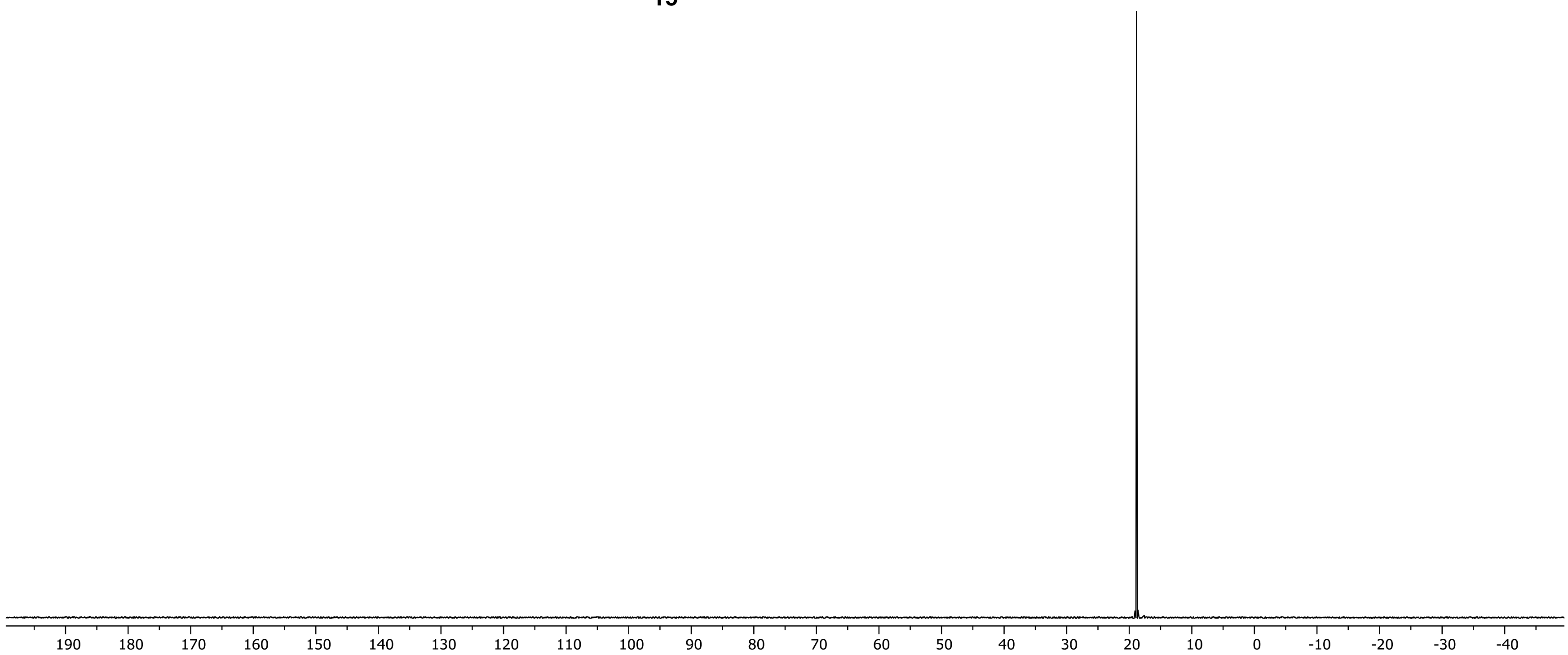

^{31}P NMR spectrum of 1,1'-(carbonyldimino)bis(methyltriphenylphosphonium) bis(tetrafluoroborate) (**15**); 161.9 MHz/CD_3CN; δ (ppm).

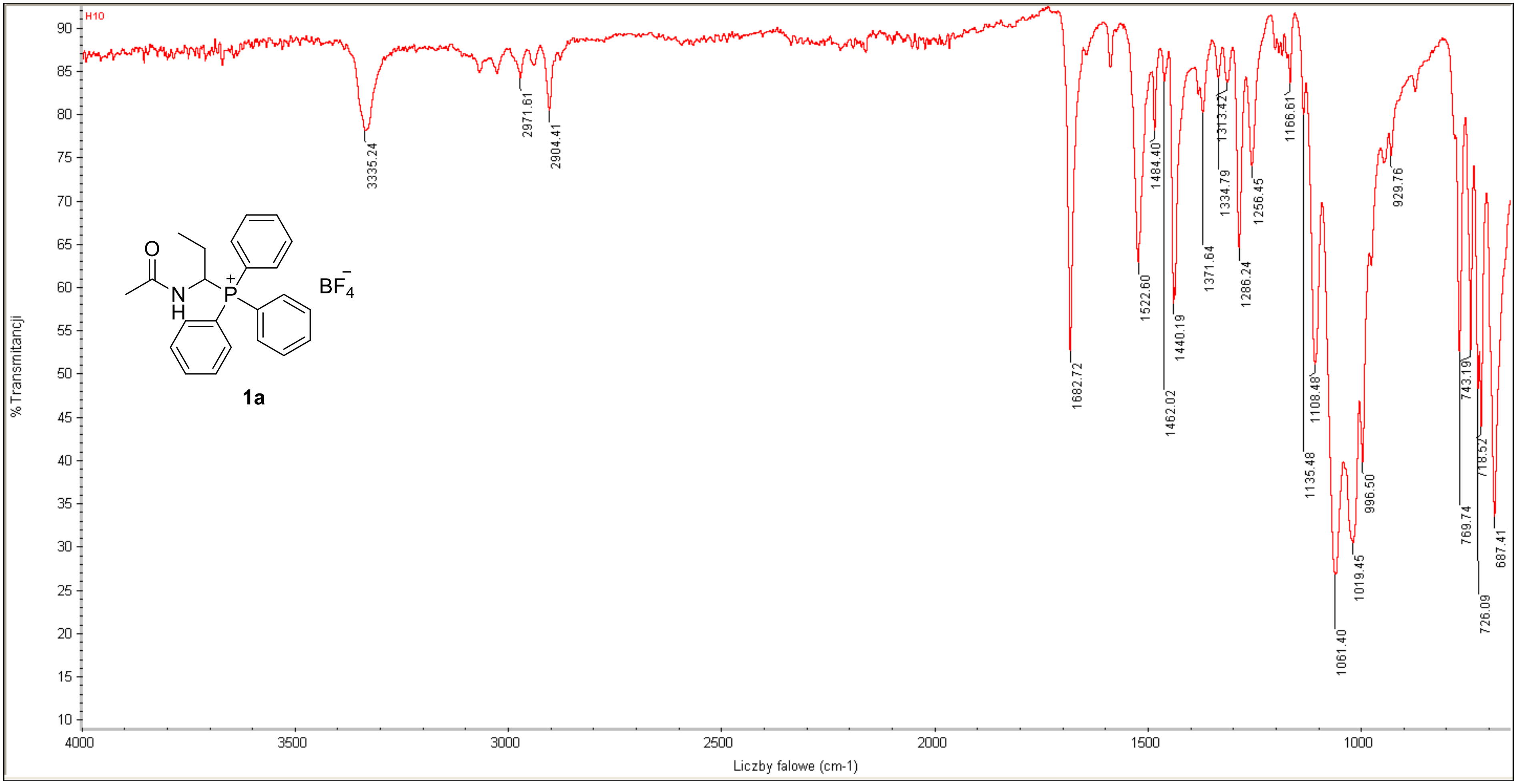

IR spectrum of 1-(*N*-acetylamino)propyltriphenylphosphonium tetrafluoroborate (**1a**); ATR (cm^{-1}).

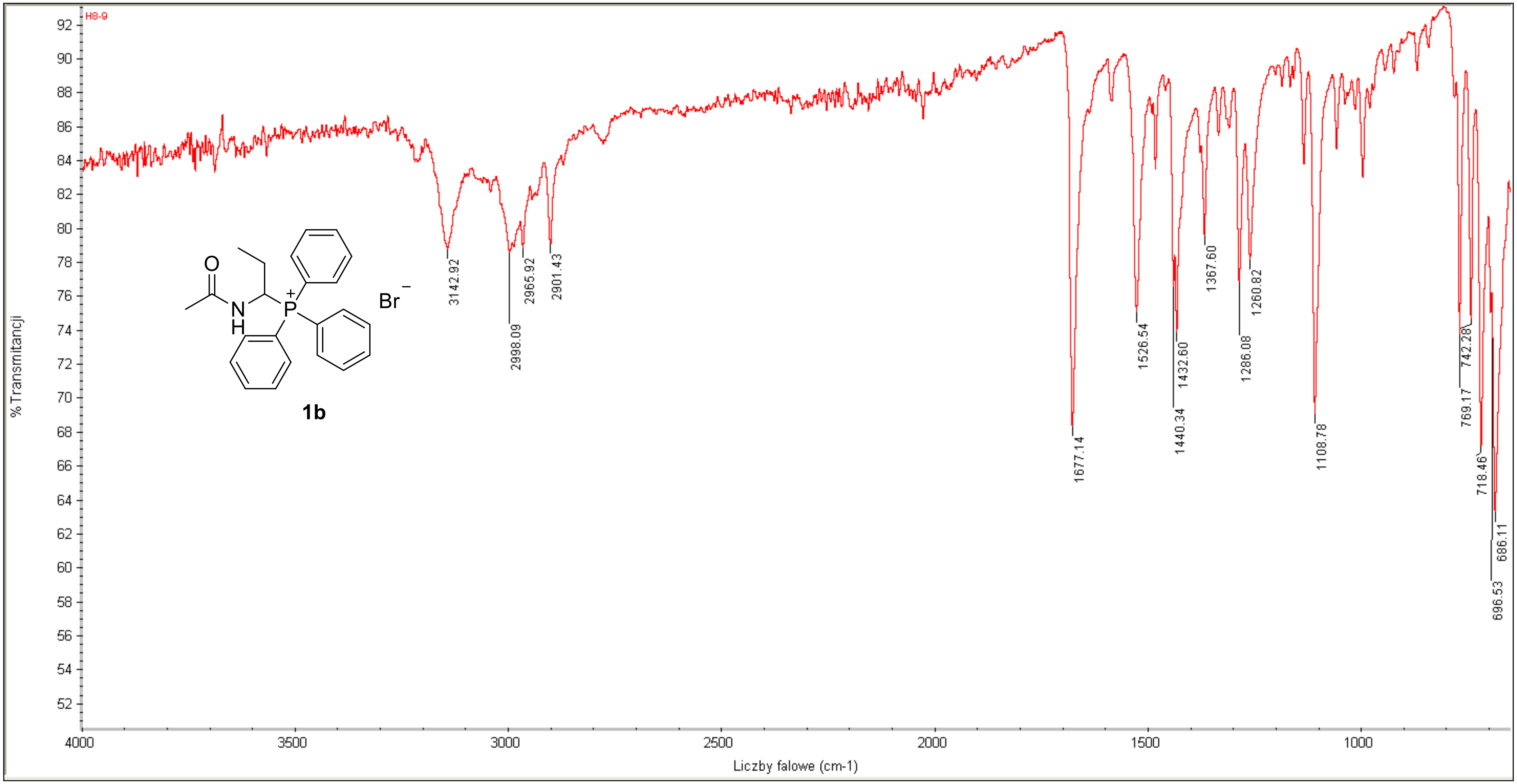

IR spectrum of 1-(*N*-acetylamino)propyltriphenylphosphonium bromide (**1b**); ATR (cm^{-1}).

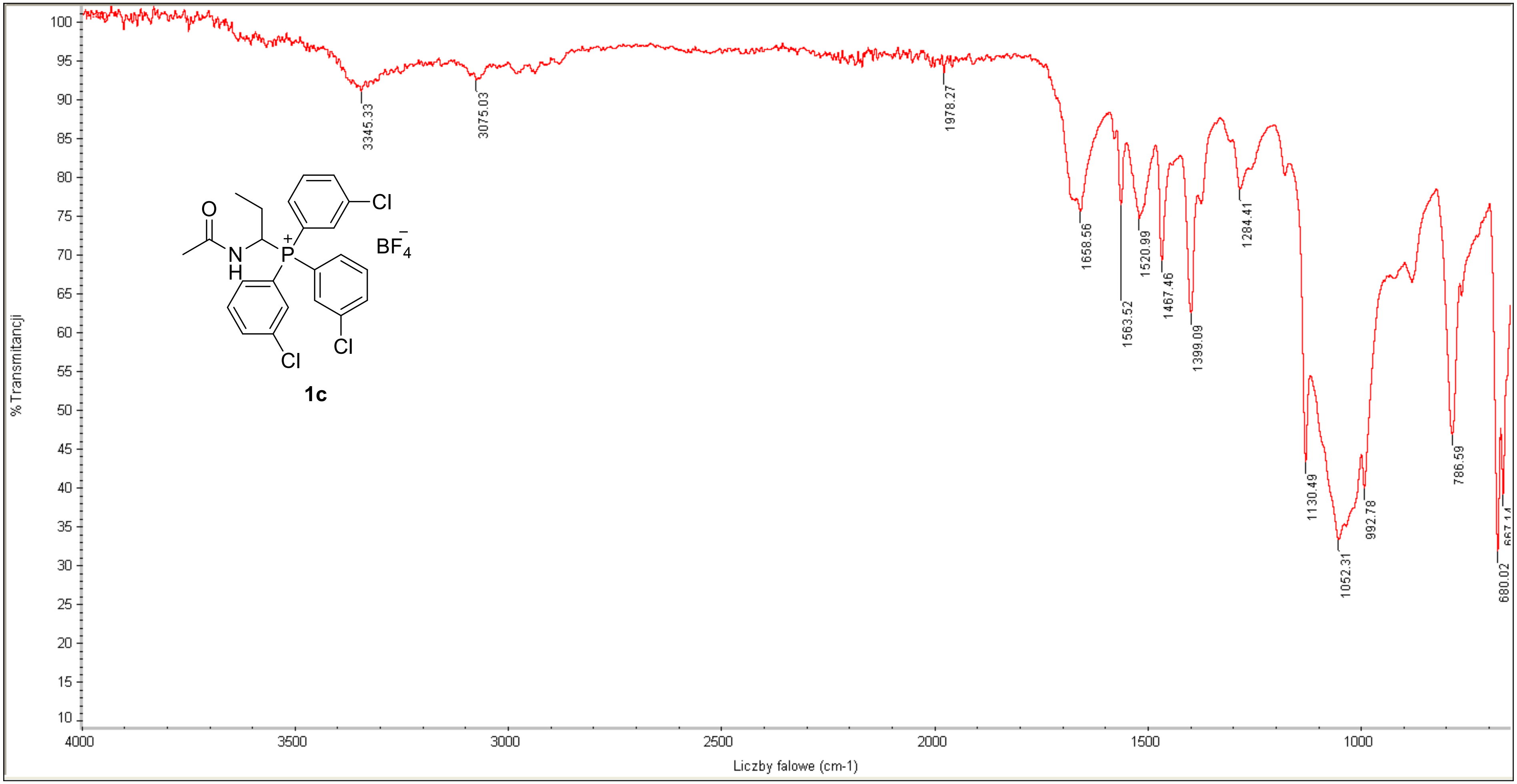

IR spectrum of 1-(*N*-acetylamino)propyltris(3-chlorophenyl)phosphonium tetrafluoroborate (**1c**); ATR (cm^{-1}).

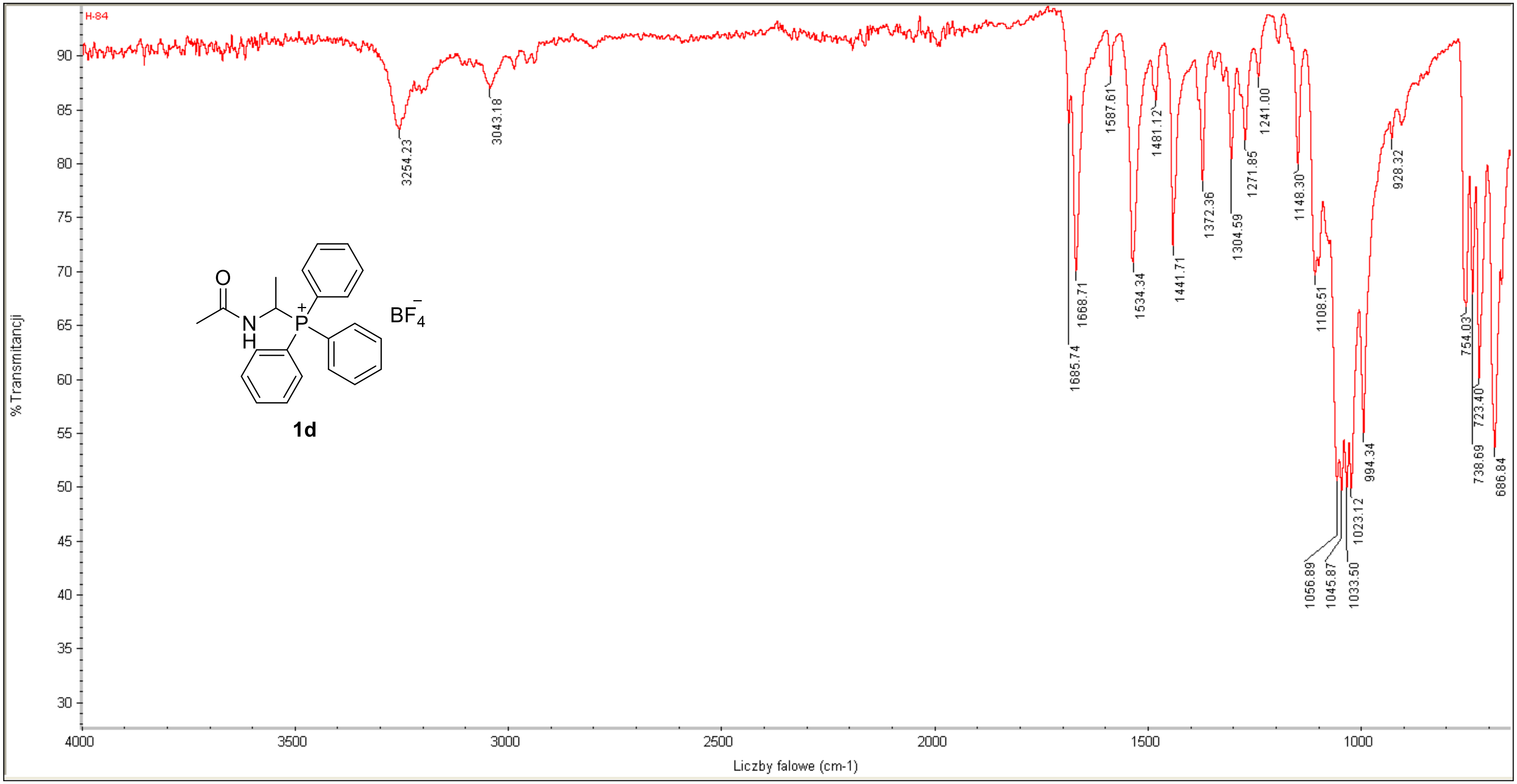

IR spectrum of 1-(*N*-acetylamino)ethyltriphenylphosphonium tetrafluoroborate (**1d**); ATR (cm^{-1}).

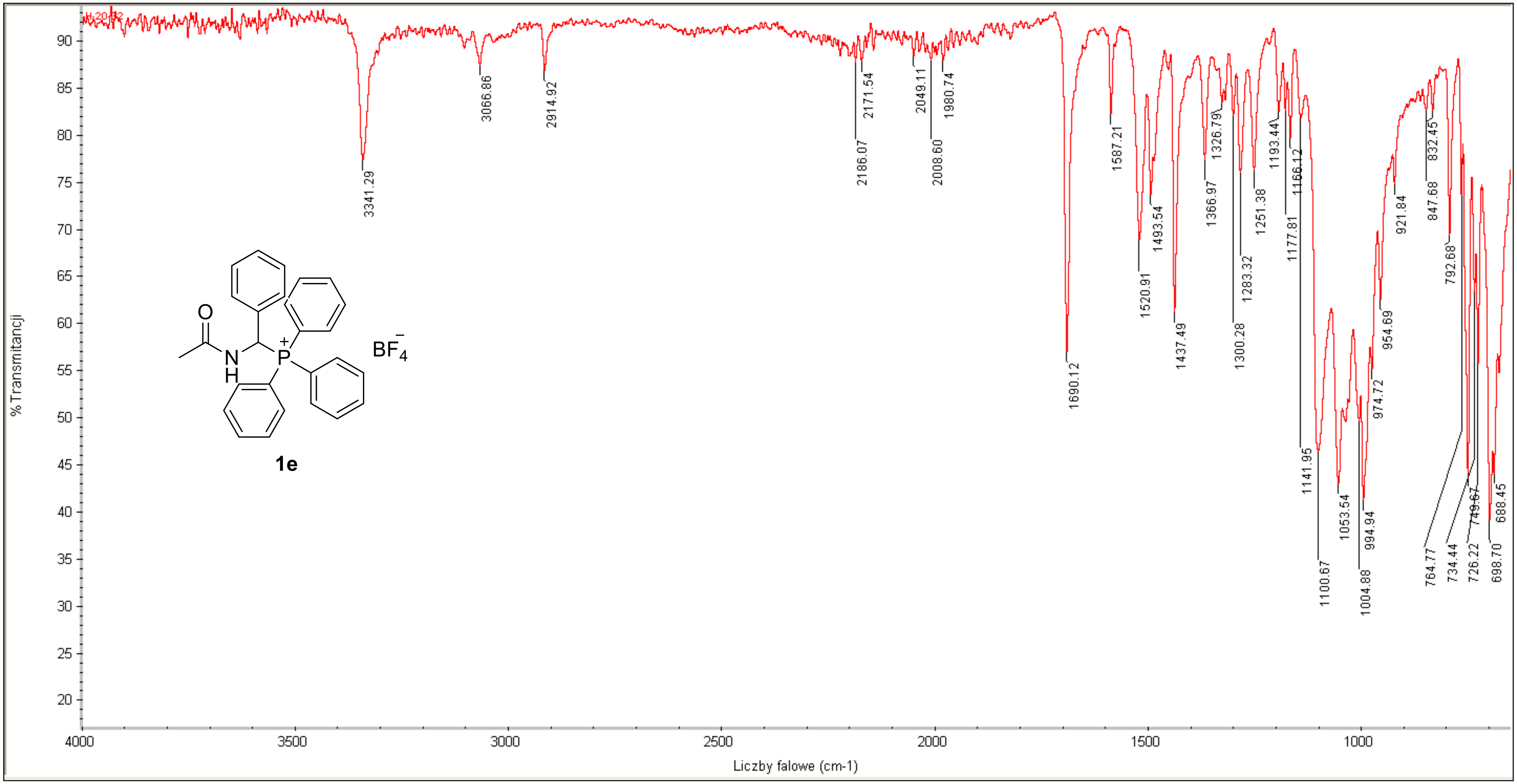

IR spectrum of (*N*-acetylamino)phenylmethyltriphenylphosphonium tetrafluoroborate (**1e**); ATR (cm^{-1}).

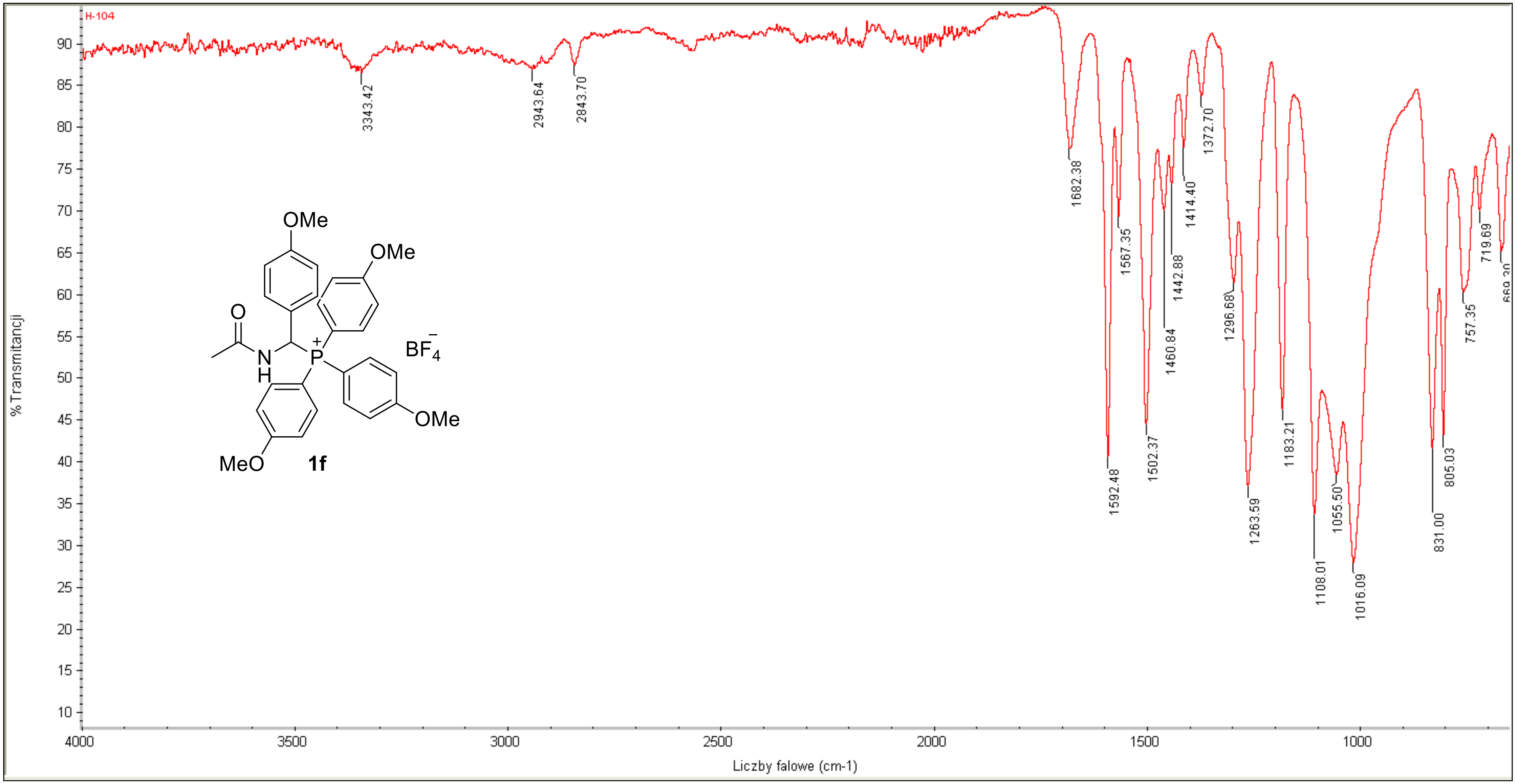

IR spectrum of 1-(*N*-acetylamino)-1-(4-metoxyphenyl)methyltris(4-metoxyphenyl)phosphonium tetrafluoroborate (**1f**); ATR (cm^{-1}).

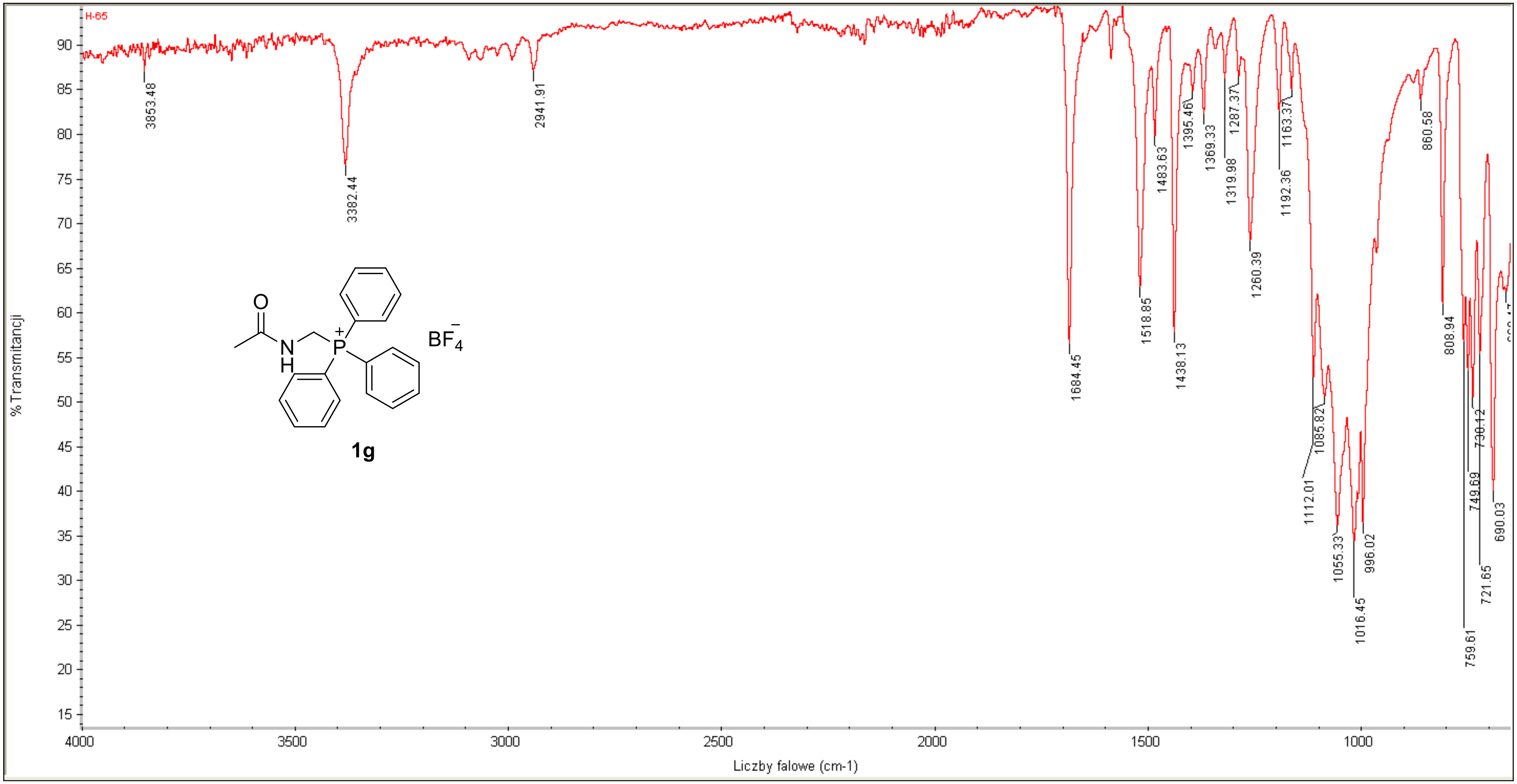

IR spectrum of (*N*-acetylamino)methyltriphenylphosphonium tetrafluoroborate (**1g**); ATR (cm^{-1}).

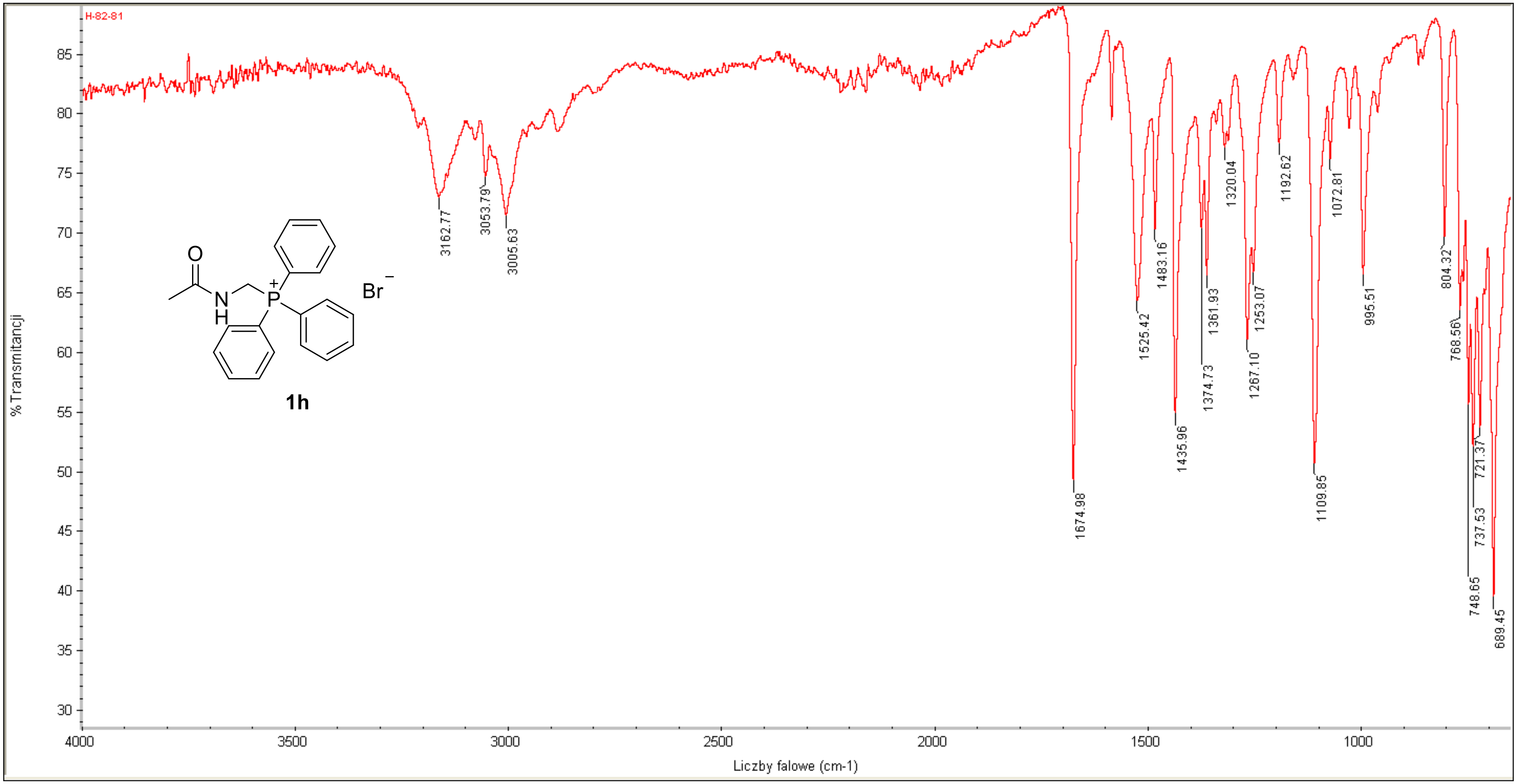

IR spectrum of (*N*-acetylamino)methyltriphenylphosphonium bromide (**1h**); ATR (cm^{-1}).

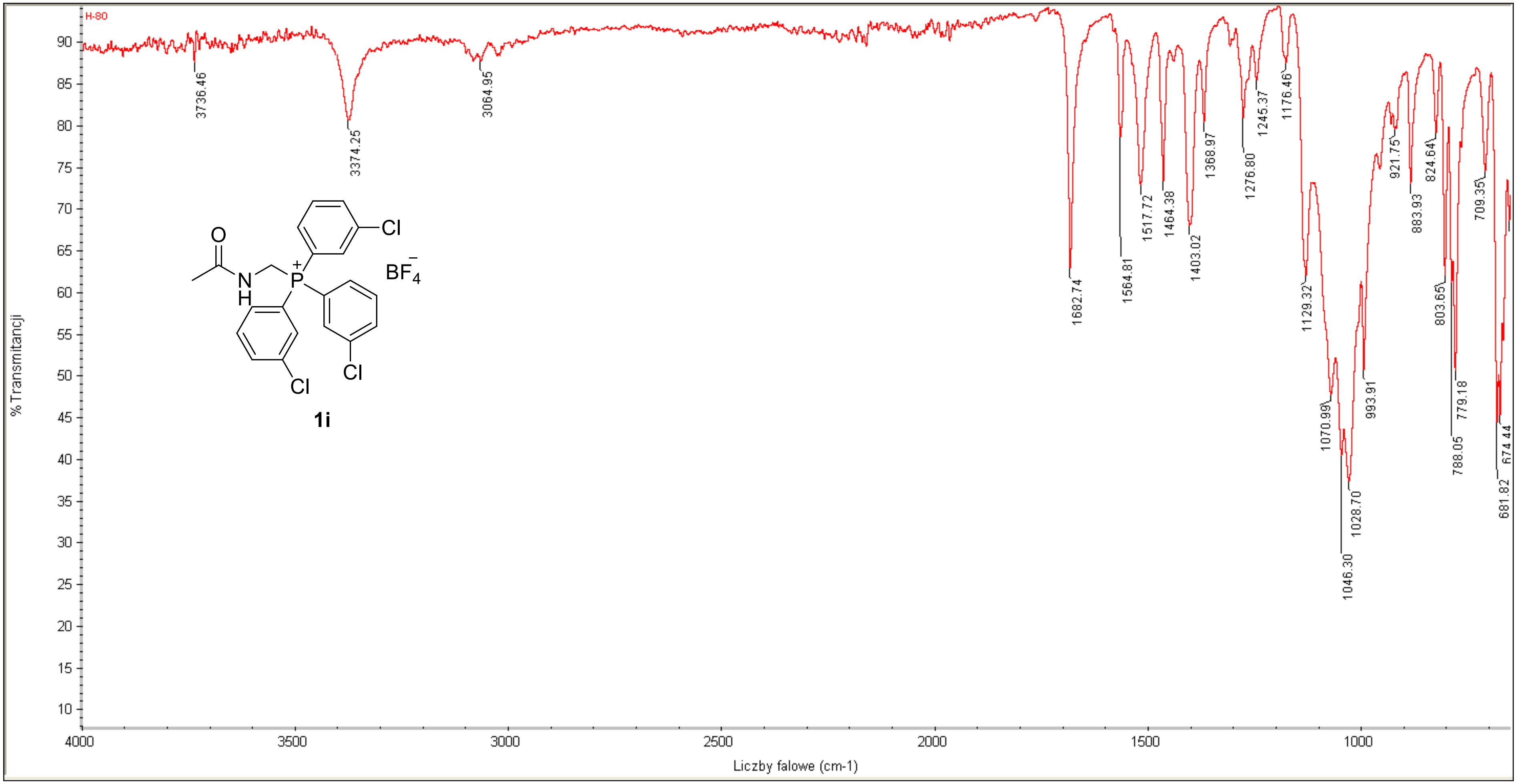

IR spectrum of (*N*-acetylamino)methyltris(3-chlorophenyl)phosphonium tetrafluoroborate (**1i**); ATR (cm^{-1}).

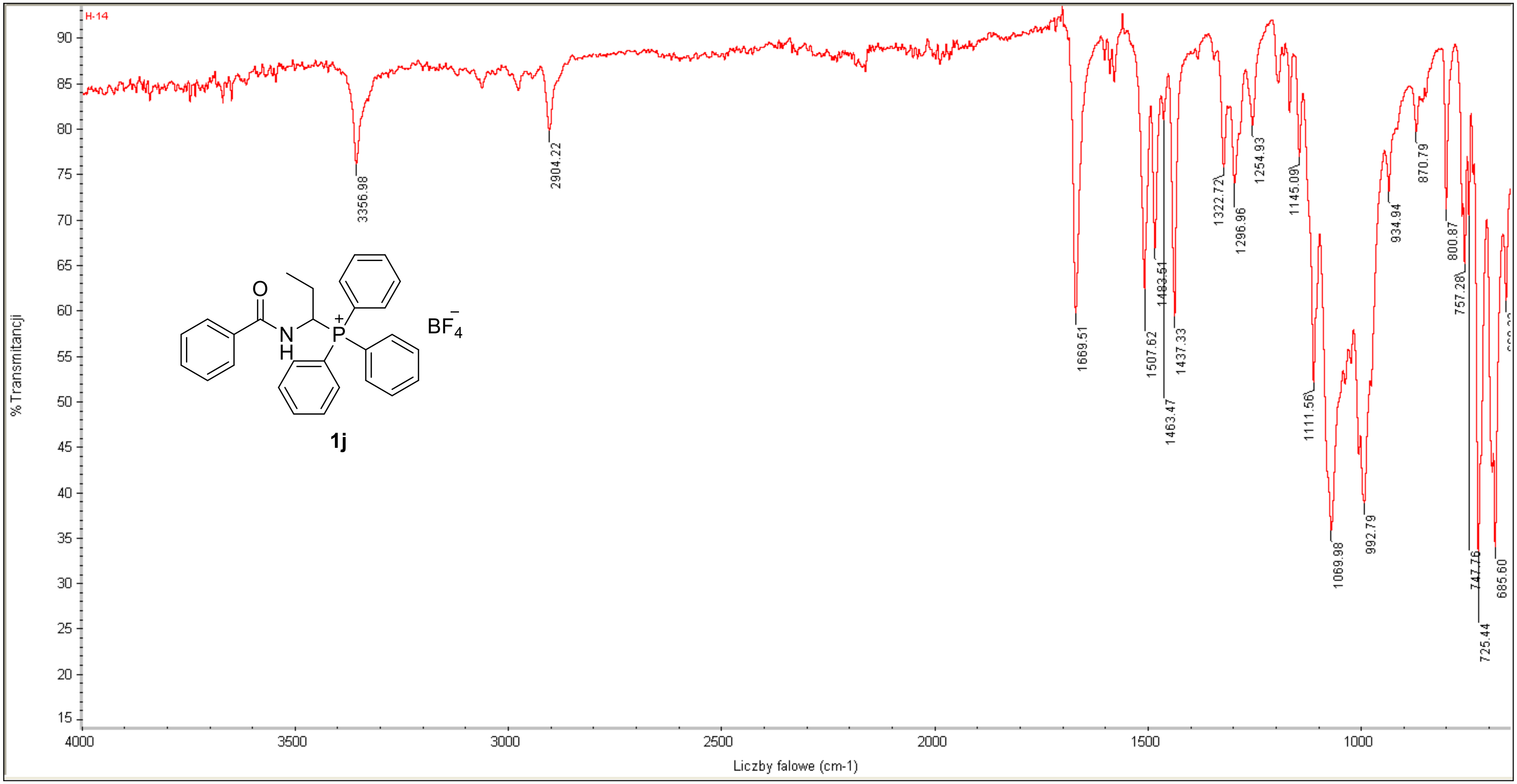

IR spectrum of 1-(*N*-benzoylamino)propyltriphenylphosphonium tetrafluoroborate (**1j**); ATR (cm^{-1}).

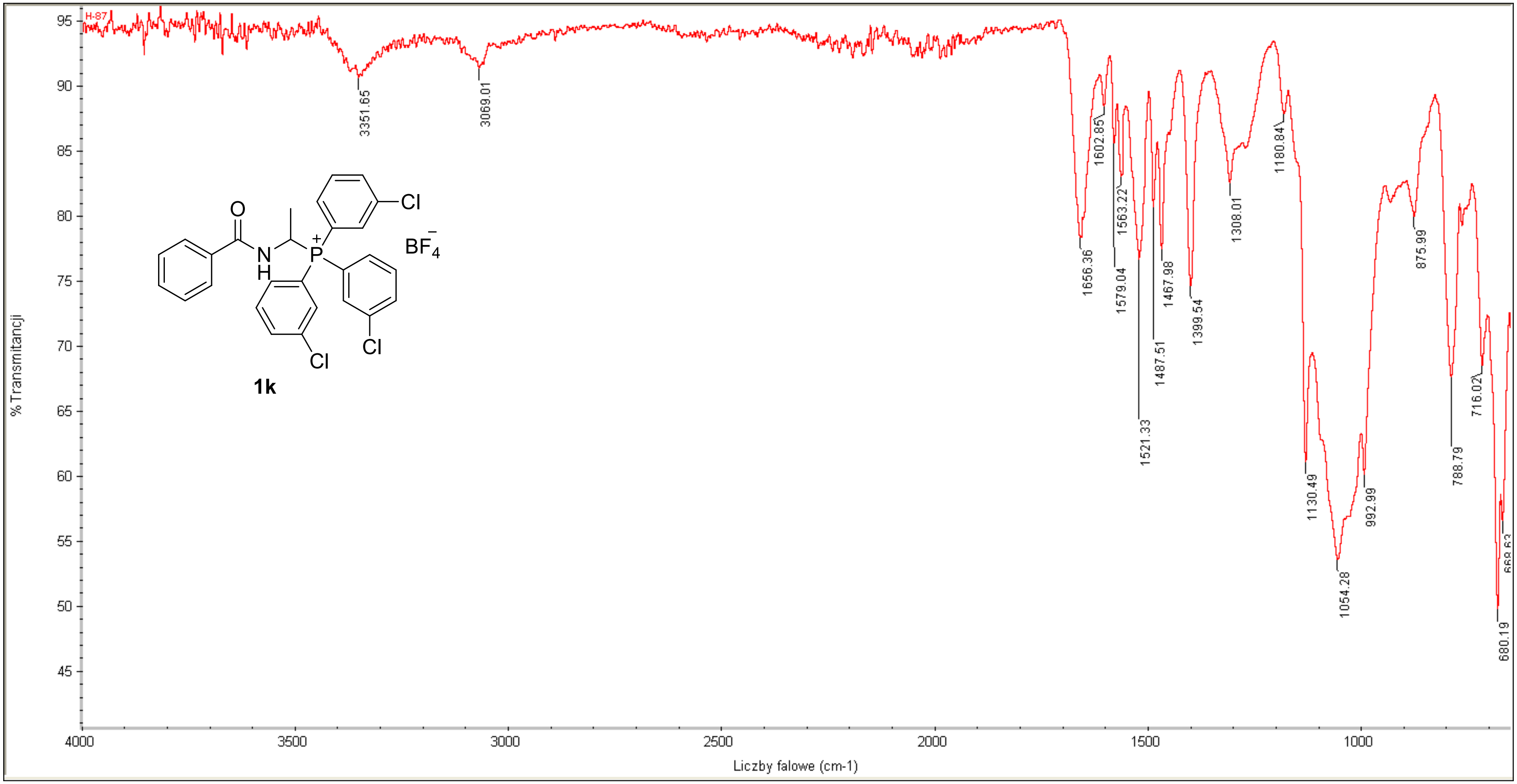

IR spectrum of 1-(*N*-benzoylamino)ethyltris(3-chlorophenyl)phosphonium tetrafluoroborate (**1k**); ATR (cm^{-1}).

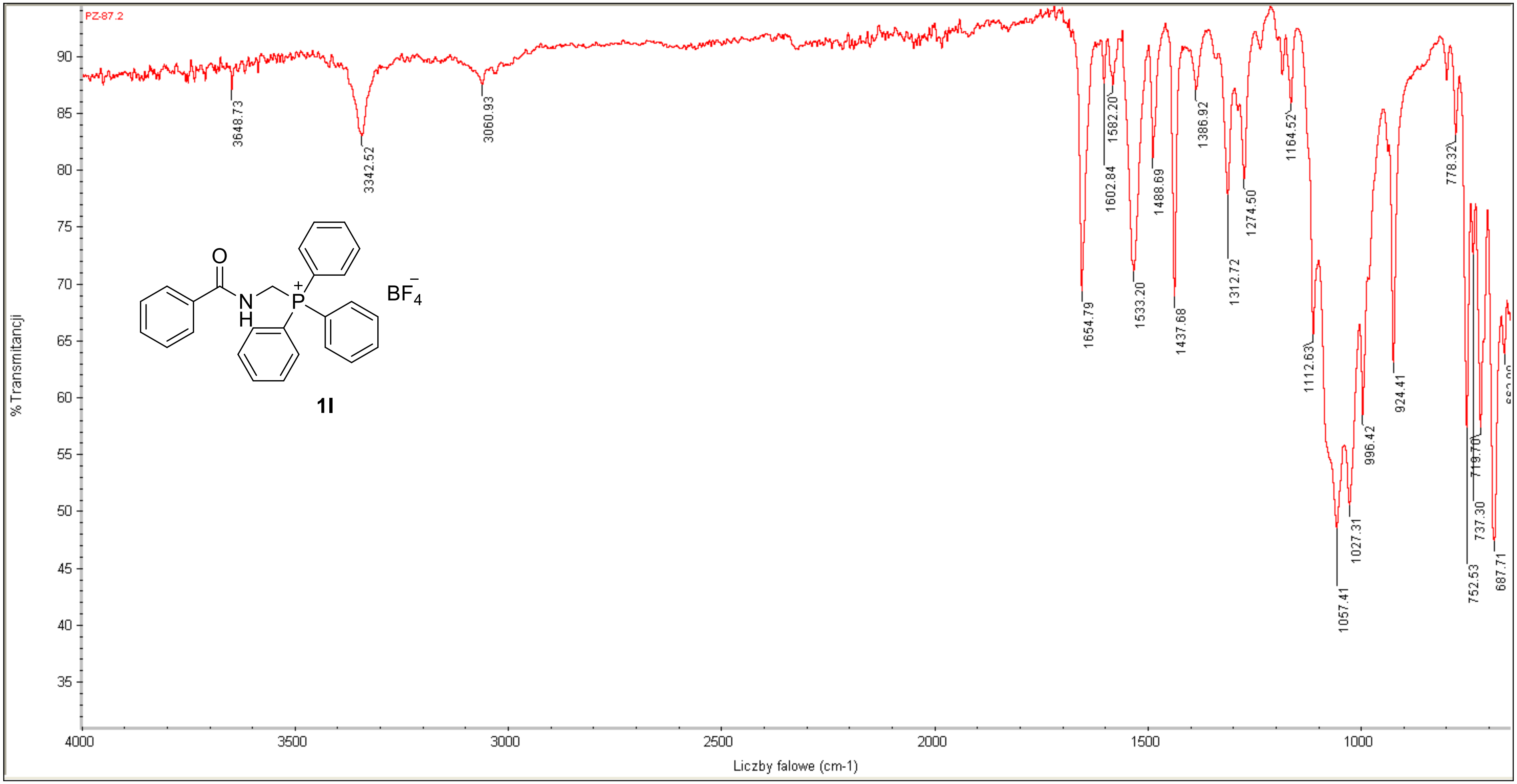

IR spectrum of (*N*-benzoylamino)methyltriphenylphosphonium tetrafluoroborate (**1l**); ATR (cm^{-1}).

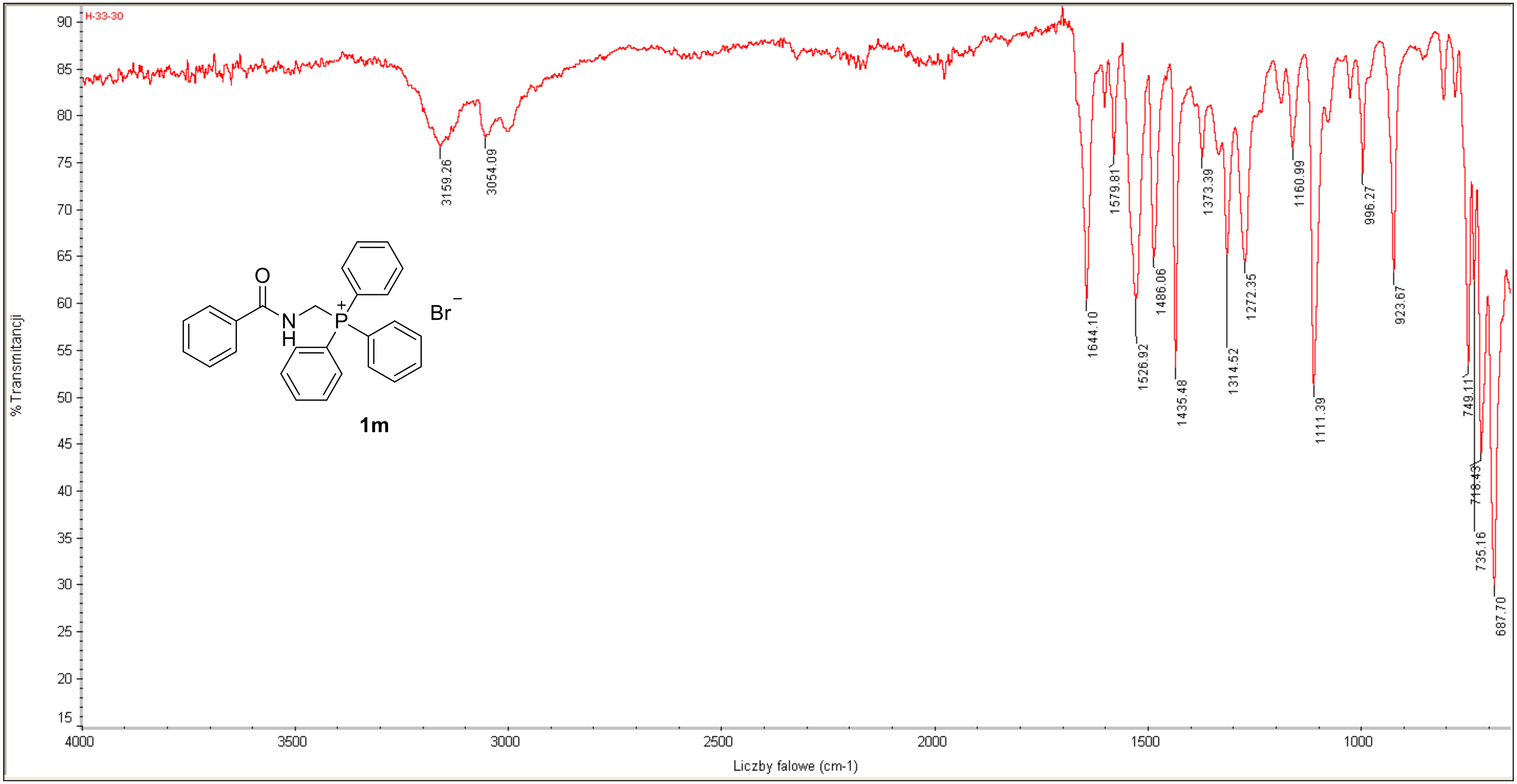

IR spectrum of (*N*-benzoylamino)methyltriphenylphosphonium bromide (**1m**); ATR (cm^{-1}).

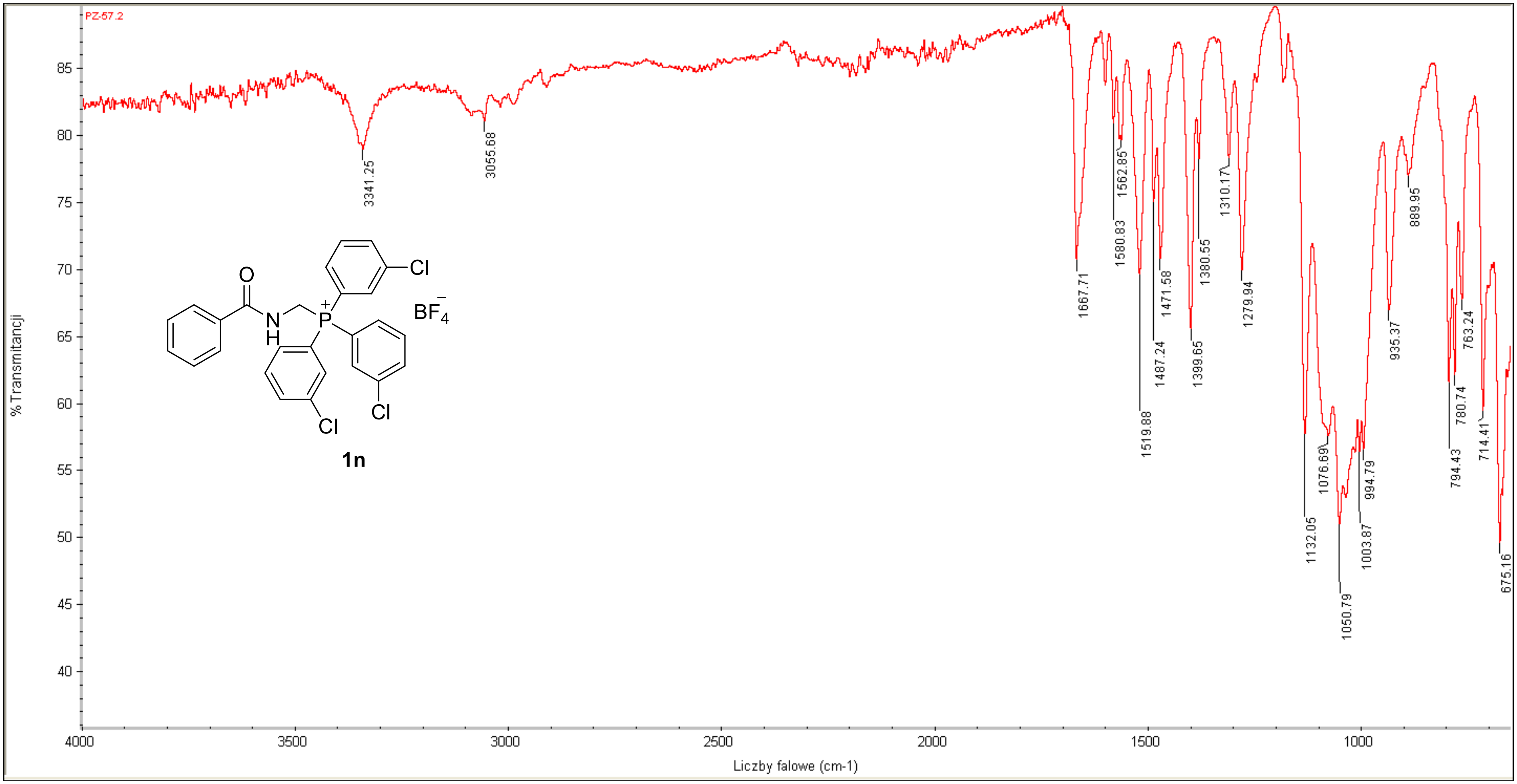

IR spectrum of (*N*-benzoylamino)methyltris(3-chlorophenyl)phosphonium tetrafluoroborate (**1n**); ATR (cm^{-1}).

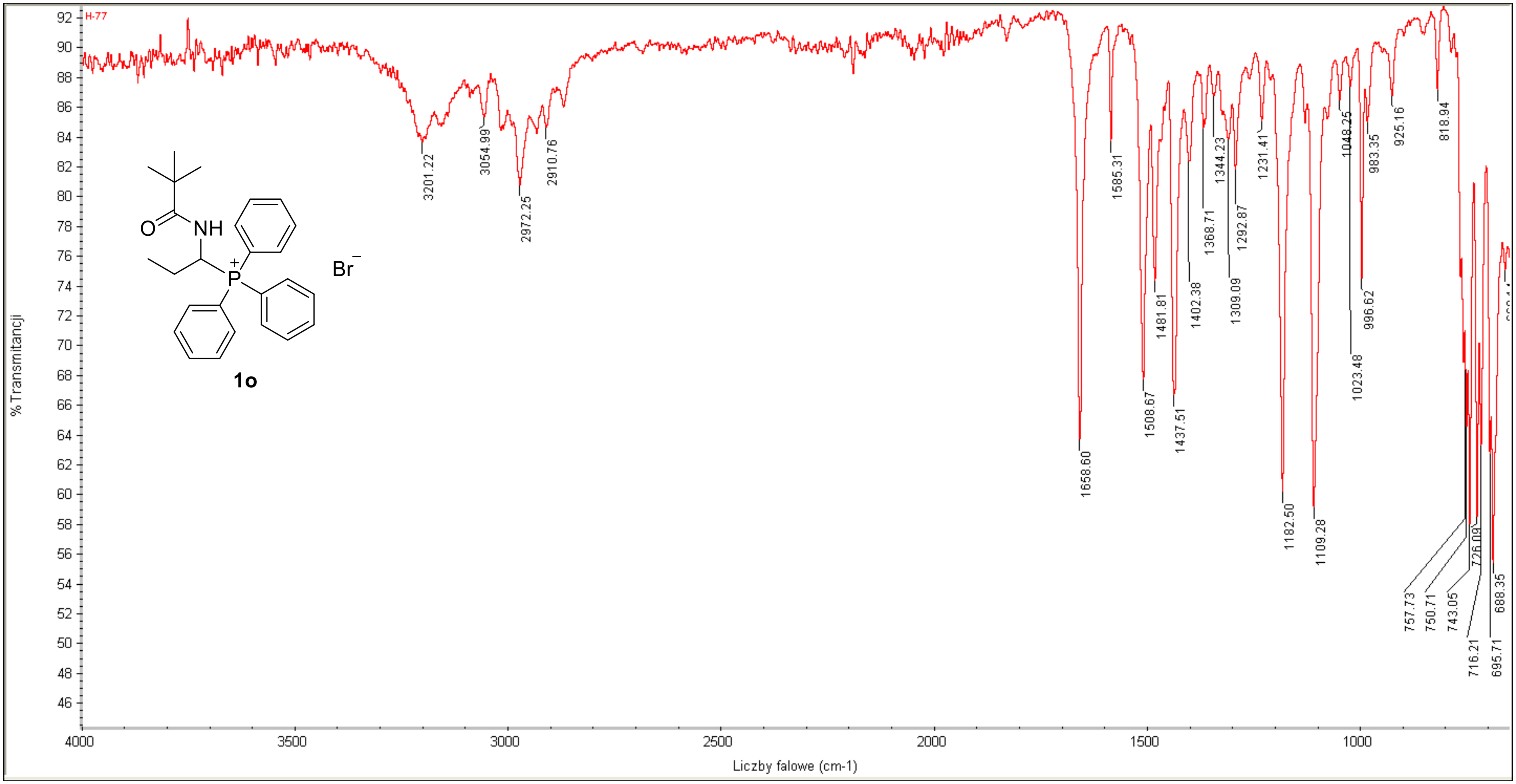

IR spectrum of 1-(*N*-pivaloylamino)propyltriphenylphosphonium bromide (**1o**); ATR (cm^{-1}).

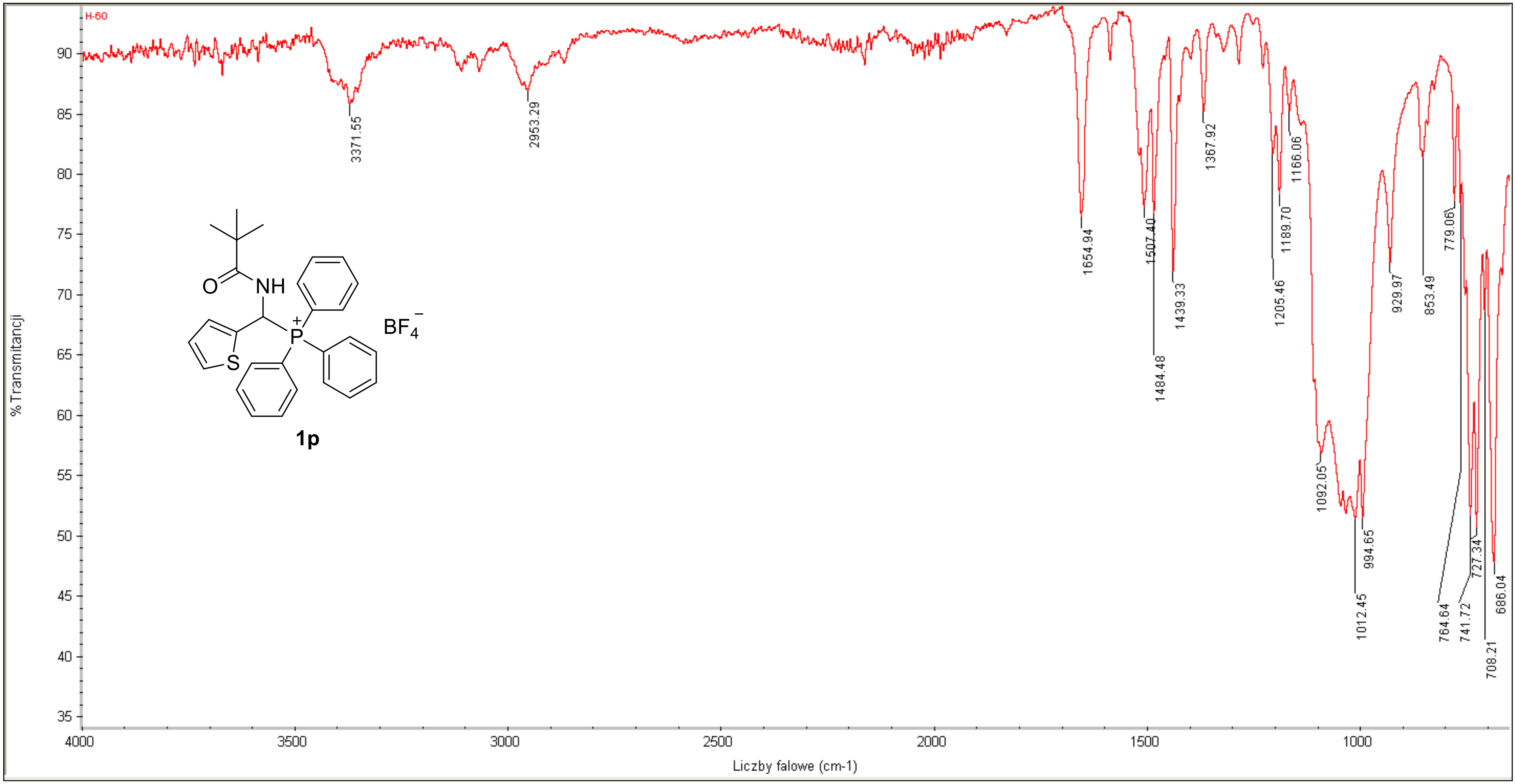

IR spectrum of 1-(*N*-pivaloylamino)-1-(2-thienyl)methyltriphenylphosphonium tetrafluoroborate (**1p**); ATR (cm^{-1}).

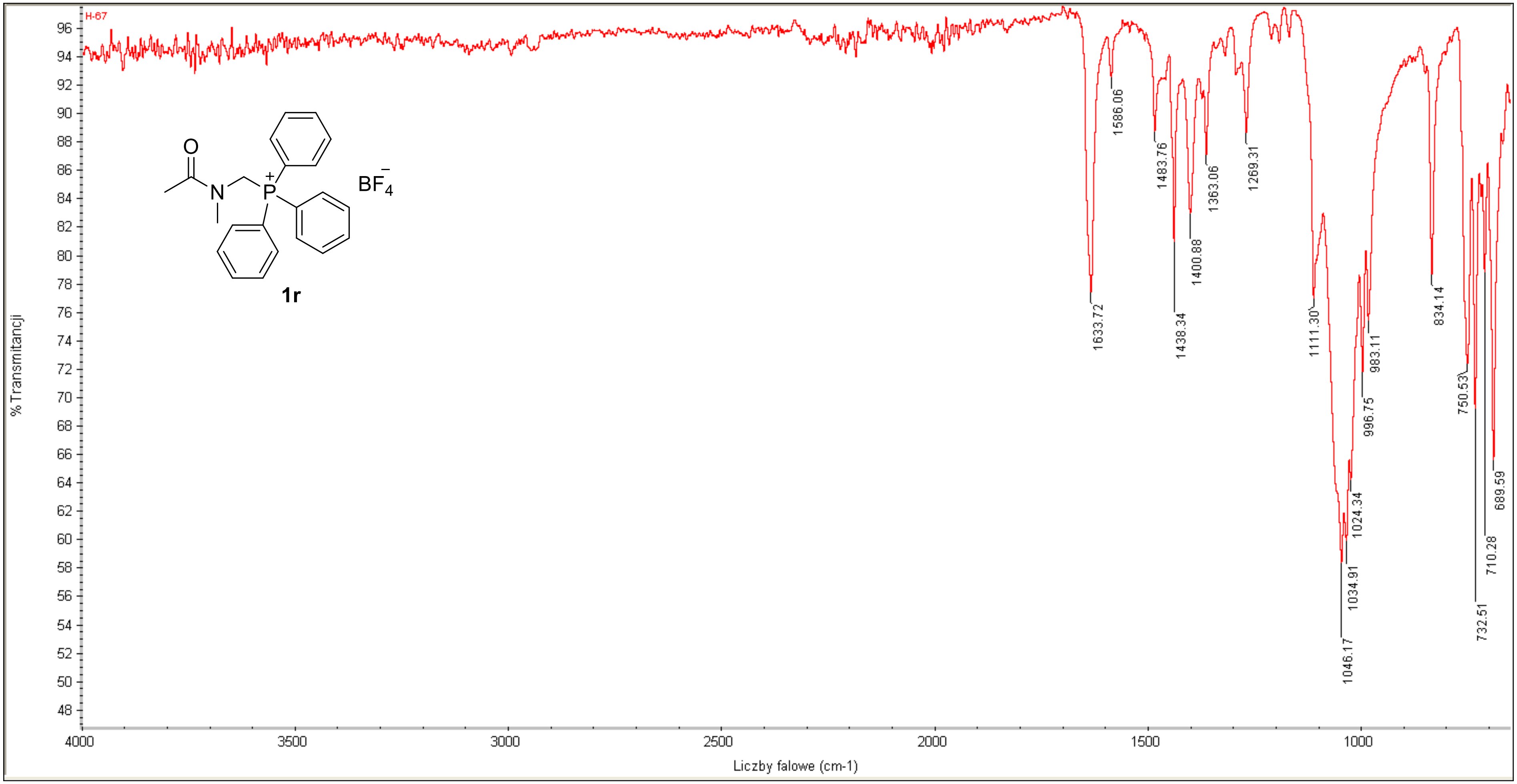

IR spectrum of *N*-(*N*-methylacetylamino)methyltriphenylphosphonium tetrafluoroborate (**1r**); ATR (cm^{-1}).

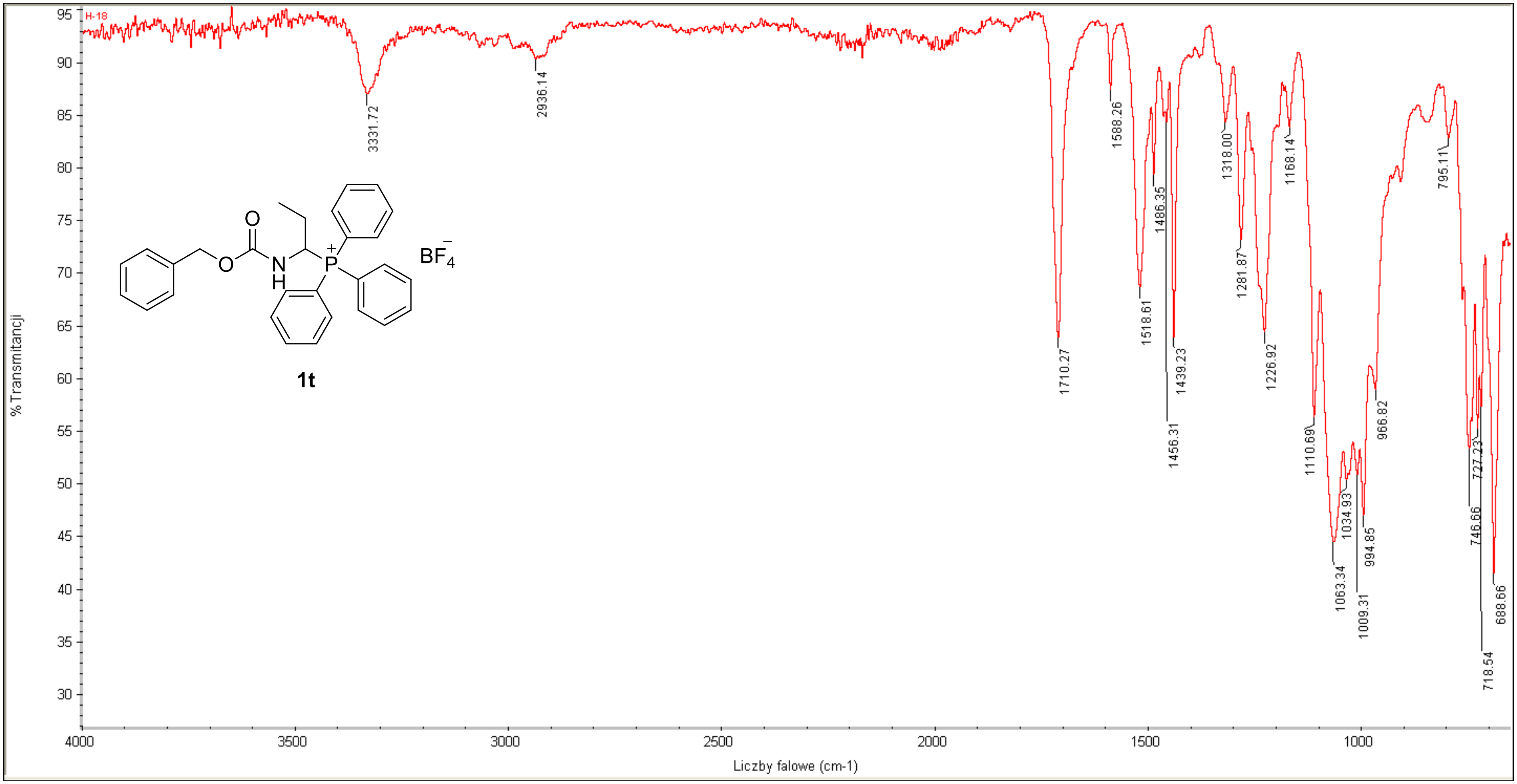

IR spectrum of 1-(*N*-benzyloxycarbonylamino)propyltriphenylphosphonium tetrafluoroborate (**1t**); ATR (cm^{-1}).

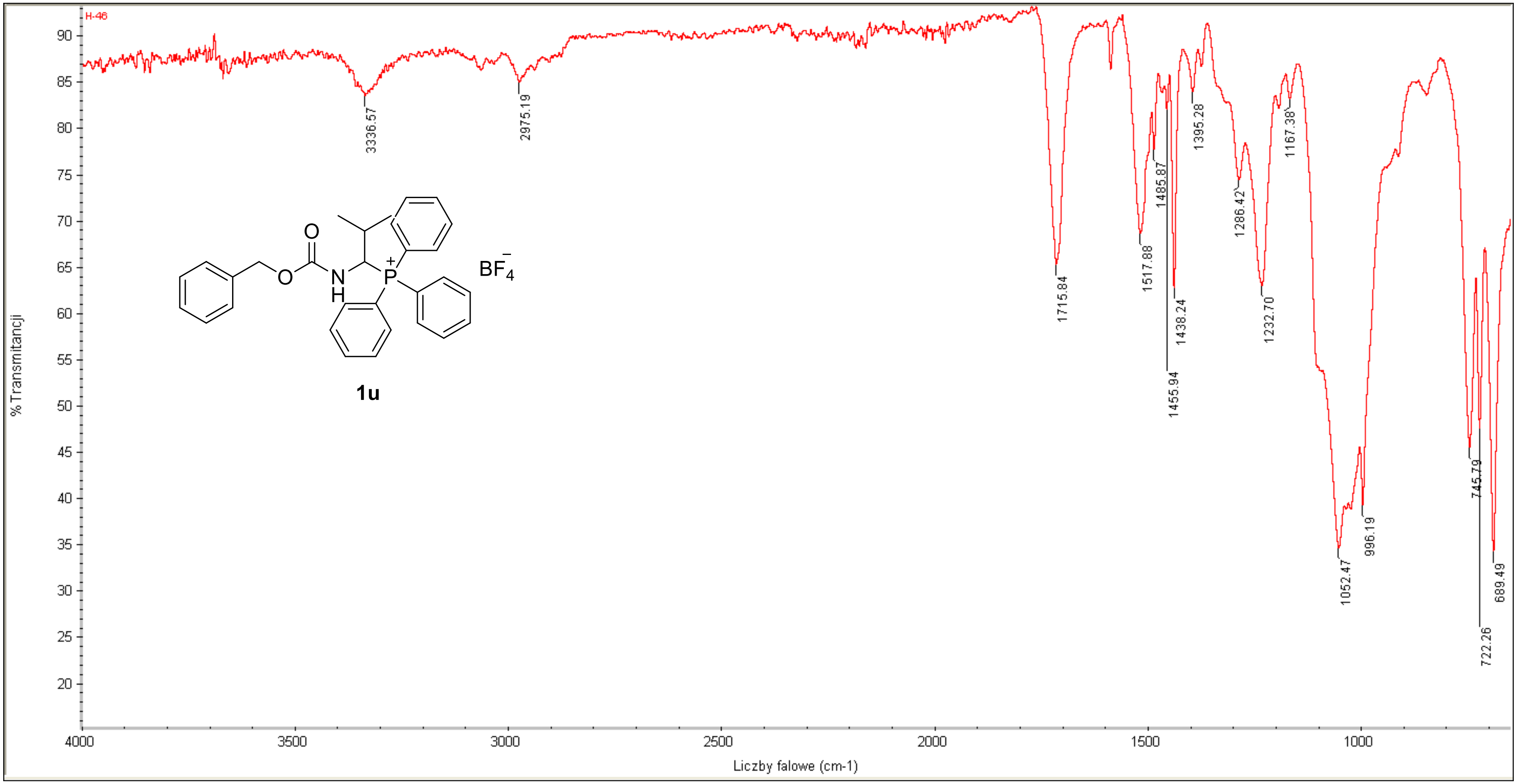

IR spectrum of 1-(*N*-benzyloxycarbonylamino)-2-methylpropyltriphenylphosphonium tetrafluoroborate (**1u**); ATR (cm^{-1}).

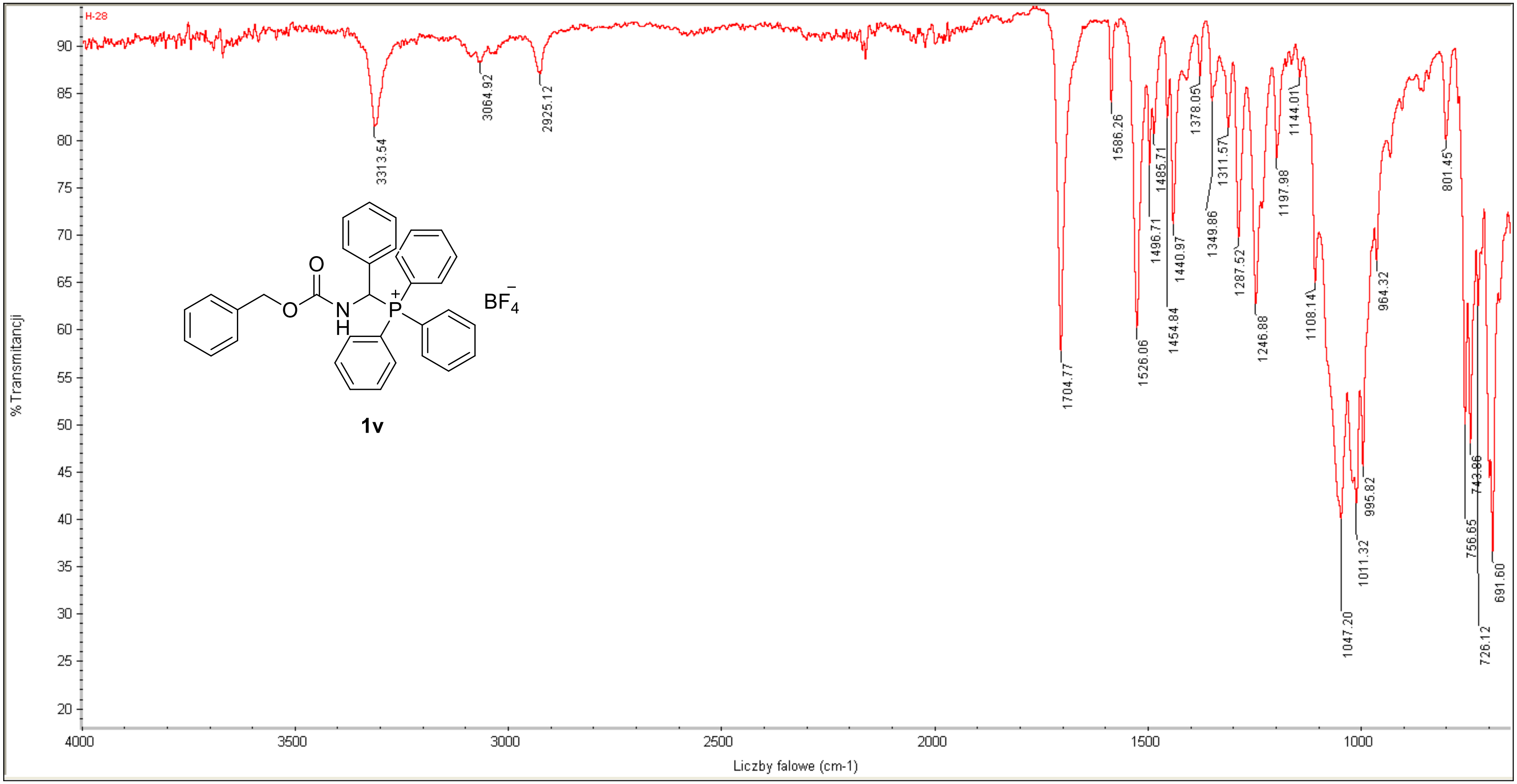

IR spectrum of (*N*-benzyloxycarbonylamino)phenylmethyltriphenylphosphonium tetrafluoroborate (**1v**); ATR (cm^{-1}).

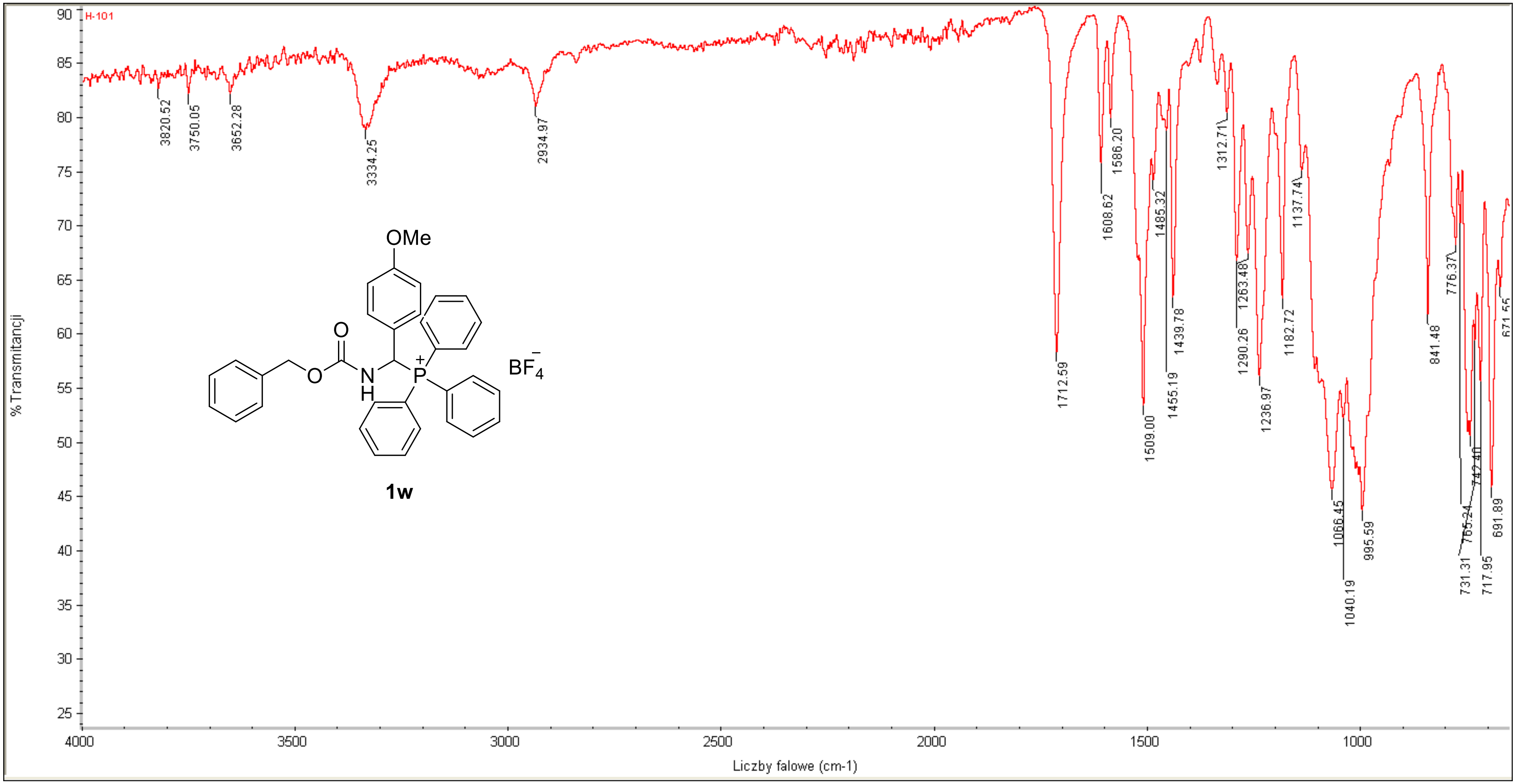

IR spectrum of 1-(*N*-benzyloxycarbonylamino)-1-(4-methoxyphenyl)methyltriphenylphosphonium tetrafluoroborate (**1w**); ATR (cm^{-1}).

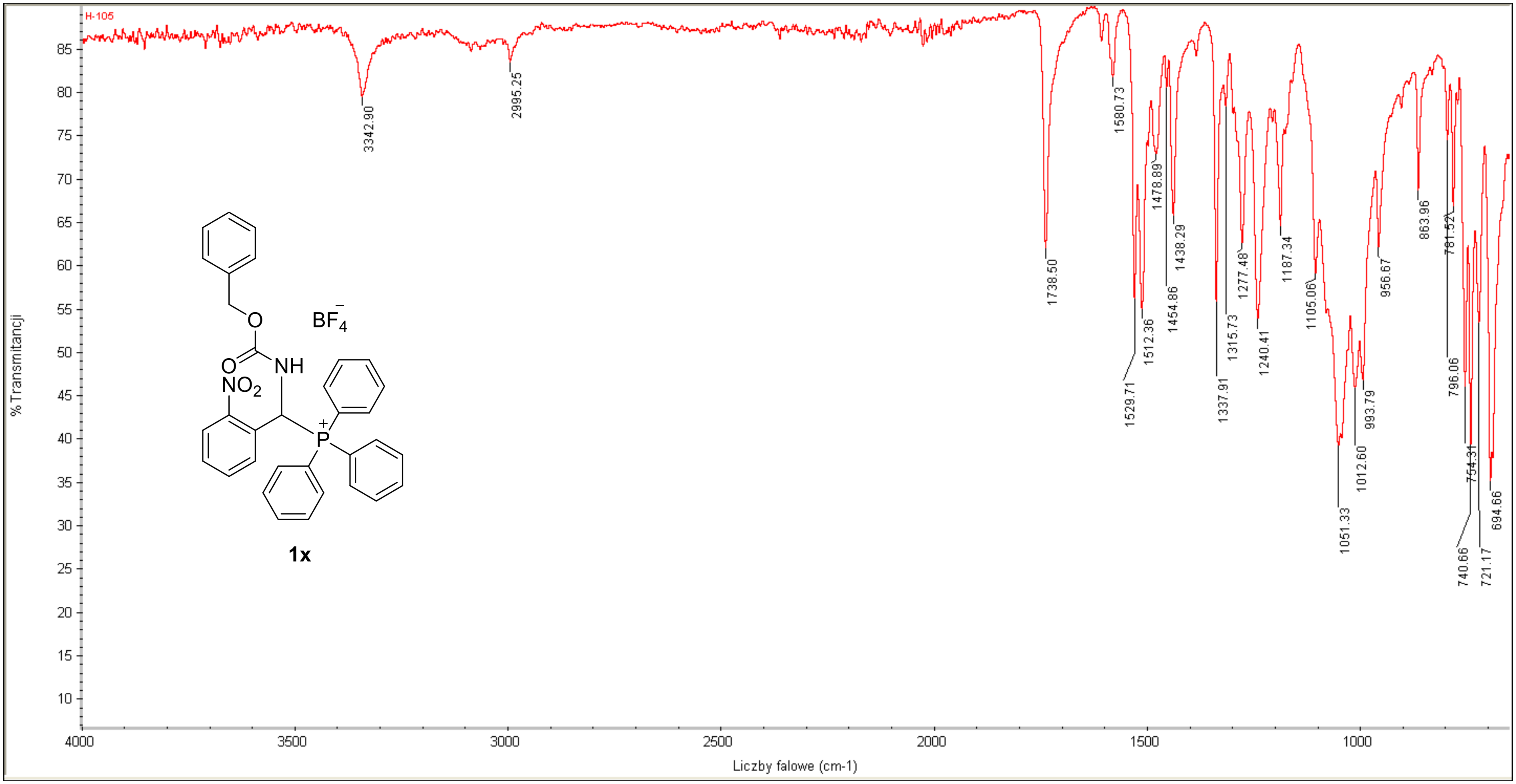

IR spectrum of 1-(*N*-benzyloxycarbonylamino)-1-(2-nitrophenyl)methyltriphenylphosphonium tetrafluoroborate (**1x**); ATR (cm^{-1}).

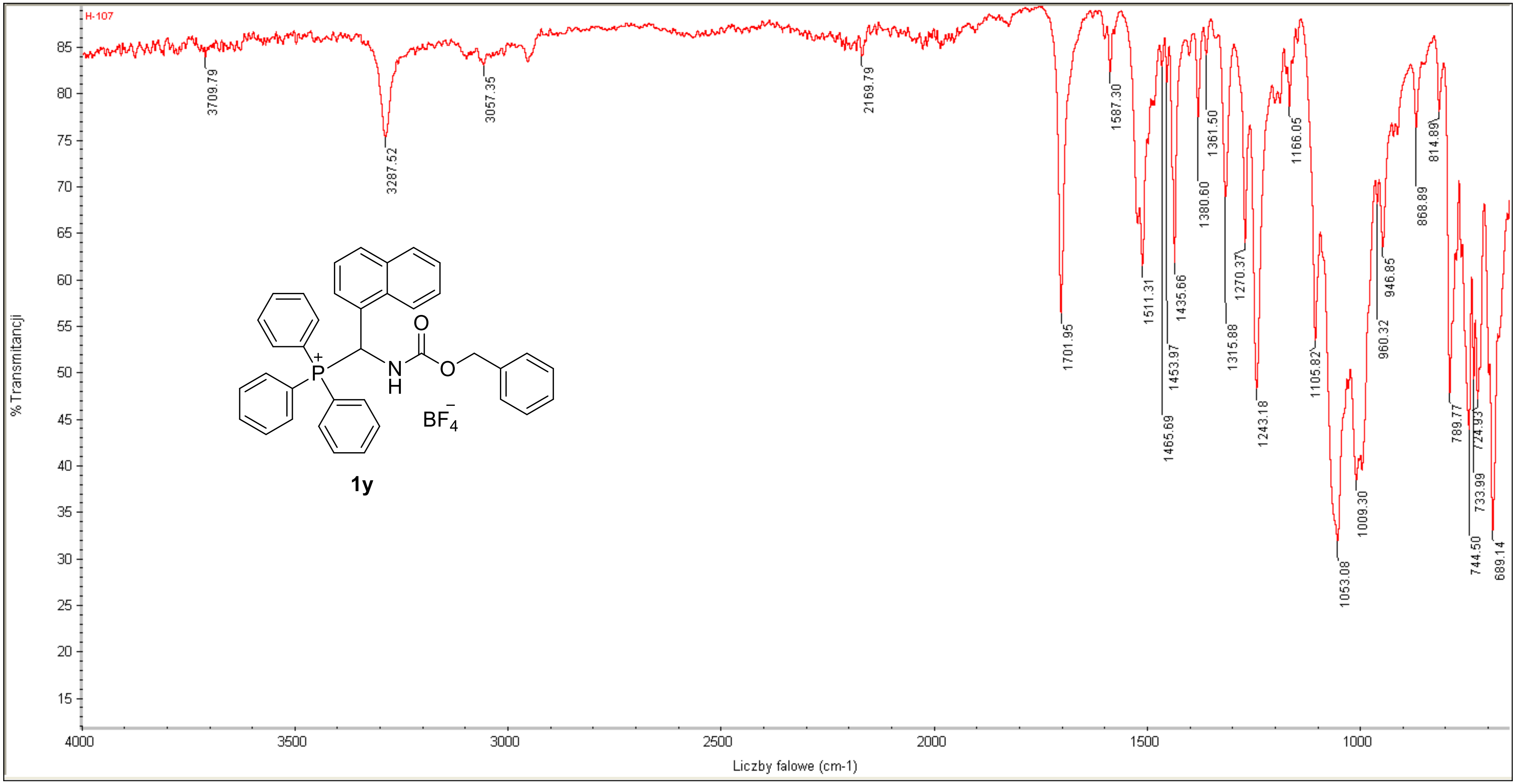

IR spectrum of 1-(*N*-benzyloxycarbonylamino)-1-(1-naphthyl)methyltriphenylphosphonium tetrafluoroborate (**1y**); ATR (cm^{-1}).

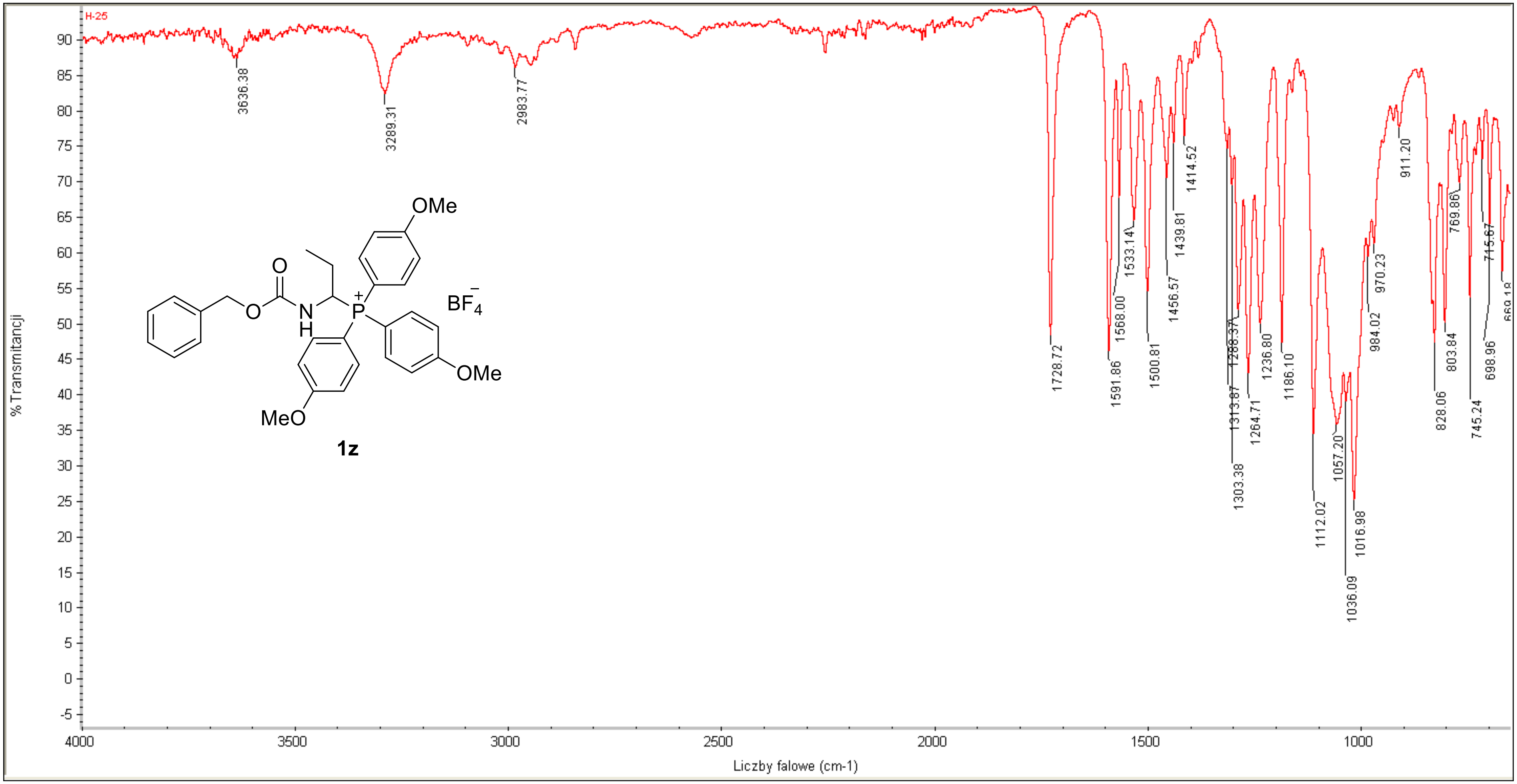

IR spectrum of 1-(*N*-benzyloxycarbonylamino)propyltris(4-methoxyphenyl)phosphonium tetrafluoroborate (**1z**); ATR (cm^{-1}).

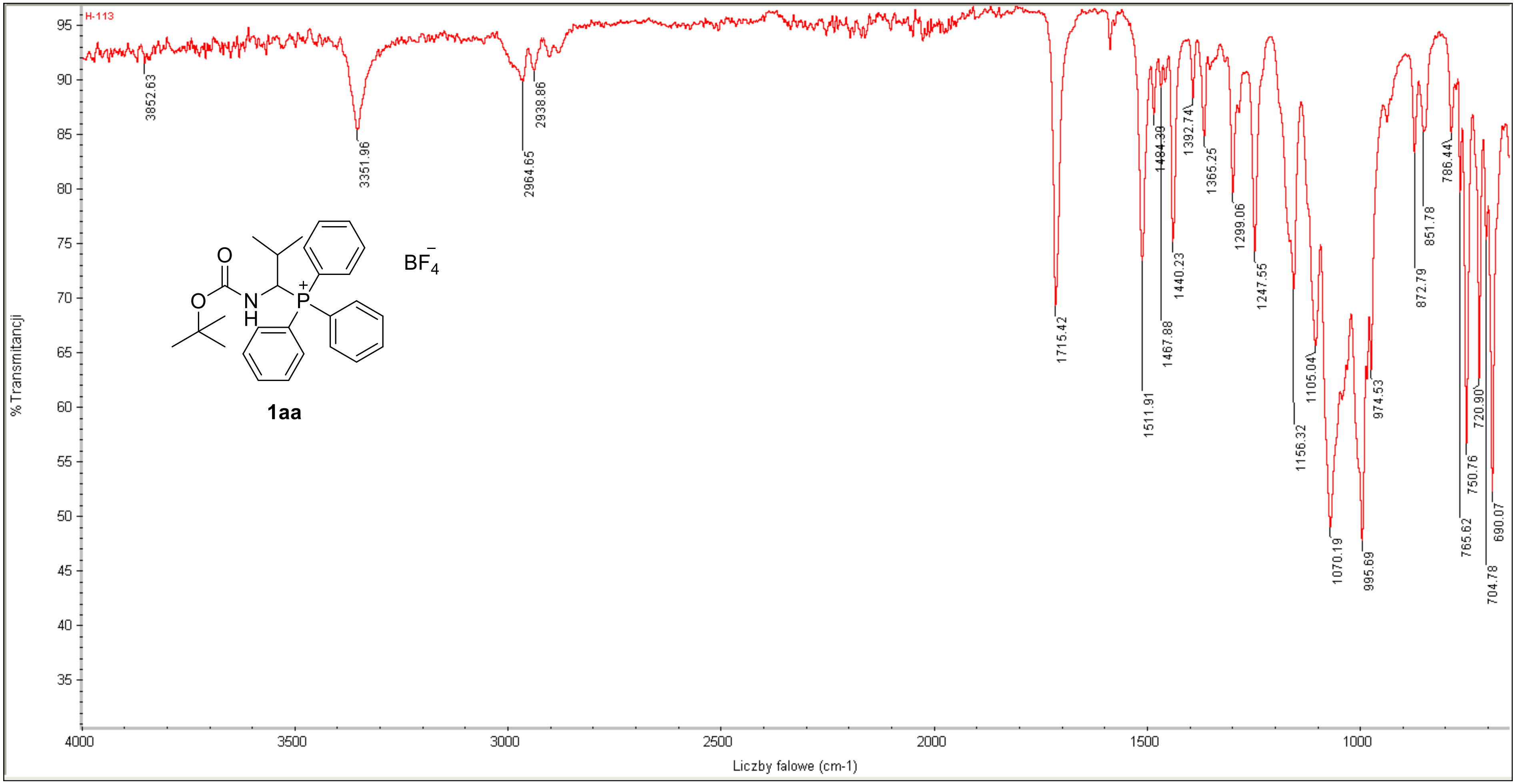

IR spectrum of 1-(*N*-tert-butoxycarbonylamino)-2-methylpropyltriphenylphosphonium tetrafluoroborate (**1aa**); ATR (cm^{-1}).

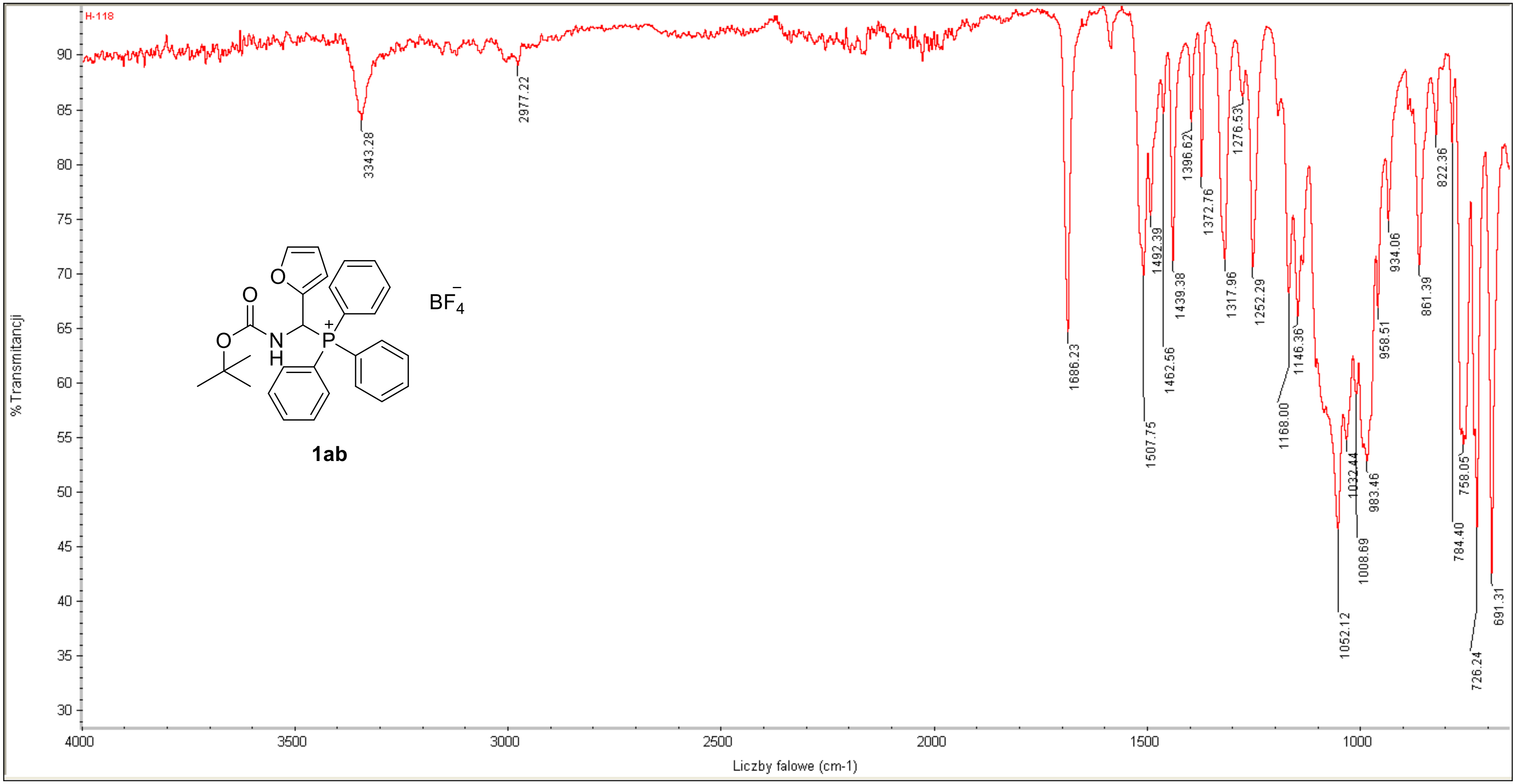

IR spectrum of 1-(*N*-tert-butoxycarbonylamino)-1-(2-furyl)methyltriphenylphosphonium tetrafluoroborate (**1ab**); ATR (cm^{-1}).

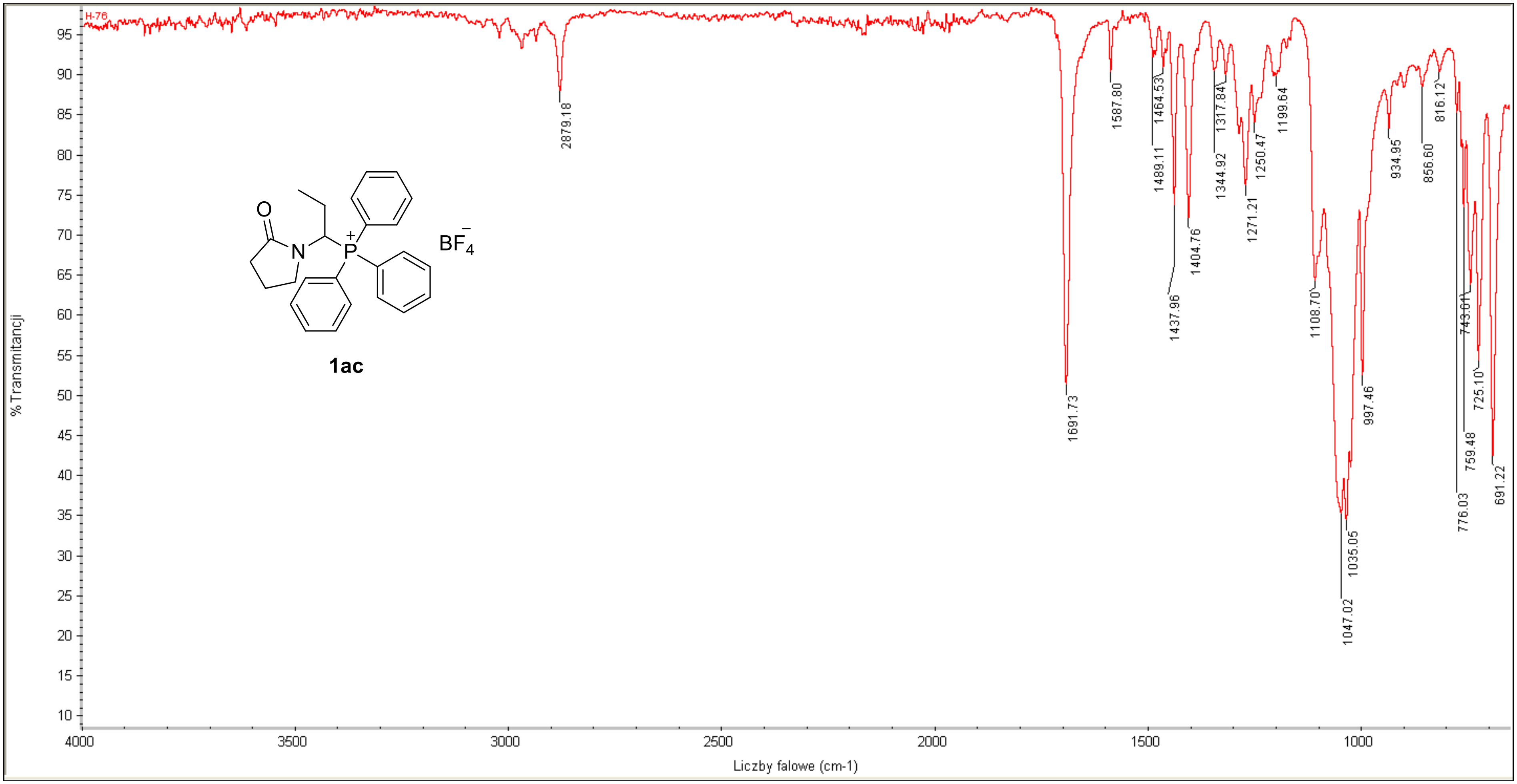

IR spectrum of 1-(2-oxopyrrolidin-1-yl)propyltriphenylphosphonium tetrafluoroborate (**1ac**); ATR (cm^{-1}).

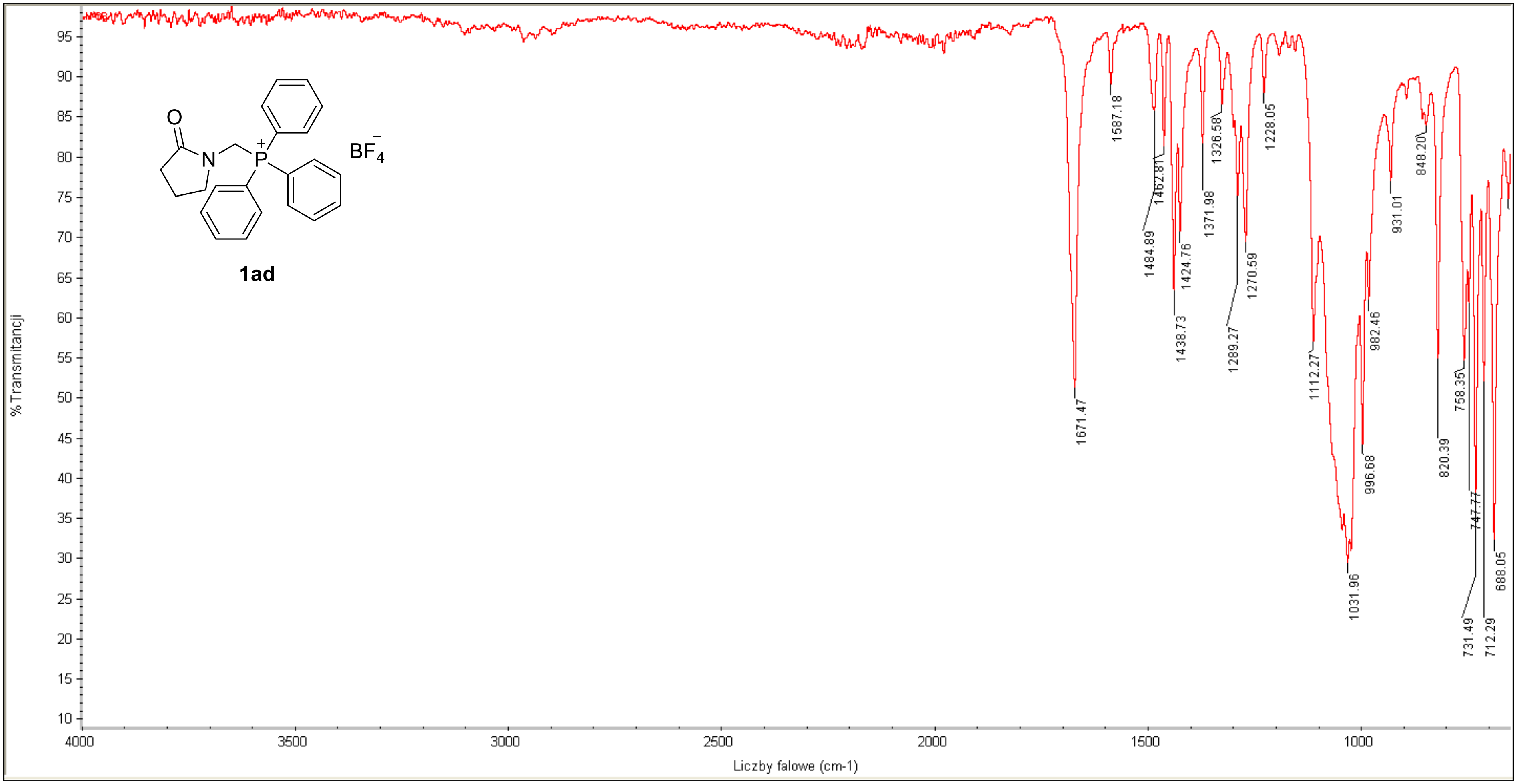

IR spectrum of 1-(2-oxopyrrolidin-1-yl)methyltriphenylphosphonium tetrafluoroborate (**1ad**); ATR (cm^{-1}).

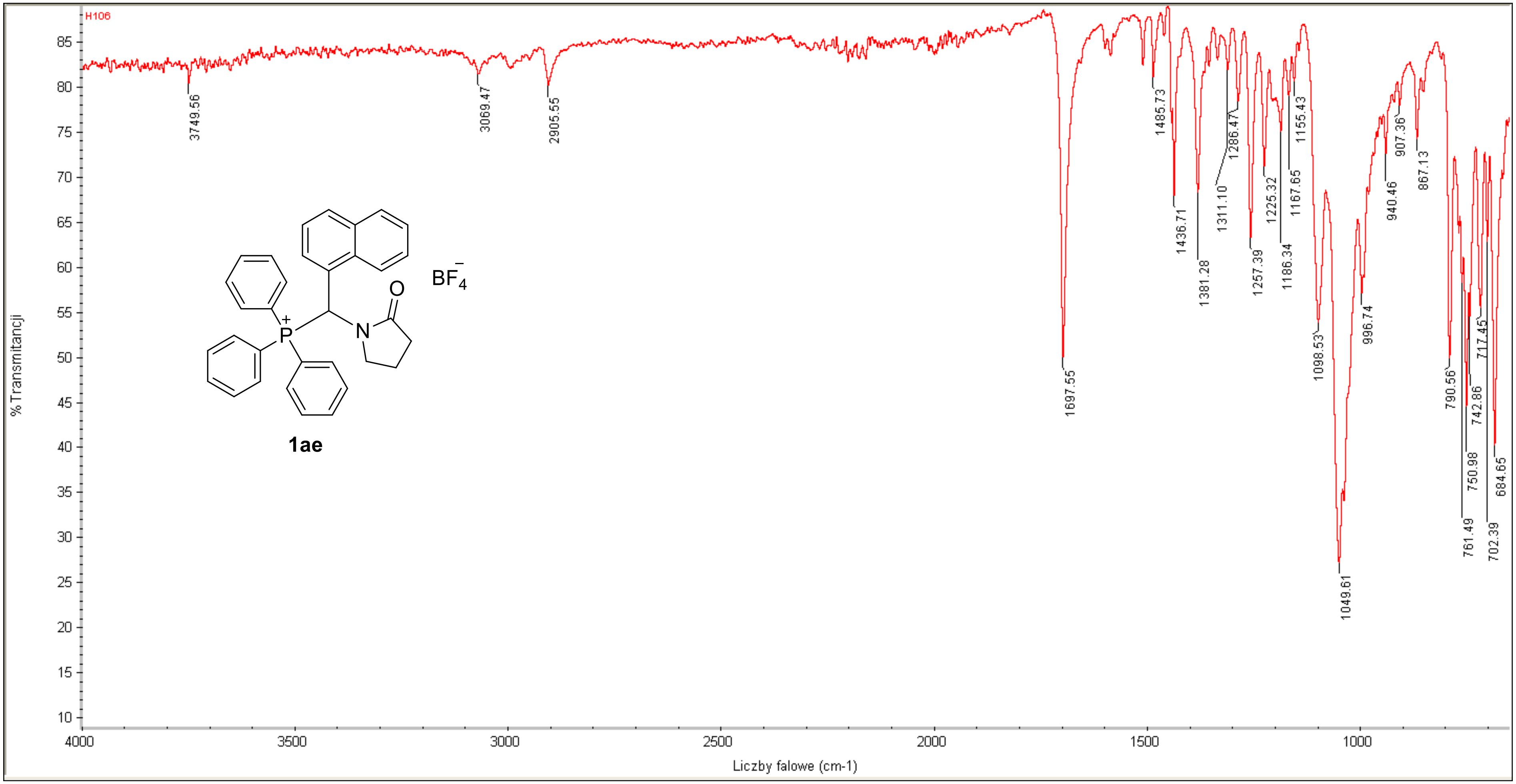

IR spectrum of 1-(2-oxopyrrolidin-1-yl)-1-(1-naphthyl)methyltriphenylphosphonium tetrafluoroborate (**1ae**); ATR (cm^{-1}).

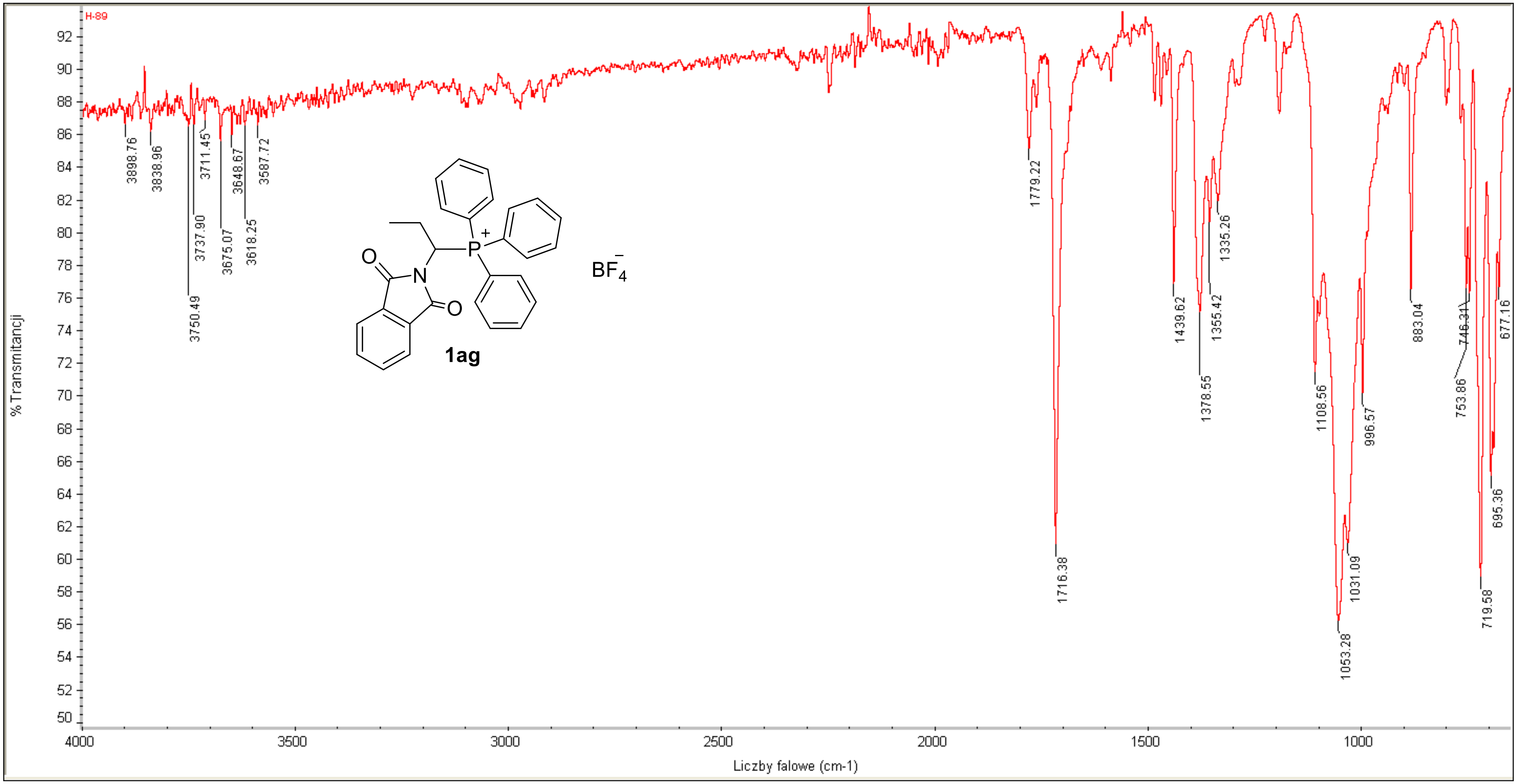

IR spectrum of 1-(*N*-phthalimido)propyltriphenylphosphonium tetrafluoroborate (**1ag**); ATR (cm^{-1}).

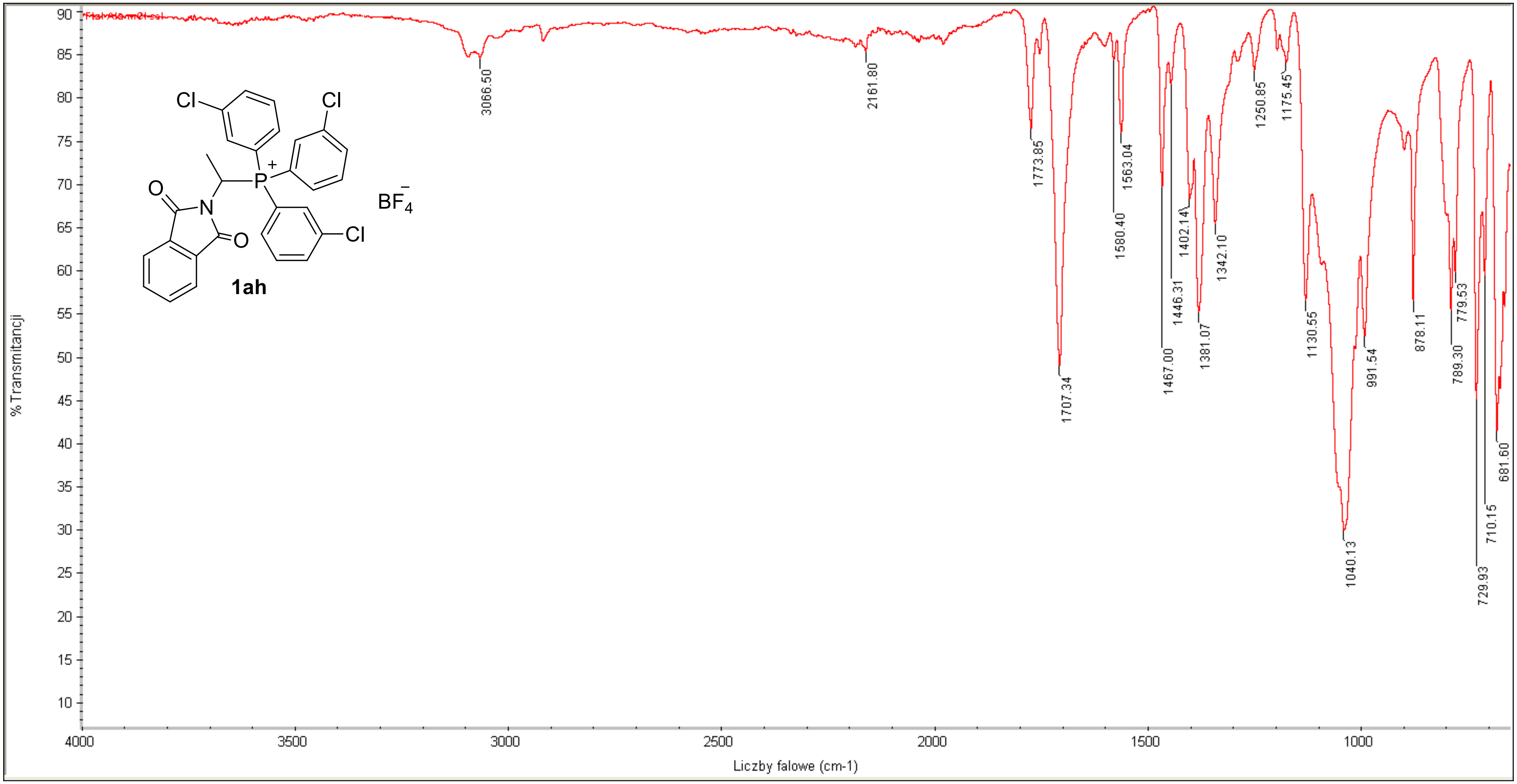

IR spectrum of 1-(*N*-phthalimido)ethyltris(3-chlorophenyl)phosphonium tetrafluoroborate (**1ah**); ATR (cm^{-1}).

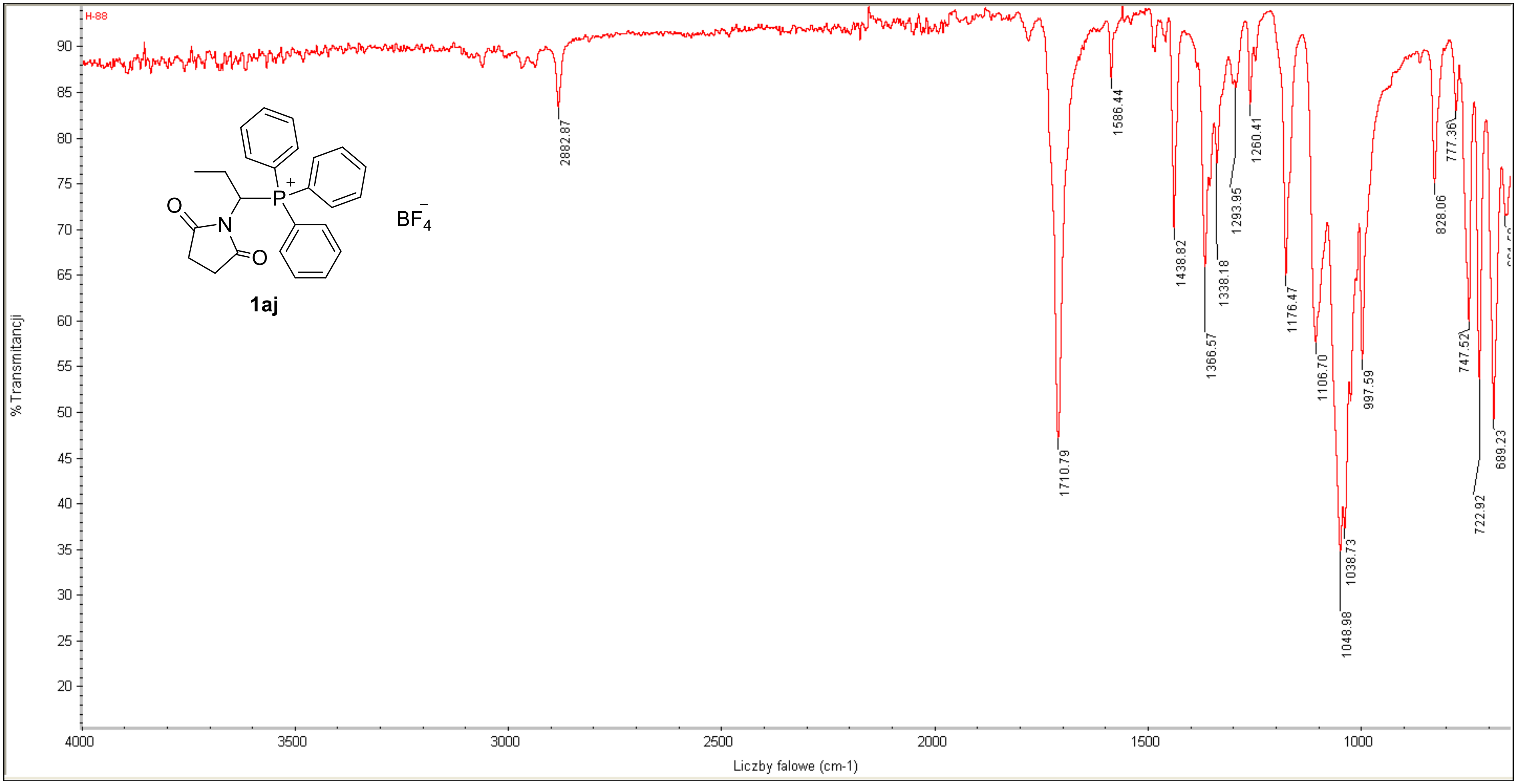

IR spectrum of 1-(*N*-succinimido)propyltriphenylphosphonium tetrafluoroborate (**1aj**); ATR (cm^{-1}).

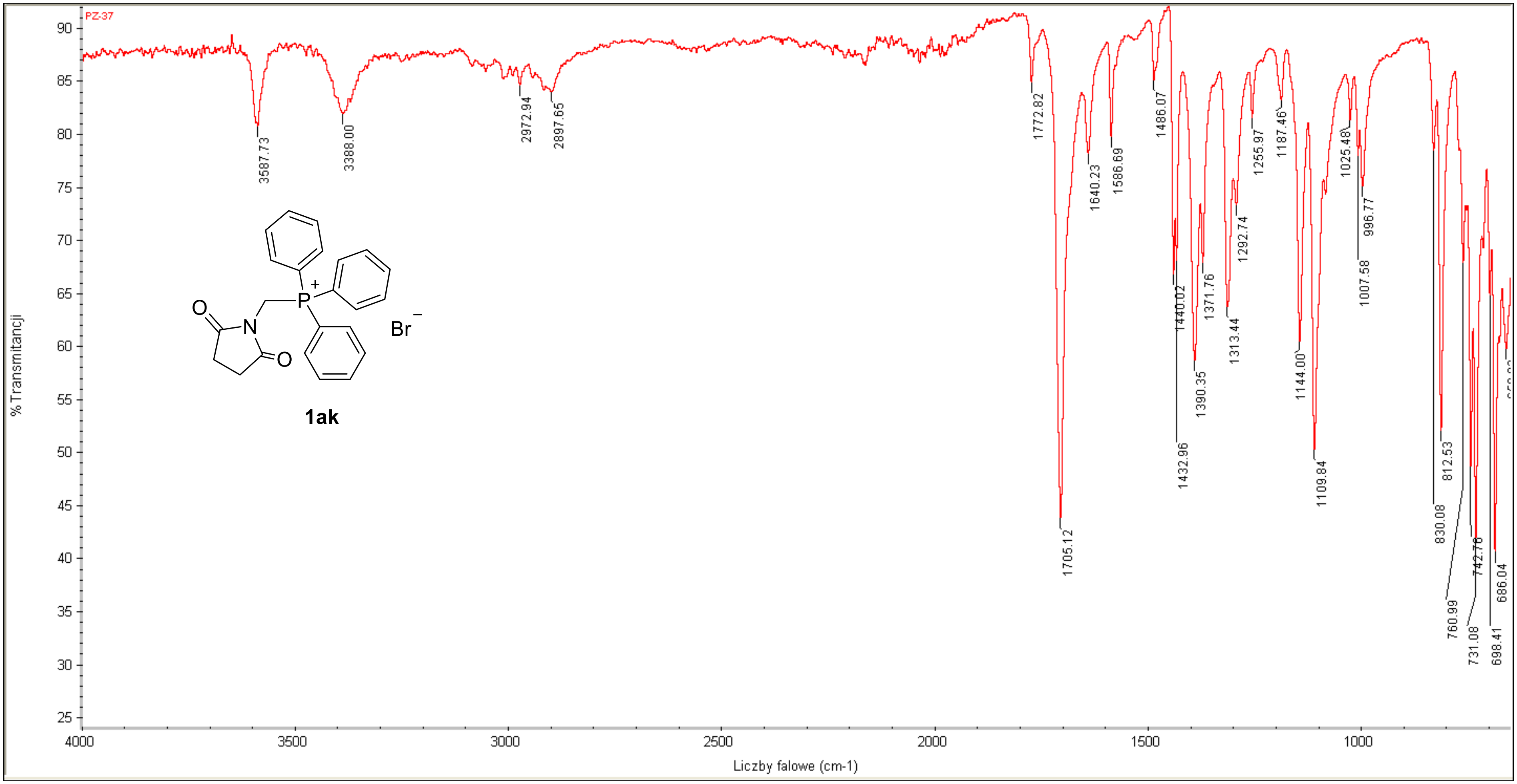

IR spectrum of 1-(*N*-succinimido)methyltriphenylphosphonium bromide (**1ak**); ATR (cm^{-1}).

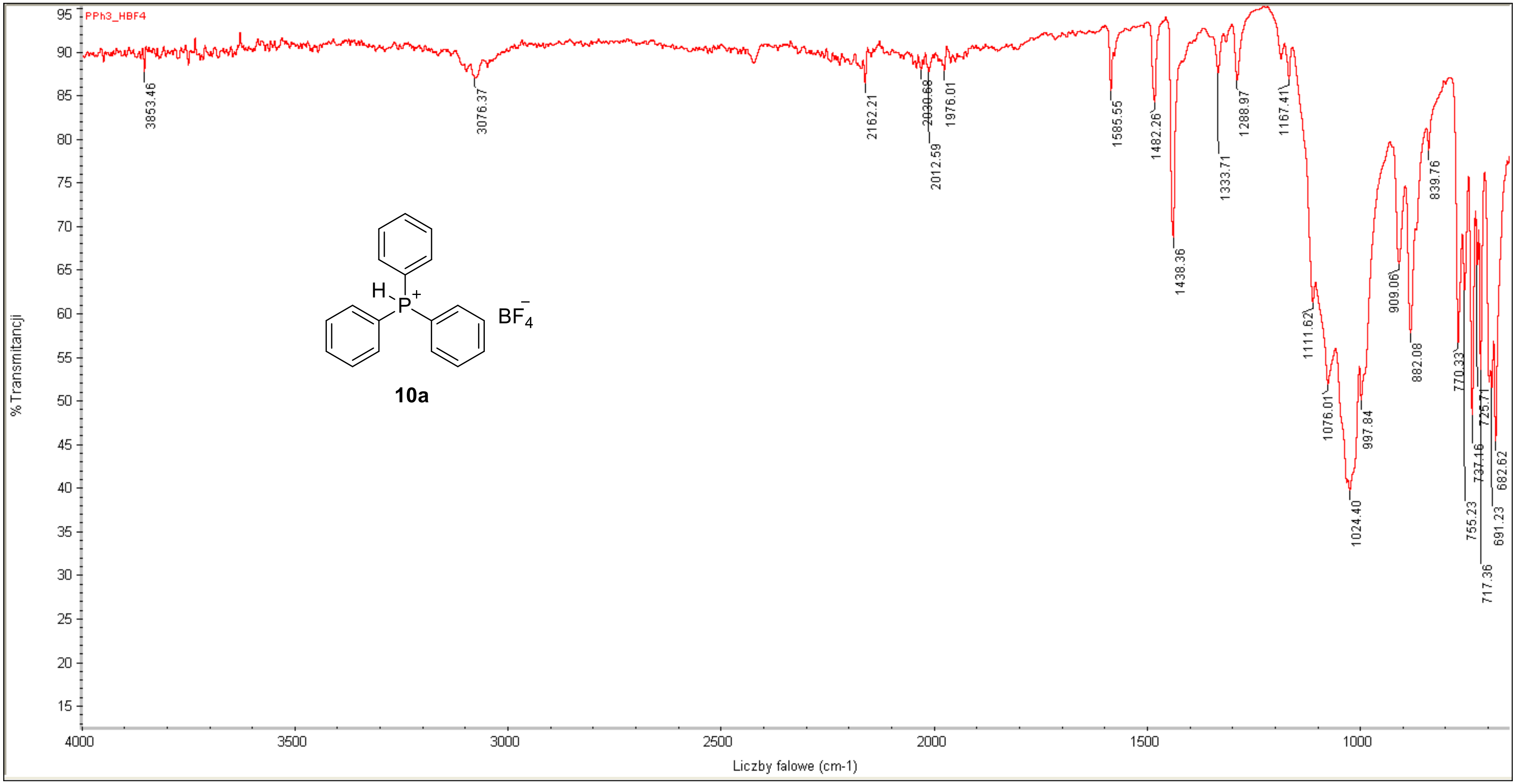

IR spectrum of triphenylphosphonium tetrafluoroborate (**10a**); ATR (cm^{-1}).

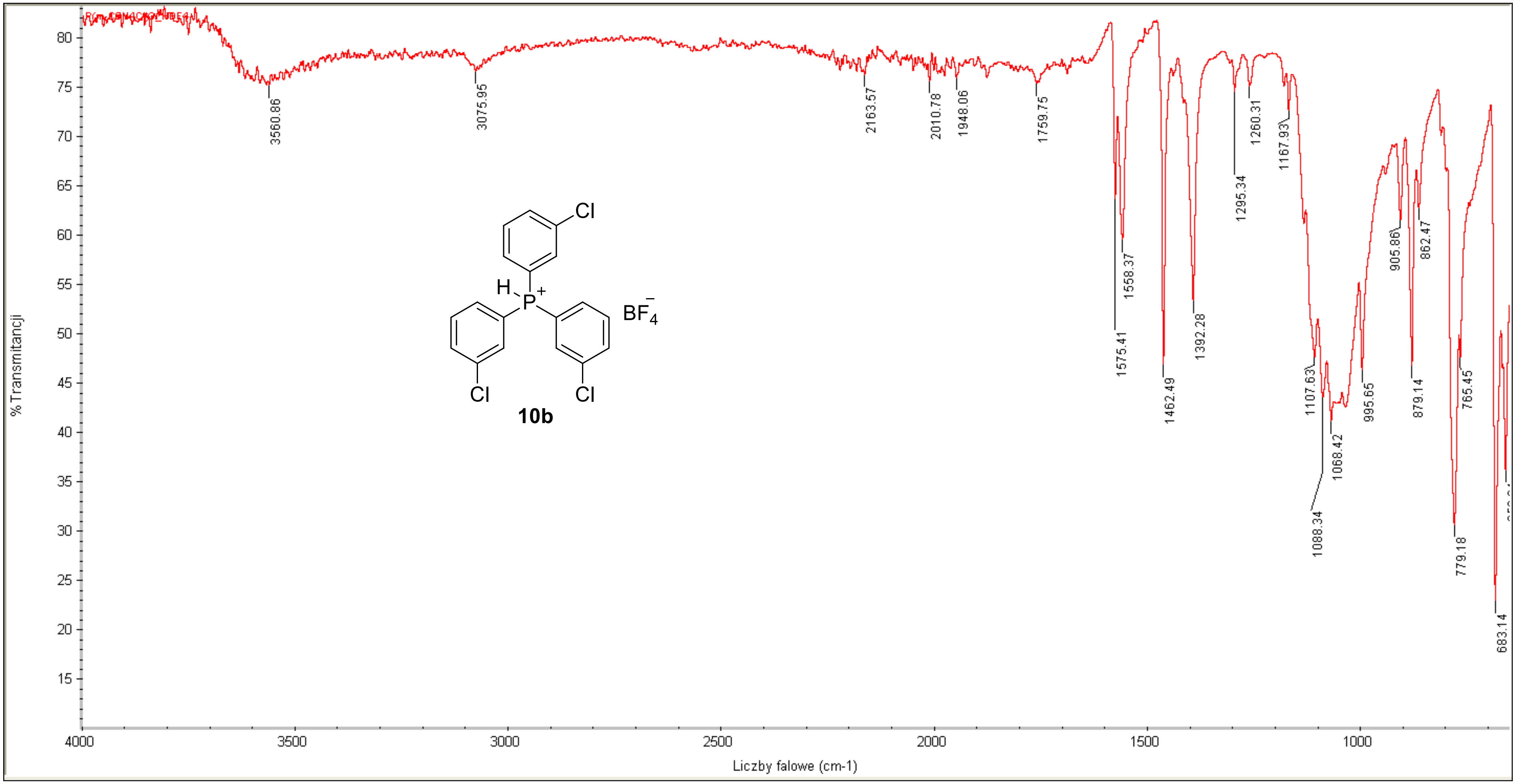

IR spectrum of tris(3-chlorophenyl)phosphonium tetrafluoroborate (**10b**); ATR (cm^{-1}).

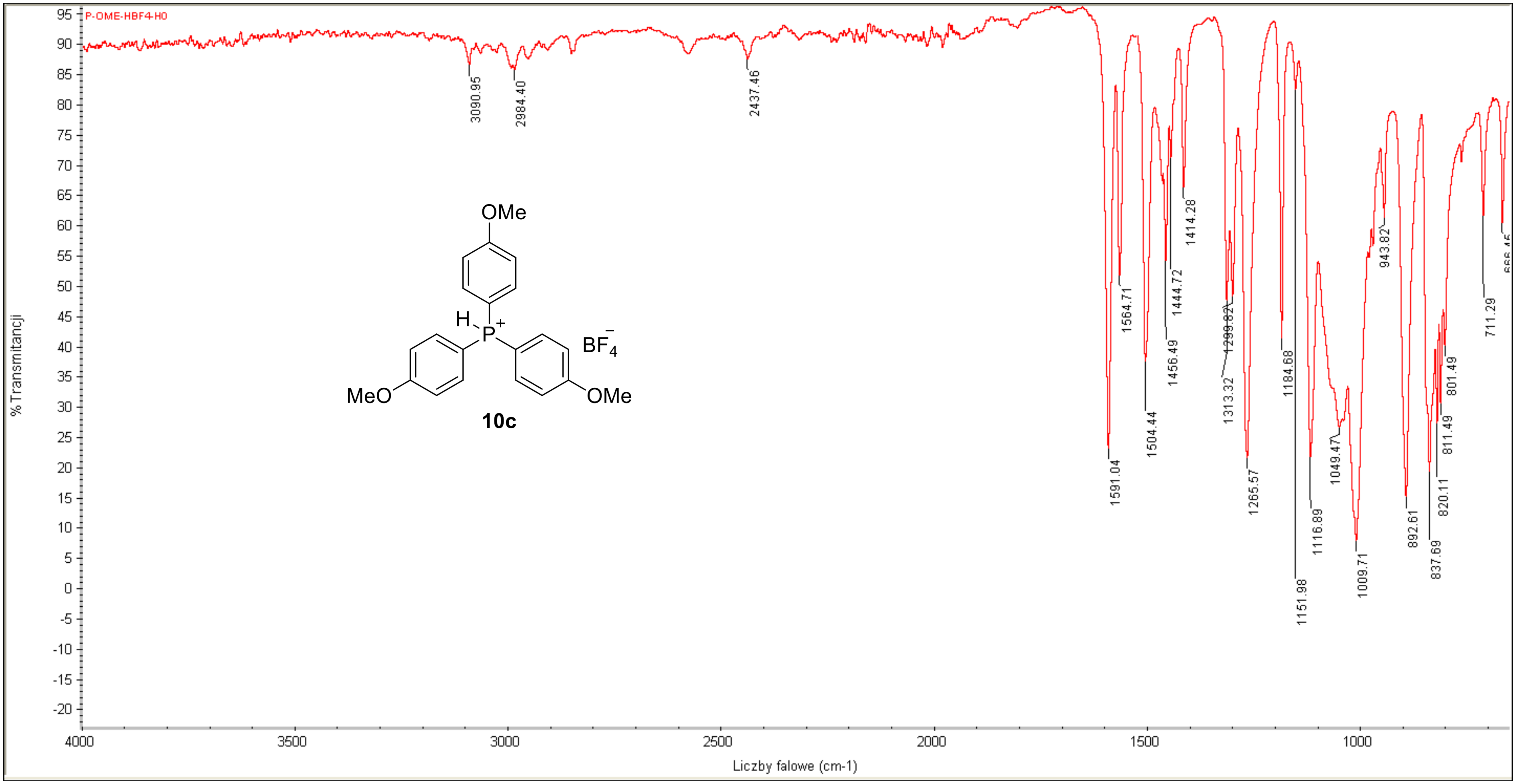

IR spectrum of tris(4-methoxyphenyl)phosphonium tetrafluoroborate (**10c**); ATR (cm^{-1}).

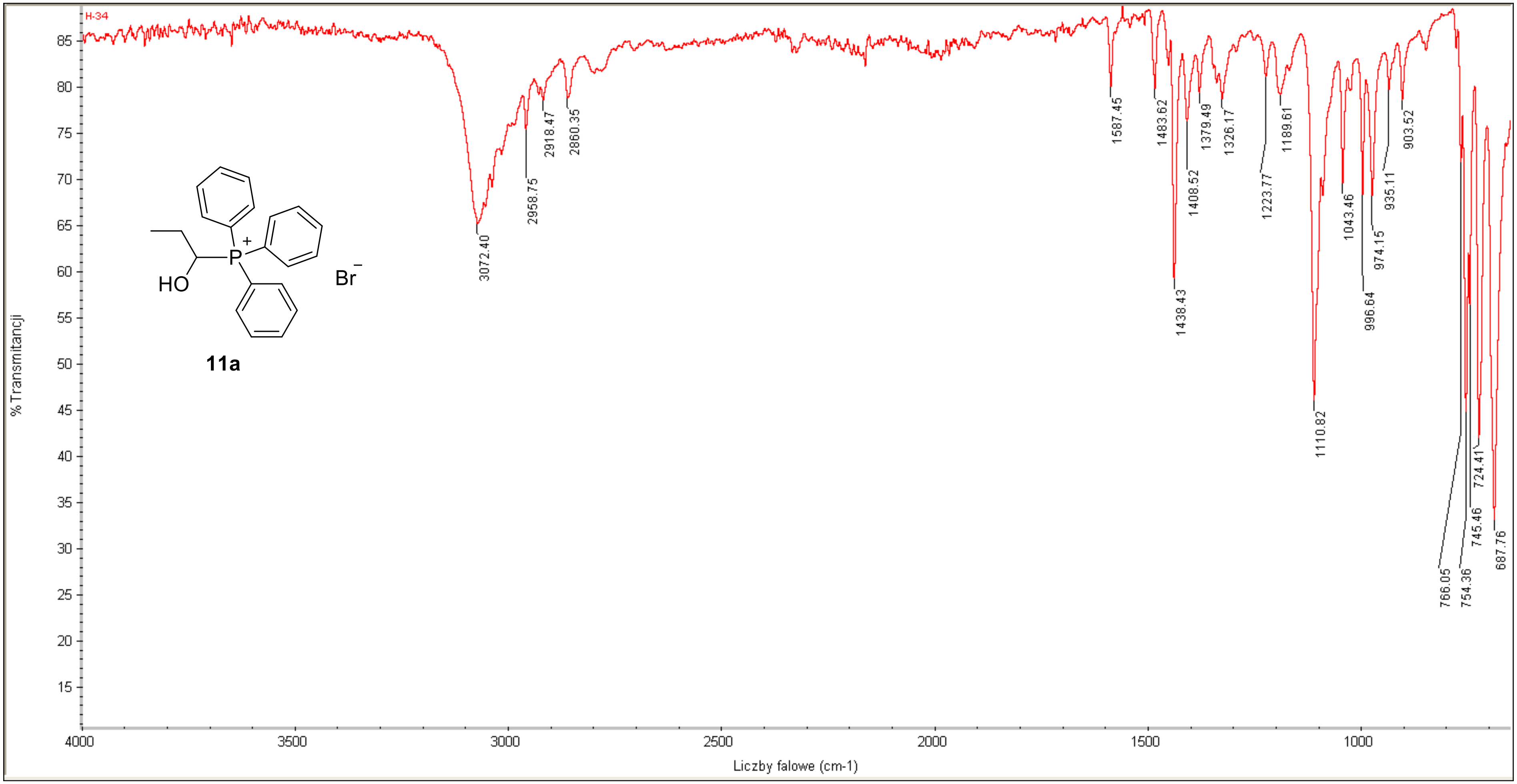

IR spectrum of 1-hydroxypropyltriphenylphosphonium bromide (**11a**); ATR (cm^{-1}).

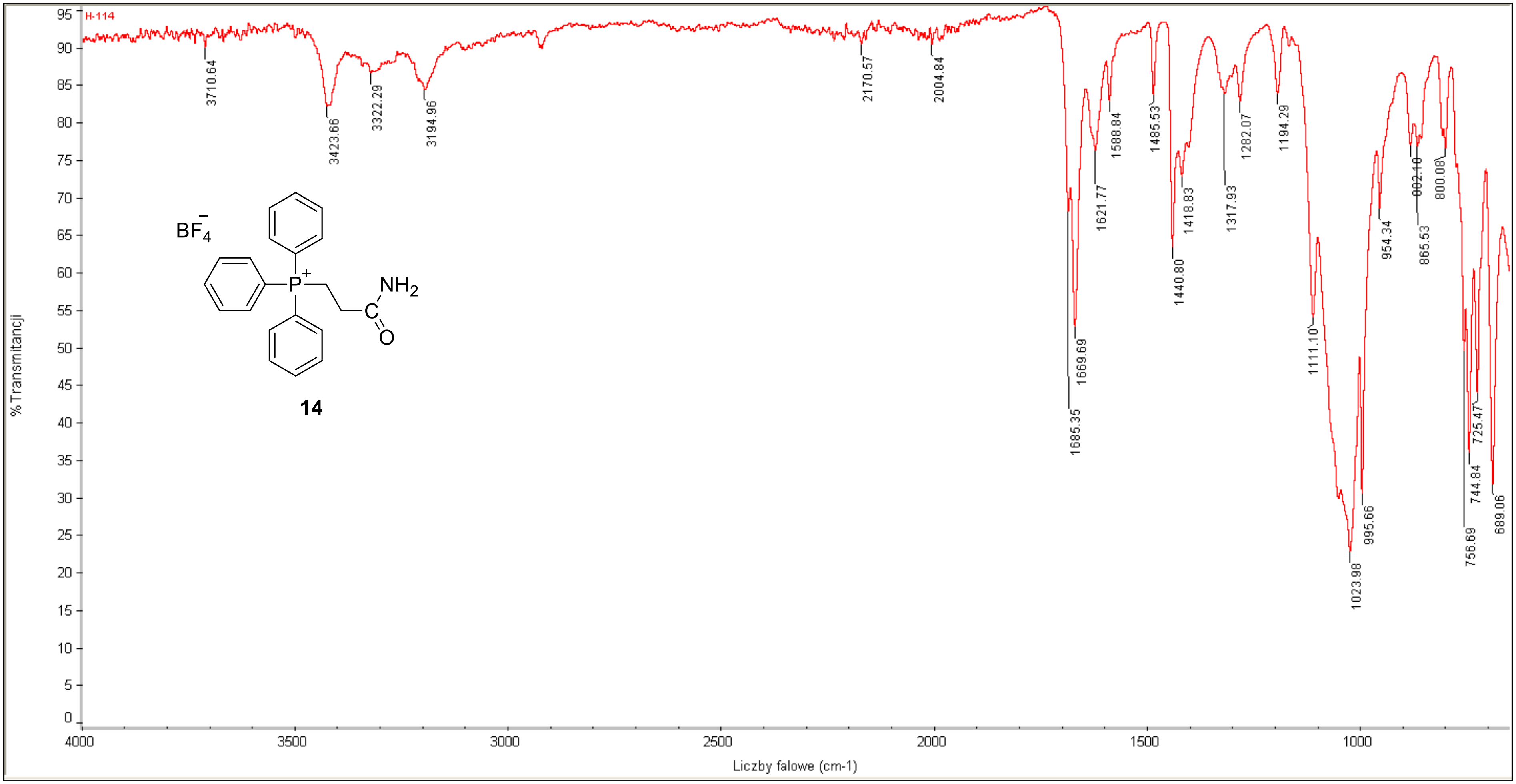

IR spectrum of 2-carbamoyletyhyltriphenylphosphonium tetrafluoroborate (**14**); ATR (cm^{-1}).

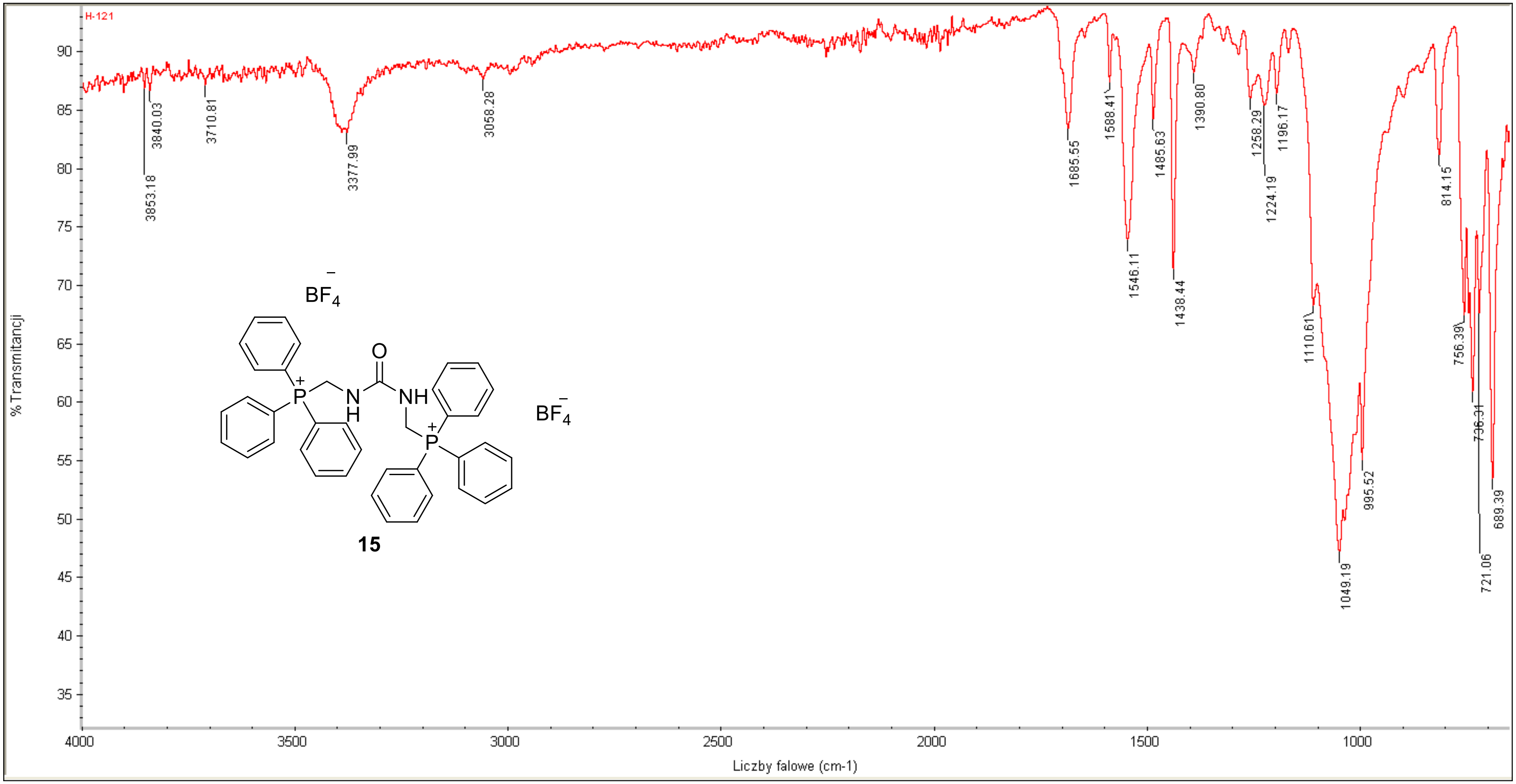

IR spectrum of 1,1'-(carbonyldimino)bis(methyltriphenylphosphonium) bis(tetrafluoroborate) (**15**); ATR (cm^{-1}).

Tolerance = 100.0 mDa / DBE: min = -10.0, max = 50.0
Element prediction: Off
Number of isotope peaks used for i-FIT = 2
Monoisotopic Mass, Even Electron Ions
17 formula(e) evaluated with 7 results within limits (up to 5 closest results for each mass)
Elements Used:

Mass	RA	Calc. Mass	mDa	PPM	DBE	Formula	i-FIT	i-FIT Norm	Fit Conf %	C	H	N	O	P
362.1674	100.00	362.1674	0.0	0.0	12.5	C23 H25 N O P	589.8	5.505	0.41	23	25	1	1	1
		362.1885	-21.1	-58.3	7.5	C20 H29 N O3 P	589.0	4.740	0.87	20	29	1	3	1
		362.1310	36.4	100.5	13.5	C22 H21 N O2 P	587.7	3.407	3.31	22	21	1	2	1
		362.2249	-57.5	-158.8	6.5	C21 H33 N O2 P	584.4	0.153	85.80	21	33	1	2	1
		362.0946	72.8	201.0	14.5	C21 H17 N O3 P	586.6	2.343	9.60	21	17	1	3	1

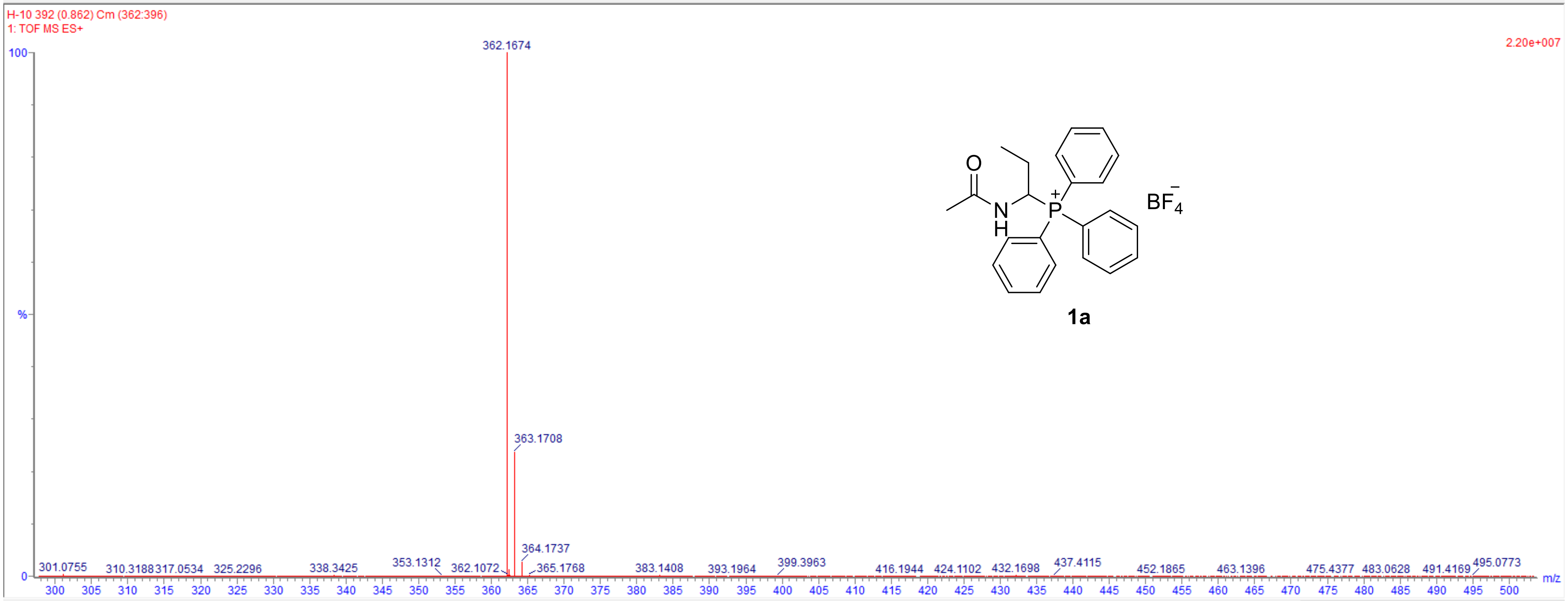

MS spectrum of 1-(*N*-acetylamino)propyltriphenylphosphonium tetrafluoroborate (**1a**).

Tolerance = 100.0 mDa / DBE: min = -10.0, max = 50.0
Element prediction: Off
Number of isotope peaks used for i-FIT = 2
Monoisotopic Mass, Even Electron Ions
6 formula(e) evaluated with 3 results within limits (all results (up to 1000) for each mass)
Elements Used:

Mass	RA	Calc. Mass	mDa	PPM	DBE	Formula	i-FIT	i-FIT Norm	Fit Conf %	C	H	N	O	P
362.1674	100.00	362.1674	0.0	0.0	12.5	C23 H25 N O P	593.4	0.798	45.01	23	25	1	1	1
		362.2613	-93.9	-259.3	5.5	C22 H37 N O P	593.2	0.611	54.27	22	37	1	1	1
		362.0735	93.9	259.3	19.5	C24 H13 N O P	597.5	4.928	0.72	24	13	1	1	1

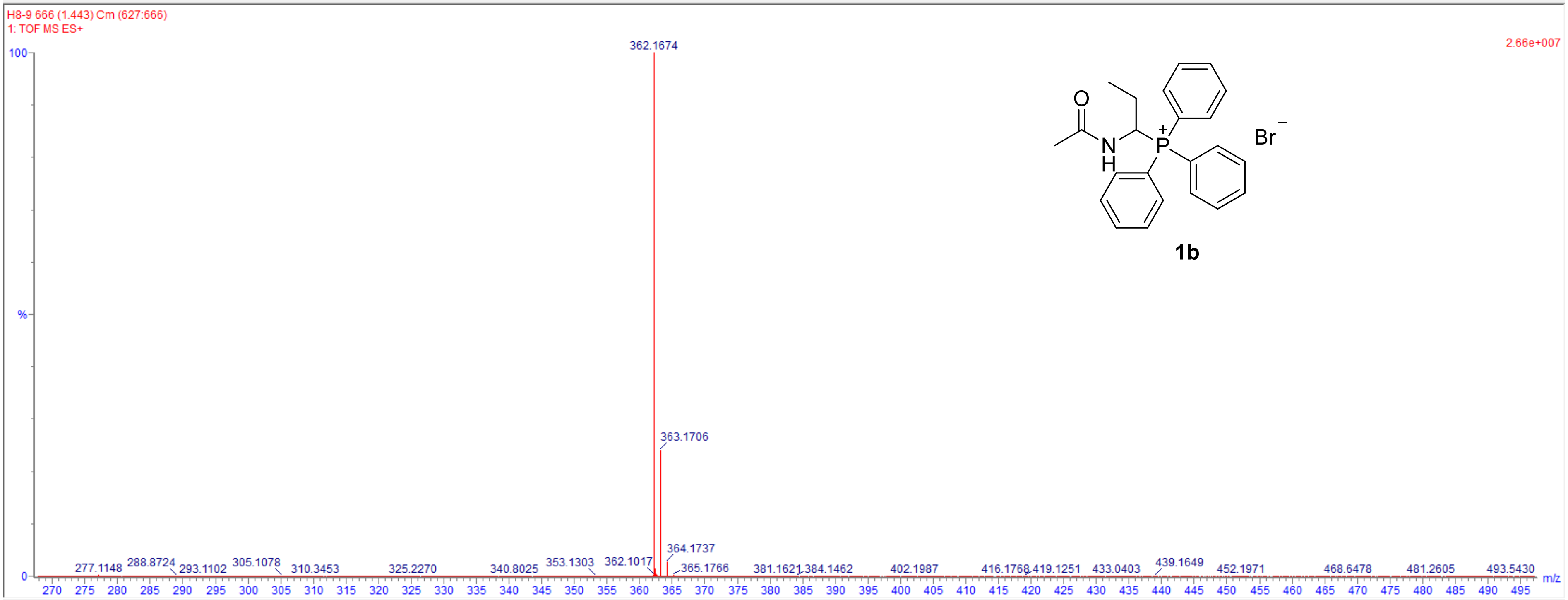

1b

MS spectrum of 1-(*N*-acetylamino)propyltriphenylphosphonium bromide (**1b**).

Multiple Mass Analysis: 2 mass(es) processed
Tolerance = 100.0 mDa / DBE: min = -10.0, max = 50.0
Element prediction: Off
Number of isotope peaks used for i-FIT = 2
Monoisotopic Mass, Even Electron Ions
32 formula(e) evaluated with 13 results within limits (up to 5 closest results for each mass)
Elements Used:

Mass	RA	Calc. Mass	mDa	PPM	DBE	Formula	i-FIT	i-FIT Norm	Fit Conf %	C	H	N	O	P	Cl
464.0504	100.00	464.0505	-0.1	-0.2	12.5	C23 H22 N O P Cl3	349.9	0.120	88.71	23	22	1	1	1	3
		464.0716	-21.2	-45.7	7.5	C20 H26 N O3 P Cl3	353.8	4.020	1.80	20	26	1	3	1	3
		464.0141	36.3	78.2	13.5	C22 H18 N O2 P Cl3	352.4	2.620	7.28	22	18	1	2	1	3
		464.1080	-57.6	-124.1	6.5	C21 H30 N O2 P Cl3	354.0	4.171	1.54	21	30	1	2	1	3
		463.9777	72.7	156.7	14.5	C21 H14 N O3 P Cl3	354.8	5.009	0.67	21	14	1	3	1	3
466.0477	99.29	466.0297	18.0	38.6	12.5	C22 H20 N O2 P Cl3	324.1	5.389	0.46	22	20	1	2	1	3
		466.0661	-18.4	-39.5	11.5	C23 H24 N O P Cl3	318.8	0.005	99.46	23	24	1	1	1	3
		466.0872	-39.5	-84.8	6.5	C20 H28 N O3 P Cl3	328.1	9.370	0.01	20	28	1	3	1	3
		465.9933	54.4	116.7	13.5	C21 H16 N O3 P Cl3	327.5	8.740	0.02	21	16	1	3	1	3
		465.9722	75.5	162.0	18.5	C24 H12 N O P Cl3	326.2	7.480	0.06	24	12	1	1	1	3

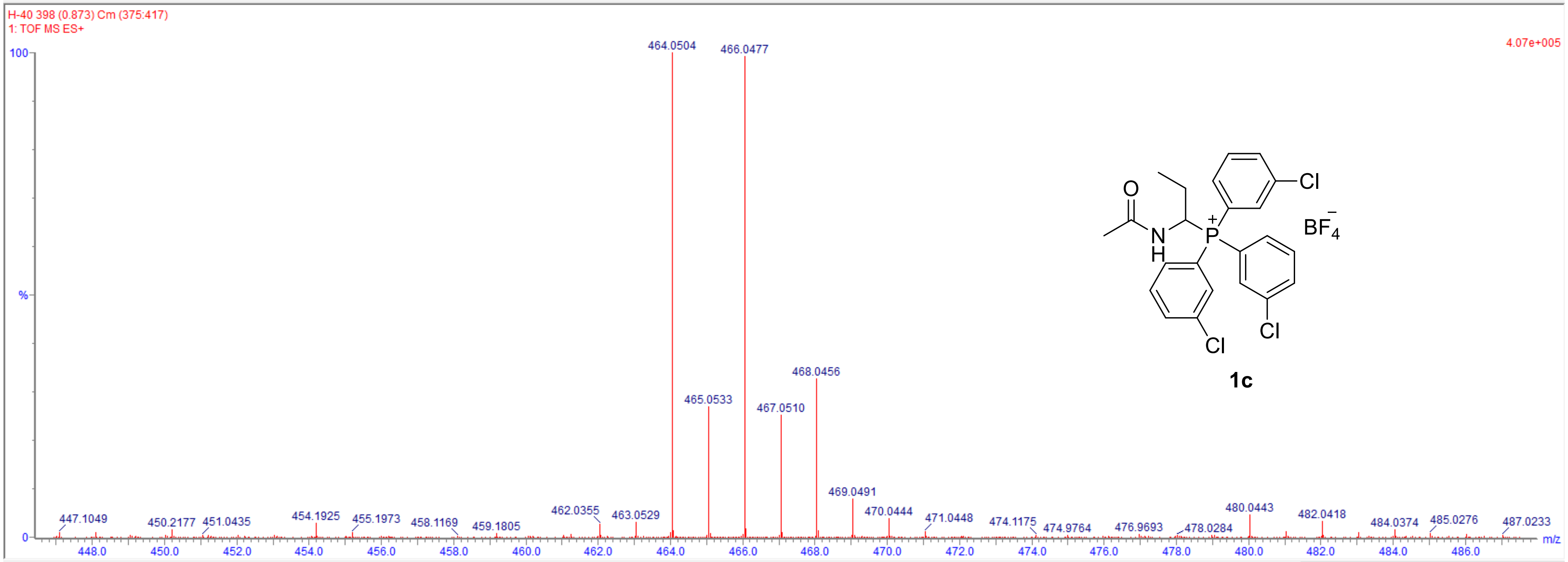

For Help, press F1

MS spectrum of 1-(*N*-acetylamino)propyltris(3-chlorophenyl)phosphonium tetrafluoroborate (**1c**).

Multiple Mass Analysis: 2 mass(es) processed
Tolerance = 50.0 mDa / DBE: min = -10.0, max = 50.0
Element prediction: Off
Number of isotope peaks used for i-FIT = 2
Monoisotopic Mass, Even Electron Ions
84 formula(e) evaluated with 10 results within limits (up to 3 closest results for each mass)
Elements Used:

Mass	RA	Calc. Mass	mDa	PPM	DBE	Formula	i-FIT	i-FIT Norm	Fit Conf %	C	H	N	O	P
530.2094	100.00	530.2096	-0.2	-0.4	16.5	C31 ...	3...	0.135	87.39	31	33	1	5	1
		530.2120	-2.6	-4.9	25.5	C38 ...	3...	3.478	3.09	38	28	1	2	
		530.1967	12.7	24.0	21.5	C34 ...	3...	2.351	9.52	34	28	1	5	
531.2125	31.08	---												

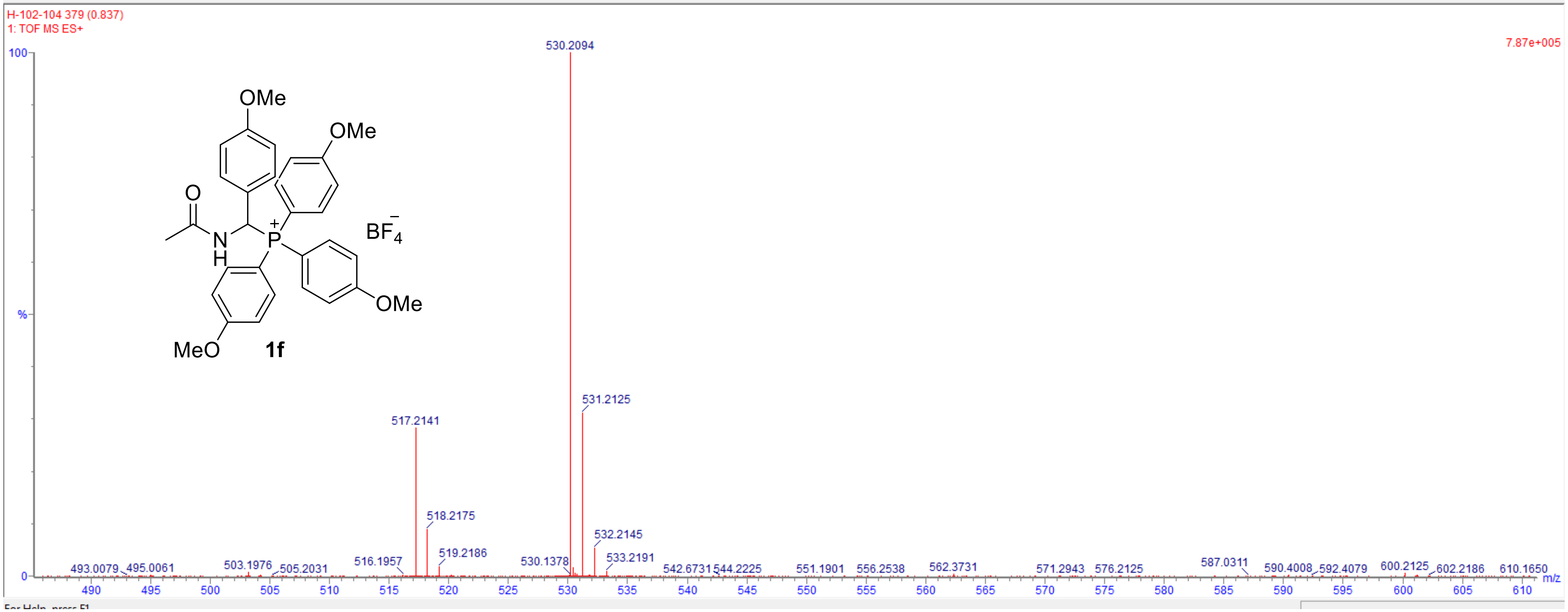

For Help, press F1

MS spectrum of 1-(*N*-acetylamino)-1-(4-metoxyphenyl)methyltris(4-metoxyphenyl)phosphonium tetrafluoroborate (**1f**).

Multiple Mass Analysis: 2 mass(es) processed
Tolerance = 50.0 mDa / DBE: min = -10.0, max = 50.0
Element prediction: Off
Number of isotope peaks used for i-FIT = 2
Monoisotopic Mass, Even Electron Ions
4939 formula(e) evaluated with 403 results within limits (up to 3 closest results for each mass)
Elements Used:

Mass	RA	Calc. Mass	mDa	PPM	DBE	Formula	i-FIT	i-FIT Norm	Fit Conf %	C	H	N	O	Na	P	S	Cl
436.0193	100.00	436.0192	0.1	0.2	12.5	C21 H18 N O P Cl3	245.1	0.028	97.21	21	18	1	1		1		3
		436.0197	-0.4	-0.9	27.5	C30 H7 N Na S	250.4	5.300	0.50	30	7	1		1		1	
		436.0197	-0.4	-0.9	21.5	C25 H11 N O3 P S	248.9	3.777	2.29	25	11	1	3		1	1	
438.0170	97.67	438.0171	-0.1	-0.2	4.5	C15 H21 N O4 Na P Cl3	312.6	0.324	72.29	15	21	1	4	1	1		3
		438.0169	0.1	0.2	21.5	C25 H9 N O5 Cl	313.8	1.512	22.04	25	9	1	5				1
		438.0167	0.3	0.7	27.5	C29 H5 N O3 Na	315.2	2.870	5.67	29	5	1	3	1			

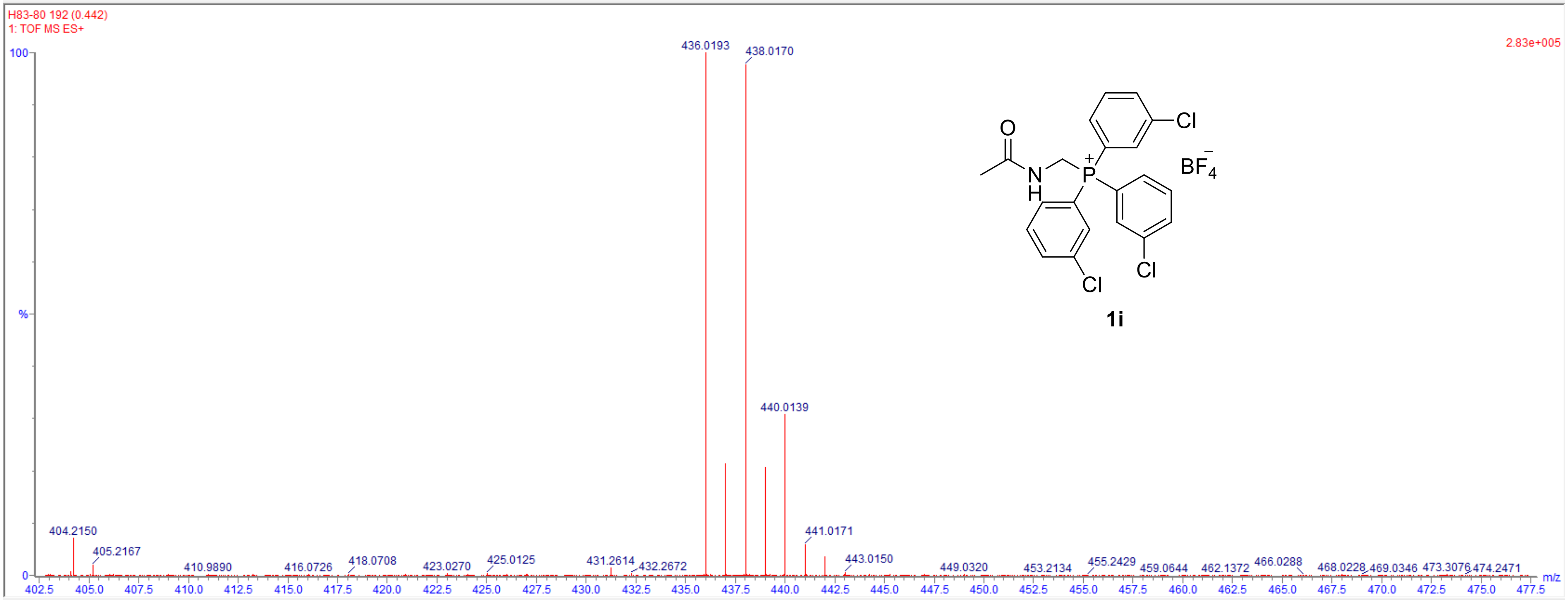

For Help, press F1

MS spectrum of (*N*-acetylamino)methyltris(3-chlorophenyl)phosphonium tetrafluoroborate (**1i**).

Tolerance = 100.0 mDa / DBE: min = -10.0, max = 50.0
Element prediction: Off
Number of isotope peaks used for i-FIT = 2
Monoisotopic Mass, Even Electron Ions
18 formula(e) evaluated with 7 results within limits (up to 5 closest results for each mass)
Elements Used:

Mass	RA	Calc. Mass	mDa	PPM	DBE	Formula	i-FIT	i-FIT Norm	Fit Conf %	C	H	N	O	P
424.1831	100.00	424.1830	0.1	0.2	16.5	C28 H27 N O P	533.1	5.505	0.41	28	27	1	1	1
		424.2042	-21.1	-49.7	11.5	C25 H31 N O3 P	527.8	0.233	79.19	25	31	1	3	1
		424.1466	36.5	86.0	17.5	C27 H23 N O2 P	532.2	4.580	1.03	27	23	1	2	1
		424.2405	-57.4	-135.3	10.5	C26 H35 N O2 P	529.9	2.323	9.80	26	35	1	2	1
		424.1103	72.8	171.6	18.5	C26 H19 N O3 P	529.9	2.345	9.59	26	19	1	3	1

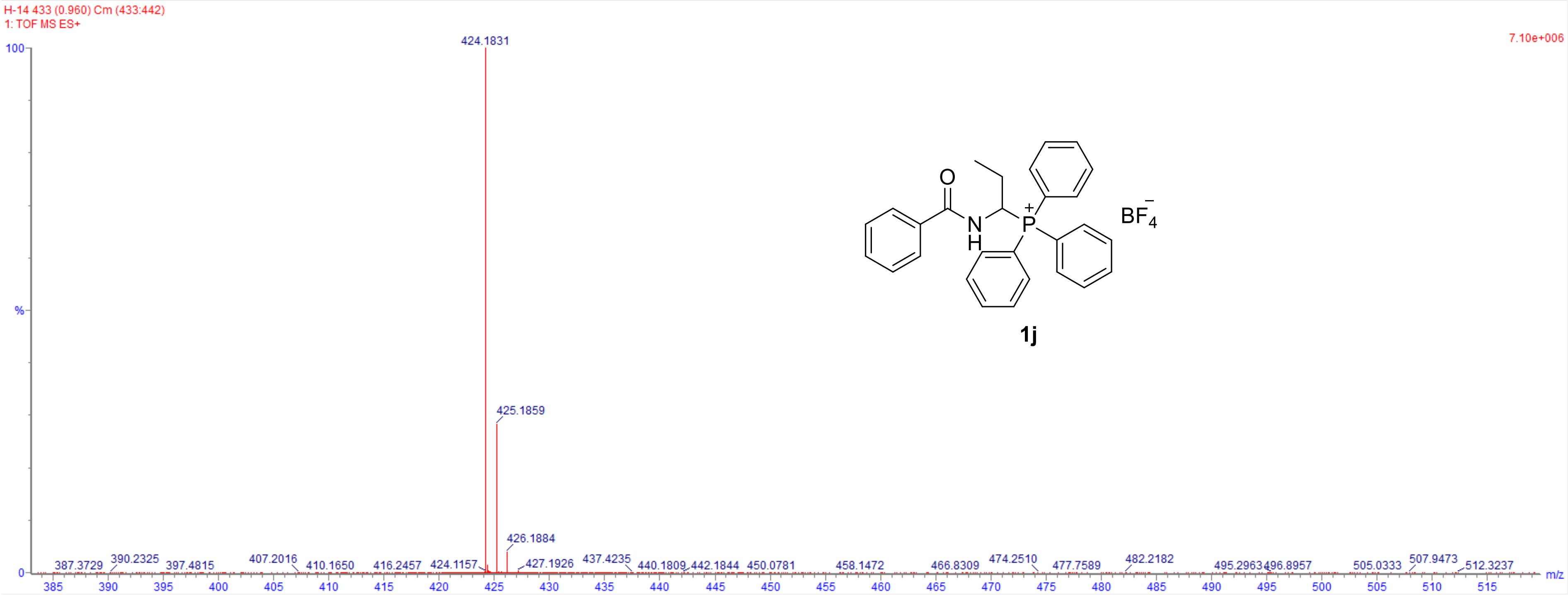

For Help, press F1

MS spectrum of 1-(*N*-benzoylamino)propyltriphenylphosphonium tetrafluoroborate (**1j**).

Multiple Mass Analysis: 2 mass(es) processed
Tolerance = 50.0 mDa / DBE: min = -10.0, max = 50.0
Element prediction: Off
Number of isotope peaks used for i-FIT = 2
Monoisotopic Mass, Even Electron Ions
1504 formula(e) evaluated with 185 results within limits (up to 3 closest results for each mass)
Elements Used:

Mass	RA	Calc. Mass	mDa	PPM	DBE	Formula	i-FIT	i-FIT Norm	Fit Conf %	C	H	N	O	P	Cl
512.0502	100.00	512.0505	-0.3	-0.6	16.5	C27 H22 N O P Cl3	113.8	0.405	66.69	27	22	1	1	1	3
		512.0488	1.4	2.7	21.5	C29 H17 N3 Cl3	115.4	2.016	13.32	29	17	3			3
		512.0486	1.6	3.1	21.5	C28 H17 N3 O P Cl2	115.0	1.610	19.99	28	17	3	1	1	2
514.0485	96.27	514.0490	-0.5	-1.0	16.5	C24 H19 N3 O4 P Cl2	100.8	5.100	0.61	24	19	3	4	1	2
		514.0492	-0.7	-1.4	16.5	C25 H19 N3 O3 Cl3	99.6	3.851	2.13	25	19	3	3		3
		514.0474	1.1	2.1	21.5	C26 H14 N5 O3 Cl2	95.8	0.028	97.26	26	14	5	3		2

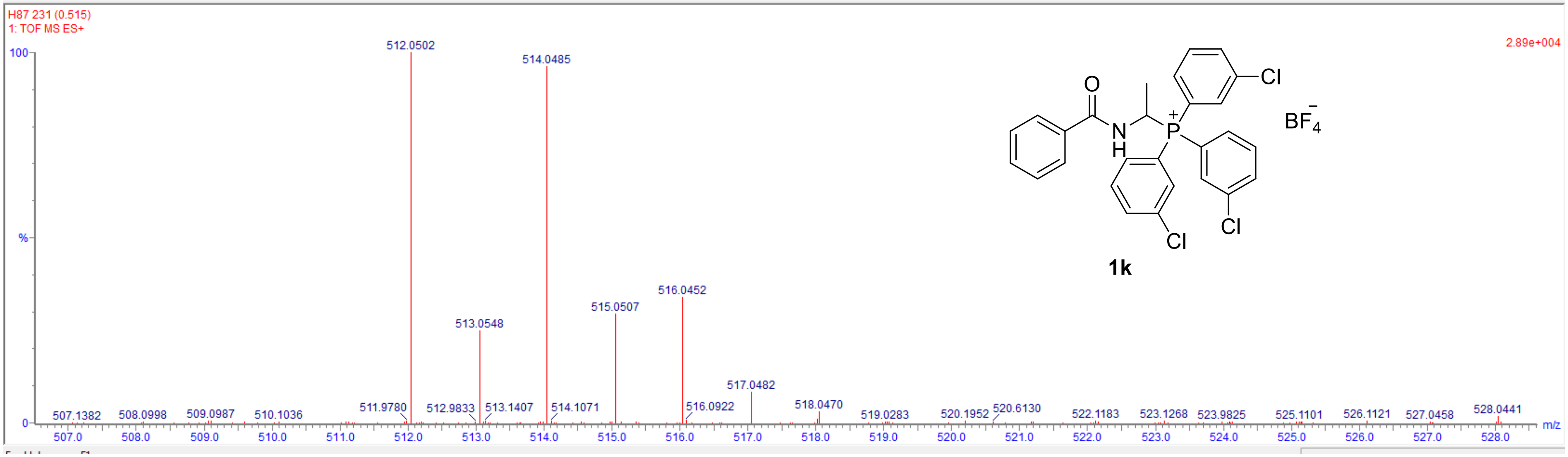

MS spectrum of 1-(*N*-benzoylamino)ethyltris(3-chlorophenyl)phosphonium tetrafluoroborate (**1k**).

Multiple Mass Analysis: 2 mass(es) processed
Tolerance = 100.0 mDa / DBE: min = -10.0, max = 50.0
Element prediction: Off
Number of isotope peaks used for i-FIT = 2
Monoisotopic Mass, Even Electron Ions
56 formula(e) evaluated with 22 results within limits (up to 5 closest results for each mass)
Elements Used:

Mass	RA	Calc. Mass	mDa	PPM	DBE	Formula	i-FIT	i-FIT Norm	Fit Conf %	C	H	N	O	P	Cl
498.0348	100.00	498.0348	0.0	0.0	16.5	C26 H20 N O P Cl3	474.3	4.987	0.68	26	20	1	1	1	3
		498.0196	15.2	30.5	12.5	C22 H20 N O4 P Cl3	472.9	3.569	2.82	22	20	1	4	1	3
		498.0559	-21.1	-42.4	11.5	C23 H24 N O3 P Cl3	469.3	0.056	94.54	23	24	1	3	1	3
		497.9984	36.4	73.1	17.5	C25 H16 N O2 P Cl3	473.3	3.990	1.85	25	16	1	2	1	3
		498.0771	-42.3	-84.9	6.5	C20 H28 N O5 P Cl3	476.1	6.833	0.11	20	28	1	5	1	3
500.0323	98.60	500.0352	-2.9	-5.8	11.5	C22 H22 N O4 P Cl3	495.6	4.018	1.80	22	22	1	4	1	3
		500.0505	-18.2	-36.4	15.5	C26 H22 N O P Cl3	497.8	6.218	0.20	26	22	1	1	1	3
		500.0141	18.2	36.4	16.5	C25 H18 N O2 P Cl3	496.3	4.785	0.84	25	18	1	2	1	3

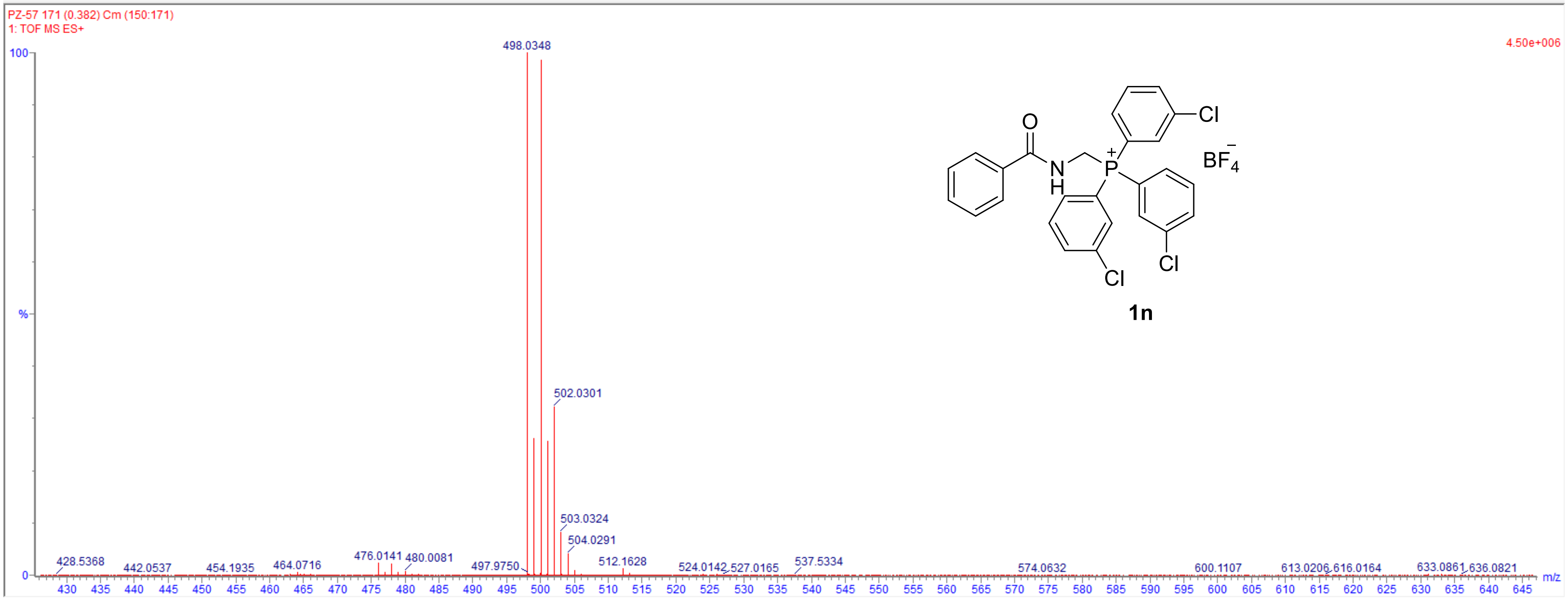

MS spectrum of (*N*-benzoylamino)methyltris(3-chlorophenyl)phosphonium tetrafluoroborate (**1n**).

Tolerance = 50.0 mDa / DBE: min = -10.0, max = 50.0
Element prediction: Off
Number of isotope peaks used for i-FIT = 2
Monoisotopic Mass, Even Electron Ions
2470 formula(e) evaluated with 179 results within limits (up to 3 closest results for each mass)
Elements Used:

Mass	RA	Calc. Mass	mDa	PPM	DBE	Formula	i-FIT	i-FIT Norm	Fit Conf %	C	H	N	O	Na	P	S	Cl
404.2144	100.00	404.2143	0.1	0.2	12.5	C26 H31 N O P	338.9	1.300	27.27	26	31	1	1		1		
		404.2145	-0.1	-0.2	12.5	C27 H31 N Cl	339.8	2.271	10.32	27	31	1					1
		404.2153	-0.9	-2.2	4.5	C21 H36 N O Na P S	338.0	0.471	62.41	21	36	1	1	1	1	1	

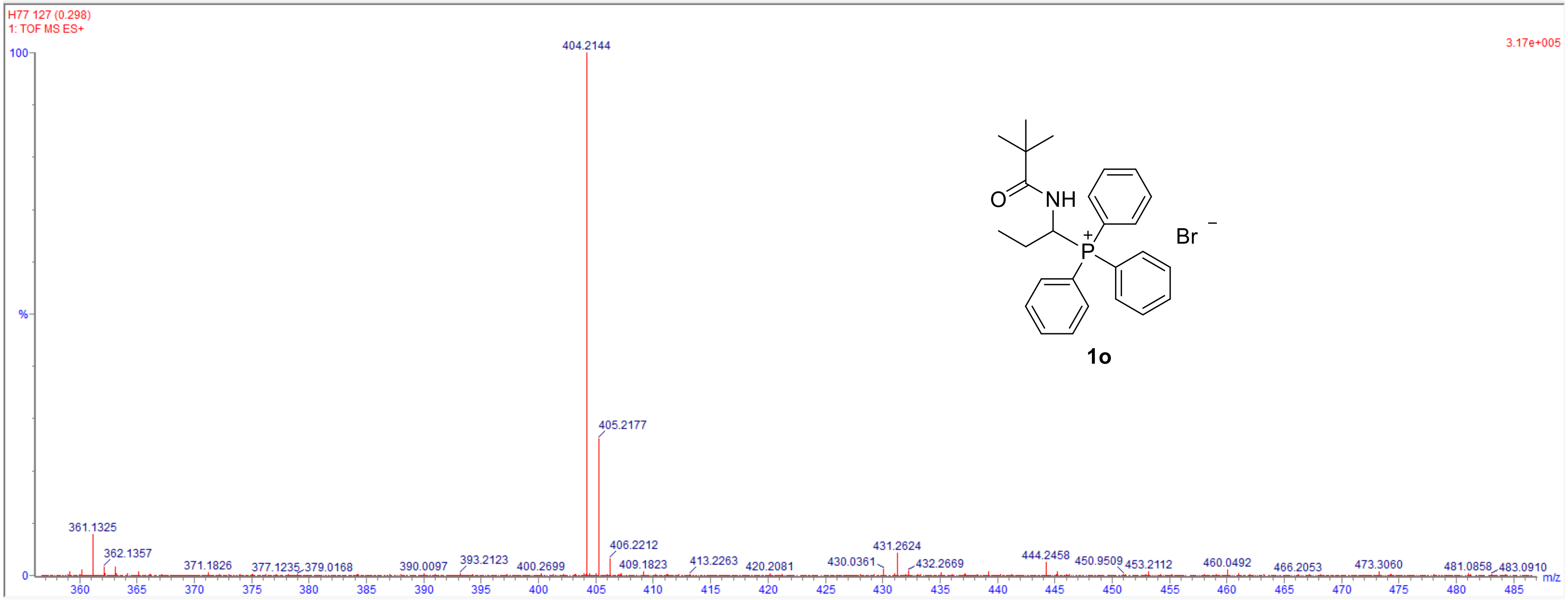

MS spectrum of 1-(*N*-pivaloylamino)propyltriphenylphosphonium bromide (**1o**).

Tolerance = 50.0 mDa / DBE: min = -10.0, max = 50.0
Element prediction: Off
Number of isotope peaks used for i-FIT = 2
Monoisotopic Mass, Even Electron Ions
2420 formula(e) evaluated with 199 results within limits (up to 3 closest results for each mass)
Elements Used:

Mass	RA	Calc. Mass	mDa	PPM	DBE	Formula	i-FIT	i-FIT Norm	Fit Conf %	C	H	N	O	Na	P	S	Cl
458.1707	100.00	458.1707	0.0	0.0	15.5	C28 H29 N O P S	468.7	1.034	35.55	28	29	1	1		1	1	
		458.1709	-0.2	-0.4	15.5	C29 H29 N S Cl	468.2	0.506	60.28	29	29	1				1	1
		458.1710	-0.3	-0.7	8.5	C23 H30 N O5 Na Cl	470.9	3.177	4.17	23	30	1	5	1			1

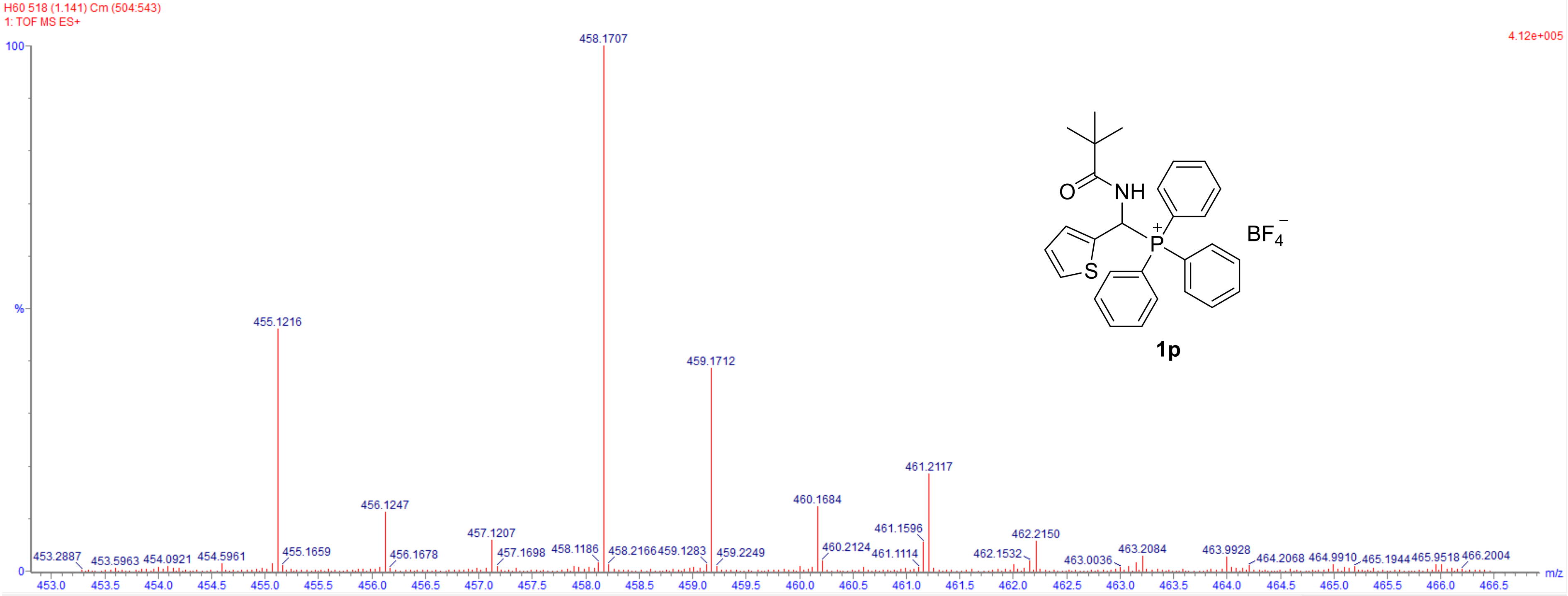

MS spectrum of 1-(*N*-pivaloylamino)-1-(2-thienyl)methyltriphenylphosphonium tetrafluoroborate (**1p**).

Tolerance = 50.0 mDa / DBE: min = -10.0, max = 50.0
Element prediction: Off
Number of isotope peaks used for i-FIT = 2
Monoisotopic Mass, Even Electron Ions
2387 formula(e) evaluated with 179 results within limits (up to 3 closest results for each mass)
Elements Used:

Mass	RA	Calc. Mass	mDa	PPM	DBE	Formula	i-FIT	i-FIT Norm	Fit Conf %	C	H	N	O	Na	P	S	Cl
348.1520	100.00	348.1517	0.3	0.9	12.5	C22 H23 N O P	344.9	0.237	78.88	22	23	1	1		1		
		348.1519	0.1	0.3	12.5	C23 H23 N Cl	346.5	1.879	15.27	23	23	1					1
		348.1527	-0.7	-2.0	4.5	C17 H28 N O Na P S	347.5	2.839	5.85	17	28	1	1	1	1	1	

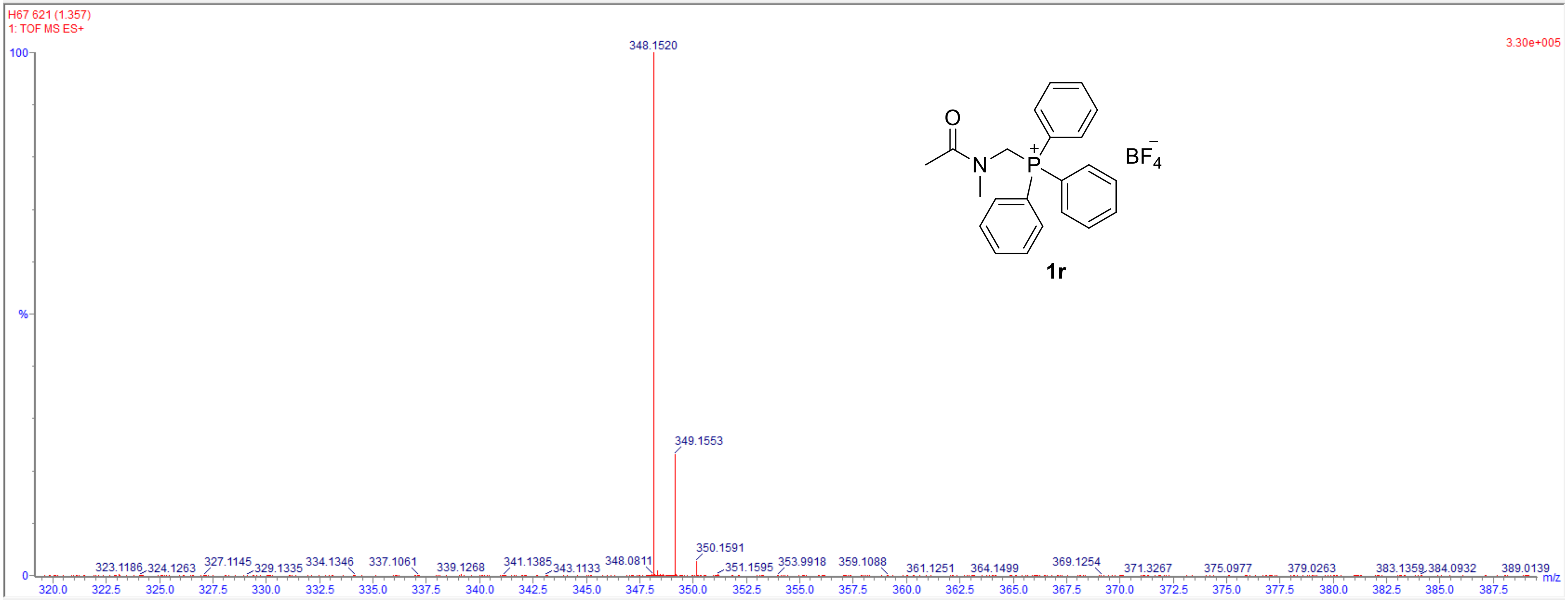

MS spectrum of *N*-(*N*-methylacetylamino)methyltriphenylphosphonium tetrafluoroborate (**1r**).

Tolerance = 100.0 mDa / DBE: min = -10.0, max = 50.0
Element prediction: Off
Number of isotope peaks used for i-FIT = 2
Monoisotopic Mass, Even Electron Ions
18 formula(e) evaluated with 7 results within limits (up to 5 closest results for each mass)
Elements Used:

Mass	RA	Calc. Mass	mDa	PPM	DBE	Formula	i-FIT	i-FIT Norm	Fit Conf %	C	H	N	O	P
454.1938	100.00	454.1936	0.2	0.4	16.5	C29 H29 N O2 P	519.2	3.253	3.87	29	29	1	2	1
		454.2300	-36.2	-79.7	15.5	C30 H33 N O P	520.9	4.881	0.76	30	33	1	1	1
		454.1572	36.6	80.6	17.5	C28 H25 N O3 P	518.2	2.172	11.39	28	25	1	3	1
		454.2511	-57.3	-126.2	10.5	C27 H37 N O3 P	516.2	0.177	83.77	27	37	1	3	1
		454.1361	57.7	127.0	22.5	C31 H21 N O P	522.1	6.157	0.21	31	21	1	1	1

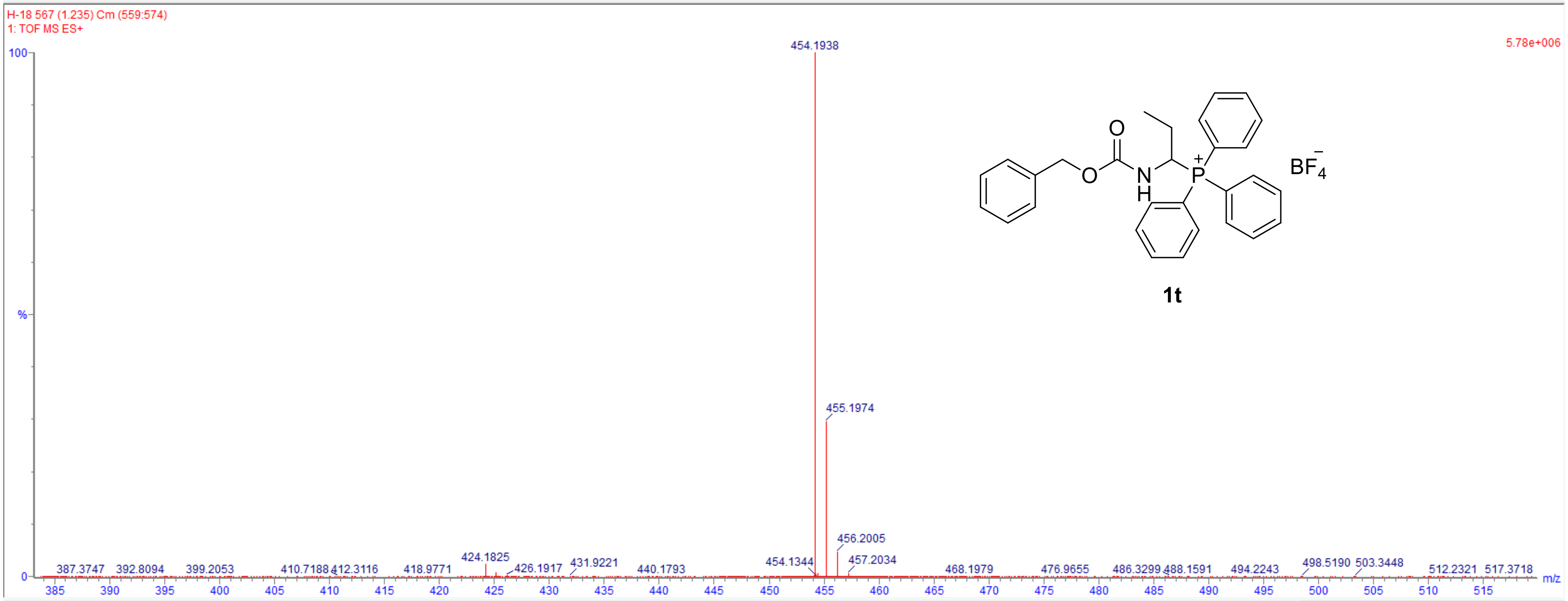

MS spectrum of 1-(*N*-benzyloxycarbonylamino)propyltriphenylphosphonium tetrafluoroborate (**1t**).

Multiple Mass Analysis: 2 mass(es) processed
Tolerance = 50.0 mDa / DBE: min = -10.0, max = 50.0
Element prediction: Off
Number of isotope peaks used for i-FIT = 2
Monoisotopic Mass, Even Electron Ions
26 formula(e) evaluated with 3 results within limits (up to 3 closest results for each mass)
Elements Used:

Mass	RA	Calc. Mass	mDa	PPM	DBE	Formula	i-FIT	i-FIT Norm	Fit Conf %	C	H	N	O	P
532.2043	100.00	532.2042	0.1	0.2	20.5	C34 ...	2...	4.369	1.27	34	31	1	3	1
		532.1830	21.3	40.0	25.5	C37 ...	2...	0.032	96.89	37	27	1	1	1
		532.2405	-36.2	-68.0	19.5	C35 ...	2...	3.996	1.84	35	35	1	2	1
533.2057	41.23	---												

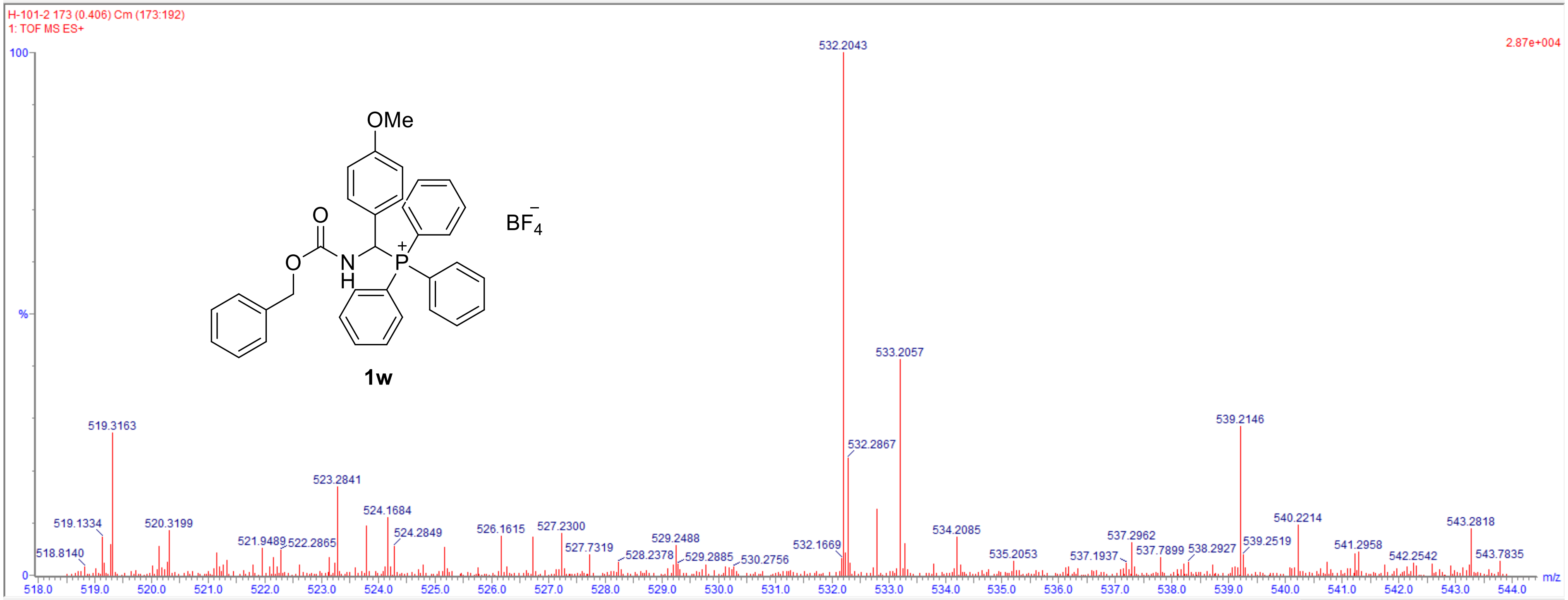

MS spectrum of 1-(*N*-benzyloxycarbonylamino)-1-(4-methoxyphenyl)methyltriphenylphosphonium tetrafluoroborate (**1w**).

Multiple Mass Analysis: 2 mass(es) processed
Tolerance = 20.0 mDa / DBE: min = -10.0, max = 50.0
Element prediction: Off
Number of isotope peaks used for i-FIT = 2
Monoisotopic Mass, Even Electron Ions
68 formula(e) evaluated with 4 results within limits (up to 3 closest results for each mass)
Elements Used:

Mass	RA	Calc. Mass	mDa	PPM	DBE	Formula	i-FIT	i-FIT Norm	Fit Conf %	C	H	N	O	P
547.1784	100.00	547.1787	-0.3	-0.5	21.5	C33 H28 N2 O4 P	329.7	0.006	99.40	33	28	2	4	1
		547.1939	-15.5	-28.3	25.5	C37 H28 N2 O P	334.8	5.114	0.60	37	28	2	1	1
548.1815	34.60	548.1779	3.6	6.6	25.5	C37 H27 N O2 P	258.4	0.161	85.13	37	27	1	2	1
		548.1991	-17.6	-32.1	20.5	C34 H31 N O4 P	260.2	1.906	14.87	34	31	1	4	1

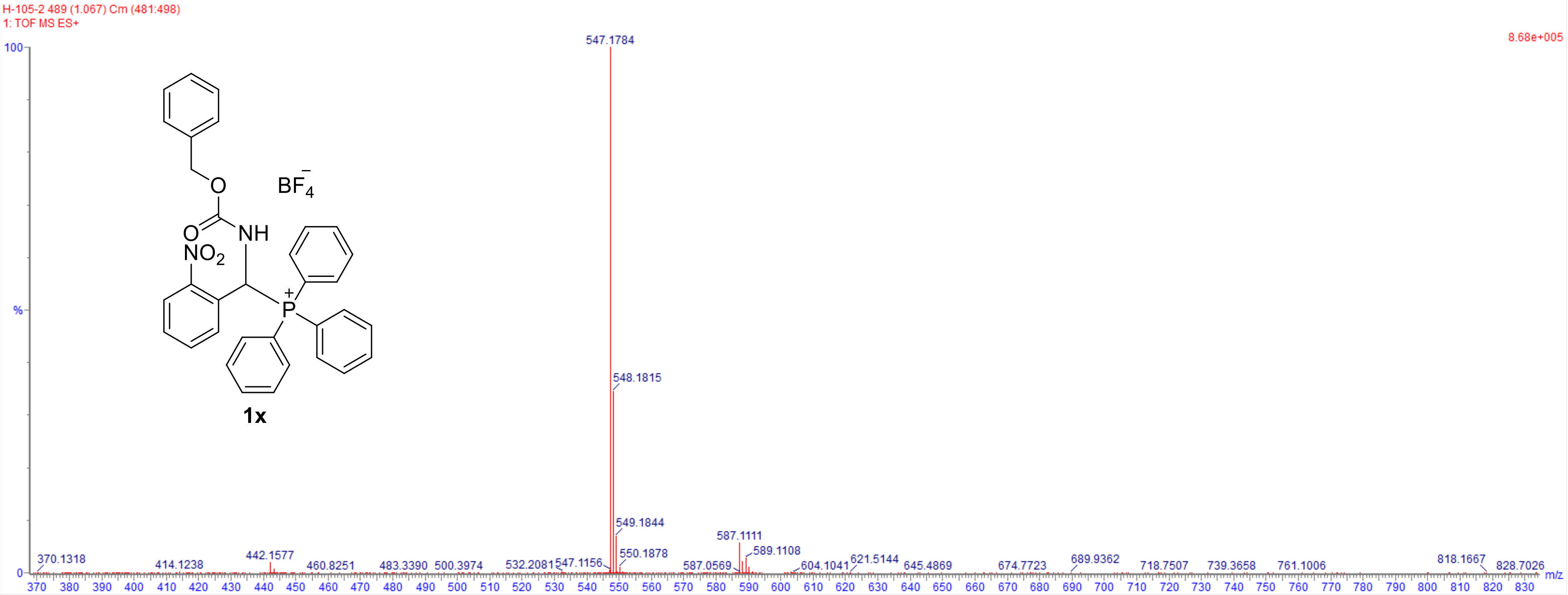

MS spectrum of 1-(*N*-benzyloxycarbonylamino)-1-(2-nitrophenyl)methyltriphenylphosphonium tetrafluoroborate (**1x**).

Multiple Mass Analysis: 3 mass(es) processed
Tolerance = 50.0 mDa / DBE: min = -10.0, max = 50.0
Element prediction: Off
Number of isotope peaks used for i-FIT = 2
Monoisotopic Mass, Even Electron Ions
36 formula(e) evaluated with 6 results within limits (up to 3 closest results for each mass)
Elements Used:

Mass	RA	Calc. Mass	mDa	PPM	DBE	Formula	i-FIT	i-FIT Norm	Fit Conf %	C	H	N	O	P
552.2095	56.96	552.2092	0.3	0.5	23.5	C37 H31 N O2 P	421.4	1.262	28.30	37	31	1	2	1
		552.2456	-36.1	-65.4	22.5	C38 H35 N O P	425.9	5.725	0.33	38	35	1	1	1
		552.1729	36.6	66.3	24.5	C36 H27 N O3 P	420.5	0.337	71.37	36	27	1	3	1
557.1802	100.00	---												
558.1837	38.45	558.1987	-15.0	-26.9	26.5	C39 H29 N O P	423.9	0.745	47.48	39	29	1	1	1
		558.1623	21.4	38.3	27.5	C38 H25 N O2 P	424.2	1.004	36.64	38	25	1	2	1
		558.2198	-36.1	-64.7	21.5	C36 H33 N O3 P	425.0	1.841	15.87	36	33	1	3	1

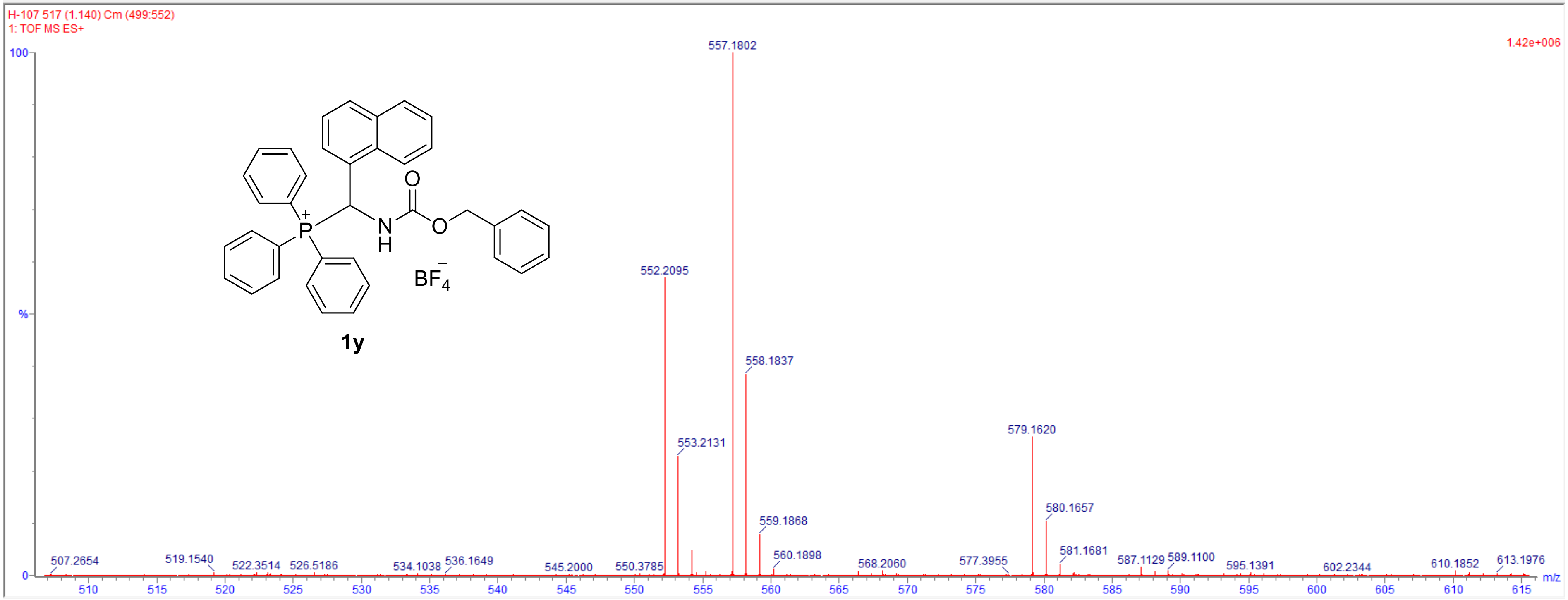

MS spectrum of 1-(*N*-benzyloxycarbonylamino)-1-(1-naphthyl)methyltriphenylphosphonium tetrafluoroborate (**1y**).

Tolerance = 50.0 mDa / DBE: min = -10.0, max = 50.0
Element prediction: Off
Number of isotope peaks used for i-FIT = 2
Monoisotopic Mass, Even Electron Ions
691 formula(e) evaluated with 65 results within limits (up to 3 closest results for each mass)
Elements Used:

Mass	RA	Calc. Mass	mDa	PPM	DBE	Formula	i-FIT	i-FIT Norm	Fit Conf %	C	H	N	O	Na	P	Cl
544.2253	100.00	544.2253	0.0	0.0	16.5	C32 H35 N O5 P	305.4	1.358	25.71	32	35	1	5		1	
		544.2257	-0.4	-0.7	-0.5	C23 H47 N O3 Na P Cl3	306.9	2.876	5.64	23	47	1	3	1	1	3
		544.2231	2.2	4.0	13.5	C31 H36 N O4 Na Cl	304.4	0.376	68.65	31	36	1	4	1		1

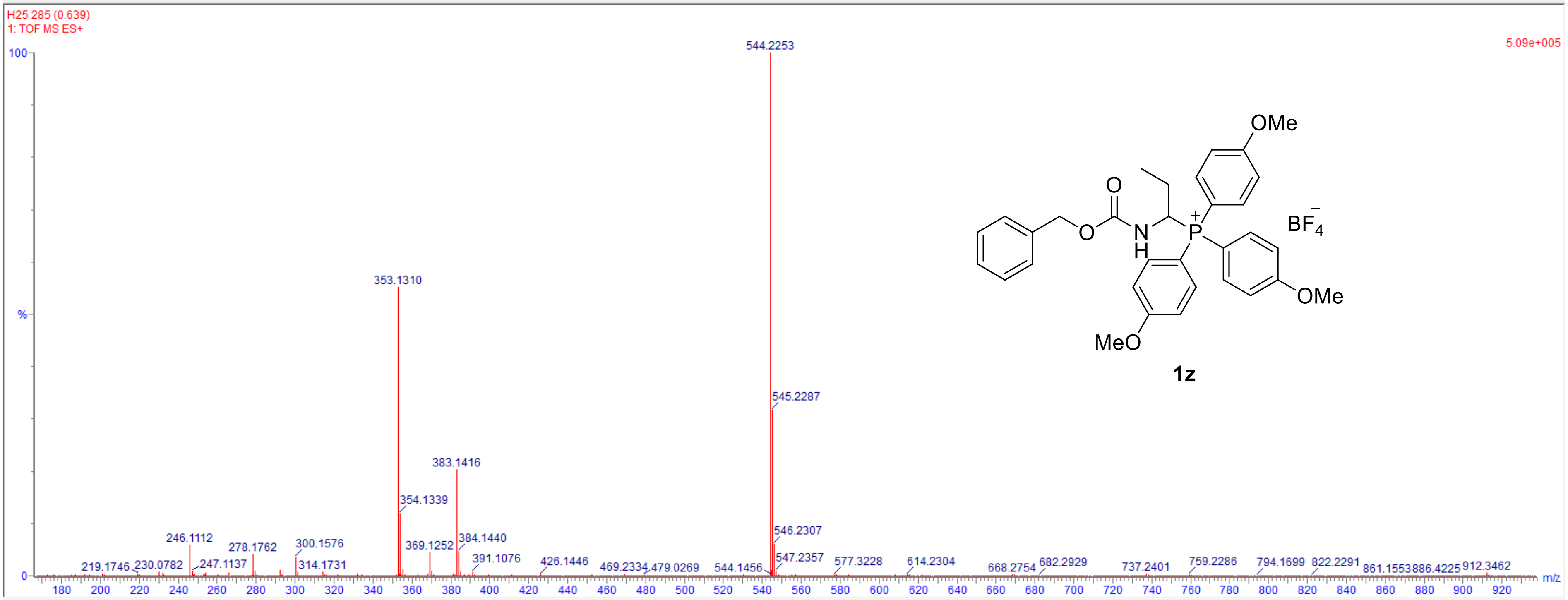

MS spectrum of 1-(*N*-benzyloxycarbonylamino)propyltris(4-methoxyphenyl)phosphonium tetrafluoroborate (**1z**).

Multiple Mass Analysis: 3 mass(es) processed
Tolerance = 50.0 mDa / DBE: min = -10.0, max = 50.0
Element prediction: Off
Number of isotope peaks used for i-FIT = 2
Monoisotopic Mass, Even Electron Ions
39 formula(e) evaluated with 3 results within limits (up to 3 closest results for each mass)
Elements Used:

Mass	RA	Calc. Mass	mDa	PPM	DBE	Formula	i-FIT	i-FIT Norm	Fit Conf %	C	H	N	O	P
458.1884	100.00	458.1885	-0.1	-0.2	15.5	C28 ...	3...	0.001	99.95	28	29	1	3	1
		458.1674	21.0	45.8	20.5	C31 ...	3...	9.685	0.01	31	25	1	1	1
		458.2249	-36.5	-79.7	14.5	C29 ...	3...	7.713	0.04	29	33	1	2	1
459.1914	31.06	---												
477.2216	31.21	---												

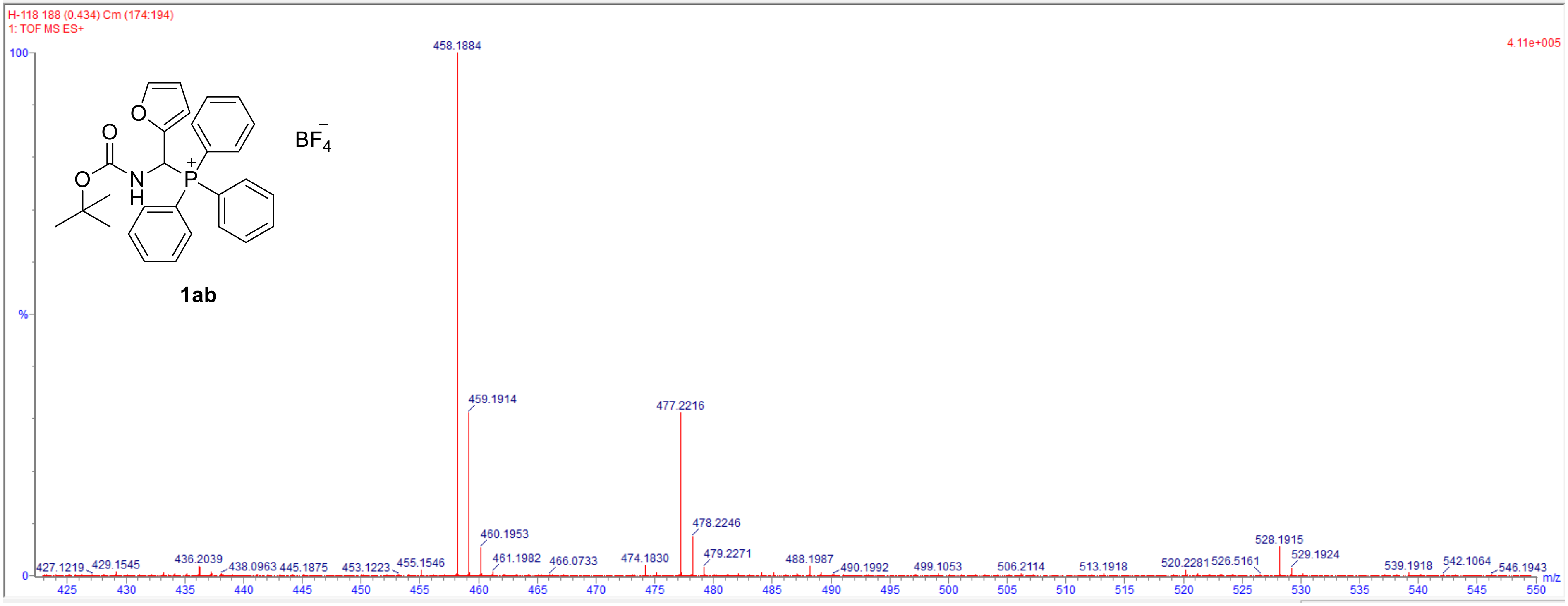

MS spectrum of 1-(*N*-tert-butoxycarbonylamino)-1-(2-furyl)methyltriphenylphosphonium tetrafluoroborate (**1ab**).

Tolerance = 50.0 mDa / DBE: min = -10.0, max = 50.0
Element prediction: Off
Number of isotope peaks used for i-FIT = 2
Monoisotopic Mass, Even Electron Ions
2454 formula(e) evaluated with 185 results within limits (up to 3 closest results for each mass)
Elements Used:

Mass	RA	Calc. Mass	mDa	PPM	DBE	Formula	i-FIT	i-FIT Norm	Fit Conf %	C	H	N	O	Na	P	S	Cl
388.1831	100.00	388.1830	0.1	0.3	13.5	C25 H27 N O P	113.1	0.140	86.91	25	27	1	1		1		
		388.1832	-0.1	-0.3	13.5	C26 H27 N Cl	115.0	2.061	12.73	26	27	1					1
		388.1840	-0.9	-2.3	5.5	C20 H32 N O Na P S	118.6	5.610	0.37	20	32	1	1	1	1	1	

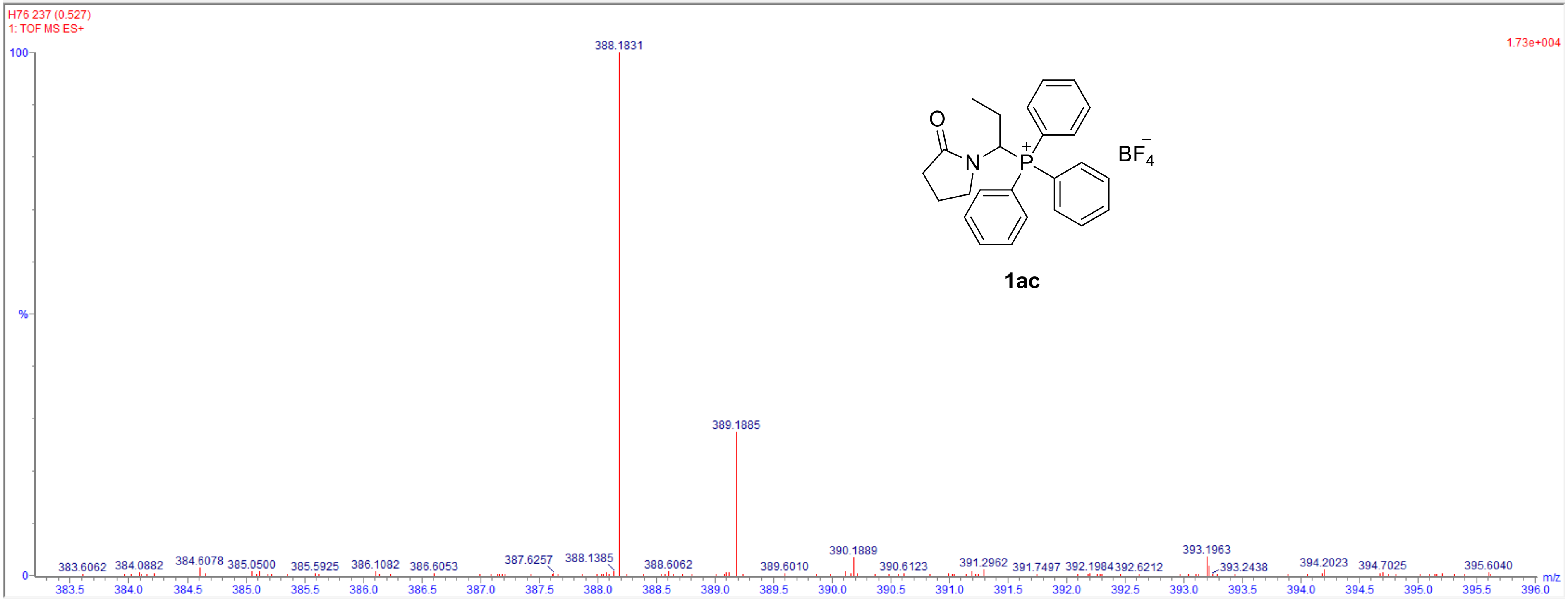

MS spectrum of 1-(2-oxopyrrolidin-1-yl)propyltriphenylphosphonium tetrafluoroborate (**1ac**).

Tolerance = 50.0 mDa / DBE: min = -10.0, max = 50.0
Element prediction: Off
Number of isotope peaks used for i-FIT = 2
Monoisotopic Mass, Even Electron Ions
2407 formula(e) evaluated with 185 results within limits (up to 3 closest results for each mass)
Elements Used:

Mass	RA	Calc. Mass	mDa	PPM	DBE	Formula	i-FIT	i-FIT Norm	Fit Conf %	C	H	N	O	Na	P	S	Cl
360.1518	100.00	360.1517	0.1	0.3	13.5	C23 H23 N O P	375.6	0.526	59.07	23	23	1	1		1		
		360.1519	-0.1	-0.3	13.5	C24 H23 N Cl	376.8	1.732	17.69	24	23	1					1
		360.1527	-0.9	-2.5	5.5	C18 H28 N O Na P S	376.6	1.460	23.23	18	28	1	1	1	1	1	

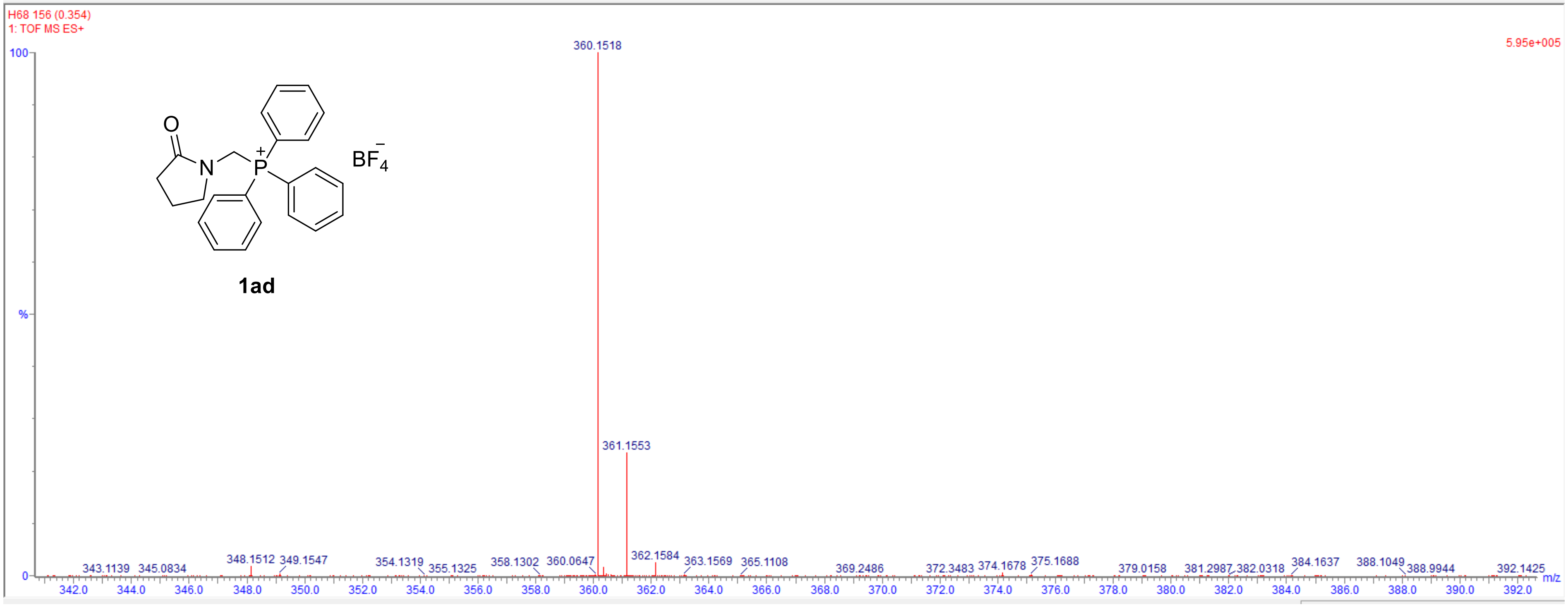

MS spectrum of 1-(2-oxopyrrolidin-1-yl)methyltriphenylphosphonium tetrafluoroborate (**1ad**).

Multiple Mass Analysis: 2 mass(es) processed
Tolerance = 50.0 mDa / DBE: min = -10.0, max = 50.0
Element prediction: Off
Number of isotope peaks used for i-FIT = 2
Monoisotopic Mass, Even Electron Ions
26 formula(e) evaluated with 3 results within limits (up to 3 closest results for each mass)
Elements Used:

Mass	RA	Calc. Mass	mDa	PPM	DBE	Formula	i-FIT	i-FIT Norm	Fit Conf %	C	H	N	O	P
486.1989	100.00	486.1987	0.2	0.4	20.5	C33 ...	3...	0.004	99.64	33	29	1	1	1
		486.2198	-20.9	-43.0	15.5	C30 ...	3...	8.112	0.03	30	33	1	3	1
		486.1623	36.6	75.3	21.5	C32 ...	3...	5.714	0.33	32	25	1	2	1
487.2018	36.25	---												

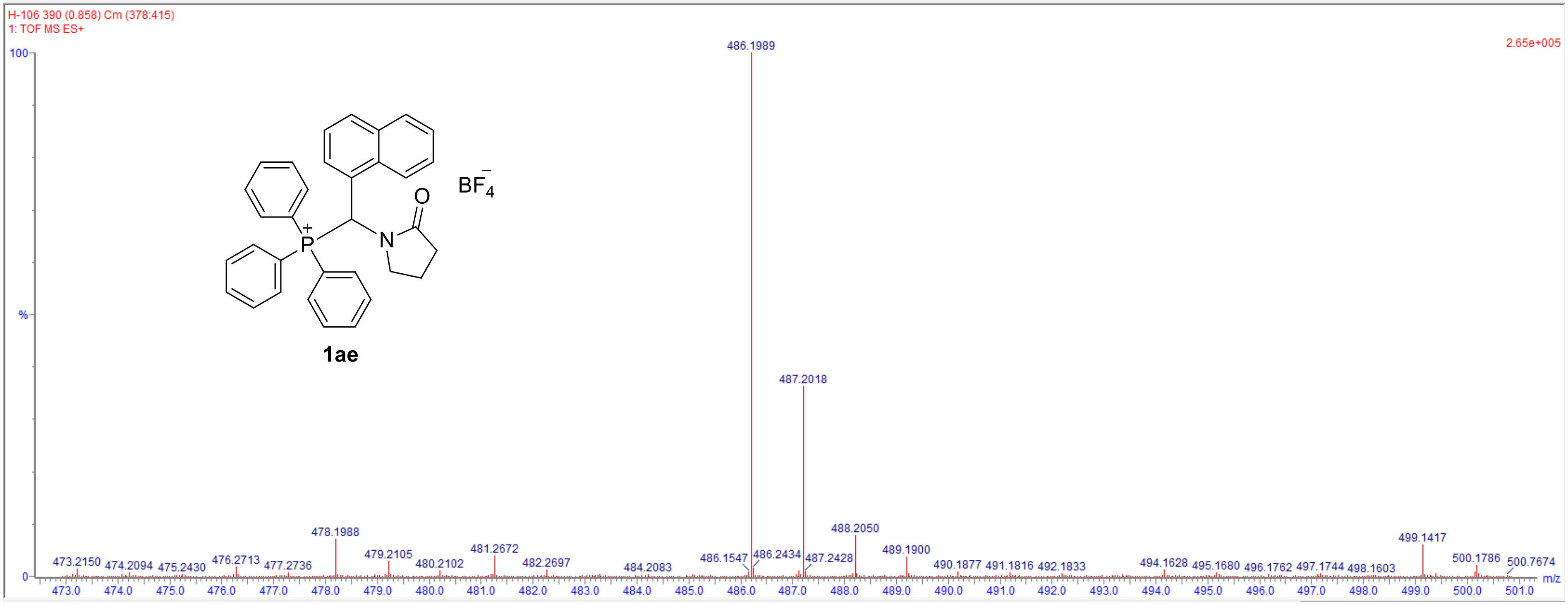

MS spectrum of 1-(2-oxopyrrolidin-1-yl)-1-(1-naphthyl)methyltriphenylphosphonium tetrafluoroborate (**1ae**).

Tolerance = 50.0 mDa / DBE: min = -10.0, max = 50.0
Element prediction: Off
Number of isotope peaks used for i-FIT = 2
Monoisotopic Mass, Even Electron Ions
2446 formula(e) evaluated with 201 results within limits (up to 3 closest results for each mass)
Elements Used:

Mass	RA	Calc. Mass	mDa	PPM	DBE	Formula	i-FIT	i-FIT Norm	Fit Conf %	C	H	N	O	Na	P	S	Cl
450.1624	100.00	450.1625	-0.1	-0.2	18.5	C30 H25 N O Cl	195.2	2.243	10.62	30	25	1	1				1
		450.1623	0.1	0.2	18.5	C29 H25 N O2 P	193.0	0.113	89.27	29	25	1	2		1		
		450.1627	-0.3	-0.7	1.5	C20 H37 N Na P Cl3	199.7	6.826	0.11	20	37	1		1	1		3

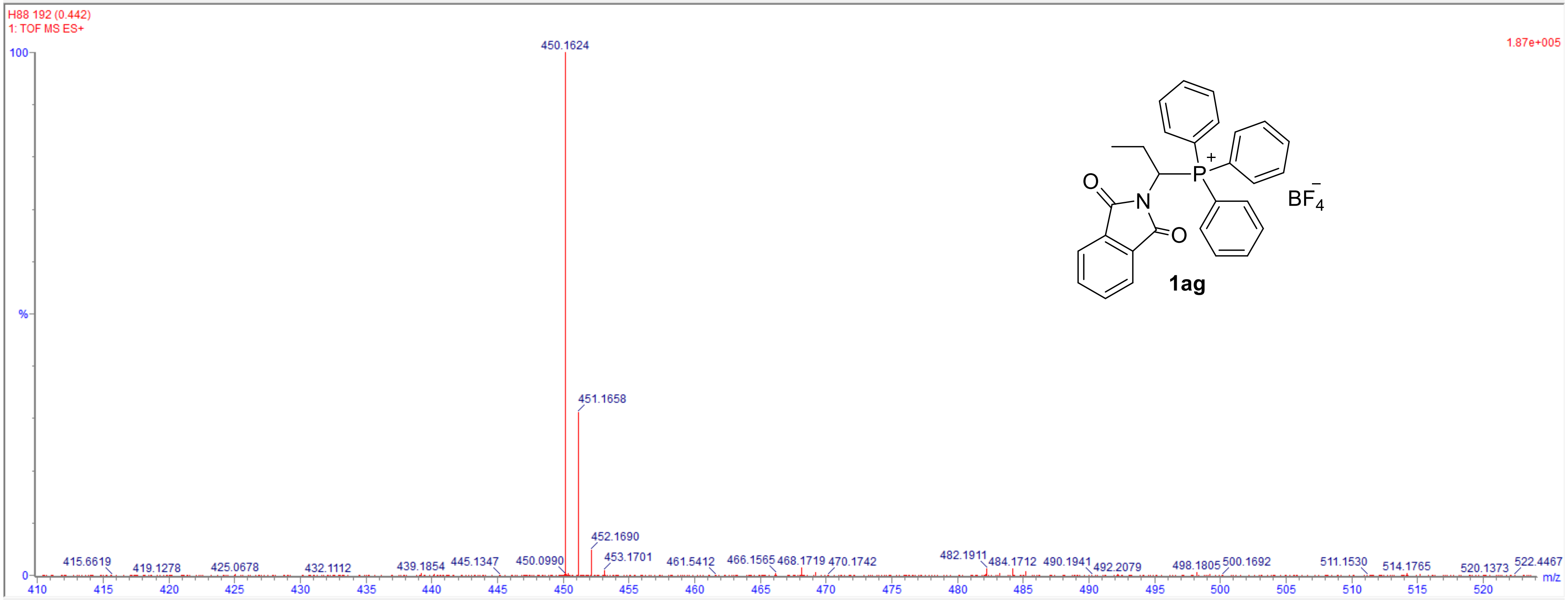

MS spectrum of 1-(*N*-phthalimido)propyltriphenylphosphonium tetrafluoroborate (**1ag**).

Tolerance = 50.0 mDa / DBE: min = -10.0, max = 50.0
Element prediction: Off
Number of isotope peaks used for i-FIT = 2
Monoisotopic Mass, Even Electron Ions
2473 formula(e) evaluated with 195 results within limits (up to 3 closest results for each mass)
Elements Used:

Mass	RA	Calc. Mass	mDa	PPM	DBE	Formula	i-FIT	i-FIT Norm	Fit Conf %	C	H	N	O	Na	P	S	Cl
402.1624	100.00	402.1623	0.1	0.2	14.5	C25 H25 N O2 P	310.8	0.273	76.11	25	25	1	2		1		
		402.1625	-0.1	-0.2	14.5	C26 H25 N O Cl	312.1	1.482	22.71	26	25	1	1				1
		402.1627	-0.3	-0.7	-2.5	C16 H37 N Na P Cl3	315.0	4.436	1.18	16	37	1		1	1		3

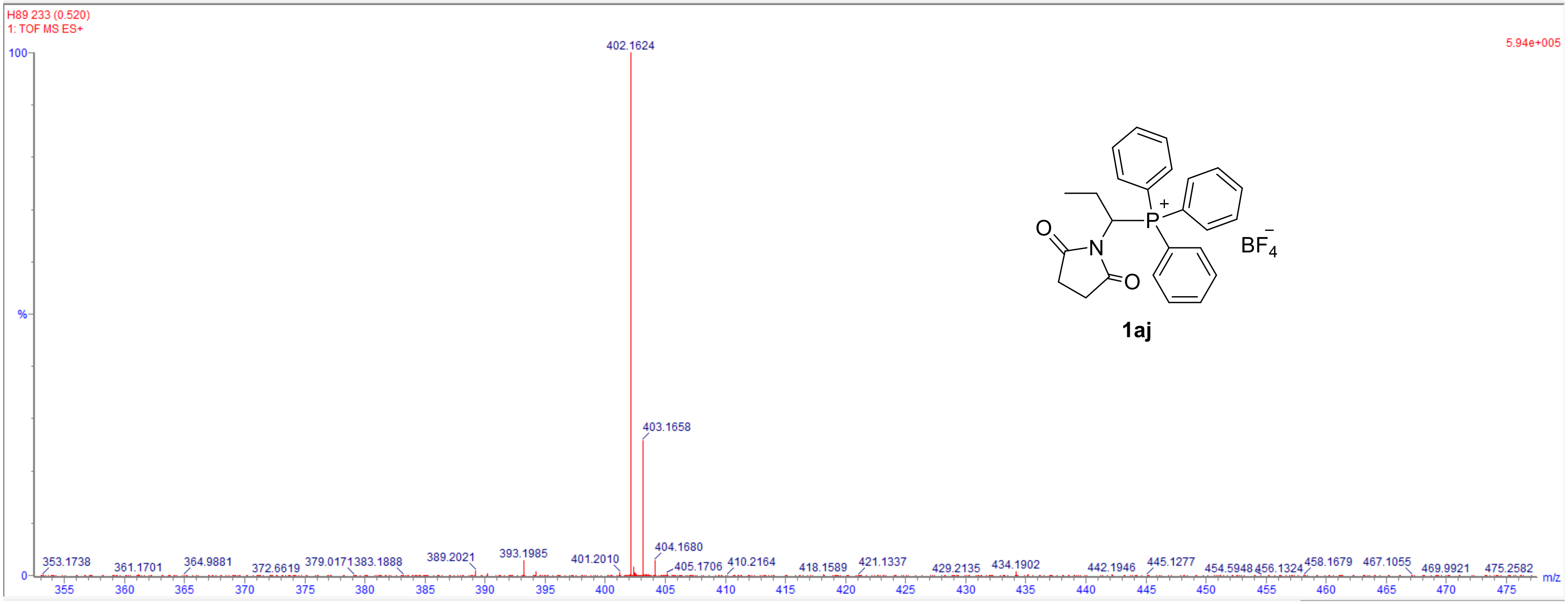

MS spectrum of 1-(*N*-succinimido)propyltriphenylphosphonium tetrafluoroborate (**1aj**).

Tolerance = 50.0 mDa / DBE: min = -10.0, max = 50.0
Element prediction: Off
Number of isotope peaks used for i-FIT = 2
Monoisotopic Mass, Even Electron Ions
2432 formula(e) evaluated with 195 results within limits (up to 3 closest results for each mass)
Elements Used:

Mass	RA	Calc. Mass	mDa	PPM	DBE	Formula	i-FIT	i-FIT Norm	Fit Conf %	C	H	N	O	Na	P	S	Cl
374.1313	100.00	374.1310	0.3	0.8	14.5	C23 H21 N O2 P	498.0	0.243	78.40	23	21	1	2		1		
		374.1312	0.1	0.3	14.5	C24 H21 N O Cl	499.3	1.570	20.80	24	21	1	1				1
		374.1314	-0.1	-0.3	-2.5	C14 H33 N Na P Cl3	502.5	4.830	0.80	14	33	1		1	1		3

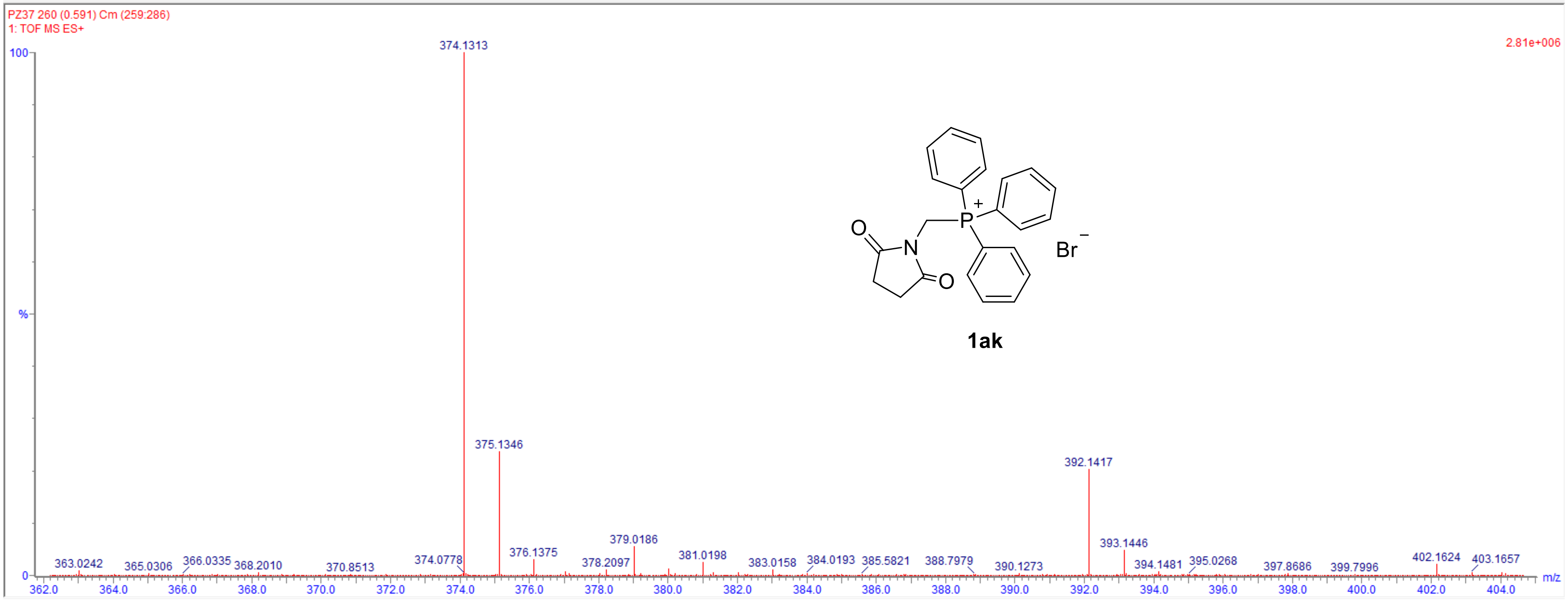

MS spectrum of 1-(*N*-succinimido)methyltriphenylphosphonium bromide (**1ak**).

Tolerance = 20.0 mDa / DBE: min = -10.0, max = 50.0
Element prediction: Off
Number of isotope peaks used for i-FIT = 2
Monoisotopic Mass, Even Electron Ions
4 formula(e) evaluated with 1 results within limits (all results (up to 1000) for each mass)
Elements Used:

Mass	RA	Calc. Mass	mDa	PPM	DBE	Formula	i-FIT	i-FIT Norm	Fit Conf %	C	H	P
263.0995	100.00	263.0990	0.5	1.9	11.5	C18 H16 P	323.7	n/a	n/a	18	16	1

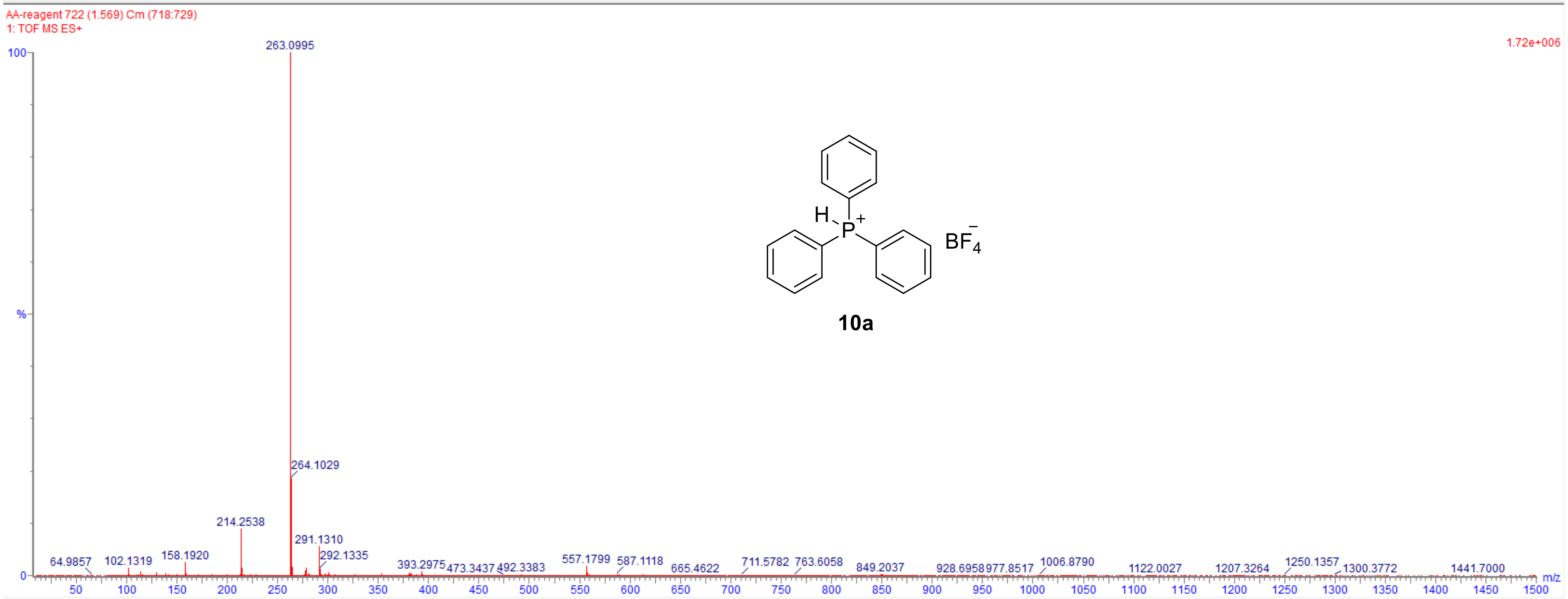

MS spectrum of triphenylphosphonium tetrafluoroborate (**10a**).

Multiple Mass Analysis: 3 mass(es) processed
Tolerance = 20.0 mDa / DBE: min = -10.0, max = 50.0
Element prediction: Off
Number of isotope peaks used for i-FIT = 2
Monoisotopic Mass, Even Electron Ions
79 formula(e) evaluated with 6 results within limits (all results (up to 1000) for each mass)
Elements Used:

Mass	RA	Calc. Mass	mDa	PPM	DBE	Formula	i-FIT	i-FIT Norm	Fit Conf %	C	H	Cl	P
364.9821	100.00	364.9820	0.1	0.3	11.5	C18 H13 Cl3 P	328.2	0.052	94.92	18	13	3	1
		364.9925	-10.4	-28.5	20.5	C24 H7 Cl2	333.6	5.391	0.46	24	7	2	
		364.9692	12.9	35.3	16.5	C21 H8 Cl3	331.2	3.074	4.62	21	8	3	
366.9788	98.73	366.9848	-6.0	-16.3	15.5	C21 H10 Cl3	315.8	4.172	1.54	21	10	3	
		366.9977	-18.9	-51.5	10.5	C18 H15 Cl3 P	311.7	0.016	98.46	18	15	3	1
368.9771	31.96	368.9661	11.0	29.8	24.5	C25 H3 Cl P	349.7	n/a	n/a	25	3	1	1

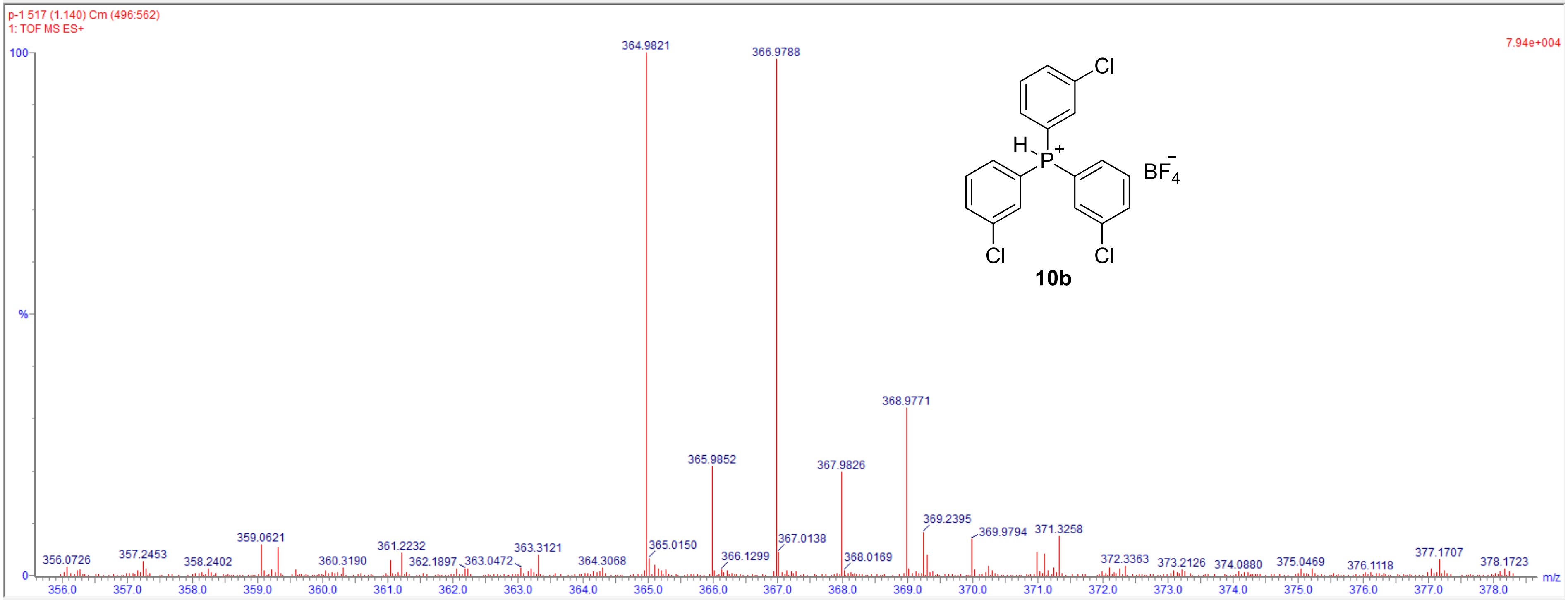

For Help, press F1

MS spectrum of tris(3-chlorophenyl)phosphonium tetrafluoroborate (**10b**).

Tolerance = 100.0 mDa / DBE: min = -10.0, max = 50.0
Element prediction: Off
Number of isotope peaks used for i-FIT = 2
Monoisotopic Mass, Odd and Even Electron Ions
17 formula(e) evaluated with 7 results within limits (up to 5 closest results for each mass)
Elements Used:

Mass	RA	Calc. Mass	mDa	PPM	DBE	Formula	i-FIT	i-FIT Norm	Fit Conf %	C	H	O	P
353.1307	100.00	353.1307	0.0	0.0	11.5	C21 H22 O3 P	460.7	0.058	94.36	21	22	3	1
		353.1095	21.2	60.0	16.5	C24 H18 O P	465.7	5.076	0.62	24	18	1	1
		353.1670	-36.3	-102.8	10.5	C22 H26 O2 P	463.8	3.168	4.21	22	26	2	1
		353.0731	57.6	163.1	17.5	C23 H14 O2 P	465.9	5.279	0.51	23	14	2	1
		353.2034	-72.7	-205.9	9.5	C23 H30 O P	466.4	5.822	0.30	23	30	1	1

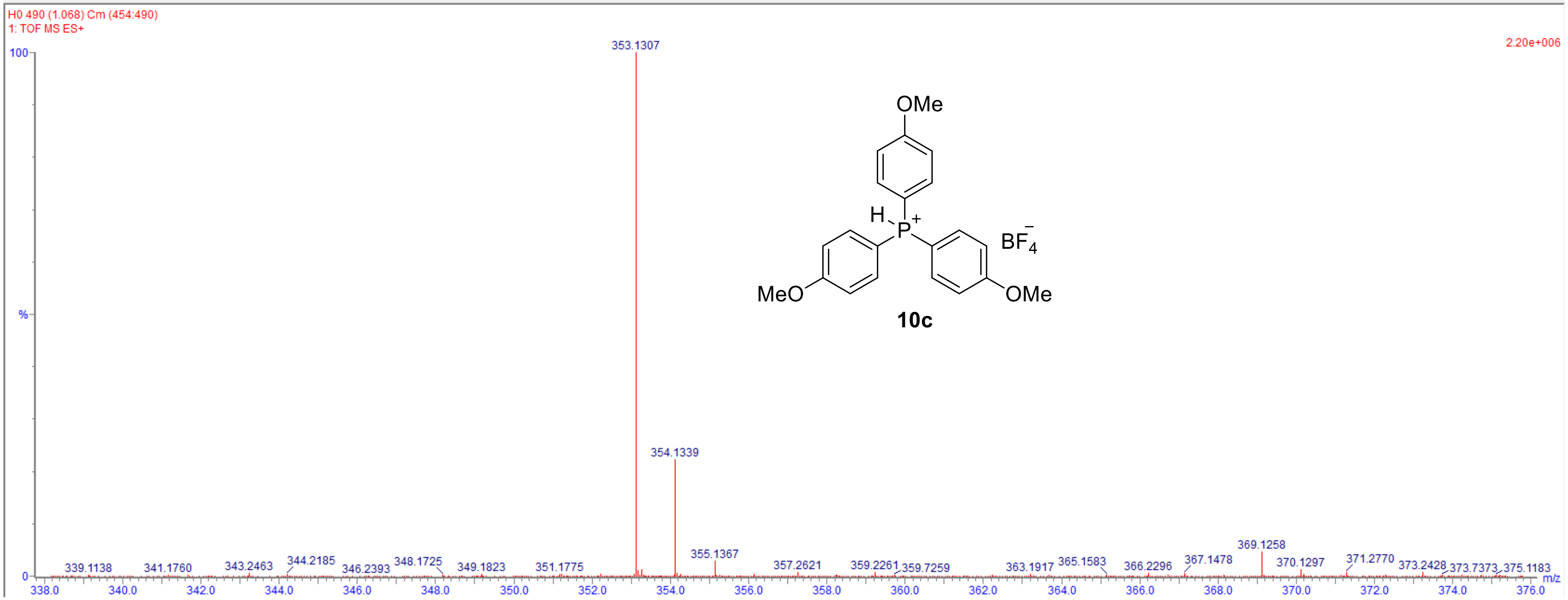

MS spectrum of tris(4-methoxyphenyl)phosphonium tetrafluoroborate (**10c**).

Tolerance = 100.0 mDa / DBE: min = -10.0, max = 50.0
Element prediction: Off
Number of isotope peaks used for i-FIT = 2
Monoisotopic Mass, Even Electron Ions
15 formula(e) evaluated with 7 results within limits (up to 5 closest results for each mass)
Elements Used:

Mass	RA	Calc. Mass	mDa	PPM	DBE	Formula	i-FIT	i-FIT Norm	Fit Conf %	C	H	O	P
321.1408	100.00	321.1408	0.0	0.0	11.5	C21 H22 O P	364.5	5.214	0.54	21	22	1	1
		321.1620	-21.2	-66.0	6.5	C18 H26 O3 P	365.9	6.640	0.13	18	26	3	1
		321.1044	36.4	113.3	12.5	C20 H18 O2 P	359.3	0.015	98.54	20	18	2	1
		321.1983	-57.5	-179.0	5.5	C19 H30 O2 P	364.4	5.128	0.59	19	30	2	1
		321.0681	72.7	226.4	13.5	C19 H14 O3 P	365.5	6.273	0.19	19	14	3	1

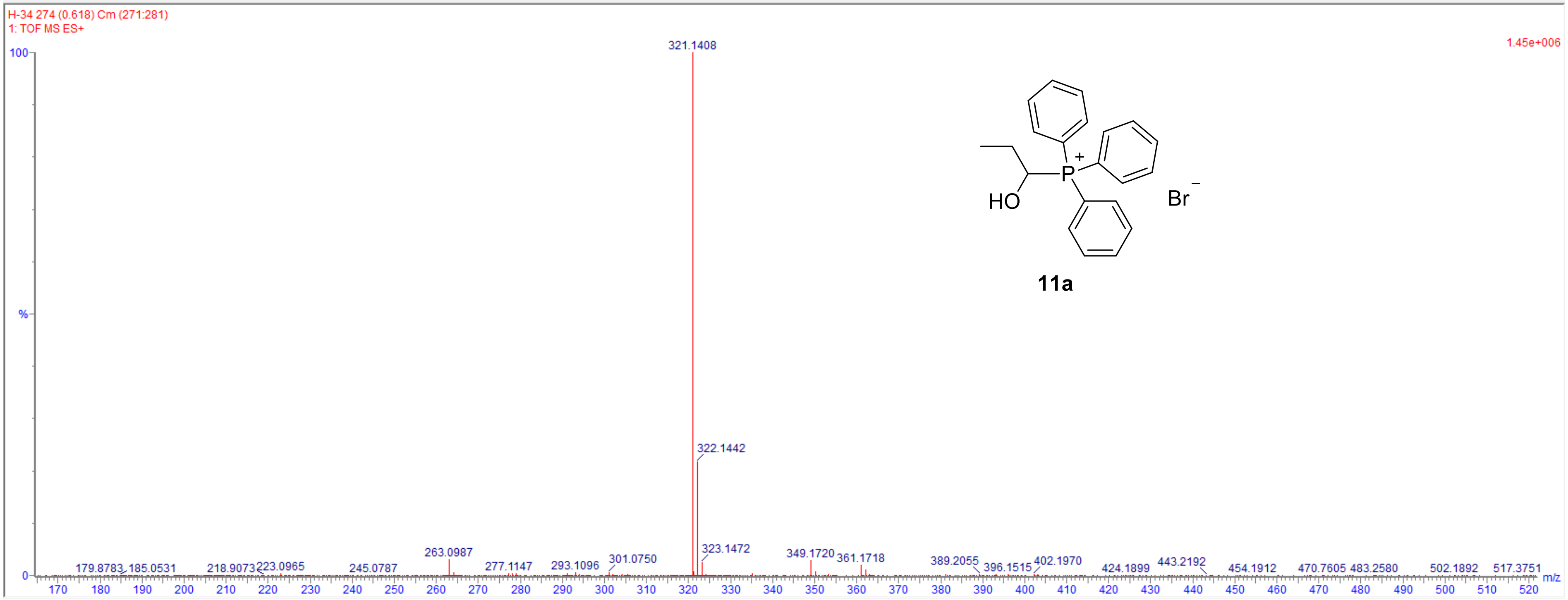

MS spectrum of 1-hydroxypropyltriphenylphosphonium bromide (**11a**).

Tolerance = 50.0 mDa / DBE: min = -10.0, max = 50.0
Element prediction: Off
Number of isotope peaks used for i-FIT = 2
Monoisotopic Mass, Even Electron Ions
43 formula(e) evaluated with 11 results within limits (up to 3 closest results for each mass)
Elements Used:

Mass	RA	Calc. Mass	mDa	PPM	DBE	Formula	i-FIT	i-FIT Norm	Fit Conf %	C	H	N	O	P
334.1366	100.00	334.1361	0.5	1.5	12.5	C21 H21 N O P	353.1	0.605	54.62	21	21	1	1	1
		334.1443	-7.7	-23.0	12.5	C21 H20 N O3	353.3	0.825	43.83	21	20	1	3	
		334.1232	13.4	40.1	17.5	C24 H16 N O	356.6	4.167	1.55	24	16	1	1	

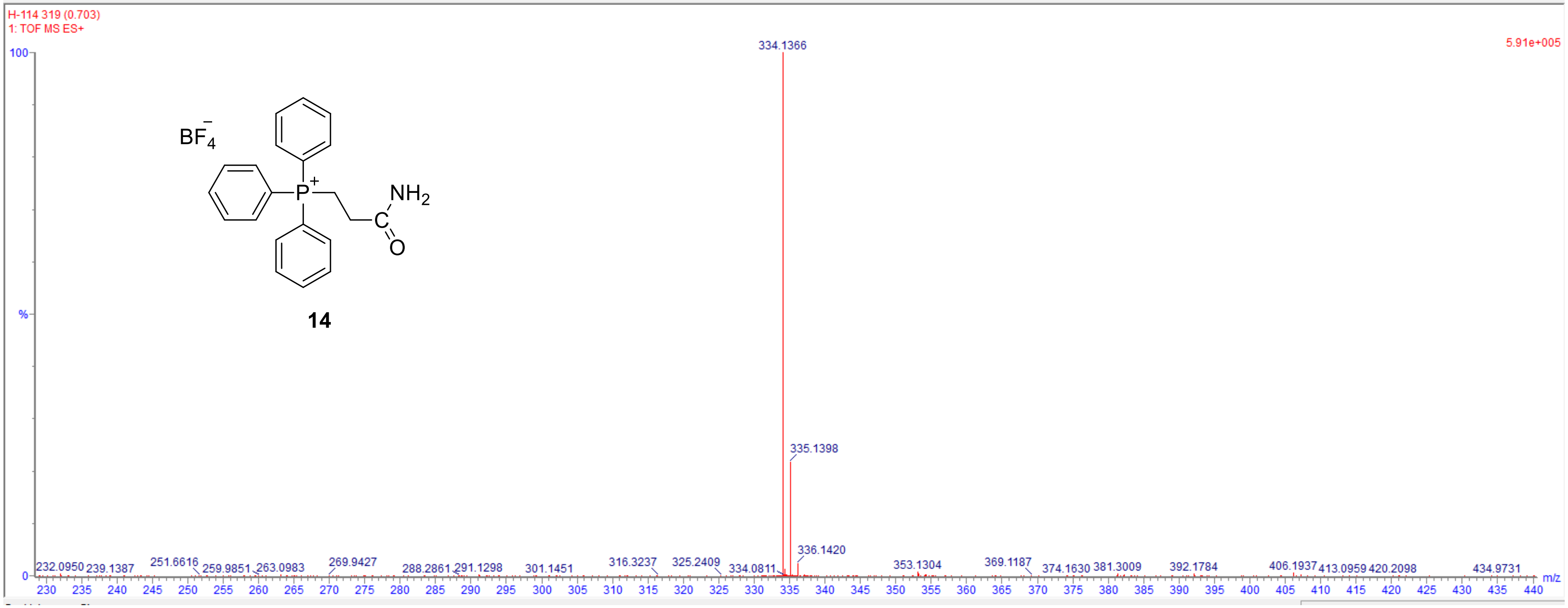

MS spectrum of 2-carbamoyletyhyltriphenylphosphonium tetrafluoroborate (**14**).

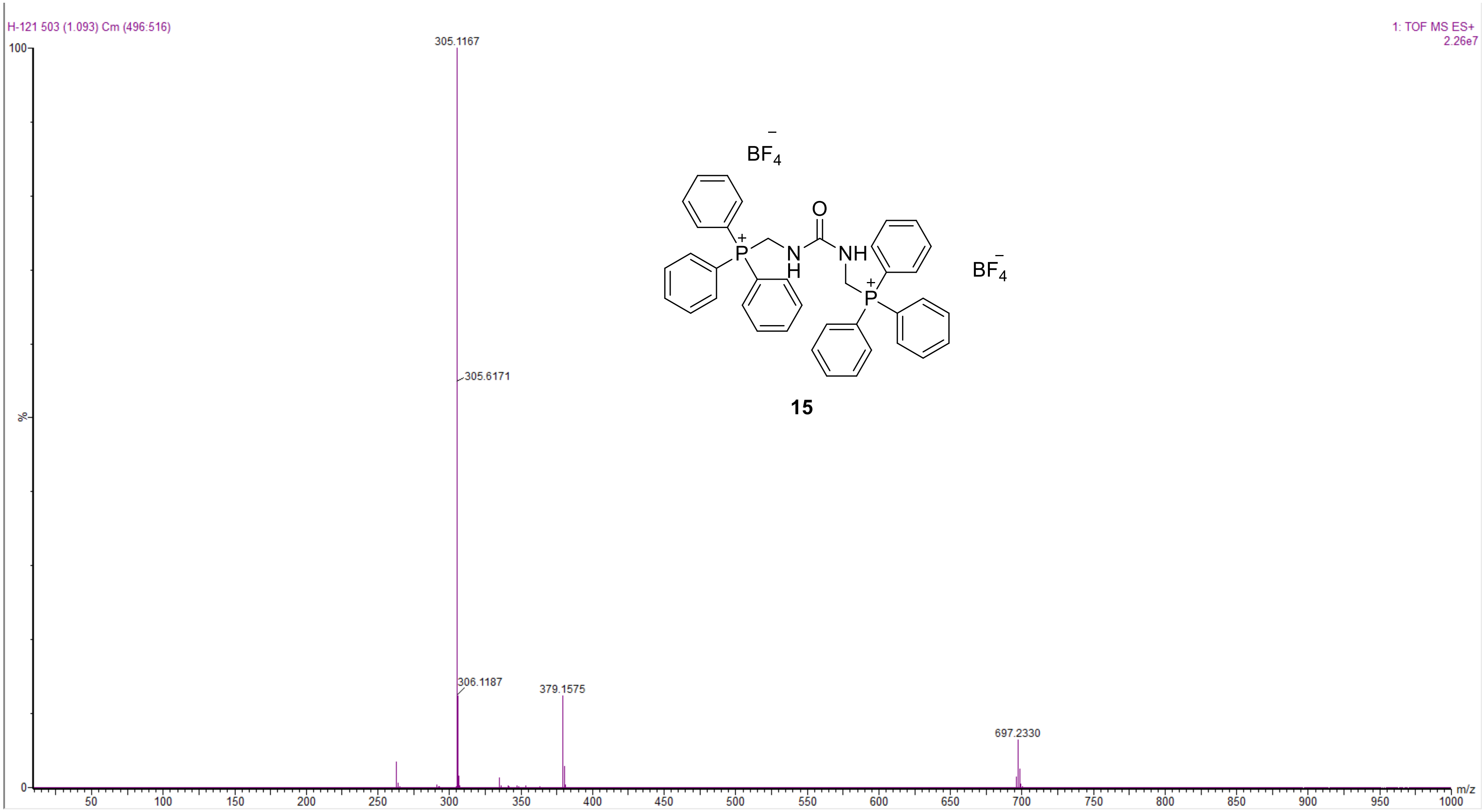

MS spectrum of 1,1’-(carbonyldimino)bis(methyltriphenylphosphonium) bis(tetrafluoroborate) (**15**).
